# Supplementary material for: A phase I open-label clinical trial to study drug-drug interactions of Dorzagliatin and Sitagliptin in patients with type 2 diabetes and obesity
Source: Nat Commun. 2023 Mar 14;14:1405. doi: 10.1038/s41467-023-36946-7 (PMC10014962; doi:10.1038/s41467-023-36946-7)
Supplement: Supplementary file 1 — Supplementary Information [file 41467_2023_36946_MOESM1_ESM.pdf]

**Supplementary Methods**

**Materials and Methods**

**PK and PD sample preparation**

For pharmacokinetics analysis, blood samples were collected with dipotassium ethylenediaminetetra acetic acid ($K_2EDTA$) tubes, and plasma samples were prepared by centrifugation for 10 min at approximately 2000 g and 4°C.

For measuring of serum glucose and C-peptide, blood samples were collected with Serum Separator Tubes, and obtained serum samples following centrifugation for 10-15 min at approximately 1100-1300 g after standing for a minimum of 30 minutes.

For measuring of plasma GLP-1, blood samples were collected into Becton Dickenson BD P800 tubes that contained $K_2EDTA$ and proprietary additives. Plasma samples were prepared by centrifugation for 20 min at approximately 1100-1300 g.

All plasma samples obtained were stored at -70°C or lower until shipment on dry ice for analysis. Serum samples were refrigerated prior to analysis.

**Analytical methods**

- **Dorzagliatin and sitagliptin**

Dorzagliatin and sitagliptin concentrations in plasma were determined by validated liquid chromatography-tandem mass spectrometry (LC-MS/MS) methods (Frontage Laboratories, Inc., Pennsylvania, USA). The concentrations were calculated by using peak area ratios and linear regression with $1/x^2$ weighting to generate the best-fit concentration-response relationship.

For Dorzagliatin, the lower limit of quantitation (LLOQ) in plasma was 1 ng/mL with linearity to 1200 ng/mL; the accuracy of this assay was between 0.0 – 8.3%, and the precision was ≤6.1%. For sitagliptin, the LLOQ of was 1 ng/mL, with linearity to 1000 ng/mL; the accuracy of this assay was between 0.7 – 9.7%, and the precision was ≤9.0%.

No obvious interference was observed in human plasma between sitagliptin and dorzagliatin. The stability of both analytes in human plasma was validated to cover the period from sample collection to sample analysis. It was proved that the LC-MS/MS methods demonstrated acceptable accuracy, precision, and stability under different conditions for both analytes.

- **GLP-1**

GLP-1 in human plasma was determined by well-validated enzyme-linked immunosorbent assays at Mercordia (Uppsala, Sweden). Mercodia Total GLP-1 NL-ELISA kit (10-1278-01) was employed for Total GLP-1 measurement, with validated LLOQ of 1.7 pmol/L and ULOQ of 940 pmol/L. Millipore High Sensitivity Active GLP-1 Chemiluminescent ELISA kit (EZGLPHS-35K) was used for active GLP-1 measurement, with validated LLOQ of 1.32 pmol/L and ULOQ of 74.52 pmol/L. The concentration of GLP-1 was interpolated using five parameter Marquardt logistic equation model.

Both assays had good relative accuracy and precision.

## Supplementary Figures

**Figure S1. Overall study design in the studies**. (CRC=clinical research center)

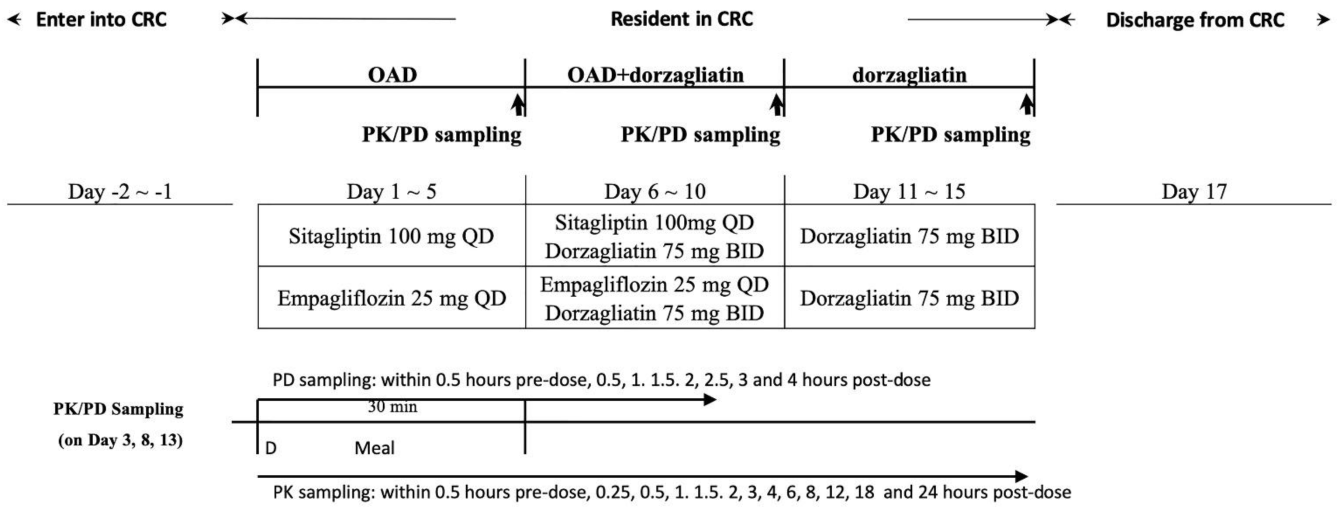

**Figure S2. Forest plot of dorzagliatin and sitagliptin drug-drug interaction assessment of PK parameters** (sitagliptin, n=14; sitagliptin+dorzagliatin, n=14; dorzagliatin, n=14) **Upper part represent the geometric means ratio (GMR) and 90% confidence interval (90%CI) for $AUC_{0-24h}$ (filled circle) and $C_{max}$ (filled triangle) of combination therapy (sitagliptin+dorzagliatin) to sitagliptin monotherapy. Lower part represent the GMR and 90%CI for $AUC_{0-24h}$ (filled circle) and $C_{max}$ (filled triangle) by comparing combination therapy (sitagliptin+dorzagliatin) to dorzagliatin monotherapy. The horizontal lines represent 90% confidence interval. The dotted lines indicate bioequivalence limits of 80% and 125%.**

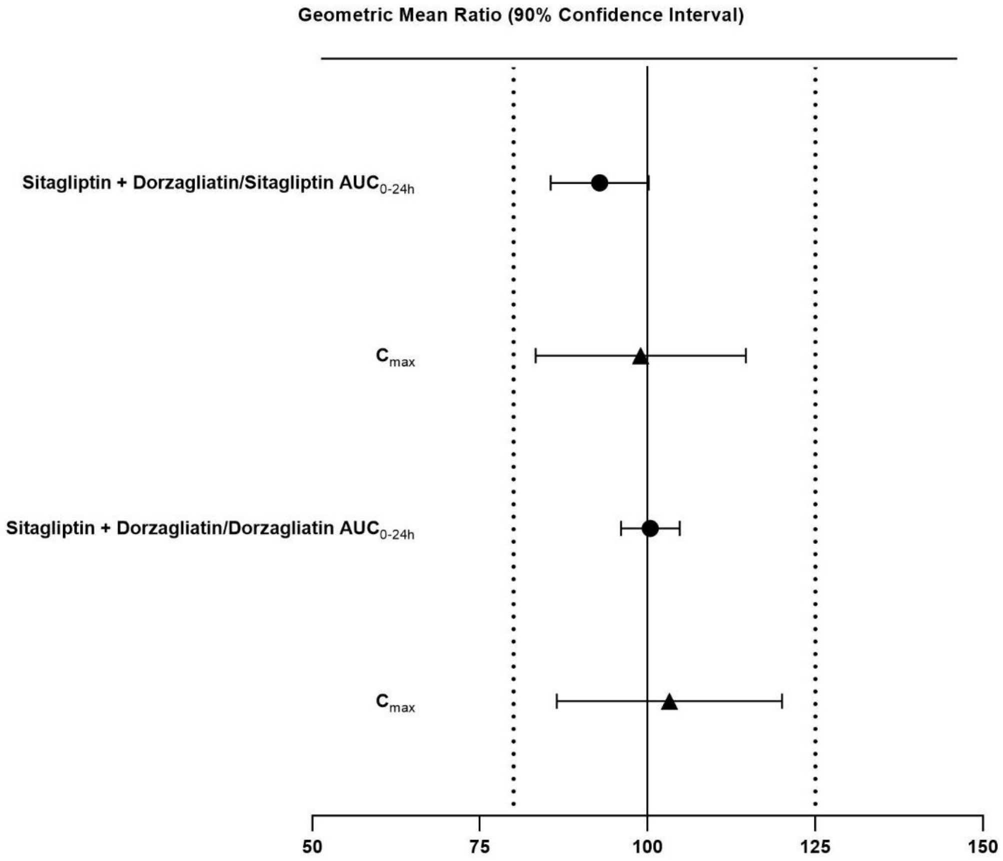

**Figure S3. (a) Glucose** (sitagliptin, n=15; sitagliptin+dorzagliatin, n=15; dorzagliatin, n=14)**, (b) C-peptide** (sitagliptin, n=15; sitagliptin+dorzagliatin, n=15; dorzagliatin, n=14), **(c) GLP-1$_{total}$** (sitagliptin, n=15; sitagliptin+dorzagliatin, n=15; dorzagliatin, n=14) **and (d) GLP-1$_{active}$** (sitagliptin, n=11; sitagliptin+dorzagliatin, n=11; dorzagliatin, n=10) **concentration-time curves under sitagliptin, sitagliptin+dorzagliatin, and dorzagliatin therapy. Empty square line represents dorzagliatin monotherapy, filled circle line represents dorzagliatin+sitagliptin therapy, empty triangle line represents sitagliptin monotherapy. Data are presented as mean values +/- SD. Error bars represent standard deviation.**

**a. Glucose**

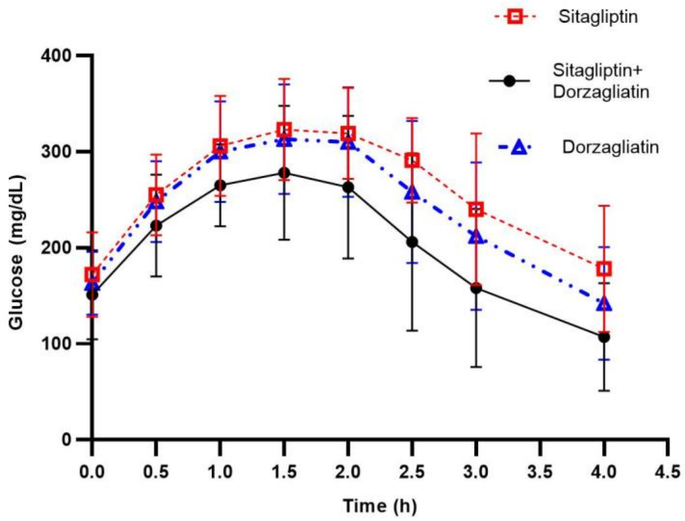


**b. C-peptide**

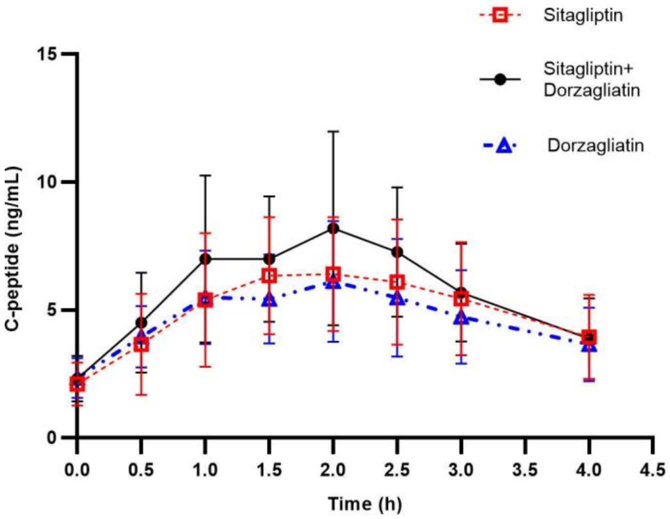

**c. GLP-1$_{total}$**

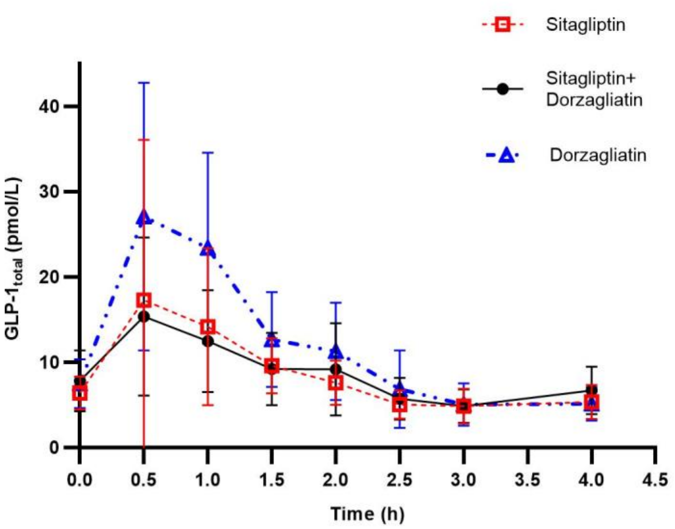


**d. GLP-1$_{active}$**

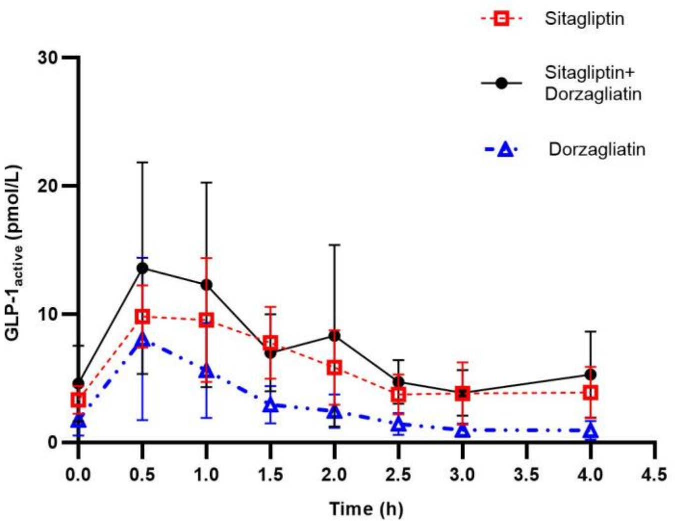

**Figure S4. Pharmacokinetic dorzagliatin exposure-time curve and GLP-1 concentration-time curve of dorzagliatin (Dorza) and sitagliptin+dorzagliatin (Dorza+Sita) therapy. Filled circle line represents dorzagliatin exposure under dorzagliatin+sitagliptin (n=15) therapy, filled triangle line represents dorzagliatin exposure under dorzagliatin (n=14) monotherapy, half-filled circle line represents GLP-1$_{total}$ concentration under dorzagliatin+sitagliptin (n=15) therapy, half-filled triangle line represents GLP-1$_{total}$ concentration under dorzagliatin (n=14) monotherapy, empty circle line represents GLP-1$_{active}$ concentration under dorzagliatin+sitagliptin (n=10) therapy, empty triangle line represents GLP-1$_{active}$ concentration under dorzagliatin (n=10) monotherapy**

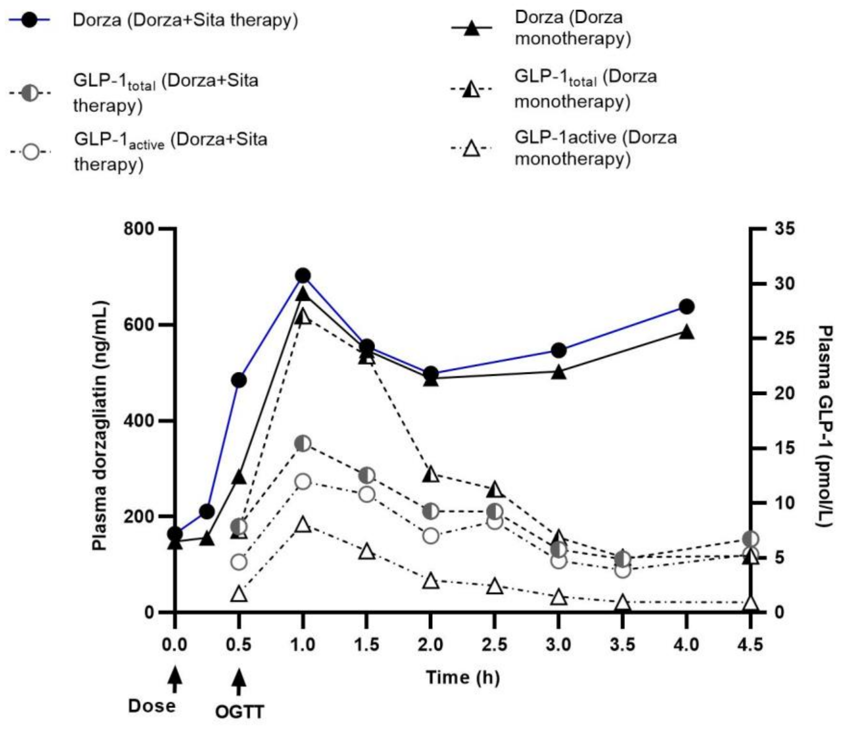

**Supplementary Table**

**Table S1. Adverse events and hypoglycaemic events (safety population)**

| | Sitagliptin (N=15) | Dorzagliatin (N=15) | Sitagliptin +Dorzagliatin (N=15) |
|---|---|---|---|
| TEAE | 5 (33.3) | 4 (26.7) | 4 (26.7) |
| Headache | 2 (13.3) | 2 (13.3) | 1 (6.7) |
| Rash | 0 | 1 (6.7) | 1 (6.7) |
| Dermatitis | 1 (6.7) | 0 | 0 |
| Pruritus | 0 | 0 | 1 (6.7) |
| Rash erythematous | 0 | 1 (6.7) | 0 |
| Constipation | 1 (6.7) | 0 | 0 |
| Abdominal pain upper | 0 | 0 | 0 |
| Nausea | 0 | 0 | 1 (6.7) |
| Vomiting | 0 | 0 | 0 |
| Vision blurred | 0 | 0 | 0 |
| Dizziness | 0 | 0 | 0 |
| Syncope | 0 | 0 | 0 |
| Anemia | 0 | 0 | 0 |
| Tinnitus | 0 | 0 | 0 |
| Chest pain | 0 | 0 | 0 |
| Hunger | 0 | 0 | 1 (6.7) |
| Glycosuria | 1 (6.7) | 0 | 1 (6.7) |
| Pruritus genital | 0 | 0 | 0 |
| Nasal congestion | 1 (6.7) | 0 | 0 |
| Hyperkalemia | 0 | 0 | 0 |
| Severe TEAE | 0 | 0 | 0 |
| SAE | 0 | 0 | 0 |

| | | | |
|---|---|---|---|
| Possibly or probably related | 1 (6.7) | 2 (13.3) | 3 (20.0) |
| Discontinuation due to AE | 0 | 1 (6.7) | 0 |
| Hypoglycemia | 0 | 0 | 1 (6.7) |
| Blood glucose < 54 mg/dL (3.0 mmol/L) | 0 | 0 | 1 (6.7) |

Values are presented as n (%). n (%) = number and percent of subjects in the specified group.

AE=adverse event. TEAE = treatment emergent adverse event. SAE=serious adverse event. Adverse events were considered treatment-emergent if not present prior to initiation of the treatment with study drug on Day 1 or already present but worsened in either severity or frequency following exposure to the treatment.

**Supplementary note**

Study protocol and amendment:

i. Protocol Version 1.3, Version Date: 05 March 2019
ii. Protocol Version 1.2, Version Date: 04 February 2019
iii. Protocol Version 1.1, Version Date: 27 December 2018
iv. Protocol Version 1.0, Version Date: 11 November 2018

## 16.1.1 PROTOCOL AND PROTOCOL AMENDMENTS

The following original protocol and protocol amendment versions were reviewed and approved by Interview IRB and were implemented for this study:

Protocol Version 1.3, Version Date: 05 March 2019

Protocol Version 1.2, Version Date: 04 February 2019

Protocol Version 1.1, Version Date: 27 December 2018

Protocol Version 1.0, Version Date: 11 November 2018

# TABLE OF CONTENTS

TABLE OF CONTENTS ... 3
1. PROTOCOL SYNOPSIS ... 9
2. LIST OF ABBREVIATIONS ... 15
3. INTRODUCTION ... 18
3.1 Background Information ... 18
3.2 Preclinical Data ... 19
3.3 Summary of Clinical Studies ... 20
3.4 Drug Interaction Potential of Dorzagliatin ... 23
4. STUDY RATIONALE AND OBJECTIVES ... 25
4.1. Study Rationale ... 25
4.2. Primary Objectives ... 25
4.3. Secondary Objective ... 26
5. INVESTIGATIONAL PLAN ... 26
5.1. Overall Study Design ... 26
5.2. Number of Subjects ... 30
5.3. Treatment Assignment ... 30
5.4. Criteria for Study Termination ... 30
6. SELECTION AND WITHDRAWAL OF SUBJECTS ... 30
6.1. Subject Inclusion Criteria ... 30
6.2. Subject Exclusion Criteria ... 32
6.3. Prohibitions and Restrictions ... 34
6.4. Subject Withdraw Criteria ... 34
7. STUDY PROCEDURES ... 35
7.1. Screening Visit and Run-in Period ... 36
7.2. Domiciled at CRC on Day -2 through Day 17 ... 37
7.2.1. Study Check-in Visit: Day -2 and Day -1 ... 37

7.2.2. Day 1 - Day 4 ... 38

7.2.3. Day 5 ... 39

7.2.4. Day 6 - Day 9 ... 39

7.2.5. Day 10 ... 40

7.2.6. Day 11 - Day 14 ... 40

7.2.7. Day 15 ... 41

7.2.8. Day 16 ... 42

7.2.9. Day17: End-of-Study Procedures ... 42

7.3. Early Withdrawal Visit ... 42

7.4. Time Windows for Procedures ... 42

8. TREATMENT OF SUBJECTS ... 43

8.1. Description of Study Drugs ... 43

8.2. Methods of Assigning Subjects to Dose Groups ... 43

8.3. Blinding ... 44

8.4. Treatment Discontinuation/Subject Withdrawal ... 44

8.5. Prior and Concomitant Medications and Substances ... 44

8.6. Meals ... 44

9. STUDY DRUG MATERIALS AND MANAGEMENT ... 45

9.1. Study Drug Packaging and Labeling ... 45

9.2. Study Drug Storage and Accountability ... 45

9.3. Study Drug Preparation ... 46

9.4. Study Drug Handling and Disposal ... 46

10. PHARMACOKINETIC/PHARMACODYNAMIC ASSESSMENTS ... 46

10.1. Pharmacokinetic Sample Collection and Storage ... 46

10.2. Pharmacokinetic Specimen Labeling ... 47

10.3. Pharmacokinetic Sample Shipping Instructions ..... 47
10.4. Bioanalytical Methodology ..... 47
10.5. Pharmacokinetic Parameters ..... 47
10.6. Pharmacodynamic Assessments ..... 48
11. ASSESSMENT OF SAFETY ..... 49
11.1. Safety Parameters ..... 49
11.1.1. Demographic/Medical History ..... 49
11.1.2. Vital Signs ..... 49
11.1.3. Physical Examination, Height, Weight, and Body Mass Index ..... 50
11.1.4. Electrocardiogram ..... 50
11.1.5. Clinical Laboratory Assessments ..... 50
11.2. Adverse Events ..... 52
11.2.1. Definitions of Adverse Events ..... 52
11.2.2. Recording Adverse Events ..... 55
11.2.3. Reporting of Serious Adverse Events ..... 55
11.2.4. Adverse Event Follow-up ..... 56
11.2.5. Hy's Law ..... 56
11.2.6. Hypoglycemia ..... 56
12. STATISTICAL METHODOLOGY ..... 57
12.1. Sample Size Determination ..... 57
12.2. Analysis Population ..... 57
12.3. Pharmacokinetics and Pharmacodynamics ..... 58
12.4. Demographic Characteristics ..... 59
12.5. Exposure to Study Drugs ..... 59
12.6. Concomitant Medications ..... 59

12.7. Safety Analyses ... 59
12.8. Interim Analyses ... 60
13. DIRECT ACCESS TO SOURCE DATA/DOCUMENTS ... 60
13.1. Study Monitoring ... 60
13.2. Sponsor's Responsibility ... 60
13.3. Audits and Inspections ... 61
14. QUALITY CONTROL AND QUALITY ASSURANCE ... 61
15. ETHICS ... 62
15.1. Ethics Review ... 63
15.2. Ethical Conduct of the Study ... 64
15.3. Written Informed Consent ... 64
16. DATA HANDLING AND RECORD KEEPING ... 65
16.1. Data Collection ... 65
16.2. Case Report Form Completion ... 65
16.3. Database Management, Data Clarification, and Quality Assurance ... 65
16.4. Inspection of Records/Source Documents ... 66
16.5. Retention of Records ... 67
17. CONFIDENTIALITY ... 68
17.1. Subject Data Protection ... 68
18. STUDY PROTOCOL AMENDMENTS ... 69
19. PUBLICATION ... 70
20. REFERENCES ... 70
APPENDIX A. KNOWN CLINICAL INHIBITORS AND INDUCERS FOR CYP3A4* ... 72
APPENDIX B. LABORATORY ASSESSMENTS ... 74

# TABLES

Table 5-1 Schedule of Procedures ... 28
Table 5-2: Treatment Assignment and PK Sample Collection ... 30

Table 10-1: PK Terms........................................................................................................ 48

## Summary of Changes

| Change from Version 1.2 (04 February 2019) to Version 1.3 (05 March 2019) | |
|---|---|
| **Section** | **Change** |
| 1.0 Synopsis<br>6.2 Subject Exclusion Criteria | Exclusion Criterion #27<br>Change from:<br>27. Use of prescription or over-the-counter (OTC) medications, and herbal (including St John's Wort, herbal teas, garlic extracts) within 14 days prior to dosing (except for sitagliptin dosing during run-in period). No angiotensin-converting enzyme (ACE) inhibitors are allowed; (Note: Use of acetaminophen at <1 g/day is permitted until 24 hours prior to dosing);<br>To:<br>27. Use of herbal (including St John's Wort, herbal teas, garlic extracts) within 14 days prior to dosing. No drugs known to be CYP3A4 inhibitors or inducers are allowed (See Appendix A for list of known CYP3A4 inhibitors or inducers). Use of other prescription and/or OTC medications within 14 days must be discussed and approved by the sponsor and the study PI prior to entry into the study. |
| 6.3 Prohibitions and Restrictions | Deleted the following two bullet points:<br>• No administration of any prescription medications (with the exception of study drug) per Exclusion Criteria #27;<br>• No use of any OTC products (with the exception of acetaminophen <1 g/day until 24 hours prior to dosing) per Exclusion Criteria #27; |
| Change from Version 1.1 (27 December 2018) to Version 1.2 (04 February 2019) | |
| **Section** | **Change** |
| 1.0 Synopsis<br>6.1 Subject Inclusion Criteria | Inclusion Criterion #1 clarified to avoid misinterpretation. Deleted word "within", changing from "Subjects diagnosed with T2DM **within** at least 3 months prior to screening and are currently taking a stable dose…." to "Subjects diagnosed with T2DM at least 3 months prior to screening and are currently taking a stable dose….". |
| Main changes from Version 1.0 (11 November 2018) to Version 1.1 (27 December 2018) | |
| **Section** | **Change** |
| 1.0 Synopsis<br>4.3 Secondary Objective, and | Wording of secondary objective changed to remove "serum" since some PD analytes are measured in plasma and some are measured in serum. |

| | |
|---|---|
| throughout protocol. | Throughout protocol, when matrix of PD analytes is noted, wording changed from “serum levels of GLP-1, glucagon, glucose, insulin, and C-peptide” to “GLP-1 and glucagon in plasma, and glucose, insulin, and C-peptide in serum”. |
| 5.1 Overall Study Design | Schedule of Procedures footnote to Run-in Period amended to reflect that sitagliptin may be dispensed to eligible subjects prior to Day -14. |
| 5.2 Number of Subjects | Changed “Evaluable subjects are defined as subjects included in the **PD** population…” to “Evaluable subjects are defined as subjects included in the **DDI** population…” |
| 6.1 Subject Inclusion Criteria | Removed “screening” to clarify that criteria applies to screening and/or Day -1. |
| 6.2 Subject Exclusion Criteria | Removed “screening” to clarify that criteria applies to screening and/or Day -1. |
| 7.2.1 Study Check-in Visit | Added physical examination on Day -1 to be consistent with remainder of protocol. |
| 7.2.3-7.2.9 (Day 5-Day 17) | Changed 18-hour post-dose PK blood draw to occur on day after dosing.<br>Removed blood glucose assessment by glucometer on Day 17 to be consistent with remainder of protocol. |
| 10.6 Pharmacodynamic Assessments | Changed volume of blood collected for PD measurements: 8.5 mL (instead of 8.0 mL) collected for measurement of plasma GLP-1 and glucagon, and 7 mL (instead of 6 mL) collected for measurement of serum glucose, insulin and C-peptide.<br>Sample collection and processing steps for measurement of GLP-1 and glucagon added. |

# 1. PROTOCOL SYNOPSIS

| | |
|---|---|
| **Name of Sponsor/Company:**<br>Hua Medicine (Shanghai) Ltd. | |
| **Name of Investigational Product:**<br>Dorzagliatin | |
| **Name of Active Ingredient:**<br>Dorzagliatin: (S)-2-[4-(2-Chloro-phenoxy)-2-oxo-2,5-dihydro-pyrrol-1-yl]-4-methyl-pentanoic acid [1-((R)-2,3-dihydroxy-propyl)-1H-pyrazol-3-yl]-amide | |
| **Title of Study:**<br>A Phase 1, Open-Label, Sequential, Multiple-Dose, Drug-Drug Interaction Study of Dorzagliatin and Sitagliptin in Subjects with Type 2 Diabetes Mellitus | |
| **Study Center(s):** | |
| **Site:** Frontage Clinical Services, Inc., Secaucus, NJ<br>**Principal Investigator:** [redacted]<br>**Sub-investigator:** [redacted] | **Site:** To be Determined |
| **Studied Period (years):**<br>Estimated date first subject enrolled: Dec 2018<br>Estimated date last subject completed: Mar 2019 | **Phase of development:** Phase 1 |
| **Objectives:**<br>**Primary:**<br>• To assess the potential pharmacokinetic interaction between dorzagliatin and sitagliptin in subjects with type 2 diabetes mellitus;<br>• To evaluate the safety and tolerability of dorzagliatin with simultaneous administration of sitagliptin in subjects with type 2 diabetes mellitus.<br>**Secondary:**<br>• To assess the pharmacodynamic responses of glucose, GLP-1, glucagon, insulin, and C-peptide following dorzagliatin, sitagliptin, or simultaneous administration of dorzagliatin and sitagliptin in subjects with type 2 diabetes mellitus | |
| **Methodology:**<br>This is a Phase 1, open-label, sequential, multiple-dose, drug-drug interaction (DDI) study of glucokinase (GK) activator dorzagliatin and sitagliptin in subjects with type 2 diabetes mellitus (T2DM).<br>Study drugs will be administered in the following treatment scheme: | |

| Day | Sitagliptin | Dorzagliatin | PK Sample Collection |
|---|---|---|---|
| 1-5 | 100 mg (QD) for 5 days | - | Day 5:<br>Sitagliptin alone |
| 6-10 | 100 mg (QD) for 5 days | 75 mg (BID) for 4 days<br>Morning dose only on Day 10 | Day 10:<br>Sitagliptin + Dorzagliatin |
| 11-15 | - | 75 mg (BID) for 4 days<br>Morning dose only on Day 15 | Day 15:<br>Dorzagliatin alone |

Study drug will be taken 60 (±5) minutes prior to meals.

Eligible subjects will have a minimum 12-day run-in period prior to admission to the Clinical Research Center (CRC), during which time they will self-administer sitagliptin 100 mg QD each morning up until and including Day -2. Sitagliptin will be dispensed by the CRC. Subjects will complete a diary to record sitagliptin doses taken each day and will be advised to monitor their blood glucose levels and report changes in health status to the CRC. A telephone call will be placed to all subjects at approximately Day 6 (±2 day) of the run-in period to assess general health and collect adverse event (AE) information.

Following completion of the run-in period, eligible subjects will be admitted to the CRC on Day -2, have a total of 18 overnight stays, and be discharged after End-of-Study procedures are completed on Day 17, or at early termination.

Blood samples for the determination of dorzagliatin and sitagliptin concentrations and corresponding pharmacokinetic (PK) analysis will be collected at the following time points on Days 5, 10 and 15: pre-dose [redacted] and [redacted] post dose.

Subjects will consume a 75-gram glucose solution 30 minutes following study drug dose administration on Days 5, 10 and 15, in lieu of breakfast. Pharmacodynamic (PD) responses will be evaluated by measuring Glucagon-like Peptide-1 (GLP-1) and glucagon in plasma, and glucose, insulin, and C-peptide in serum within 60 minutes prior to oral glucose intake and 0.5, 1, 1.5, 2, 2.5, 3 and 4 hours after oral glucose intake.

Safety assessments will include monitoring of AEs, blood glucose via glucometer readings, vital signs (blood pressure, pulse rate, respiratory rate and oral temperature), clinical laboratory findings, resting 12-lead electrocardiograms (ECGs), and physical examination (PE) findings.

Vital signs will be assessed at screening, on Day -1, on Days 1-15 within 60 minutes prior to each morning and evening dose, and on Day 17. Blood glucose will be measured using a glucometer three times each day on Days -2 (prior to first meal after admission) through Day 16, within 60 minutes before each meal and after study drug dosing when applicable. Clinical laboratory evaluations (chemistry, hematology, urinalysis) will be performed at screening, on Days -1, 5, 10, 15 and 17. A resting 12-lead ECG will be completed at screening, on Day -1, and 2 hours post-dose on Days 5, 10, 15 and 17. Physical examinations will be conducted at screening, on Day -1, and on Day 17.

Subjects who terminate study participation early will have Day 17 assessments performed, if possible, prior to discharge from the CRC.

**Number of Subjects (planned):**

This study will plan to enroll up to 15 eligible subjects. Subjects may be enrolled singly or in groups. In order to have at least 12 evaluable subjects complete the study, additional subjects may be enrolled to replace subjects who discontinue study participation prematurely.

**Inclusion Criteria:**

1. Subjects diagnosed with T2DM at least 3 months prior to screening and are currently taking a stable dose of either of the following therapy with no change in the dose for at least 4 weeks prior to screening:
   a. ≥1000 mg per day of metformin
   b. a dipeptidyl peptidase 4 (DPP-4) inhibitor
   c. a sodium-glucose cotransporter-2 (SGLT2) inhibitor
   d. metformin plus a DPP-4 inhibitor
   e. metformin plus a SGLT2 inhibitor
2. Subjects must agree to change their current therapy to 100 mg sitagliptin QD for at least 14 days prior to dosing on Day 1;
3. In stable general health as determined by the Investigator at screening evaluation performed no more than 42 days prior to dosing on Day 1;
4. Male and/or female subjects between the ages of 30 and 65 years, inclusive;
5. Are capable of giving informed consent and complying with study procedures;
6. Body Mass Index (BMI) of 19 to 38 $kg/m^2$, inclusive, at screening;
7. Fasting C-peptide test result >0.3 nmol/L (>0.90 ng/mL);
8. HbA1c ≥7% and ≤10.5%;
9. Females must be using one of the following birth control methods: a non-hormonal intrauterine device (IUD) in place for at least 3 months; barrier method (condom, diaphragm) with spermicide for at least 14 days prior to dosing on Day 1 and throughout the study; surgical sterilization of the partner (vasectomy for 6 months minimum); OR are surgically sterile OR postmenopausal, as defined below:
   a. Surgically sterile for at least 3 months prior to screening by one of the following means:
      - Bilateral tubal ligation
      - Salpingectomy (with or without oophorectomy)
      - Surgical hysterectomy
      - Bilateral oophorectomy (with or without hysterectomy)
   b. Postmenopausal, defined as following:
      - Last menstrual period greater than 12 months prior to screening, confirmed by FSH and estradiol levels at screening;
10. Nonsmoker, defined as not having smoked or used any form of tobacco for more than 6 months prior to screening;
11. Willing and able to adhere to study restrictions and to be confined at the clinical research center.

**Exclusion Criteria:**

1. Fasting blood glucose at screening or Day -1 ≤110 or ≥270 mg/dL (one repeat screening fasting blood glucose allowed);
2. Type 1 diabetes mellitus, or latent autoimmune diabetes in adults; diabetic neuropathy, retinopathy or nephropathy;
3. Reported incidence of severe hypoglycemia, defined by denoting severe cognitive impairment requiring external assistance for recovery, within 3 months prior to screening and/or within 3 months prior to dosing on Day 1;
4. Known contraindications to sitagliptin;

5. Clinically significant gastrointestinal disorder (e.g., gastroparesis, constipation, diarrhea, colitis, gastrointestinal tract obstruction, hiatal hernia with reflux esophagitis, or cholestasis);
6. History or symptoms of clinically significant cardiovascular disease, particularly coronary artery disease, arrhythmias, atrial tachycardia, or congestive heart disease within one year prior to screening;
7. History of more than three urinary tract infections and/or more than three genital fungal infections in the last 12 months. If one or more genital fungal infections in the last 12 months required systemic treatment to resolve, Investigator will determine eligibility based on treatment required;
8. Reported history of clinically significant central nervous system disease including: transient ischemic attack, stroke, seizure disorder, depression, or behavioral disturbances within one year prior to screening;
9. Reported history of liver disease (e.g., hepatitis, cirrhosis within one year of screening and/or liver enzymes (alanine aminotransferase (ALT), aspartate aminotransferase (AST), gamma-glutamyltransferase (GGT), total bilirubin (TBiL)) >2 times the upper limit of normal (ULN));
10. Reported history of clinically significant renal disease;
11. Estimated glomerular filtration rate (eGFR) ≤60 mL/min/1.73m$^2$;
12. Acute or chronic metabolic acidosis, including diabetic ketoacidosis;
13. Known or suspected malignancy;
14. Any reported hypersensitivity (anaphylaxis and angioedema) or intolerance to sitagliptin;
15. Antidiabetic treatment with insulin, sulfonylureas, thiazolidinediones or GLP-1 agonist within 3 months prior to screening;
16. Systolic blood pressure <90 or ≥160 mmHg or diastolic blood pressure <60 or ≥100 mmHg at screening (may be repeated once);
17. A hospital admission or major surgery within 90 days prior to screening;
18. Uncontrolled hypertriglyceridemia >500 mg/dL;
19. Positive blood screen for human immunodeficiency virus (HIV), hepatitis B surface antigen (HBsAg), or hepatitis C antibody;
20. Positive pregnancy test result;
21. Female is breast-feeding or planning to become pregnant;
22. Treated with any investigational drugs within 6 weeks prior to screening;
23. Reported history of prescription drug abuse, or illicit drug use within 6 months prior to screening;
24. Reported history of alcohol abuse according to medical history within 6 months prior to screening;
25. A positive screen for alcohol or drugs of abuse;
26. Reported history of donation or blood collection of more than 1 unit (approximate 450 mL) of blood (or blood products) or acute loss of blood during the 90 days prior to screening;
27. Use of herbal (including St John's Wort, herbal teas, garlic extracts) within 14 days prior to dosing. No drugs known to be CYP3A4 inhibitors or inducers are allowed (See Appendix A for list of known CYP3A4 inhibitors or inducers). Use of other prescription and/or OTC medications within 14 days must be discussed and approved by the sponsor and the study PI prior to entry into the study.
28. An unwillingness of male participants to use appropriate contraceptive measures if engaging in sexual intercourse with a female partner of childbearing potential. Appropriate measures include use of a condom and spermicide and, for female partners, use of an IUD, diaphragm with

spermicide, oral contraceptives, injectable progesterone, progesterone subdermal implants, or a tubal ligation. Sexual intercourse with pregnant or lactating women is prohibited.

**Study Drugs, Dosage and Mode of Administration:**

Dorzagliatin will be provided as a film-coated tablets in 75 mg strength for oral administration 60 minutes prior to a meal.

Sitagliptin will be provided as [redacted] 100 mg tablets for oral administration 60 minutes prior to a meal.

**Duration of Treatment:**

The total duration of participation in the study for each subject is about 59 days (up to 28-day screening period, 12-day run-in period and 19-day in-clinic period).

**Criteria for Evaluation:**

**Safety:**

Safety assessments will include monitoring of AEs, blood glucose via glucometer readings, vital signs (blood pressure, pulse rate, respiratory rate and oral temperature), clinical laboratory findings, 12-lead ECGs, and PE findings.

**Pharmacokinetics:**

The plasma concentration-time data for dorzagliatin and sitagliptin will be analyzed using non-compartmental methods to calculate pharmacokinetic parameters. Actual dosing and sampling times will be used for analyses. The primary PK parameters of interest are: $C_{max}$, $T_{max}$ and $AUC_{0-24h}$. Additional parameters may be estimated and reported, as appropriate.

**Pharmacodynamics:**

Pharmacodynamic (PD) responses will be evaluated by measurement of GLP-1 and glucagon in plasma, and glucose, insulin and C-peptide in serum. The PD parameters may include, but not limited to, $AUEC_{0-4h}$, $CE_{max}$ and $CE_{av}$.

**Statistical Methods:**

Baseline demographic, concentration and safety data will be listed and summarized by descriptive statistics (n, mean, standard deviation, median, minimum, maximum) or frequency counts. Descriptive statistics will be calculated for dorzagliatin and sitagliptin concentrations in plasma.

Dorzagliatin and sitagliptin PK parameters ($C_{max}$, $T_{max}$ and $AUC_{0-24h}$) will be computed for each subject using non-compartmental methods based on the actual sampling times. Descriptive statistics will be calculated for the PK parameters for Days 5, 10 and 15.

The effect of sitagliptin on the exposure of dorzagliatin will be determined by a PK comparison between the exposure of dorzagliatin on Day 10 vs. Day 15. The effect of dorzagliatin on the exposure of sitagliptin will be determined by a PK comparison between the exposure of sitagliptin on Day 10 vs. Day 5.

The primary PK comparison will be based on the $C_{max}$ and $AUC_{0-24h}$ of dorzagliatin and sitagliptin. A mixed model under the sequential design will be used for analyzing $C_{max}$ and $AUC_{0-24h}$ values, based on natural log-transformed values, with treatments as fixed effects and subjects as random effects. The adjusted geometric mean ratios between two treatments for these parameters and the corresponding 90% confidence intervals (CI) for the ratio will be obtained by exponentiating the adjusted mean difference in logarithms and will be used for assessing PK differences between the two treatments within each PK comparison.

Baseline corrected PD markers will be calculated by subtracting the baseline value from the values post oral glucose intake. Plots of mean PD markers versus the scheduled time will be generated for

| |
|---|
| each treatment. Derivations and statistical analysis methods of PD parameters ($AUEC_{0-4h}$, $CE_{max}$ and $CE_{av}$) will be described in detail in Statistical Analysis Plan (SAP). |
| This study will be conducted in accordance with the guidelines of Good Clinical Practices (GCPs) including archiving of essential documents. |

# 2. LIST OF ABBREVIATIONS

The following abbreviations and specialist terms are used in this study protocol.

| Abbreviation | Term |
|---|---|
| ACE | Angiotensin-Converting Enzyme |
| AE | Adverse Event |
| ALT | Alanine Aminotransferase |
| ANOVA | Analysis of Variance |
| API | Active Pharmaceutical Ingredient |
| AST | Aspartate Aminotransferase |
| AUC | Area Under the Concentration-Time Curve |
| $AUC_{0-24h}$ | Area Under the Concentration-Time Curve from 0 to 24 Hours |
| $AUEC_{0-4h}$ | Area Under the Effect-Time Curve of Change from Baseline |
| BID | Twice Daily |
| BMI | Body Mass Index |
| BP | Blood Pressure |
| BUN | Blood Urea Nitrogen |
| $C_{max}$ | Observed Maximum Plasma Concentration |
| $CE_{av}$ | Average Change from Baseline |
| $CE_{max}$ | Maximum Change from Baseline |
| CFDA | China Food and Drug Administration |
| CFR | Code of Federal Regulations |
| CI | Confidence Interval |
| CL/F | Apparent Total Plasma Clearance of Drug after Oral Administration |
| CNS | Central Nervous System |
| CRC | Clinical Research Center |
| CRO | Contract Research Organization |
| CYP | Cytochrome P450 |
| DDI | Drug Drug Interaction |
| DMP | Data Management Plan |
| DPP-4 | Dipeptidyl Peptidase-4 |
| ECG | Electrocardiography |
| eCRF | Electronic Case Report Form |
| eGFR | Estimated Glomerular Filtration Rate |
| EOS | End-of-Study |
| FDA | Food and Drug Administration |

| Abbreviation | Term |
|---|---|
| FSH | Follicle Stimulating Hormone |
| GCP | Good Clinical Practice |
| GGT | Gamma-Glutamyl-Transferase |
| GK | Glucokinase |
| GKA | Glucokinase Activator |
| GLP | Good Laboratory Practice |
| GLP-1 | Glucagon-Like Peptide 1 |
| GMP | Good Manufacturing Practice |
| GSIR | Glucose Stimulated Insulin Release |
| HbA1c | Hemoglobin A1c |
| HBsAg | Hepatitis B Surface Antigen |
| Hct | Hematocrit |
| HCV | Hepatitis C Virus |
| HDL | High-Density Lipoprotein |
| hERG | Human Ether-a-go-go-Related Gene |
| Hgb | Hemoglobin |
| HIV | Human Immunodeficiency Virus |
| HLM | Human Liver Microsome(s) |
| IB | Investigational Brochure |
| $IC_{50}$ | The Half Inhibitory Concentration |
| ICF | Informed Consent Form |
| ICH | International Conference on Harmonisation |
| IEC | Independent Ethics Committee |
| IND | Investigational New Drug Application |
| IRB | Institutional Review Board |
| IUD | Intrauterine Device |
| $K_{el}$ | Elimination Rate Constant |
| LC-MS/MS | Liquid Chromatography-Tandem Mass Spectrometry |
| LDH | Lactate Dehydrogenase |
| LDL | Low-Density Lipoprotein |
| MAD | Multiple Ascending Dose |
| MATE | Multidrug and Toxin Extrusion |
| MedDRA | Medical Dictionary for Regulatory Activities |
| MTD | Maximum Tolerated Dose |
| NOAEL | No Observed Adverse Effect Level |
| OAT | Organic Anion Transporter |

| Abbreviation | Term |
|---|---|
| OATP | Organic Anion Transporting Polypeptide |
| OGTT | Oral Glucose Tolerance Test |
| OTC | Over-The-Counter |
| P-gp | P-Glycoprotein |
| PHI | Protected Health Information |
| PIS | Patient Information Sheet |
| PK | Pharmacokinetic(s) |
| QC | Quality Control |
| QD | Once Daily |
| QTc | Corrected QT Interval |
| RBC | Red Blood Cell |
| $S_{0.5}$ | Substrate Concentration to Give ½ $V_{max}$ |
| SAD | Single Ascending Dose |
| SAE | Serious Adverse Event |
| SAP | Statistical Analysis Plan |
| SDV | Source Data Verification |
| SGLT2 | Sodium-Glucose Cotransporter-2 |
| SOP | Standard Operating Procedure |
| $T_{1/2}$ | Terminal Elimination Half-Life |
| T2DM | Type 2 Diabetes Mellitus |
| TBiL | Total Bilirubin |
| TDI | Time Dependent Inhibition |
| TEAE | Treatment-Emergent Adverse Event |
| $T_{max}$ | Time at which $C_{max}$ was first observed |
| ULN | Upper Limit of Normal |
| $V_{max}$ | Maximum Metabolic Rate |
| $V_z/F$ | Apparent Volume of Distribution during Terminal Phase after Oral Administration |
| WBC | White Blood Count |
| WHO | World Health Organization |

# 3. INTRODUCTION

## 3.1 Background Information

Hua Medicine (Shanghai) Ltd. (hereinafter "sponsor" or "Hua Medicine") is developing dorzagliatin, an investigational novel glucokinase activator (GKA), indicated for use as an adjunct to diet and exercise to improve glycemic control in adults with type 2 diabetes mellitus (T2DM).

Glucokinase (GK) activators represent a promising new class of investigational drugs for the treatment of T2DM. Glucokinase activators lower blood glucose levels by enhancing the ability of pancreatic β-cells to "sense glucose" and increase insulin secretion in a glucose dependent manner. Simultaneously, GKAs can suppress glucose production and increase glucose utilization in the liver. Glucokinase activators may also function through other GK-expressing cells, such as entero-endocrine K and L-cells, and many GKAs have been shown to exert anti-apoptotic effects on β-cells.

Dorzagliatin (also referred to as HMS5552) is the 4$^{th}$ generation of GKAs. Dorzagliatin is an allosteric activator of GK which has been shown to increase the affinity of its substrate glucose by decreasing $S_{0.5}$ and increasing the $V_{max}$ of GK. Dorzagliatin has only a minor effect on the Hill coefficient nH and preserves the positive cooperativity of GK for glucose, a unique kinetic feature of GK. Dorzagliatin enhances glucose stimulated insulin release (GSIR) in rodent pancreatic islets and increases glucose uptake in cultured rodent primary hepatocytes. The selectivity profile of dorzagliatin was evaluated by screening 402 Ambit protein kinases and 78 CEREP receptors. In all cases the $IC_{50}$ values were [redacted], which was the highest concentration tested.[1]

Dorzagliatin is a potent glucose lowering agent showing excellent dose-related effects on fasting, basal and post-prandial glucose levels in several rodent models of T2DM, both in acute as well as chronic studies. Dorzagliatin augments GSIR and improves hepatic glucose disposal *in vivo*. Furthermore, treatment of normal mice with dorzagliatin has been associated with an increase in the levels of total Glucagon-like Peptide-1 (GLP-1). Therefore, dorzagliatin is a potential new antidiabetic agent for use as an adjunct to diet and exercise to improve glycemic control in adults with T2DM.

## 3.2 Preclinical Data

Preclinical evaluations including safety pharmacology, general toxicology studies in rats and dogs, reproductive toxicology, genetic toxicology were conducted following the guidance of International Conference on Harmonisation (ICH) and China Food and Drug Administration (CFDA), in compliance with the U.S. and CFDA Good Laboratory Practices (GLP) regulations. [1]

No QTc prolongation or dorzagliatin-related qualitative electrocardiographic events or abnormalities were observed at dose level up to [redacted] of dorzagliatin in safety pharmacology studies using telemetry technology in conscious dogs. [redacted] No adverse effect on central nervous system or respiratory system was observed in rats at [redacted] dorzagliatin (maximum dose).[1]

The repeat-dose toxicity and toxicokinetic profiles for orally administered dorzagliatin have been characterized in rats for 4 weeks, 13 weeks and 26 weeks and in dogs for 4 weeks, 13 weeks, and 39 weeks. [redacted] No adverse effect (including peripheral neuropathy) was found in dogs after dorzagliatin treatment [redacted]

No adverse effect on male reproductive organs and reproductivity was found in male fertility study [redacted] . [redacted]

[redacted] In the later phase of clinical trials, exclusive criteria have included the subjects who might potentially be pregnant.

No evidence for genotoxicity or mutagenicity of dorzagliatin was identified using in vitro Ames test and chromosomal aberration assay in human peripheral blood lymphocytes, and in vivo micronucleus test in rat bone marrow.[1]

These study results indicate that dorzagliatin has an adequate safety margin to support its development in the clinical setting.

## 3.3 Summary of Clinical Studies

To date, five clinical studies evaluating dorzagliatin have been completed, four of which were Phase 1 studies and one was a Phase 2 study. A total of 335 subjects have been exposed to dorzagliatin.[1]

Study HMM0101: In a Phase 1 Single Ascending Dose (SAD) study, a total of 48 healthy subjects received single oral doses of HMS5552 ranging from 5 mg to 50 mg. HMS5552 appeared to be safe and was well tolerated at all doses studied. No serious adverse events (SAEs) were reported and no withdrawal occurred due to an adverse event (AE). All AEs were mild in intensity and no treatment was required. [redacted] $C_{max}$ and AUC were apparently proportional to dose. No marked gender difference of $C_{max}$ and AUC was noticed, and the PK profile of HMS5552 indicated it was suitable for twice daily (BID) administration.

Study HMM0102: In a Phase 1 Multiple Ascending Dose (MAD) study, a total of 43 T2DM subjects received multiple twice-daily oral doses of HMS5552 ranging from 25 mg to 200 mg for 8 days. HMS5552 appeared to be safe and was well tolerated at all doses. There was no apparent sign or change in the pattern of any clinical laboratory value, vital signs or ECG parameters. All AEs were mild in intensity. No severe AE or SAE was reported. The most common AEs were related to mild hypoglycemia. No subjects reported serious hypoglycemia. All subjects recovered quickly without requiring additional intervention. The PK of HMS5552 appeared to be dose-proportional over the range tested without appreciable drug accumulation or food-effect. [redacted]

[redacted]

Study HMM0103: In a Phase 1c study, 24 T2DM subjects received daily doses of 75 mg or 150 mg (75 mg BID) HMS5552 daily for 4 weeks. HMS5552 displayed excellent safety and tolerability in subjects who participated in the study. There were no incidents of SAE, death, early termination due to AEs or severe hypoglycemia. All AEs were considered mild in intensity. No clinically significant changes were observed in laboratory, 12-lead electrocardiogram (ECG) or physical examination (PE) tests. Consistent PK Profiles were observed as in the MAD study. [redacted] renal excretion was not the major elimination pathway of HMS5552. [redacted] Functions of pancreatic β-cells were improved in both groups 3 days after the 28-day treatment ended, compared with baseline. The sensitivity of pancreatic β-cells to blood glucose was enhanced after 7-8 drug $T_{1/2}$ had passed.

Study HMM0104: In a Phase 1 drug-drug interaction (DDI) study conducted in the US, 15 T2DM subjects received HMS5552 50 mg BID or metformin 500 mg BID alone or in combination for a total of 13 days (metformin alone for 3 days, dorzagliatin and metformin co-administration for 5 days, then dorzagliatin alone for 5 days). It appeared treatment with HMS5552 50 mg BID alone or co-administration with metformin 500 mg BID was safe and well-tolerated. There were no incidents of SAE, death, or early termination due to AEs. All treatment-emergent adverse events (TEAEs) were mild in intensity and subjects recovered after intervention. There was no clear evidence to support the causal relationship between the study drug and TEAEs, and no incidence of hypoglycemic events. No clinically significant changes were observed in laboratory tests, vital signs, ECGs or PEs in this study. Combined treatment

with metformin did not affect the PK of HMS5552. [redacted] Overall, the study demonstrated that there is no apparent clinically significant DDI between HMS5552 and metformin. In contrast, the combined treatment resulted in improved glycemic control in T2DM subjects compared to treatment with HMS5552 or metformin alone.

Study HMM0201: A dose-ranging, randomized, double-blind, placebo-controlled, Phase 2 study was conducted in Chinese patients with T2DM, aiming to identify a minimum effective dose of dorzagliatin in patients with type 2 diabetes.[1,2]

Two hundred fifty-eight (258) T2DM patients enrolled in the Phase 2 study and were randomly assigned to receive placebo (n=53), 75 mg dorzagliatin once daily (n=53), 100 mg dorzagliatin once daily (n=50), 50 mg dorzagliatin twice daily (n=51), or 75 mg dorzagliatin twice daily (n=51) for 12 weeks.

No deaths, drug-related SAEs, or drug-related severe AEs were reported. Most AEs were mild and considered unrelated to study medication by investigators. The incidence of AEs was similar among groups. Adverse events that occurred in ≥5% of patients in any group (including placebo) were upper respiratory tract infection, hyperuricemia, dizziness, protein present in urine, urinary tract infection, blood creatine phosphokinase increased, white blood cells (WBC) urine positive, hepatic function abnormal, high-density lipoprotein (HDL) decreased, ventricular extrasystole and nasopharyngitis. Incidence of hypoglycemia was low, with a rate of 5.4% for ≤3.9 mmol/L, and a rate of only 1% for ≥3.0 mmol/L in the Phase 2 study in the drug-treated groups. No severe hypoglycemia was reported.

[redacted]

In conclusion, preclinical pharmacology studies demonstrated that HMS5552 is an effective allosteric activator of GK both *in vitro* and *in vivo*. Safety pharmacology, general toxicity studies

in rats and dogs up to 26 weeks and 39 weeks, reproductive toxicology, genetic toxicology and carcinogenicity studies suggested that HMS5552 has an acceptable safety profile when projected for human use. Furthermore, safety data from clinical phase 1 and phase 2 studies conducted in China and US support the continued development of HMS5552.

## 3.4 Drug Interaction Potential of Dorzagliatin

In *in vitro* studies using human liver microsomes (HLM) dorzagliatin showed no inhibition of liver cytochrome P450 (CYP) 1A2, CYP2A6, CYP2C9, CYP2C19, CYP2D6, CYP2E1 and CYP3A4/5 activities ($IC_{50}$ >50 µM) at concentrations studied.

Drug interaction potential of dorzagliatin via CYP induction was evaluated in primary human hepatocytes and results indicated that dorzagliatin was unlikely to produce induction on CYP1A2 or CYP2B6. While an absence of induction of dorzagliatin on CYP3A4 could not be excluded from this vitro study, it was later confirmed in preclinical and clinical PK studies as no decrease in exposure of dorzagliatin was observed after repeated dosing.

*In vitro* results suggest that dorzagliatin is a substrate of P-glycoprotein (P-gp) but not a P-gp inhibitor. In addition, dorzagliatin did not show clinically relevant inhibitory effect on organic anion transporting polypeptide (OATP)1B1, OATP1B3, organic anion transporter (OAT)1 and OAT3, nor is dorzagliatin a substrate of these four transporters. These study results demonstrate that transporter-related DDI is unlikely.

Since dorzagliatin is predominantly metabolized by CPY3A4 and is a substrate of P-gp, it is predicted that exposure of dorzagliatin will be increased when co-administered with CYP3A4 inhibitors and decreased when co-administered with CYP3A4 inducers.[1]

Dipeptidyl peptidase-4 (DPP-4) inhibitors are a class of oral drugs for the treatment of patients with T2DM who have not responded well to drugs such as metformin and sulfonylureas. DPP-4 inhibitors block the action of DPP-4, an enzyme that destroys incretins. Incretins are gastrointestinal hormones that help stimulate insulin production. FDA-approved DPP-4 inhibitors include sitagliptin [redacted], saxagliptin [redacted] and linagliptin [redacted]. The reported half-lives of sitagliptin, saxagliptin (and active metabolite) and linagliptin are 12.4, 2.5 (and 3.1), and 12 hours (effective half-life) respectively.[3,4,5]

Sitagliptin is mainly (79%) cleared as parent drug by urine with metabolism being a minor pathway of elimination. In vitro studies indicated that the primary enzyme responsible for the limited metabolism of sitagliptin was CYP3A4, with additional contribution from CYP2C8. Sitagliptin is a substrate for OAT 3, which may be involved in the renal elimination of sitagliptin.

Sitagliptin is not an inhibitor of CYP3A4, 2C8, 2C9, 2D6, 1A2, 2C19 or 2B6, and is not an inducer of CYP3A4. Sitagliptin is a P-gp substrate but does not inhibit P-gp mediated transport of digoxin. Based on these results sitagliptin is considered unlikely to cause interactions with other drugs that utilize these pathways. Sitagliptin is not extensively bound to plasma proteins. Therefore, the propensity of sitagliptin to be involved in clinically meaningful DDIs mediated by plasma protein binding displacement is very low.[3]

Collectively, the available PK and metabolic information for dorzagliatin and sitagliptin summarized above suggest that the DDI potential between these two drugs when co-administered is low.

In this DDI study, prior to enrollment subjects must be taking a stable dose of either metformin alone, metformin in combination with a marketed brand of a sodium-glucose cotransporter-2 (SGLT2) inhibitor or a DPP-4 inhibitor, or a marketed brand of an SGLT2 or DPP-4 inhibitor as monotherapy.

Metformin [redacted] is an oral antihyperglycemic drug that is commonly recommended as the first-line pharmacotherapy for treatment of T2DM. Mean time to peak plasma concentration ($T_{max}$) following administration of a metformin tablet is about 2-3 hours. Renal excretion is the major route of metformin elimination. Following oral administration, approximately 90% of the absorbed drug is eliminated via the renal route within the first 24 hours, with a plasma elimination half-life of approximately 6.2 hours.[6]

Sodium-glucose cotransporter-2 inhibitors are a class of oral drugs for the treatment of patients with T2DM that lower blood sugar by blocking SGLT2, a glucose transporter in the kidney, and preventing the kidney from reabsorbing glucose and releasing it into the blood. FDA-approved SGLT2 inhibitors include canagliflozin [redacted], dapagliflozin [redacted] and empagliflozin [redacted]. The reported half-lives of canagliflozin, dapagliflozin and empagliflozin are 10-13.1, 12.9 and 12.4 hours, respectively.[7,8,9]

# 4. STUDY RATIONALE AND OBJECTIVES

## 4.1. Study Rationale

Although the available drug interaction and PK data of dorzagliatin and sitagliptin detailed in Section 3.4 do not suggest a potential drug interaction, other drug transporter-mediated interactions cannot be ruled out. Furthermore, pharmacodynamic-based interaction potential needs to be evaluated. Based on the high likelihood that dorzagliatin may be co-administered with sitagliptin in T2DM patients in a clinical setting, this study will provide clinical evidence of the DDI potential between these two drugs.

The basis for the 100 mg QD dosing regimen of sitagliptin is based on the recommended 100 mg once daily dosing of [redacted] for adults.[3] The 75 mg BID for dorzagliatin is the dose regimen proven to be safe and effective in Phase 1 and Phase 2 studies and selected for two current Phase 3 studies evaluating dorzagliatin as a mono-therapy and as an add-on treatment to metformin. In the present study, both drugs will be given for five days to ensure that steady-state is attained.

The sequential, multiple-dose study is designed to determine whether the steady-state pharmacokinetics of dorzagliatin and sitagliptin are affected, while at the same time whether there's a synergistic therapeutic effect between these two drugs when co-administered. The proposed dosing regimens with dorzagliatin and sitagliptin will allow the potential interactions to be assessed under conditions that are expected to provide maximal exposure at the doses being studied. The elimination half-lives of dorzagliatin and sitagliptin are relatively short which justifies the 24-hour interval for collecting all samples to characterize the pharmacokinetics of both drugs.

## 4.2. Primary Objectives

The primary objectives of this study are:

- To assess the potential pharmacokinetic interaction between dorzagliatin and sitagliptin in subjects with type 2 diabetes mellitus;
- To evaluate the safety and tolerability of dorzagliatin with simultaneous administration of sitagliptin in subjects with type 2 diabetes mellitus.

## 4.3. Secondary Objective

The secondary objective of this study is:

- To assess the pharmacodynamic responses of glucose, GLP-1, glucagon, insulin, and C-peptide following dorzagliatin, sitagliptin, or simultaneous administration of dorzagliatin and sitagliptin in subjects with type 2 diabetes mellitus.

# 5. INVESTIGATIONAL PLAN

## 5.1. Overall Study Design

This is a Phase 1, open-label, sequential, multiple-dose, DDI study of GK activator dorzagliatin and sitagliptin in subjects with T2DM.

It is planned that 15 subjects will be enrolled to have at least 12 evaluable subjects. All subjects will receive:

- Sitagliptin 100 mg QD in the morning on Days 1-5;
- Sitagliptin 100 mg QD in the morning and dorzagliatin 75 mg BID (morning and evening) on Days 6-10, with only the morning dose on Day 10;
- Dorzagliatin 75 mg BID (morning and evening) on Days 11-15, with only the morning dose on Day 15.

Eligible subjects will have a minimum 12-day run-in period prior to admission to the CRC, during which time they will self-administer sitagliptin 100 mg QD up until and including Day -2. Sitagliptin will be dispensed by the CRC. Sitagliptin will be purchased and provided by the sponsor. Subjects will complete a diary to record sitagliptin doses taken each day and will be advised to monitor their blood glucose levels and report changes in health status to the CRC. A telephone call will be placed to all subjects at approximately Day 6 (±2 day) of the run-in period to assess general health and collect AE information.

Following completion of the run-in period, eligible subjects will be admitted to the CRC on Day -2, have a total of 18 overnight stays, and be discharged after End-of-Study procedures are completed on Day 17, or at early termination.

Blood samples for the determination of dorzagliatin and sitagliptin concentrations and corresponding PK analysis will be collected at the following time points on Days 5, 10 and 15: [redacted].

Subjects will consume a 75-gram glucose solution 30 minutes following study drug dose administration on Days 5, 10 and 15, in lieu of a breakfast. Pharmacodynamic responses will be evaluated by measuring GLP-1 and glucagon in plasma, and glucose, insulin, and C-peptide in serum at baseline (prior to the study drug dose and within 60 minutes prior to oral glucose intake) and 0.5, 1, 1.5, 2, 2.5, 3 and 4 hours after oral glucose intake.

Safety assessments will include monitoring of AEs, blood glucose via glucometer readings, vital signs (blood pressure (BP), pulse rate (PR), respiratory rate (RR) and oral temperature (T)), clinical laboratory findings, resting ECGs, and PE findings.

Vital signs will be assessed at screening, on Day -1, on Days 1-15 within 60 minutes prior to each morning and evening dose, and on Day 17. Blood glucose will be measured using a glucometer three times each day on Days -2 (prior to first meal after admission) through Day 16, within 60 minutes before each meal and after study drug dosing when applicable. Clinical laboratory evaluations (chemistry, hematology, urinalysis) will be performed at screening, on Days -1, 5, 10, 15 and 17. A resting 12-lead ECG will be completed at screening, on Day -1, and 2 hours post-dose on Days 5, 10, 15, and 17. Physical exams will be conducted at screening, on Day -1, and on Day 17.

Subjects who receive their Day 1 dose and then terminate study participation early will have Day 17 assessments performed, if possible, prior to discharge from the CRC.

Morning dosing will occur at approximately 08:00 a.m. and evening dosing will occur at approximately 06:00 p.m. All doses will be administered 60 (±5) minutes prior to a standardized meal and with approximately 240 mL (8 fluid ounces) of room temperature water. On Days 5, 10 and 15 there will be no meal offered after the morning dose and instead the oral glucose solution will be administered 30 minutes after the study drug dose. The actual time of each dose, each post-dose meal, and time of oral glucose solution administration will be recorded.

See Table 5-1 for the details of all study procedures for subjects in the study.

**Table 5-1 Schedule of Procedures**

| Visit | Screening | Run-in* | In-patient at the CRC* | | | | | | | | | |
|---|---|---|---|---|---|---|---|---|---|---|---|---|
| **Day** | **-42 to -15** | **-14 to -3** | **-2** | **-1** | **1-4** | **5** | **6-9** | **10** | **11-14** | **15** | **16** | **17 EOS** |
| Informed consent | X | | | | | | | | | | | |
| Dispense/collect sitagliptin, at-home sitagliptin QD 100 mg. Tel call at Day -8±2 (Day 6 of the 12-day run-in period)* | | X | X | | | | | | | | | |
| Admission to CRC | | | X | | | | | | | | | |
| Eligibility assessment | X | | | X | | | | | | | | |
| Demographics | X | | | | | | | | | | | |
| Medical history | X | | X | | | | | | | | | |
| Physical examination | X | | | X | | | | | | | | X |
| Height (cm), Weight (kg) | X | | | X[1] | | | | | | | | X[1] |
| Body mass index | X | | | | | | | | | | | |
| Standard 12-lead ECG[2] | X | | | X | | X | | X | | X | | X |
| Vital signs (BP, PR, RR, T) | X | | | X | Within 60 min prior to each study drug dose. | | | | | | | X |
| Clinical laboratory samples[3] | X | | | X | | X | | X | | X | | X |
| HIV, hepatitis B & C | X | | | | | | | | | | | |
| Urine drug /saliva alcohol | X | | X | | | | | | | | | |
| Urine pregnancy test[4] | X | | X | | | | | | | | | X |
| FSH and estradiol[4] | X | | | | | | | | | | | |
| OGTT/PD markers [5] | | | | | | X | | X | | X | | |
| C-peptide and HbA1c | X | | | | | | | | | | | |
| Dose administration[6] | | | | Sitagliptin 100 mg QD on Day -1; Assigned treatment Days 1-15 | | | | | | | | |
| AE & con med reporting[7] | | X | X | X | X | X | X | X | X | X | X | X |
| PK blood samples[8] | | | | | | X | X | X | X | X | X | |
| Glucometer (finger stick) | | | Within 60 minutes before each meal and after study drug dosing when applicable | | | | | | | | | |
| Discharge from CRC | | | | | | | | | | | | X |

AE= adverse event; BP = blood pressure; con med = concomitant medications; CRC = clinical research center; ECG = electrocardiogram; EOS =End-of-Study; FSH: Follicle stimulating hormone; HIV = human immunodeficiency virus; PR = pulse rate; PK = pharmacokinetics; RR = respiratory rate; T = temperature.

*Run-in period is a minimum of 12 days. If longer than 12 days, admission to CRC remains on Day -2. Sitagliptin will be dispensed by CRC to eligible subjects any day prior to start of Day -14 at-home dosing.

[1]Obtain weight only.

[2]ECG obtained in supine position after at least 5 minutes rest. On Days 5, 10 and 15 ECGs obtained 2 hours (±15 minutes) after dose administration.

[3]Clinical laboratory samples include: hematology, chemistry and urinalysis. All samples collected prior to study drug dose on days of study drug administration.

[4]For female subjects, urine pregnancy test must be negative to enroll in the study. Serum FSH and estradiol will be evaluated for postmenopausal females to confirm status.

[5] Oral glucose solution administered 30 (±5) minutes after study drug dose on Days 5, 10 and 15. PD samples to measure GLP-1 and glucagon in plasma, and glucose, insulin, and C-peptide in serum collected at baseline (prior to the study drug dose and within 60 minutes prior to oral glucose intake) and at 0.5, 1, 1.5, 2, 2.5, 3 and 4 hours after oral glucose intake. All post-glucose collection times are ±5 minutes from nominal time.

[6]All subjects will receive: sitagliptin 100 mg QD on Days 1-5 (a.m. dose); sitagliptin 100 mg QD and dorzagliatin 75 mg BID on Days 6-10 (a.m. dose only on Day 10); and dorzagliatin 75 mg BID on Days 11-15 (a.m. dose only on Day 15). Morning dosing will occur at approximately 08:00 a.m. and evening dosing will occur at approximately 06:00 p.m. All doses are 60 (±5) minutes prior to meals except for Days 5, 10 and 15 morning doses when no meals are offered following the study drug dose. Subjects are to resume their regular medication on Day 16.

[7]SAE collection starts after signing ICF.

[8]PK samples collected [redacted] on Days 5, 10 and 15. Blood collection time windows: ±5 minutes from 0-4 hours post-dose and ±10 minutes ≥6 hours post-dose.

## 5.2. Number of Subjects

This study will plan to enroll up to 15 eligible subjects. Subjects may be enrolled singly or in groups. In order to have at least 12 evaluable subjects complete the study, additional subjects may be enrolled to replace subjects who discontinue study participation prematurely. Evaluable subjects are defined as subjects included in the DDI population as described in Section 12.2.

## 5.3. Treatment Assignment

All subjects will receive the same treatment assignment as follows:

**Table 5-2: Treatment Assignment and PK Sample Collection**

| Day | Sitagliptin | Dorzagliatin | PK Sample Collection |
|---|---|---|---|
| 1-5 | 100 mg (QD) for 5 days | - | Day 5:<br>Sitagliptin alone |
| 6-10 | 100 mg (QD) for 5 days | 75 mg (BID) for 4 days<br>Morning dose only on Day 10 | Day 10:<br>Sitagliptin + Dorzagliatin |
| 11-15 | - | 75 mg (BID) for 4 days<br>Morning dose only on Day 15 | Day 15:<br>Dorzagliatin alone |

## 5.4. Criteria for Study Termination

The Investigator has the right to terminate the study in the interest of subject safety and welfare in consultation with sponsor. Additionally, the sponsor reserves the right to terminate or amend the study at any time for administrative reasons or if continuation of the protocol would present a potential safety risk to the subjects.

# 6. SELECTION AND WITHDRAWAL OF SUBJECTS

## 6.1. Subject Inclusion Criteria

To participate in the study, subjects must meet all the following eligibility criteria:

1. Subjects diagnosed with T2DM at least 3 months prior to screening and are currently taking a stable dose of either of the following therapy with no change in the dose for at least 4 weeks prior to screening:
   a. ≥1000 mg per day of metformin

   b. a dipeptidyl peptidase 4 (DPP-4) inhibitor
   c. a sodium-glucose cotransporter-2 (SGLT2) inhibitor
   d. metformin plus a DPP-4 inhibitor
   e. metformin plus a SGLT2 inhibitor
2. Subjects must agree to change their current therapy to 100 mg sitagliptin QD for at least 14 days prior to dosing on Day 1;
3. In stable general health as determined by the Investigator at screening evaluation performed no more than 42 days prior to dosing on Day 1;
4. Male and/or female subjects between the ages of 30 and 65 years, inclusive;
5. Are capable of giving informed consent and complying with study procedures;
6. Body Mass Index (BMI) of 19 to 38 kg/m$^2$, inclusive, at screening;
7. Fasting C-peptide test result >0.3 nmol/L (>0.90 ng/mL);
8. HbA1c ≥7% and ≤10.5%;
9. Females must be using one of the following birth control methods: a non-hormonal intrauterine device (IUD) in place for at least 3 months; barrier method (condom, diaphragm) with spermicide for at least 14 days prior to dosing on Day 1 and throughout the study; surgical sterilization of the partner (vasectomy for 6 months minimum); OR are surgically sterile OR postmenopausal, as defined below:
   a. Surgically sterile for at least 3 months prior to screening by one of the following means:
      - Bilateral tubal ligation
      - Salpingectomy (with or without oophorectomy)
      - Surgical hysterectomy
      - Bilateral oophorectomy (with or without hysterectomy)
   b. Postmenopausal, defined as following:
      - Last menstrual period greater than 12 months prior to screening, confirmed by FSH and estradiol levels at screening;
10. Nonsmoker, defined as not having smoked or used any form of tobacco for more than 6 months prior to screening;
11. Willing and able to adhere to study restrictions and to be confined at the clinical research center.

## 6.2. Subject Exclusion Criteria

Subjects will be excluded from study entry if any of the following exclusion criteria are present:

1. Fasting blood glucose at screening or Day -1 ≤110 or ≥270 mg/dL (one repeat screening fasting blood glucose allowed);
2. Type 1 diabetes mellitus, or latent autoimmune diabetes in adults; diabetic neuropathy, retinopathy or nephropathy;
3. Reported incidence of severe hypoglycemia, defined by denoting severe cognitive impairment requiring external assistance for recovery, within 3 months prior to screening and/or within 3 months prior to dosing on Day 1;
4. Known contraindications to sitagliptin;
5. Clinically significant gastrointestinal disorder (e.g., gastroparesis, constipation, diarrhea, colitis, gastrointestinal tract obstruction, hiatal hernia with reflux esophagitis, or cholestasis);
6. History or symptoms of clinically significant cardiovascular disease, particularly coronary artery disease, arrhythmias, atrial tachycardia, or congestive heart disease within one year prior to screening;
7. History of more than three urinary tract infections and/or more than three genital fungal infections in the last 12 months. If one or more genital fungal infections in the last 12 months required systemic treatment to resolve, Investigator will determine eligibility based on treatment required;
8. Reported history of clinically significant central nervous system disease including: transient ischemic attack, stroke, seizure disorder, depression, or behavioral disturbances within one year prior to screening;
9. Reported history of liver disease (e.g., hepatitis, cirrhosis within one year of screening and/or liver enzymes (alanine aminotransferase (ALT), aspartate aminotransferase (AST), gamma-glutamyltransferase (GGT), total bilirubin (TBiL)) >2 times the upper limit of normal (ULN));
10. Reported history of clinically significant renal disease;
11. Estimated glomerular filtration rate (eGFR) ≤60 mL/min/1.73m$^2$;
12. Acute or chronic metabolic acidosis, including diabetic ketoacidosis;
13. Known or suspected malignancy;

14. Any reported hypersensitivity (anaphylaxis and angioedema) or intolerance to sitagliptin;
15. Antidiabetic treatment with insulin, sulfonylureas, thiazolidinediones or GLP-1 agonist within 3 months prior to screening;
16. Systolic blood pressure <90 or ≥160 mmHg or diastolic blood pressure <60 or ≥100 mmHg at screening (may be repeated once);
17. A hospital admission or major surgery within 90 days prior to screening;
18. Uncontrolled hypertriglyceridemia >500 mg/dL;
19. Positive blood screen for human immunodeficiency virus (HIV), hepatitis B surface antigen (HBsAg), or hepatitis C antibody;
20. Positive pregnancy test result;
21. Female is breast-feeding or planning to become pregnant;
22. Treated with any investigational drugs within 6 weeks prior to screening;
23. Reported history of prescription drug abuse, or illicit drug use within 6 months prior to screening;
24. Reported history of alcohol abuse according to medical history within 6 months prior to screening;
25. A positive screen for alcohol or drugs of abuse;
26. Reported history of donation or blood collection of more than 1 unit (approximate 450 mL) of blood (or blood products) or acute loss of blood during the 90 days prior to screening;
27. Use of herbal (including St John's Wort, herbal teas, garlic extracts) within 14 days prior to dosing. No drugs known to be CYP3A4 inhibitors or inducers are allowed (See Appendix A for list of known CYP3A4 inhibitors or inducers). Use of other prescription and/or OTC medications within 14 days must be discussed and approved by the sponsor and the study PI prior to entry into the study.
28. An unwillingness of male participants to use appropriate contraceptive measures if engaging in sexual intercourse with a female partner of childbearing potential. Appropriate measures include use of a condom and spermicide and, for female partners, use of an IUD, diaphragm with spermicide, oral contraceptives, injectable progesterone, progesterone subdermal implants, or a tubal ligation. Sexual intercourse with pregnant or lactating women is prohibited.

## 6.3. Prohibitions and Restrictions

Subjects must be willing to adhere to the following prohibitions and restrictions from times noted and for the duration of the study:

- No illicit drug use, abuse of alcohol and use of tobacco-containing products within 6 months prior to screening;
- No consumption of alcohol or food containing alcohol;
- No consumption of food or drinks containing caffeine;
- No consumption of grapefruit juices;
- Subjects must remain upright for 4 hours following administration of study drug (except during measurement of vital signs and ECGs) following the morning dose on Days 5, 10 and 15.

## 6.4. Subject Withdraw Criteria

Subjects will be informed that they have the right to withdraw from the study at any time for any reason without prejudice to their medical care. The Investigator also has the right to withdraw subjects from the study if it is in the best interest of the subject, or if the subject experiences an adverse event that warrants premature withdrawal.

Subjects may be withdrawn from the study if they:

- Have entered the study in violation of the protocol
- Use or require the use of prohibited concomitant medication(s)
- Are non-compliant with study procedures
- Experience an AE that warrants premature withdrawal

Subjects who experience emesis after dosing are not required to withdraw from the study. The event and time of emesis should be documented, and the subject permitted to continue at the discretion of the Investigator.

All treated subjects should be followed according to the Schedule of Procedures (Table 5-1). All subjects who receive a Day 1 dose, even those who have discontinued prematurely, should have all evaluations for the Day 17 End-of-Study visit performed, if possible. All procedures should

be documented in the electronic case report form (eCRF). For all subjects who withdraw prematurely, the Investigator will indicate one of the following reasons for withdrawal in the eCRF:

- Adverse event
- Death
- Protocol violation
- Lost to follow-up
- Subject's decision
- Other (reason to be specified by the Investigator)

In case of subjects' discontinuation from the study due to an AE, such subjects will be closely monitored until the resolution or stabilization of the AE. The Investigator should document the reason for discontinuation in the source documentation and eCRF.

In the event that a subject withdraws participation from the study early, early withdrawal should be documented by the Investigator (or designee) in the appropriate eCRF pages and source documents when confirmed. The Day 17 End-of-Study assessments should be performed when a subject is discontinued.

# 7. STUDY PROCEDURES

Morning dosing will occur at approximately 08:00 a.m. and the evening dosing will occur at approximately 06:00 p.m. All doses will be administered 60 (±5) minutes prior to meals (except for on Days 5, 10 and 15 when meals are not offered after the morning dose) with about 240 mL (8 fluid ounces) of room temperature water. Actual times for each dose and start times for meals as well as oral glucose intake on days of dosing will be recorded. Standardized meals that are consistent with general dietary recommendations for diabetes will be provided while subjects are staying at CRC.

Eligible subjects will have a minimum 12-day run-in period prior to admission to the CRC on Day -2, during which time they will self-administer sitagliptin 100 mg QD each morning. Subjects will complete a diary to record sitagliptin doses taken each day and will be advised to monitor their blood glucose levels and report changes in health status to the CRC. A telephone

call will be placed to all subjects at approximately Day 6 (±2 day) of the run-in period to assess general health and collect AE information. Subjects will be admitted to the CRC on Day -2 and remain sequestered at the study site until after all End-of-Study procedures are completed on Day 17 or at early termination.

The following sections describe in detail all study procedures. The schedule of procedures is presented in Table 5-1.

## 7.1. Screening Visit and Run-in Period

Subjects will report to the CRC for a screening outpatient visit between Day -42 and Day -15 relative to the Day 1 dosing day.

As outlined in Section 15.3, prior to the performance of any study-related activities or evaluations, the subject must be thoroughly informed about all aspects of the study, including scheduled visits and activities. Each subject will sign the study-specific consent form prior to any screening procedures. A signed copy of the informed consent form (ICF) will be given to each consenting subject and another signed copy will be retained in the subject's study records.

The following information and procedures will be performed and documented as part of the screening assessment:

- Collection of demographic information, including sex, race, ethnic origin, date of birth.
- Medical history, including review of prior and ongoing medications taken in previous 30 days, except for therapy to treat diabetes, which must be recorded for previous 3 months.
- Height and weight measurement and BMI calculation.
- Urine sample collection for urinalysis and test for drugs of abuse.
- Urine sample pregnancy test for women.
- Blood test for evaluation of FSH and estradiol for postmenopausal women.
- Saliva sample collection to test use of alcohol.
- Standard 12-lead ECG after resting supine for at least 5 minutes.
- Vital signs (BP, PR, RR and oral T) measurement after resting supine for at least 5 minutes.
- Blood sample collection for clinical chemistry, hematology, HIV, and hepatitis B and C evaluations, C-peptide measurement and HbA1c test.

- Physical examination.
- Assessment of eligibility according to inclusion/exclusion criteria.

Compliance with inclusion criteria (listed in Section 6.1) and exclusion criteria (listed in Section 6.2) will be verified against information collected and documented in the source documents and the eCRF. Laboratory results obtained at screening and on Day -1 will be used to verify eligibility.

Eligible subjects will be scheduled for a visit to the CRC for dispensing of sitagliptin 100 mg and start their 12-day run-in period. Subjects will be instructed to take a single dose of sitagliptin each morning, and to record each dose in a diary that will be provided by the CRC. Subjects will be advised to monitor their blood glucose levels and report changes in health status to the CRC. A telephone call will be placed to all subjects at approximately Day 6 (±2 day) of the run-in period to assess general health and collect AE information. Any signs or symptoms reported by subjects that develop or worsen compared to the subject's medical history after the first dose of sitagliptin in the run-in period will be recorded as AEs and will be followed by the Investigator. Additionally, subjects will be advised to contact their primary care physician.

An appointment will be scheduled for subjects to be admitted to the CRC on Day -2. Subjects will be advised to take their dose of sitagliptin at home prior to admission, and to bring remaining drug and their diary with them to the CRC on Day -2.

## 7.2. Domiciled at CRC on Day -2 through Day 17

### 7.2.1. Study Check-in Visit: Day -2 and Day -1

Subjects will be admitted to the CRC on Day -2 and remain for 18 overnight stays until completion of all study procedures on Day 17 or early termination.

The following assessments will be performed on Day -2:

- Collection of remaining sitagliptin and review of diary to confirm compliance with daily dosing of sitagliptin during the run-in period. Subjects with <90% compliance or compliance recorded as >110% are not eligible to continue study participation.
- Update of medical history, assessment of AEs, and medication use since the screening visit.
- Urine sample testing for drugs of abuse and pregnancy, as applicable.

- Alcohol saliva test.
- Assess blood glucose using a glucometer within 60 minutes before each meal and after study drug dosing when applicable.

The following assessments will be performed on Day -1:

- Blood and urine sample collection for clinical chemistry, hematology and urinalysis in the morning under fasting condition (after at least a 10-hour fast).
- Physical examination.
- Vital signs (BP, PR, RR and oral T) measurement after resting supine for at least 5 minutes.
- Measure weight
- Standard 12-lead ECG after resting supine for at least 5 minutes.
- Subjects will be administered sitagliptin 100 mg by the clinic staff in the morning at approximately 08:00 a.m. prior to a standardized meal.
- Assess blood glucose using a glucometer within 60 minutes before each meal and after study drug dosing when applicable.
- Confirm fasting blood glucose on Day -1 >110 and <270 mg/dL.
- Confirmation of eligibility according to inclusion/exclusion criteria.
- Assessment of AEs and concomitant medications.

### 7.2.2. Day 1 - Day 4

- Vital signs (BP, PR, RR and oral T) measurement after resting supine for at least 5 minutes within 60 minutes before each study drug dose.
- Administer 100 mg sitagliptin QD at 08:00 a.m. (±5 minutes), 60 (±5) minutes prior to a standardized meal.
- Assess blood glucose using a glucometer within 60 minutes before each meal and after study drug dosing when applicable.
- Assessment of AEs and concomitant medications.

### 7.2.3. Day 5

- Blood and urine sample collection for clinical chemistry, hematology and urinalysis in the morning under fasting condition (after at least a 10-hour fast) and before study drug dose.
- Vital signs (BP, PR, RR and oral T) measurement after resting supine for at least 5 minutes within 60 minutes before study drug dose.
- Administer 100 mg sitagliptin at 08:00 a.m. (±5 minutes). Subjects must remain upright for at least 4 hours following dosing except for measurement of vital signs or ECGs.
- Pharmacokinetic blood sample collections for the measurement of sitagliptin concentrations [redacted].
- Consume a 75-gram glucose solution 30 (±5) minutes following study drug dose in lieu of a breakfast.
- Pharmacodynamic blood sample collections to measure GLP-1 and glucagon in plasma, and glucose, insulin, and C-peptide in serum at baseline (prior to study drug dose and within 60 minutes prior to oral glucose intake) and 0.5, 1, 1.5, 2, 2.5, 3 and 4 hours after oral glucose intake.
- Assess blood glucose using a glucometer within 60 minutes before each meal and after study drug dosing when applicable.
- Standard 12-lead ECG after resting supine for at least 5 minutes, taken 2 hours (±15 minutes) after study drug dose.
- Assessment of AEs and concomitant medications.

### 7.2.4. Day 6 - Day 9

- Pharmacokinetic blood sample collections for the measurement of sitagliptin concentrations 18 and 24 hours post-dose (Day 6).
- Vital signs (BP, PR, RR and oral T) measurement after resting supine for at least 5 minutes within 60 minutes before each study drug dose.
- Administer 100 mg sitagliptin QD at 08:00 a.m. (±5 minutes) and 75 mg dorzagliatin BID (08:00 a.m., 06:00 p.m. (±5 minutes), each dose 60 (±5) minutes prior to a standardized meal.

- Assess blood glucose using a glucometer within 60 minutes before each meal and after study drug dosing when applicable.
- Assessment of AEs and concomitant medications.

### 7.2.5. Day 10

- Blood and urine sample collection for clinical chemistry, hematology and urinalysis in the morning under fasting condition (after at least a 10-hour fast) and before study drug dose.
- Vital signs (BP, PR, RR and oral T) measurement after resting supine for at least 5 minutes within 60 minutes before each study drug dose.
- Administer 100 mg sitagliptin and 75 mg dorzagliatin at 08:00 a.m. (±5 minutes). Subjects must remain upright for at least 4 hours following dosing except for measurement of vital signs or ECGs.
- Pharmacokinetic blood sample collections for the measurement of sitagliptin and dorzagliatin concentrations [redacted].
- Consume a 75-gram glucose solution 30 (±5) minutes following study drug dose in lieu of a breakfast.
- Pharmacodynamic blood sample collections to measure GLP-1 and glucagon in plasma, and glucose, insulin, and C-peptide in serum at baseline (prior to study drug dose and within 60 minutes prior to oral glucose intake) and 0.5, 1, 1.5, 2, 2.5, 3 and 4 hours after oral glucose intake.
- Assess blood glucose using a glucometer within 60 minutes before each meal and after study drug dosing when applicable.
- Standard 12-lead ECG after resting supine for at least 5 minutes, taken 2 hours (±15 minutes) after study drug dose.
- Assessment of AEs and concomitant medications.

### 7.2.6. Day 11 - Day 14

- Pharmacokinetic blood sample collections for the measurement of sitagliptin and dorzagliatin concentrations 18 and 24 hours post-dose (Day 11).

- Vital signs (BP, PR, RR and oral T) measurement after resting supine for at least 5 minutes within 60 minutes before each study drug dose.
- Administer 75 mg dorzagliatin BID (08:00 a.m., 06:00 p.m. (±5 minutes) 60 (±5) minutes prior to a standardized meal.
- Assess blood glucose using a glucometer within 60 minutes before each meal and after study drug dosing when applicable.
- Assessment of AEs and concomitant medications.

### 7.2.7. Day 15

- Blood and urine sample collection for clinical chemistry, hematology and urinalysis in the morning under fasting condition (after at least a 10-hour fast) and before each study drug dose.
- Vital signs (BP, PR, RR and oral T) measurement after resting supine for at least 5 minutes within 60 minutes before each study drug dose.
- Administer 75 mg dorzagliatin at 08:00 a.m. (±5 minutes). Subjects must remain upright for at least 4 hours following dosing except for measurement of vital signs or ECGs.
- Pharmacokinetic blood sample collections for the measurement of dorzagliatin concentrations [redacted].
- Consume a 75-gram glucose solution 30 (±5) minutes following study drug dose in lieu of a breakfast.
- Pharmacodynamic blood sample collections to measure GLP-1 and glucagon in plasma, and glucose, insulin, and C-peptide in serum at baseline (prior to study drug dose and within 60 minutes prior to oral glucose intake) and 0.5, 1, 1.5, 2, 2.5, 3 and 4 hours after oral glucose intake.
- Assess blood glucose using a glucometer within 60 minutes before each meal and after study drug dosing when applicable.
- Standard 12-lead ECG after resting supine for at least 5 minutes, taken 2 hours (±15 minutes) after study drug dose.
- Assessment of AEs and concomitant medications.

#### 7.2.8. Day 16

- Pharmacokinetic blood sample collections for the measurement of dorzagliatin concentrations 18 and 24 hours post-dose.
- Assess blood glucose using a glucometer within 60 minutes before each meal.
- Subjects will resume their regular medication schedule.
- Assessment of AEs and concomitant medications.

#### 7.2.9. Day17: End-of-Study Procedures

The following procedures will be performed at the End-of-Study or Termination Visit:

- Blood and urine sample collection for clinical chemistry, hematology and urinalysis in the morning under fasting condition (after at least a 10-hour fast).
- Urine pregnancy test for women.
- Physical examination.
- Measure weight.
- Vital signs (BP, PR, RR and oral T) measurement after resting supine for at least 5 minutes.
- Standard 12-lead ECG after resting supine for at least 5 minutes.
- Assessment of AEs and concomitant medications.
- Subjects will be offered breakfast and may be discharged from the CRC upon completion of all study procedures.

### 7.3. Early Withdrawal Visit

As outlined in Section 6.4, in the event that a subject discontinues study participation after Day 1 dose administration but prior to the final End-of-Study visit, if possible, the subject should complete all End-of Study assessments prior to being discharged from the CRC, as described in Section 7.2.9 for Day 17. The reason for discontinuation must be fully documented in the subject's source documentation and in the eCRF.

### 7.4. Time Windows for Procedures

Any procedure performed outside the stated time windows below will be recorded as a protocol deviation.

- Sitagliptin and dorzagliatin administered by clinic staff will be taken 60 (±5) minutes prior to meals.
- Standard resting 12-lead ECGs will be performed 2 hours (±15 minutes) after the study drug dose, after the subject has rested in the supine position for at least 5 minutes.
- The oral glucose solution will be administered 30 (±5) minutes after study drug dose on Days 5, 10 and 15.
- Blood samples for PK analyses will be collected ±5 minutes from 0-4 hours post-dose and ±10 minutes ≥6 hours post-dose.
- Blood samples for PD analyses will be collected ±5 minutes from 0-4 hours after glucose intake.
- Meal start times will be within ±10 minutes of scheduled start time.

# 8. TREATMENT OF SUBJECTS

## 8.1. Description of Study Drugs

Dorzagliatin (HMS5552) is an investigational novel GKA, indicated as an adjunct to diet and exercise to improve glycemic control in adults with T2DM.

The anticipated dosing regimen is twice daily as oral administration. The proposed dosage form for this study is a film-coated tablet with 75 mg dorzagliatin. [redacted]

[redacted]

Sitagliptin ([redacted] 100 mg tablets for oral administration) will be supplied by Hua Medicine.

## 8.2. Methods of Assigning Subjects to Dose Groups

There is no randomization since this is an open label, single sequence study. Subjects may be enrolled singly or in groups.

## 8.3. Blinding

This is an open label study. There will be no blinding for this study.

## 8.4. Treatment Discontinuation/Subject Withdrawal

Subjects may withdraw from the study at any time at their own request, or they may be withdrawn at any time at the discretion of the Investigator or sponsor for safety, behavioral, or administrative reasons. If a subject discontinues study participation at any time prior to the final End-of-Study assessments, if possible, the subject should complete all End-of Study assessments. The reason for discontinuation must be fully documented in the subject’s source documentation and in the eCRF.

## 8.5. Prior and Concomitant Medications and Substances

All prescription medications and OTC products, including herbal products, taken within 30 days prior to screening and during the study period will be documented in the subject’s source documentation and the eCRF. Therapy to treat T2DM taken within 3 months prior to screening will also be documented in the subject’s source documentation and the eCRF. The documented use of prescription sitagliptin during the run-in period is required.

## 8.6. Meals

Standardized meals based on the recommended American Diabetes Association diet, will be offered to all subjects. An evening snack (no later than 09:00 p.m.) will be offered on the evening of admission. Subjects will be domiciled and dosed, and have meals provided by the CRC. Standard meals will be served at approximately 60 minutes after dosing and shall be completed within 30 minutes when meals are administered after a study drug dose. While domiciled at the CRC, meals will be offered at approximately 09:00 a.m., 12:30 p.m. and 07:00 p.m., and a light evening snack will be offered at approximately 9:00 p.m. or earlier. Meal start times will be within ±10 minutes when the meal time coincides with a scheduled PK or PD blood draw. Soft drinks (sodas) without caffeine or sugar, or non-grapefruit juices without added sugar will be offered with meals and ad libitum beginning 4 hours post-dose.

Water intake will be restricted from 1 hour prior to dosing until after the 1-hour post-dose PK blood sample is collected (with the exception of 240 mL of water given with the dosing) on study Days 5, 10 and 15.

# 9. STUDY DRUG MATERIALS AND MANAGEMENT

## 9.1. Study Drug Packaging and Labeling

Dorzagliatin drug products are supplied as 75 mg strength tablets. The drug product tablets are packaged in Alu-Pla Blister as in total 8 tablets per plate.

Product will be labeled according to applicable regulatory requirements. The label will include at least the following information:

- Name and address of manufacturer
- Protocol number or other identifier to reference the study
- Name of the sponsor, product name and batch number
- Place to record subject number and initials
- Storage conditions

The CRC pharmacist or designee will prepare the study drug and maintain the drug packaging and labeling log.

Sitagliptin ([redacted] 100 mg tablets for oral administration) will be supplied by Hua Medicine.

## 9.2. Study Drug Storage and Accountability

All study drug supplies will be stored in a secure, locked area, under the responsibility of the Investigator or other authorized individual.

Dorzagliatin must be stored under controlled room temperature (10-30°C or 50-86°F) in tightly closed containers protected from light. Sitagliptin must be stored at 20-25°C (68-77°F) with excursions permitted to 15-30°C (59-86°F). The temperature and humidity of the stored room should be monitored and recorded.

The Investigator or designee must maintain accurate records of the receipt of all study drug, including date received, lot number, expiration date if applicable, amount received, condition of the package and the disposition of all study drug.

Current dispensing records will also be maintained including the date and amount of study drug dispensed and the identity of the subject receiving the study drug.

## 9.3. Study Drug Preparation

Sitagliptin 100 mg tablets will be dispensed to subjects for at-home dosing during the 12-day run-in period. Study drug reconciliation will be performed at the end of the run-in period by tablet count.

Dorzagliatin 75 mg and sitagliptin 100 mg tablets will be dispensed to subjects while domiciled at the CRC according the study treatment scheme. Morning and evening doses of study drug will be administered by CRC staff with approximately 240 mL (8 fluid ounces) room temperature water at approximately 08:00 a.m. and 06:00 p.m., 60 (±5) minutes prior to a meal, except for Days 5, 10 and 15 when there will be no meal following the morning dose.

## 9.4. Study Drug Handling and Disposal

All unused study drug and supplies must be returned to sponsor or disposed according to sponsor's instruction after the study is completed and the drug accountability log is reconciled.

# 10. PHARMACOKINETIC/PHARMACODYNAMIC ASSESSMENTS

## 10.1. Pharmacokinetic Sample Collection and Storage

A total of 42 blood samples (approximately 6 mL each) will be collected from each subject into $K_2EDTA$ tubes for determination of dorzagliatin and sitagliptin concentration and PK assessments. Samples will be collected [redacted] on Days 5, 10 and 15 using an indwelling catheter or direct venipuncture.

When PK blood collection times coincide with other procedures, the blood sample should be collected first, using the following post-dose windows: ±5 minutes from 0-4 hours post-dose and ±10 minutes $\geq$6 hours post-dose.

Contents of tube will be mixed thoroughly with gentle inversion at least 8 times to mix the anti-coagulant and stored on ice for no more than 30 minutes before centrifugation at approximately 4 (±1)°C at ~2000 g for 10 minutes. The harvested plasma will be split into two approximately equal aliquots and stored in 2 mL or appropriate size cryovial tubes.

Plasma samples will be stored at -70 (±10)°C within approximately 60 minutes of harvesting pending shipment to the bioanalytical laboratory for analysis. Full instructions for the collection, storage, and shipment of these samples will be provided in a separate PK Laboratory Manual.

## 10.2. Pharmacokinetic Specimen Labeling

Labels will be affixed to the cryovials in a manner that will prevent the label from being detached after being wet or freezing. The tube labels will contain the subject number, treatment, nominal day, and nominal collection time, as appropriate.

The site will provide a sample inventory page, listing the information above for each sample. The sample inventory page will also include the treatment cohort for each series of tubes.

## 10.3. Pharmacokinetic Sample Shipping Instructions

All PK plasma samples will be kept frozen and shipped on dry ice by the same day or overnight courier to:

Frontage Laboratories, Inc.
Sample Coordinator
[redacted]

Plasma samples will be shipped in two separate shipments. The first shipment of samples (the primary aliquot of each sample) will be shipped after subjects have completed the study. The second aliquot of each sample will be shipped after notification from the laboratory of receipt of primary samples.

## 10.4. Bioanalytical Methodology

The PK plasma samples will be analyzed using validated, specific and sensitive methods of liquid chromatographic separation with tandem mass spectrometric (LC-MS/MS) detection for concentrations of dorzagliatin and sitagliptin by the designated bioanalytical lab.

## 10.5. Pharmacokinetic Parameters

Dorzagliatin and sitagliptin PK parameters ($C_{max}$, $T_{max}$ and $AUC_{0-24h}$) will be computed for each subject using non-compartmental methods by Phoenix WinNonlin version 8.1 (Certara,

Princeton, NJ USA) based on the actual sampling times. Additional PK parameters ($T_{1/2}$, $K_{el}$, $V_z/F$, CL/F) may be calculated if needed. Descriptive statistics will be calculated for the PK parameters for Days 5, 10 and 15. A description of PK terms is provided below.

**Table 10-1: PK Terms**

| PK Term | Description |
|---|---|
| $C_{max}$ | Observed maximum plasma concentration |
| $T_{max}$ | Time at which $C_{max}$ was first observed |
| $AUC_{0-24h}$ | Area under the concentration-time curve from 0 to 24 hours |
| $T_{1/2}$ | Terminal elimination half-life |
| $K_{el}$ | Elimination rate constant |
| $V_z/F$ | Apparent volume of distribution during terminal phase after oral administration |
| CL/F | Apparent total plasma clearance of drug after oral administration |

## 10.6. Pharmacodynamic Assessments

Subjects will consume a 75-gram glucose solution 30 (±5) minutes following study drug administration on Days 5, 10 and 15, in lieu of a breakfast. The glucose solution will be consumed within a 5-minute timeframe. A total of 48 blood samples (24 samples of approximately 8.5 mL each for measurement of plasma GLP-1 and glucagon, and 24 samples of approximately 7 mL each for measurement of serum glucose, insulin, and C-peptide will be collected from each subject for PD assessments. Samples will be collected at baseline (prior to the study drug dose and within 60 minutes prior to oral glucose intake) and 0.5, 1, 1.5, 2, 2.5, 3 and 4 hours after oral glucose intake on Days 5, 10 and 15.

When PD blood collection times coincide with other procedures, the blood sample should be collected first within the ±5 minutes time window.

For measurement of plasma GLP-1 and glucagon, approximately 8.5 mL blood will be collected into Becton Dickenson BD P800 tubes that contain spray-dried $K_2EDTA$ anticoagulant and other proprietary additives. Contents will be gently inverted 8-10 times and centrifuged within 60 minutes of collection at 1100-1300 g for 20 minutes at 18-25°C. Three aliquots of plasma will be harvested as 1.0 mL for measurement of glucagon and active GLP-1, 1.0 mL for measurement of total GLP-1 and 1.5 mL as a back-up sample. All samples will be stored immediately at ≤70°C until shipment to the Bioanalytical laboratory. Samples will be shipped on dry ice to:

[redacted]
Mercodia AB
[redacted]

For measurement of serum glucose, insulin and C-Peptide, the sample collection tubes and processing instructions will be provided by BioReference Laboratories, the facility that will measure all safety laboratory assessments.

Full instructions for the collection, storage, and shipment of PD samples will be provided in a separate PD Laboratory Manual.

# 11. ASSESSMENT OF SAFETY

Safety assessments will include monitoring of AEs, blood glucose via glucometer readings, vital signs, clinical laboratory findings, resting 12-lead ECGs and PE findings.

## 11.1. Safety Parameters

### 11.1.1. Demographic/Medical History

Demographic characteristics (age, sex, race and ethnicity) will be collected at the screening visit. Medical history will be reviewed and collected at the screening and on Day -2.

### 11.1.2. Vital Signs

Vital signs, including BP, PR, RR, and oral T, will be measured at screening, on Day -1, on Days 1-15 within 60 minutes prior to each morning and evening dose, and on Day 17 or at the end of the study. Vital signs will be measured after resting supine for at least 5 minutes.

Vital signs may be repeated for clinically significant abnormal vital signs at the discretion of the Investigator.

As a guideline, vital signs outside the ranges shown below may be considered abnormal, at the discretion of the Investigator:

SBP: 95-160 mmHg

DBP: 55-100 mmHg

PR: 45-100 bpm

RR: 10-20 bpm

T: 36.0-37.2°C

### 11.1.3. Physical Examination, Height, Weight, and Body Mass Index

Full PEs will be performed by qualified personnel at the screening visit, Day -1, Day 17 and the End-of-Study visit. At the screening visit, height (centimeters) and weight (kilograms) will be measured and BMI will be calculated. Weight will also be measured on Day -1 and Day 17. All abnormal findings will be documented in the source documentation and in the eCRF.

### 11.1.4. Electrocardiogram

Standard resting 12-lead ECGs will be performed at the screening visit, Day -1, 2 hours (±15 minutes) post-dose on Days 5, 10 and 15, and on Day 17 at the End-of-Study visit. All ECGs will be performed after the subject has rested supine for at least 5 minutes.

Post-dose ECGs must be evaluated for safety by the Investigator or his/her designee. The final conduction intervals entered into the eCRF will be those generated by the ECG machine, unless deemed significantly inaccurate by the review performed by the Investigator or designee. In these cases, the over-read intervals will be documented in the eCRF. The Investigator will also record an overall assessment of the ECG.

Clinically significant abnormal ECGs may be repeated at the discretion of the Investigator.

As a guideline, ECG parameters outside the ranges shown below may be considered abnormal, at the discretion of the Investigator:

VR: 45-100 bpm

PR: 120-210 msec

QRS: ≤120 msec

QT: <500 msec

QTc: <450 msec

### 11.1.5. Clinical Laboratory Assessments

Hematology, blood chemistry, and urinalysis evaluations will be performed at the screening visit, on Day -1, Days 5, 10, and 15, and Day 17 (End-of-Study Visit). Blood and urine samples will be collected after an overnight fast of at least 10 hours, and within 30 minutes prior to the

morning dose of study drug on Days 5, 10 and 15. The list of clinical laboratory assessments is included in Appendix B.

C-peptide and HbA1c testing will be performed at the screening visit to determine eligibility.

Blood glucose will be measured using a glucometer three times each day on Days -2 through Day 16, within 60 minutes before each meal and after study drug dosing when applicable.

The results of clinical laboratory tests conducted at the screening visit and on Day -1 must be assessed by the Investigator to determine each subject's eligibility for participation in the study. The Investigator should signify review of the laboratory reports by signing and dating the report. If subjects' values are out of range and the Investigator deems the out of range value is clinically significant, the Investigator must discuss and agree upon individual cases with sponsor's Medical Monitor prior to enrollment.

Any significant abnormalities should be fully investigated. Whenever possible, the etiology of the abnormal findings will be documented on the eCRF. Laboratory results with clinically significant abnormal values may be repeated for verification. Additional tests and other evaluations required to establish the significance or etiology of an abnormal result or to monitor the course of an AE should be obtained when clinically indicated. In particular, if a clinically significant abnormal result is observed that is not resolved by the final study visit, repeat tests will be performed if possible and resolution or stability of the abnormality will be recorded in the source documentation.

Any clinically significant laboratory abnormalities that are either serious (e.g., results in hospital admission) or unexpected will be promptly reported to the representative of sponsor. Any additional relevant laboratory results obtained by the Investigator during the course of this study will be reported to sponsor.

Virus serology (HIV, and hepatitis B and C) will be assessed at the screening visit and must be negative to qualify enrollment.

Urine drug screens and saliva alcohol tests will be conducted at the screening visit and on Day -2. Results must be negative to qualify for dosing on Day 1.

Urine pregnancy test for all female subjects will be performed at the screening visit, on Day -2 and on Day 17 (End-of-Study). Results at screening and Day -2 must be negative to qualify for

dosing on Day 1. Any positive pregnancy test results on Day 17 will be reported to the sponsor within 24 hours of awareness of the pregnancy using a Pregnancy Report provided by Frontage.

Postmenopausal status of females will be confirmed by serum FSH and estradiol levels at screening.

## 11.2. Adverse Events

Adverse events will be recorded after the first dose of sitagliptin in the run-in period. Subjects will be instructed to report AEs during the study and staff will query subjects regarding AEs throughout the study. The Investigator or designee must document all AEs reported by the subject after the first dose of sitagliptin in the run-in period through completion of the End-of-Study visit. Any subject who is withdrawn from the study due to an AE shall be followed until the event has resolved or stabilized, and the Investigator will document available follow-up information on the subject's source documentation and eCRF.

### 11.2.1. Definitions of Adverse Events

Adverse event means any untoward medical occurrence in a patient or clinical investigation subject administered a pharmaceutical product and which does not necessarily have to have a causal relationship with this treatment. An AE can therefore be any unfavorable and unintended sign, symptom, or disease temporally associated with the use of medicinal product, whether or not related to the medicinal product. An AE is any symptom, sign, illness, or experience that develops or worsens in severity and/or frequency during the course of the study. Adverse events reported after administration of the first dose of study drugs (Day 1) will be considered treatment-emergent. Adverse events will be considered treatment-emergent if not present prior to the initiation of the treatment with study drug on Day 1 or already present but worsens in either severity or frequency following exposure to the treatment.

Adverse events include serious and non-serious AEs. A Serious Adverse Event (SAE) is defined as an AE occurring during any clinical study period that meets one or more following criteria:

- Results in death
- Is life threatening
- Requires inpatient hospitalization or prolongation of existing hospitalization
- Results in persistent or significant disability/incapacity

- Is a congenital anomaly/birth defect
- Is a medically important event that may jeopardize the subject or may require medical or surgical intervention to prevent one of the other outcomes listed above

The term "life threatening" in the definition of "serious" refers to an event in which the subject was at risk of death at the time of the event; it does not refer to an event that hypothetically might have caused death if it were more severe. Death is an outcome of an event. The event that resulted in death should be recorded in source documents, the eCRF and reported on the SAE form. All SAEs that occur after consent to the End-of-Study visit must be reported to sponsor using the SAE Reporting Form provided by Frontage (see Section 11.2.3).

Abnormal results of laboratory tests or diagnostic procedures (such as test results from hematology, blood biochemistry, urinalysis, ECG, physical examination, vital signs evaluations, etc.) are considered to be AEs if the abnormality is considered by the Investigator as clinically significant, or the clinical significance worsens compared to the subject's baseline.

Any of the following abnormal results are considered clinically significant:

- The abnormality suggests a disease and/or organ toxicity that is new or has worsened from baseline;
- The abnormality needs to adjust investigational product dosage and usage, e.g., drug discontinuation;
- The abnormality requires additional active intervention, for instance an increase or a modification of the concomitant medication, close observation, or further diagnostic investigation, etc.

If a clinically significant laboratory abnormality is a manifestation of disease, then only the diagnosis will be recorded as AE. If a clinically significant laboratory abnormality is not a manifestation of disease, then the abnormality itself is recorded as AE. An appropriate description is used to record test results that are either lower or higher than normal range. If the result meets diagnostic criteria, the clinical diagnosis is recorded as AE.

The severity of each AE will be graded by the Investigator according to the following criteria:

- Mild: awareness of sign or symptom, but easily tolerated.
- Moderate: discomfort sufficient to cause interference with normal activities.

- Severe: incapacitating, with inability to perform normal activities.

The Investigator must assess the relationship between the AE and the study drug by using the following definitions:

1. **Related**: The occurrence of AE follows a reasonable temporal sequence from administration of the study drug; adverse event is consistent with known characteristics of the study drug; the event is improved when the dose of study drug is decreased or stopped; the event re-occurs when the study drug treatment is re-started. It cannot be explained by the medical condition of the subject or alternative treatment.
2. **Possibly Related**: The occurrence of AE follows a reasonable temporal sequence from administration of the study drug; AE is consistent with a known characteristic of the study drug; reducing the dosage or stop the drug will cause the AE to be alleviated or no obvious change. The medical condition of the subject or alternative treatment may have contributed to the event.
3. **Unlikely Related**: The occurrence of AE, whose temporal sequence from administration of the study drug, is unclear. The AE may not be consistent with a known characteristic of the study drug. The medical condition of the subject or alternative treatments may have contributed to the event.
4. **Not Related**: There is no reasonable temporal relationship between the AE occurrence and study drug administration; the AE is not consistent with known characteristic of the study drug; the medical condition of the subject or alternative treatments may have contributed to the event. The AE improves or disappears when disease condition improves, or alternative treatment is stopped.

The outcome of an AE can be described as:

- Recovered: the subject returns to baseline state.
- Recovering: the events haven’t been resolved completely, but subject is improving.
- Not recovered: the events are ongoing, for example, irreversible congenital anomaly
- Recovered with sequelae: only if the subject will suffer from life-time sequelae, for example, the blindness caused by diabetes and the hemiplegia after a stroke.
- Fatal: the death date is the event end date.

- Unknown: Investigator can't obtain the outcomes of AEs, e.g., the subject is lost to follow-up.

If the AE outcome is assessed as "Recovered", "Recovered with sequelae", or "Fatal", the end date of the AE must be recorded.

### 11.2.2. Recording Adverse Events

All AEs (regardless of seriousness or relationship to study drug) are to be recorded in the subject's source documents and on the corresponding page(s) in the eCRF. Whenever possible, symptoms, signs, and laboratory abnormalities should be grouped as a single syndrome or diagnosis. The Investigator should specify the date of onset and stop date of event, severity and the time of severity worsening, action taken with respect to study drug, corrective treatment/therapy given, seriousness, outcome, hospitalization date (if applicable), discharge date (if applicable), cause of the death (if applicable), date of death (if applicable), autopsy (if applicable) and his/her opinion as to whether there is a reasonable possibility that the AE was caused by the study drug, according to the definitions noted in Section 11.2.1. All medications administered to treat an AE must be recorded in the subject's source documentation and documented in the eCRF.

### 11.2.3. Reporting of Serious Adverse Events

Reporting of SAEs will be conducted in accordance with the appropriate regulatory guidelines. All SAEs that occur from the time of consent to the End-of-Study visit must be reported, whether or not the event is considered associated with the study drug. The Investigator must complete the SAE Reporting Form provided by Frontage and submit it by fax or email with other relevant source documentation to sponsor within 24 hours of awareness of the event to:

[redacted]

The Investigator must also provide with urgent priority (upon receipt of a request) other relevant documentation (e.g., copies of diagnostic test results, hospital discharge summary, and/or autopsy report) and send this information by fax or email to sponsor.

All SAEs must be recorded in the subject's source documentation and documented in the eCRF. Medications administered in association with the SAE must be documented in the eCRF and in the subject's source documentation. The Investigator must also promptly notify the Institutional Review Board (IRB) /Independent Ethics Committee (IEC) of SAEs, including any follow-up information, in accordance with local institutional policy and applicable regulatory requirements.

Regulatory authorities will be notified of any AE associated with the use of the study drug that is both serious and unexpected by sponsor, in accordance with the appropriate local regulatory guidelines. Notification of the event will be made by a written, expedited safety report.

#### 11.2.4. Adverse Event Follow-up

The Investigator should take all appropriate measures to ensure the safety of the subjects, including referral to a specialist if indicated. The Investigator should follow up on the outcome of any AE until the event has resolved or stabilized.

This implies that follow-up may continue after the subject discontinues from the study and that additional information may be requested. Any SAE follow-up information that occurs after database lock should be reported to sponsor.

#### 11.2.5. Hy's Law

The study will utilize Hy's Law to monitor any potential drug-induced liver injury. Hy's Law usually means that AST or ALT > 3 x ULN and TBiL > 2 x ULN, non-biliary increase (usually alkaline phosphatase < 2 x ULN) without any other diseases can explain this increase. If ALT or AST > 3 x ULN, and TBiL > 2 x ULN without any other past diseases can explain this increase, no matter ALP increases or not, re-evaluation need to be performed. Investigator will conduct subject re-evaluation, and closely follow-up of the subjects or stop the study drug due to liver function abnormality, according to Section 6.4 Subject withdrawal. The Investigator will evaluate the etiology and perform every essential examination to rule out drug-induced liver injury. If Investigator confirms the occurrence of Hy's Law, an SAE must be reported.

#### 11.2.6. Hypoglycemia

Hypoglycemia events shall be recorded on an eCRF page. The following information will be recorded for each occurrence of hypoglycemia:

- Start/end date and time
- Blood glucose values
- Symptom descriptions
- Action taken
- Severe hypoglycemia or not
- Resolved or not
- Predisposing factors
- Causal relationship with the study drug

Hypoglycemia should be treated using standard medical practice at the Investigator's discretion. Severe hypoglycemia events will be reported as an SAE.

# 12. STATISTICAL METHODOLOGY

## 12.1. Sample Size Determination

Sample size calculations based on study design and intra-subject variability were performed by the sponsor. At least 10 evaluable subjects in the sequence will be required to achieve a power of at least 0.8 for the geometric mean ratios between two treatments (sitagliptin + dorzagliatin vs. dorzagliatin alone or sitagliptin + dorzagliatin vs. Sitagliptin alone) for $C_{max}$ or $AUC_{0\text{-}24h}$, with the equivalence bounds of 0.8 and 1.25, assuming a true geometric mean ratio of 1 and an intra-subject variability (coefficient of variation) of 16.1%, in an equivalence test using two one-sided test at a significance level of 0.05. The intra-subject variability for sitagliptin $C_{max}$ and AUC are reported to be 16.1% and 5.7%, respectively.[10] The intra-subject variability for dorzagliatin $C_{max}$ and $AUC_{0\text{-}24h}$ are estimated to be 14.0% and 6.2%, respectively.

Therefore, to ensure a satisfactory DDI assessment, and assuming a drop-out rate of 20%, we plan to enroll 15 eligible subjects by aiming to obtain 12 evaluable subjects for DDI assessment.

## 12.2. Analysis Population

The Safety Population will be defined as all subjects who receive study drug.

The PK Population will be defined as all subjects who receive study drug, have no major protocol violations, and have sufficient PK data to obtain reliable estimates of the key PK variables.

The PD Population will be defined as all subjects who receive study drug, have no major protocol violations, and have sufficient PD data to obtain reliable estimates of the key PD parameters.

The DDI Population will consist of the PK Population subjects who complete all treatments as defined by the protocol.

## 12.3. Pharmacokinetics and Pharmacodynamics

Pharmacokinetic parameters described in Section 10 will be summarized by descriptive statistics (n, mean, standard deviation, median, minimum, maximum) or frequency counts. Descriptive statistics will be calculated for dorzagliatin and sitagliptin concentrations in plasma.

Dorzagliatin and sitagliptin PK parameters ($C_{max}$, $T_{max}$ and $AUC_{0-24h}$) will be computed for each subject using non-compartmental methods and based on the actual sampling times. Descriptive statistics will be calculated for the PK parameters on Days 5, 10 and 15.

The effect of sitagliptin on the exposure of dorzagliatin will be determined by a PK comparison between the exposure of dorzagliatin on Day 10 vs. Day 15. The effect of dorzagliatin on the exposure of sitagliptin will be determined by a PK comparison between the exposure of sitagliptin on Day 10 vs. Day 5.

The primary PK comparison will be based on the $C_{max}$ and $AUC_{0-24h}$ of dorzagliatin and sitagliptin. A mixed model under the sequential design will be used for analyzing $C_{max}$ and $AUC_{0-24h}$ values, based on natural log-transformed values, with treatments as fixed effects and subjects within sequence as random effects. The adjusted geometric mean ratios between two treatments for these parameters and the corresponding 90% confidence intervals (CI) for the ratio will be obtained by exponentiating the adjusted mean difference in logarithms and will be used for assessing PK differences between the two treatments within each PK comparison.

A Statistical Analysis Plan (SAP) will be developed and signed prior to database lock, and will describe in more detail how PK parameters will be derived.

Pharmacodynamic measurements (glucose, GLP-1, glucagon, insulin, and C-peptide) will be listed by subject for all subjects with actual sampling time. Summary statistics of glucose, GLP-1, glucagon, insulin, and C-peptide will be provided by scheduled (nominal) time point and treatment, respectively. Baseline corrected PD markers will be calculated by subtracting the

baseline value from the values post oral glucose intake. The baseline for PD correction will be defined in the SAP. Plots of mean PD markers versus the scheduled time will be generated for each treatment. Derivations and statistical analysis methods of PD parameter ($AUEC_{0-4h}$, $CE_{max}$ and $CE_{av}$) will be described in detail in the SAP.

## 12.4. Demographic Characteristics

Demographic characteristics will be summarized for the subjects enrolled in the study using descriptive statistics.

An attempt will be made to enroll similar numbers of men and women in the study.

## 12.5. Exposure to Study Drugs

Each subject's exposure to study drug will be summarized using descriptive statistics, i.e., the number of subjects exposed to each treatment.

## 12.6. Concomitant Medications

Concomitant medications will be coded using the World Health Organization (WHO) Enhanced dictionary.

## 12.7. Safety Analyses

Safety evaluations will be based on the incidence, severity, and relatedness of AEs and changes in subjects' PE findings, ECGs, vital signs, and clinical laboratory results.

Safety variables will be tabulated and presented for all subjects in the Safety Population. Data will be summarized by treatment. Adverse events will be coded using the Medical Dictionary for Regulatory Activities (MedDRA) for preferred term and system organ class, listed by subject. TEAEs will be summarized by treatment. All AEs will be summarized by relationship to study drug and by severity.

Deaths, SAEs, and AEs resulting in study discontinuation will be tabulated and detailed in narratives.

Change from baseline, defined as time of admission to the CRC or screening, whichever value is the last value prior to first dose, in clinical laboratory parameters, 12-lead ECGs, and vital sign parameters will be summarized by treatment.

Additional safety analyses may be defined in the SAP.

### 12.8. Interim Analyses

No interim analyses are planned for this study.

## 13. DIRECT ACCESS TO SOURCE DATA/DOCUMENTS

### 13.1. Study Monitoring

At regular intervals during the study, the site will be contacted through monitoring visits, letters, and telephone calls by a representative to review study progress, Investigator and subject compliance with study protocol requirements, and any emergent problems. During monitoring visits, the following points will be reviewed in accordance with all applicable regulatory requirements and Standard Operating Procedures (SOPs): original medical records and other source documents, the Investigator site file, screening logs, ICF, subject recruitment and follow-up, SAE documentation and reporting, documentation and reporting of endpoints, study drug allocation, subject compliance with the study drug regimen, study drug accountability, concomitant therapy use, and quality of data. In addition, other required regulatory documents will be reviewed, including but not limited to: IRB/IEC composition and correspondence, laboratory certification(s), delegation of authority, and Investigator and study personnel curricula vitae.

### 13.2. Sponsor's Responsibility

The sponsor or its designee is responsible for the following:

- Selecting qualified Investigators;
- Providing Investigators with the information they need to properly conduct an investigation;
- Ensuring proper monitoring of the investigation;
- Ensuring that the applicable regulatory authorities, and all participating Investigators are properly informed of significant new information regarding AEs or risks associated with the medication being studied.

As the sponsor, Hua Medicine has delegated some responsibilities to Frontage Clinical Services, a Contract Research Organization (CRO).

### 13.3. Audits and Inspections

The sponsor's Quality Assurance Unit (or representative) may conduct audits at the study site(s). Audits will include, but are not limited to: drug supply, presence of required documents, the informed consent process, laboratory specimen processing, and comparison of eCRFs with source documents. The Investigator agrees to cooperate with audits conducted at a reasonable time and in a reasonable manner.

Regulatory authorities worldwide may also inspect the Investigator site during or after the study. The Investigator should contact the sponsor immediately if this occurs and must fully cooperate with the Inspector.

The Investigator is required to make all study documentation promptly available for inspection, review or audit at the study site upon request by sponsor, its representatives, or any appropriate regulatory agencies.

## 14. QUALITY CONTROL AND QUALITY ASSURANCE

Each investigational site will perform internal quality management of study conduct, data and biological specimen collection, documentation and completion. An individualized quality management plan may be developed to describe a site's quality management.

Quality control (QC) procedures will be implemented beginning with the data entry system and data QC checks that will be run on the database will be generated. Any missing data or data anomalies will be communicated to the site(s) for clarification/resolution.

Following written SOPs, the monitors will verify that the clinical trial is conducted, and data are generated, and biological specimens are collected, documented (recorded), and reported in compliance with the protocol, ICH GCP, and applicable regulatory requirements (e.g., GLP, Good Manufacturing Practices (GMP)).

The investigational sites will provide direct access to all trial related sites, source data/documents, and reports for the purpose of monitoring and auditing by the sponsor, and inspection by local and regulatory authorities.

# 15. ETHICS

The procedures set out in this study protocol, pertaining to the conduct, evaluation, and documentation of this study, are designed to ensure that the sponsor and the Investigator abide by GCP, including but not limited to Title 21 Code of Federal Regulations (CFR) Parts 50, 56, and 312 and the ICH guidelines and directives. Compliance with these regulations also constitutes compliance with the ethical principles described in the current revision of the Declaration of Helsinki, Fortaleza 2013 and applicable local regulatory requirements and law.

Copies of these materials are available from Hua Medicine and Frontage Clinical Services (the CRO) designee by request. The purpose of these regulations, legal obligations and guidance is to define the standards and principles for the proper conduct of clinical studies that have been developed by the medical, scientific, and regulatory communities. They are not intended to impede or restrict clinical research.

The ethical standards defined within GCP are intended to ensure that:

1. Human subjects are provided with an adequate understanding of the possible risks of their participation in the study, and that they have a free choice to participate or not;
2. The study is conducted with diligence and in conformance with the protocol in such a way as to protect subject safety and ensure the integrity of the findings;
3. The potential benefits of the research justify the risks.

The Investigator is responsible for protecting the rights, safety, and welfare of subjects under his/her care, and for the control of the medications under investigation.

The Principal Investigator will ensure that all persons assisting with the study are adequately informed about the protocol, any amendments to the protocol, the study drug, and their study related duties and functions. The Investigator will maintain a list of sub-Investigators and other appropriately qualified persons to whom he or she has delegated significant study-related duties. Individuals ineligible to conduct or work on clinical studies, including those ineligible as a result of debarment under the Generic Drug Enforcement Act of 1992, will not be allowed to conduct or work on studies sponsored by Hua Medicine. The Investigator is required to immediately disclose to sponsor in writing, if any person involved in the conduct of the study is debarred

pursuant to a hearing by US FDA under this anti-fraud law, or if any proceeding for debarment is pending, or is (to the best of the Investigator's knowledge) threatened.

The rest of this section of the protocol describes in more detail the specific GCP-defined responsibilities the Investigator assumes by agreeing to participate in this study.

## 15.1. Ethics Review

The Investigator (or designee) must submit this study protocol, the sponsor approved ICF, information intended for distribution to subjects, subject recruitment materials, and other appropriated documents to the appropriate IRB or IEC. Following IRB or IEC review, a copy of the signed, written and dated approval will be provided to the sponsor, along with a list of the IRB/IEC composition.

The approval/favorable opinion should clearly state the study (study number, protocol title, and version number), the documents reviewed (Protocol, ICF, IB, etc.) and the date of the review. The study will not commence at the study site until sponsor has received a copy of this written and dated approval/favorable opinion.

During the study, any amendment to the protocol and the ICF (as appropriate) shall be submitted to the IRB/IEC. The IRB/IEC should also be informed of any event likely to affect the safety of subjects or the continued conduct of the study, in particular any change in safety. Additionally, all updates to the IB will be sent to the IRB/IEC. A progress report will be sent to the IRB/IEC and the protocol will be reviewed annually (e.g., re-approved) or more frequently, as required by IRB/IEC or local regulations.

The Investigator will notify the IRB/IEC of the conclusion of the clinical study within 1 month of completion or termination of the study. The final report sent to the IRB/IEC will also be sent to sponsor, along with the completed eCRFs and all necessary regulatory documents, thereby fulfilling the Investigator's regulatory responsibility.

The Investigator will maintain a copy of all correspondence with the IRB/IEC, including copies of approved documents. The Investigator will also maintain a copy of the IRB/IEC membership list, including members' occupation and qualifications (or a statement confirming compliance with GCP requirements for committee composition). An IRB or IEC General Assurance Number may be accepted in lieu of a membership roster.

## 15.2. Ethical Conduct of the Study

This study will be conducted in accordance with GCP as delineated by Title 21 CFR Parts 50, 56, and 312, and the ICH guidelines and directives. Participating Investigators, including members of the Committees and National Coordinators, will receive compensation for their time but will receive no financial profit from their activities related to the study.

Before the first subject is enrolled in the study, all ethical, regulatory, and legal requirements must be met.

An Investigator participating in this study is required to maintain complete and accurate study documentation in compliance with current GCP standards and all applicable local regulations related to the conduct of a clinical study.

## 15.3. Written Informed Consent

Prior to performing any study-related activities under this protocol, including screening tests and assessments, written informed consent with the approved ICF must be obtained from the subject or subject's legally authorized representative, as applicable, in accordance with local practice and regulations. Written informed consent must be obtained from all subjects participating in a clinical study conducted by Hua Medicine.

The background of the proposed study, the procedures, the benefits and risks of the study, and that study participation is voluntary for the subject must be explained to the subject. The subject must be given sufficient time to consider whether to participate in the study.

A copy of the ICF, signed and dated by the subject, must be given to the subject. Confirmation of a subject's informed consent must also be documented in the subject's source documentation prior to any testing under this protocol, including screening tests and assessments.

Each consent form should contain an authorization allowing the Principal Investigator(s) and the sponsor to use and disclose patient health information (PHI) in compliance with local law.

The originally signed consent form will be retained with the study records.

# 16. DATA HANDLING AND RECORD KEEPING

## 16.1. Data Collection

All data obtained for analysis in the clinical study described in this protocol will be documented in the eCRF. Data reported in the eCRFs should be consistent with and substantiated by the subject's medical record and original source documents.

Prior to the start of the study, the Principal Investigator will complete a Delegation of Authority form (Site Signature and Delegation Log), showing the signatures and handwritten initials of all individuals and the delegation of responsibilities, such as identifying those individuals who are authorized to make or change entries on eCRFs.

## 16.2. Case Report Form Completion

All eCRF entries, corrections, and alterations must be made by the investigator or authorized study-site personnel. Data within the eCRF will be monitored by the Clinical Research Associate according to the Monitoring Plan. Queries will be generated based on discrepancies found while monitoring. Site personnel will review and respond to these queries appropriately. Additionally, the sponsor may periodically perform aggregate data reviews, which could result in queries being generated for site personnel resolution. The final, completed eCRF for each subject must be signed and dated by the Principal Investigator to signify that the Principal Investigator has reviewed the eCRF and certifies it to be complete and accurate.

## 16.3. Database Management, Data Clarification, and Quality Assurance

A designated CRO will be responsible for data management. The Data Manager will develop a Data Management Plan (DMP) document and provide it to sponsor for approval. The DMP document will define all activities in the data collection and cleaning process. The detailed DMP will be based on the protocol, work scope, contract, analysis plans, data-flows, eCRFs, data cleaning procedures, other supporting documents, and data management standards and practices.

The programmed data validations will be run, and manual review will be conducted to check for database completeness and consistency, and queries will be generated upon data entry or via review by a Clinical Data Manager after entry. The sites will respond to the data queries in a timely manner.

Prior and concomitant medications will be coded using the World Health Organization (WHO) Drug Enhanced Dictionary (latest version, to be noted in DMP). All medical history and AEs will be coded using the Medical Dictionary for Regulatory Activities (MedDRA), using latest version, to be noted in DMP.

Quality control procedures will be conducted prior to database lock according to the designated CRO SOPs.

When the database has been declared to be complete and accurate, it will be locked. Any changes to the database after that time will only be made by joint written agreement between Hua Medicine, the study Statistician, the Data Manager, and the Quality Assurance Auditor according to designated CRO SOPs.

## 16.4. Inspection of Records/Source Documents

According to the ICH guidelines for GCP, the sponsor or designee must verify data entered in the eCRF entries against the source documents, except for the pre-identified source data directly documented in the eCRF (if applicable). The ICF will include a statement by which the subject allows the sponsor's duly authorized personnel, the IRB/IEC, and the regulatory authorities to have direct access to source data that supports the data in the eCRF (e.g., subject's medical file, appointment books, and original laboratory records). These personnel, bound by professional secrecy, must keep confidential all personal medical information.

The objective of source document verification (SDV) is to comply with GCP and international regulatory requirements and to reduce the risks of fraud. Source document verification means ensuring that the source documents are an accurate and verifiable reflection of the subject's participation in the study and that all relevant information that is recorded in the source document is accurately entered into the eCRF.

Where source documents serve as the basis for deriving data for the study, SDV should ensure that these documents are correctly labeled and filed, and that the data derived from them are correct. All source documents pertaining to this study will be maintained by the Investigator and made available for inspection by authorized persons. If electronic progress notes and other electronic source documents are not Title 21 CFR Part 11 compliant, they are not considered a valid source for this study. All progress notes must be dated and signed by the Principal

Investigator or sub-Investigator at the time of the visit. The sponsor reserves the right to terminate the study for refusal of the Investigator to supply original source documentation for this clinical study.

The Investigator will note in a source independent from the eCRF the following information:

1. Information to confirm that the subject exists (e.g., initials, date of birth, sex)
2. Confirmation that the subject satisfies the inclusion/exclusion criteria
3. Confirmation that the subject is taking part in the clinical study
4. Confirmation of the informed consent process
5. Visit dates and documentation of protocol assessments and procedures
6. Information concerning all AEs
7. Details of concomitant and investigational medications

Source document verification is not a substitute for clinical study monitoring, the purpose of which is to ensure that the protocol has been followed correctly, the eCRF has been fully and accurately completed, SDV has been carried out, and the study timelines and enrollment goals and requirements have been met.

## 16.5. Retention of Records

The Investigator/institution must maintain all study documentation as confidential and take measures to prevent accidental or premature destruction of these documents.

Essential documents should be retained until at least 2 years after the last approval of a marketing application in an ICH region and until there are no pending or contemplated marketing applications in an ICH region or at least 2 years have elapsed since the formal discontinuation of clinical development of the investigational product (e.g., the Investigational New Drug application is withdrawn). These documents should be retained for a longer period, however, if required by the applicable regulatory requirements or by an agreement with sponsor.

The Investigator/institution must notify sponsor prior to destroying any study essential documents.

If the Investigator/institution can no longer ensure archiving, he/she shall inform the sponsor. The relevant records shall be transferred to a mutually agreed upon designee.

# 17. CONFIDENTIALITY

All information disclosed or provided by sponsor (or designee) or produced during the study including, but not limited to, the protocol, the eCRFs, the IB, and the results obtained during the course of the study (if applicable), are confidential. The Investigator or any person under his/her authority agrees to undertake to keep confidential and not to disclose the information to any third party without the prior written approval of sponsor.

However, submission of this protocol and any other necessary documentation to the IRB/IEC is expressly permitted, the IRB/IEC members having the same obligation of confidentiality.

All information generated in this study must be considered highly confidential and must not be disclosed to any persons not directly concerned with the study without prior written permission from sponsor. However, authorized regulatory officials and sponsor personnel (or designee) will be allowed full access to inspect and copy the records. The copied and inspected records will remain at the site and will not be transmitted or removed from the site.

All study drugs, subject bodily fluids, and/or other materials collected shall be used solely in accordance with this protocol, unless otherwise agreed to in writing by sponsor and responsible ethics committee(s) or regulatory authorities.

Subjects will be identified only by unique subject numbers in eCRFs. Their full names may, however, be made known to a regulatory agency or other authorized officials in the event of inspections. Documents containing the full name or other personally identifiable information of the subject are to remain at the site. This information will not be transferred to sponsor nor be contained in regulatory filings. In the event of inspections by authorized agencies, this subject identification may be disclosed.

## 17.1. Subject Data Protection

Prior to any testing under this protocol, including screening tests and assessments, candidates must also provide all authorizations required by local law (e.g., PHI authorization in North America).

The subject will not be identified by name in the eCRF or in any study reports and these reports will be used for research purposes only. The sponsor, its partner(s) and designee(s), and various

government health agencies may inspect the records of this study. Every effort will be made to keep the subject's personal medical data confidential.

The sponsor will protect individual subject information to the fullest extent possible during this study. At no time will a subject become identified in any publication or presentation.

However, the subject may have to become identified in the event of a regulatory authority auditor inspection in order to verify the accuracy of the data. Access to subject information is at the discretion of sponsor and cannot occur prior to database lock or other specified events as determined solely by the discretion of sponsor.

# 18. STUDY PROTOCOL AMENDMENTS

The Investigator will not make any changes to this protocol without prior written consent from sponsor and subsequent approval by the IRB/IEC. Any permanent change to the protocol, whether it is an overall change or a change for specific study center(s), must be handled as a protocol amendment. Any amendment to the protocol that appears necessary as the study progresses will be fully discussed by the Investigator(s) and sponsor. If agreement is reached regarding the need for an amendment, it will be written by sponsor. The written amendment must be submitted to the chairman of the IRB/IEC identified with this responsibility. Except for administrative amendments, Investigators must await IRB/IEC approval of protocol amendments before implementing the change(s).

Administrative amendments are defined as having no effect on the safety of the research subjects, scope of the investigation, or quality of the study. However, a protocol change intended to eliminate an apparent immediate hazard to subjects should be implemented immediately, and the IRB/IEC notified within 5 days. The sponsor will ensure submission of any protocol amendments to the appropriate regulatory agencies.

When, in the judgment of the chairman of the local IRB/IEC, the Investigators, and/or sponsor, the amendment to the protocol substantially alters the study design and/or increases the potential risk to the subject; the currently approved written ICF will require similar modification. In such cases, the Investigator will obtain repeat informed consent from subjects enrolled in the study before expecting continued participation.

# 19. PUBLICATION

All information concerning the product as well as any information such as clinical indications for the study drugs, their formula, their formulation, methods of manufacture and other scientific data relating to it, that have been provided by sponsor or designee, and are unpublished, are confidential and must remain the sole property of Hua Medicine.

The Investigator will agree to use the information only for the purposes of carrying out this study and for no other purpose unless prior written permission from sponsor is obtained. The sponsor has full ownership of the eCRFs completed as part of the study.

By signing the study protocol, the Investigator agrees that the results of the study may be used for the purposes of national and international registration, publication, and information for medical and pharmaceutical professionals by sponsor. If necessary, the authorities will be notified of the Investigator's name, address, qualifications, and extent of involvement.

The sponsor or designee will prepare a final report on the study. The Investigator may not publish or present any information on this study without first presenting the information to sponsor for review.

# 20. REFERENCES

1. Hua Medicine (Shanghai), Ltd. China: Investigator's Brochure for Dorzagliatin (HMS5552), Second edition, December 29, 2017.
2. Zhu D, Gan S, Liu Y, et. al. Dorzagliatin monotherapy in Chinese patients with type 2 diabetes: dose-ranging, randomised, double-blind, placebo-controlled, phase 2 study. Lancet Diabetes Endocrinol, 2018; 6 (8): 627-636.
3. [redacted] Package Insert, 2018, [redacted] USA
4. [redacted] Package Insert, 2018, [redacted] USA.
5. [redacted] Package Insert, 2017, [redacted] USA.
6. [redacted] Package Insert, 2017, [redacted] USA.

7. [redacted] Package Insert, 2017, [redacted] USA.

8. [redacted] Package Insert, 2017, [redacted] USA.

9. [redacted] Package Insert, 2017, [redacted] USA.

10. Chung I, Oh J, Lee SH, et. al. A post hoc analysis of intra-subject coefficients of variation in pharmacokinetic measures to calculate optimal sample sizes for bioequivalence studies. Transl Clin Pharmacol, 2017; 25 (4): 179-182.

# APPENDIX A. KNOWN CLINICAL INHIBITORS AND INDUCERS FOR CYP3A4*

| **1. Clinical Inhibitors** | | |
|---|---|---|
| **Strong Inhibitors** | **Moderate Inhibitors** | **Weak Inhibitors** |
| **Antiviral:** | **Antifungal:** | **Muscle relaxant:** |
| Boceprevir | Clotrimazole | Chlorzoxazone |
| Cobicistat | Fluconazole | **Anticoagulant:** |
| Ritonavir | **Antibiotics:** | Cilostazol |
| Telaprevir | Ciprofloxacin | Ticagrelor |
| Nelfinavir | Erythromycin | **Anti-gastrointestinal abnormalities:** |
| Danoprevir and Ritonavir | **Immunological:** | |
| Elvitegravir and Ritonavir | Cyclosporine | Fosaprepitant |
| Indinavir and Ritonavir | **Antineoplastic:** | Ranitidine |
| Paritaprevir and Ritonavir and (Ombitasvir and/or Dasabuvir) | Crizotinib | **Central nervous system:** |
| | Imatinib | Istradefylline |
| Saquinavir and Ritonavir | **Anti-cardiovascular abnormalities:** | **Antineoplastic:** |
| Lopinavir and Ritonavir | | Ivacaftor |
| Tipranavir and Ritonavir | Dronedarone | **Lipid lowering:** |
| **Antineoplastic:** | Verapamil | Lomitapide |
| Idelalisib | **Antipsychotic disorder:** | **Anti-cardiovascular abnormalities:** |
| **Antifungal:** | Fluvoxamine | |
| Itraconazole | Tofisopam | Ranolazine |
| Ketoconazole | **Anti-gastrointestinal abnormalities:** | **Immunological:** |
| Posaconazole | | Tacrolimus |
| Voriconazole | Aprepitant | |
| **Antibiotics:** | Cimetidine | |
| Troleandomycin | | |
| Clarithromycin | | |
| **Anti-cardiovascular abnormalities:** | | |
| Conivaptan | | |
| Diltiazem | | |
| **Antipsychotic disorder:** | | |
| Nefazodone | | |

| **2. Clinical Inducers** | | |
|---|---|---|
| **Strong Inducers** | **Moderate Inducers** | **Weak Inducers** |
| **Anti-epileptic:**<br>Carbamazepine<br>Phenytoin sodium<br>**Antineoplastic:**<br>Enzalutamide<br>Mitotane<br>**Antipsychotic disorder:**<br>Hyperforin perforatum (St. John's wort)<br>**Antituberculous:**<br>Rifampin | **Antiviral:**<br>Efavirenz<br>Etravirine<br>**Anti-pulmonary hypertension:**<br>Bosentan<br>**Treatment of narcolepsy:**<br>Modafinil | **Wake-promoting agents:**<br>Armodafinil<br>**Anti-epileptic:**<br>Rufinamide |

*FDA's Web site on Drug Development and Drug Interactions can be found at: https://www.fda.gov/Drugs/DevelopmentApprovalProcess/DevelopmentResources/DrugInteractionsLabeling/ucm093664.htm

# APPENDIX B. LABORATORY ASSESSMENTS

| Hematology | Clinical Chemistry | Urinalysis |
|---|---|---|
| Hemoglobin (Hgb) | Blood urea nitrogen (BUN) | pH |
| Hematocrit (Hct) | Creatinine | Specific gravity |
| Platelet cout | Total bilirubin | Protein |
| Red blood cell (RBC) count | Alkaline phosphatase | Glucose |
| White blood cell (WBC) count | Aspartate transaminase (AST) | Ketones |
| | Alanine transaminase (ALT) | Bilirubin |
| with differential | Gamma-glutamyl | Blood |
| | transferase (GGT) | Nitrites |
| | Lactic dehydrogenase (LDH) | Leukocytes |
| | Glucose | Urobilinogen |
| | Albumin | Microscopic urine analysis |
| | Total protein | |
| | Bicarbonate | |
| | Phosphate | |
| | Sodium | |
| | Potassium | |
| | Chloride | |
| | Calcium | |
| | Total cholesterol | |
| | Triglyceride | |
| | HDL-C | |
| | LDL-C | |
| | Urate | |

| **Urine/Saliva Drug Screen** | **Serology Screen** |
|---|---|
| Amphetamines | Human immunodeficiency virus (HIV) |
| Barbiturates | Hepatitis B surface antigen (HBsAg) |
| Cannabinoids | Hepatitis C virus (HCV) |
| Cocaine metabolites | **Other** |
| Opiates | Urine Pregnancy test (all women, screening, Day -1, End-of-Study) |
| Benzodiazepines | Serum FSH and estradiol (postmenopausal women, screening) |
| Ethyl alcohol | HbA1c test (screening only) |
| | C-peptide test (screening only) |
| | **PD assessments** |
| | Glucose |
| | GLP-1 |
| | Glucagon |
| | Insulin |
| | C-peptide |

# TABLE OF CONTENTS

TABLE OF CONTENTS ............ 3
1. PROTOCOL SYNOPSIS ............ 8
2. LIST OF ABBREVIATIONS ............ 14
3. INTRODUCTION ............ 17
3.1 Background Information ............ 17
3.2 Preclinical Data ............ 18
3.3 Summary of Clinical Studies ............ 19
3.4 Drug Interaction Potential of Dorzagliatin ............ 22
4. STUDY RATIONALE AND OBJECTIVES ............ 24
4.1. Study Rationale ............ 24
4.2. Primary Objectives ............ 24
4.3. Secondary Objective ............ 25
5. INVESTIGATIONAL PLAN ............ 25
5.1. Overall Study Design ............ 25
5.2. Number of Subjects ............ 29
5.3. Treatment Assignment ............ 29
5.4. Criteria for Study Termination ............ 29
6. SELECTION AND WITHDRAWAL OF SUBJECTS ............ 29
6.1. Subject Inclusion Criteria ............ 29
6.2. Subject Exclusion Criteria ............ 31
6.3. Prohibitions and Restrictions ............ 33
6.4. Subject Withdraw Criteria ............ 33
7. STUDY PROCEDURES ............ 34
7.1. Screening Visit and Run-in Period ............ 35
7.2. Domiciled at CRC on Day -2 through Day 17 ............ 36
7.2.1. Study Check-in Visit: Day -2 and Day -1 ............ 36

7.2.2. Day 1 - Day 4 ..... 37

7.2.3. Day 5 ..... 38

7.2.4. Day 6 - Day 9 ..... 38

7.2.5. Day 10 ..... 39

7.2.6. Day 11 - Day 14 ..... 40

7.2.7. Day 15 ..... 40

7.2.8. Day 16 ..... 41

7.2.9. Day17: End-of-Study Procedures ..... 41

7.3. Early Withdrawal Visit ..... 41

7.4. Time Windows for Procedures ..... 42

8. TREATMENT OF SUBJECTS ..... 42

8.1. Description of Study Drugs ..... 42

8.2. Methods of Assigning Subjects to Dose Groups ..... 43

8.3. Blinding ..... 43

8.4. Treatment Discontinuation/Subject Withdrawal ..... 43

8.5. Prior and Concomitant Medications and Substances ..... 43

8.6. Meals ..... 43

9. STUDY DRUG MATERIALS AND MANAGEMENT ..... 44

9.1. Study Drug Packaging and Labeling ..... 44

9.2. Study Drug Storage and Accountability ..... 44

9.3. Study Drug Preparation ..... 45

9.4. Study Drug Handling and Disposal ..... 45

10. PHARMACOKINETIC/PHARMACODYNAMIC ASSESSMENTS ..... 45

10.1. Pharmacokinetic Sample Collection and Storage ..... 45

10.2. Pharmacokinetic Specimen Labeling ..... 46

10.3. Pharmacokinetic Sample Shipping Instructions .......... 46

10.4. Bioanalytical Methodology .......... 47

10.5. Pharmacokinetic Parameters .......... 47

10.6. Pharmacodynamic Assessments .......... 47

11. ASSESSMENT OF SAFETY .......... 48

11.1. Safety Parameters .......... 48

11.1.1. Demographic/Medical History .......... 48

11.1.2. Vital Signs .......... 49

11.1.3. Physical Examination, Height, Weight, and Body Mass Index .......... 49

11.1.4. Electrocardiogram .......... 49

11.1.5. Clinical Laboratory Assessments .......... 50

11.2. Adverse Events .......... 51

11.2.1. Definitions of Adverse Events .......... 51

11.2.2. Recording Adverse Events .......... 54

11.2.3. Reporting of Serious Adverse Events .......... 55

11.2.4. Adverse Event Follow-up .......... 55

11.2.5. Hy’s Law .......... 56

11.2.6. Hypoglycemia .......... 56

12. STATISTICAL METHODOLOGY .......... 56

12.1. Sample Size Determination .......... 56

12.2. Analysis Population .......... 57

12.3. Pharmacokinetics and Pharmacodynamics .......... 57

12.4. Demographic Characteristics .......... 58

12.5. Exposure to Study Drugs .......... 58

12.6. Concomitant Medications .......... 59

12.7. Safety Analyses ... 59

12.8. Interim Analyses ... 59

13. DIRECT ACCESS TO SOURCE DATA/DOCUMENTS ... 59

13.1. Study Monitoring ... 59

13.2. Sponsor's Responsibility ... 60

13.3. Audits and Inspections ... 60

14. QUALITY CONTROL AND QUALITY ASSURANCE ... 61

15. ETHICS ... 61

15.1. Ethics Review ... 62

15.2. Ethical Conduct of the Study ... 63

15.3. Written Informed Consent ... 64

16. DATA HANDLING AND RECORD KEEPING ... 64

16.1. Data Collection ... 64

16.2. Case Report Form Completion ... 64

16.3. Database Management, Data Clarification, and Quality Assurance ... 65

16.4. Inspection of Records/Source Documents ... 65

16.5. Retention of Records ... 67

17. CONFIDENTIALITY ... 67

17.1. Subject Data Protection ... 68

18. STUDY PROTOCOL AMENDMENTS ... 68

19. PUBLICATION ... 69

20. REFERENCES ... 70

APPENDIX A. LABORATORY ASSESSMENTS ... 71

# TABLES

Table 5-1 Schedule of Procedures ... 27

Table 5-2: Treatment Assignment and PK Sample Collection ... 29

Table 10-1: PK Terms ... 47

# Summary of Changes

| Change from Version 1.1 (27 December 2018) to Version 1.2 (04 February 2019 | |
|---|---|
| **Section** | **Change** |
| 1.0 Synopsis<br>6.1 Subject Inclusion Criteria | Inclusion Criterion #1 clarified to avoid misinterpretation. Deleted word "within", changing from "Subjects diagnosed with T2DM **within** at least 3 months prior to screening and are currently taking a stable dose…." to "Subjects diagnosed with T2DM at least 3 months prior to screening and are currently taking a stable dose….". |
| Main changes from Version 1.0 (11 November 2018) to Version 1.1 (27 December 2018) | |
| **Section** | **Change** |
| 1.0 Synopsis<br>4.3 Secondary Objective, and throughout protocol. | Wording of secondary objective changed to remove "serum" since some PD analytes are measured in plasma and some are measured in serum.<br>Throughout protocol, when matrix of PD analytes is noted, wording changed from "serum levels of GLP-1, glucagon, glucose, insulin, and C-peptide" to "GLP-1 and glucagon in plasma, and glucose, insulin, and C-peptide in serum". |
| 5.1 Overall Study Design | Schedule of Procedures footnote to Run-in Period amended to reflect that sitagliptin may be dispensed to eligible subjects prior to Day -14. |
| 5.2 Number of Subjects | Changed "Evaluable subjects are defined as subjects included in the **PD** population…" to "Evaluable subjects are defined as subjects included in the **DDI** population…" |
| 6.1 Subject Inclusion Criteria | Removed "screening" to clarify that criteria applies to screening and/or Day -1. |
| 6.2 Subject Exclusion Criteria | Removed "screening" to clarify that criteria applies to screening and/or Day -1. |
| 7.2.1 Study Check-in Visit | Added physical examination on Day -1 to be consistent with remainder of protocol. |
| 7.2.3-7.2.9 (Day 5-Day 17) | Changed 18-hour post-dose PK blood draw to occur on day after dosing.<br>Removed blood glucose assessment by glucometer on Day 17 to be consistent with remainder of protocol. |
| 10.6 Pharmacodynamic Assessments | Changed volume of blood collected for PD measurements: 8.5 mL (instead of 8.0 mL) collected for measurement of plasma GLP-1 and glucagon, and 7 mL (instead of 6 mL) collected for measurement of serum glucose, insulin and C-peptide.<br>Sample collection and processing steps for measurement of GLP-1 and glucagon added. |

# 1. PROTOCOL SYNOPSIS

| | |
|---|---|
| **Name of Sponsor/Company:**<br>Hua Medicine (Shanghai) Ltd. | |
| **Name of Investigational Product:**<br>Dorzagliatin | |
| **Name of Active Ingredient:**<br>Dorzagliatin: (S)-2-[4-(2-Chloro-phenoxy)-2-oxo-2,5-dihydro-pyrrol-1-yl]-4-methyl-pentanoic acid [1-((R)-2,3-dihydroxy-propyl)-1H-pyrazol-3-yl]-amide | |
| **Title of Study:**<br>A Phase 1, Open-Label, Sequential, Multiple-Dose, Drug-Drug Interaction Study of Dorzagliatin and Sitagliptin in Subjects with Type 2 Diabetes Mellitus | |
| **Study Center(s):** | |
| **Site:** Frontage Clinical Services, Inc., Secaucus, NJ<br>**Principal Investigator:** [redacted]<br>**Sub-investigator:** [redacted] | **Site:** To be Determined |
| **Studied Period (years):**<br>Estimated date first subject enrolled: Dec 2018<br>Estimated date last subject completed: Mar 2019 | **Phase of development:** Phase 1 |
| **Objectives:**<br>**Primary:**<br>• To assess the potential pharmacokinetic interaction between dorzagliatin and sitagliptin in subjects with type 2 diabetes mellitus;<br>• To evaluate the safety and tolerability of dorzagliatin with simultaneous administration of sitagliptin in subjects with type 2 diabetes mellitus.<br>**Secondary:**<br>• To assess the pharmacodynamic responses of glucose, GLP-1, glucagon, insulin, and C-peptide following dorzagliatin, sitagliptin, or simultaneous administration of dorzagliatin and sitagliptin in subjects with type 2 diabetes mellitus | |
| **Methodology:**<br>This is a Phase 1, open-label, sequential, multiple-dose, drug-drug interaction (DDI) study of glucokinase (GK) activator dorzagliatin and sitagliptin in subjects with type 2 diabetes mellitus (T2DM).<br>Study drugs will be administered in the following treatment scheme: | |

| Day | Sitagliptin | Dorzagliatin | PK Sample Collection |
|---|---|---|---|
| 1-5 | 100 mg (QD) for 5 days | - | Day 5:<br>Sitagliptin alone |
| 6-10 | 100 mg (QD) for 5 days | 75 mg (BID) for 4 days<br>Morning dose only on Day 10 | Day 10:<br>Sitagliptin + Dorzagliatin |
| 11-15 | - | 75 mg (BID) for 4 days<br>Morning dose only on Day 15 | Day 15:<br>Dorzagliatin alone |

Study drug will be taken 60 (±5) minutes prior to meals.

Eligible subjects will have a minimum 12-day run-in period prior to admission to the Clinical Research Center (CRC), during which time they will self-administer sitagliptin 100 mg QD each morning up until and including Day -2. Sitagliptin will be dispensed by the CRC. Subjects will complete a diary to record sitagliptin doses taken each day and will be advised to monitor their blood glucose levels and report changes in health status to the CRC. A telephone call will be placed to all subjects at approximately Day 6 (±2 day) of the run-in period to assess general health and collect adverse event (AE) information.

Following completion of the run-in period, eligible subjects will be admitted to the CRC on Day -2, have a total of 18 overnight stays, and be discharged after End-of-Study procedures are completed on Day 17, or at early termination.

Blood samples for the determination of dorzagliatin and sitagliptin concentrations and corresponding pharmacokinetic (PK) analysis will be collected at the following time points on Days 5, 10 and 15: pre-dose [redacted] and [redacted] post dose.

Subjects will consume a 75-gram glucose solution 30 minutes following study drug dose administration on Days 5, 10 and 15, in lieu of breakfast. Pharmacodynamic (PD) responses will be evaluated by measuring Glucagon-like Peptide-1 (GLP-1) and glucagon in plasma, and glucose, insulin, and C-peptide in serum within 60 minutes prior to oral glucose intake and 0.5, 1, 1.5, 2, 2.5, 3 and 4 hours after oral glucose intake.

Safety assessments will include monitoring of AEs, blood glucose via glucometer readings, vital signs (blood pressure, pulse rate, respiratory rate and oral temperature), clinical laboratory findings, resting 12-lead electrocardiograms (ECGs), and physical examination (PE) findings.

Vital signs will be assessed at screening, on Day -1, on Days 1-15 within 60 minutes prior to each morning and evening dose, and on Day 17. Blood glucose will be measured using a glucometer three times each day on Days -2 (prior to first meal after admission) through Day 16, within 60 minutes before each meal and after study drug dosing when applicable. Clinical laboratory evaluations (chemistry, hematology, urinalysis) will be performed at screening, on Days -1, 5, 10, 15 and 17. A resting 12-lead ECG will be completed at screening, on Day -1, and 2 hours post-dose on Days 5, 10, 15 and 17. Physical examinations will be conducted at screening, on Day -1, and on Day 17.

Subjects who terminate study participation early will have Day 17 assessments performed, if possible, prior to discharge from the CRC.

**Number of Subjects (planned):**

This study will plan to enroll up to 15 eligible subjects. Subjects may be enrolled singly or in groups. In order to have at least 12 evaluable subjects complete the study, additional subjects may be enrolled to replace subjects who discontinue study participation prematurely.

**Inclusion Criteria:**

1. Subjects diagnosed with T2DM at least 3 months prior to screening and are currently taking a stable dose of either of the following therapy with no change in the dose for at least 4 weeks prior to screening:
   a. ≥1000 mg per day of metformin
   b. a dipeptidyl peptidase 4 (DPP-4) inhibitor
   c. a sodium-glucose cotransporter-2 (SGLT2) inhibitor
   d. metformin plus a DPP-4 inhibitor
   e. metformin plus a SGLT2 inhibitor
2. Subjects must agree to change their current therapy to 100 mg sitagliptin QD for at least 14 days prior to dosing on Day 1;
3. In stable general health as determined by the Investigator at screening evaluation performed no more than 42 days prior to dosing on Day 1;
4. Male and/or female subjects between the ages of 30 and 65 years, inclusive;
5. Are capable of giving informed consent and complying with study procedures;
6. Body Mass Index (BMI) of 19 to 38 kg/m$^2$, inclusive, at screening;
7. Fasting C-peptide test result >0.3 nmol/L (>0.90 ng/mL);
8. HbA1c ≥7% and ≤10.5%;
9. Females must be using one of the following birth control methods: a non-hormonal intrauterine device (IUD) in place for at least 3 months; barrier method (condom, diaphragm) with spermicide for at least 14 days prior to dosing on Day 1 and throughout the study; surgical sterilization of the partner (vasectomy for 6 months minimum); OR are surgically sterile OR postmenopausal, as defined below:
   a. Surgically sterile for at least 3 months prior to screening by one of the following means:
      - Bilateral tubal ligation
      - Salpingectomy (with or without oophorectomy)
      - Surgical hysterectomy
      - Bilateral oophorectomy (with or without hysterectomy)
   b. Postmenopausal, defined as following:
      - Last menstrual period greater than 12 months prior to screening, confirmed by FSH and estradiol levels at screening;
10. Nonsmoker, defined as not having smoked or used any form of tobacco for more than 6 months prior to screening;
11. Willing and able to adhere to study restrictions and to be confined at the clinical research center.

**Exclusion Criteria:**

1. Fasting blood glucose at screening or Day -1 ≤110 or ≥270 mg/dL (one repeat screening fasting blood glucose allowed);
2. Type 1 diabetes mellitus, or latent autoimmune diabetes in adults; diabetic neuropathy, retinopathy or nephropathy;
3. Reported incidence of severe hypoglycemia, defined by denoting severe cognitive impairment requiring external assistance for recovery, within 3 months prior to screening and/or within 3 months prior to dosing on Day 1;
4. Known contraindications to sitagliptin;

5. Clinically significant gastrointestinal disorder (e.g., gastroparesis, constipation, diarrhea, colitis, gastrointestinal tract obstruction, hiatal hernia with reflux esophagitis, or cholestasis);
6. History or symptoms of clinically significant cardiovascular disease, particularly coronary artery disease, arrhythmias, atrial tachycardia, or congestive heart disease within one year prior to screening;
7. History of more than three urinary tract infections and/or more than three genital fungal infections in the last 12 months. If one or more genital fungal infections in the last 12 months required systemic treatment to resolve, Investigator will determine eligibility based on treatment required;
8. Reported history of clinically significant central nervous system disease including: transient ischemic attack, stroke, seizure disorder, depression, or behavioral disturbances within one year prior to screening;
9. Reported history of liver disease (e.g., hepatitis, cirrhosis within one year of screening and/or liver enzymes (alanine aminotransferase (ALT), aspartate aminotransferase (AST), gamma-glutamyltransferase (GGT), total bilirubin (TBiL)) >2 times the upper limit of normal (ULN));
10. Reported history of clinically significant renal disease;
11. Estimated glomerular filtration rate (eGFR) ≤60 mL/min/1.73m$^2$;
12. Acute or chronic metabolic acidosis, including diabetic ketoacidosis;
13. Known or suspected malignancy;
14. Any reported hypersensitivity (anaphylaxis and angioedema) or intolerance to sitagliptin;
15. Antidiabetic treatment with insulin, sulfonylureas, thiazolidinediones or GLP-1 agonist within 3 months prior to screening;
16. Systolic blood pressure <90 or ≥160 mmHg or diastolic blood pressure <60 or ≥100 mmHg at screening (may be repeated once);
17. A hospital admission or major surgery within 90 days prior to screening;
18. Uncontrolled hypertriglyceridemia >500 mg/dL;
19. Positive blood screen for human immunodeficiency virus (HIV), hepatitis B surface antigen (HBsAg), or hepatitis C antibody;
20. Positive pregnancy test result;
21. Female is breast-feeding or planning to become pregnant;
22. Treated with any investigational drugs within 6 weeks prior to screening;
23. Reported history of prescription drug abuse, or illicit drug use within 6 months prior to screening;
24. Reported history of alcohol abuse according to medical history within 6 months prior to screening;
25. A positive screen for alcohol or drugs of abuse;
26. Reported history of donation or blood collection of more than 1 unit (approximate 450 mL) of blood (or blood products) or acute loss of blood during the 90 days prior to screening;
27. Use of prescription or over-the-counter (OTC) medications, and herbal (including St John's Wort, herbal teas, garlic extracts) within 14 days prior to dosing (except for sitagliptin dosing during run-in period). No angiotensin-converting enzyme (ACE) inhibitors are allowed; (Note: Use of acetaminophen at <1 g/day is permitted until 24 hours prior to dosing);
28. An unwillingness of male participants to use appropriate contraceptive measures if engaging in sexual intercourse with a female partner of childbearing potential. Appropriate measures include use of a condom and spermicide and, for female partners, use of an IUD, diaphragm with spermicide, oral contraceptives, injectable progesterone, progesterone subdermal implants, or a tubal ligation. Sexual intercourse with pregnant or lactating women is prohibited.

**Study Drugs, Dosage and Mode of Administration:**

Dorzagliatin will be provided as a film-coated tablets in 75 mg strength for oral administration 60 minutes prior to a meal.

Sitagliptin will be provided as [redacted]® 100 mg tablets for oral administration 60 minutes prior to a meal.

**Duration of Treatment:**

The total duration of participation in the study for each subject is about 59 days (up to 28-day screening period, 12-day run-in period and 19-day in-clinic period).

**Criteria for Evaluation:**

**Safety:**

Safety assessments will include monitoring of AEs, blood glucose via glucometer readings, vital signs (blood pressure, pulse rate, respiratory rate and oral temperature), clinical laboratory findings, 12-lead ECGs, and PE findings.

**Pharmacokinetics:**

The plasma concentration-time data for dorzagliatin and sitagliptin will be analyzed using non-compartmental methods to calculate pharmacokinetic parameters. Actual dosing and sampling times will be used for analyses. The primary PK parameters of interest are: $C_{max}$, $T_{max}$ and $AUC_{0\text{-}24h}$. Additional parameters may be estimated and reported, as appropriate.

**Pharmacodynamics:**

Pharmacodynamic (PD) responses will be evaluated by measurement of GLP-1 and glucagon in plasma, and glucose, insulin and C-peptide in serum. The PD parameters may include, but not limited to, $AUEC_{0\text{-}4h}$, $CE_{max}$ and $CE_{av}$.

**Statistical Methods:**

Baseline demographic, concentration and safety data will be listed and summarized by descriptive statistics (n, mean, standard deviation, median, minimum, maximum) or frequency counts. Descriptive statistics will be calculated for dorzagliatin and sitagliptin concentrations in plasma.

Dorzagliatin and sitagliptin PK parameters ($C_{max}$, $T_{max}$ and $AUC_{0\text{-}24h}$) will be computed for each subject using non-compartmental methods based on the actual sampling times. Descriptive statistics will be calculated for the PK parameters for Days 5, 10 and 15.

The effect of sitagliptin on the exposure of dorzagliatin will be determined by a PK comparison between the exposure of dorzagliatin on Day 10 vs. Day 15. The effect of dorzagliatin on the exposure of sitagliptin will be determined by a PK comparison between the exposure of sitagliptin on Day 10 vs. Day 5.

The primary PK comparison will be based on the $C_{max}$ and $AUC_{0\text{-}24h}$ of dorzagliatin and sitagliptin. A mixed model under the sequential design will be used for analyzing $C_{max}$ and $AUC_{0\text{-}24h}$ values, based on natural log-transformed values, with treatments as fixed effects and subjects as random effects. The adjusted geometric mean ratios between two treatments for these parameters and the corresponding 90% confidence intervals (CI) for the ratio will be obtained by exponentiating the adjusted mean difference in logarithms and will be used for assessing PK differences between the two treatments within each PK comparison.

Baseline corrected PD markers will be calculated by subtracting the baseline value from the values post oral glucose intake. Plots of mean PD markers versus the scheduled time will be generated for each treatment. Derivations and statistical analysis methods of PD parameters ($AUEC_{0\text{-}4h}$, $CE_{max}$ and $CE_{av}$) will be described in detail in Statistical Analysis Plan (SAP).

| |
|---|
| This study will be conducted in accordance with the guidelines of Good Clinical Practices (GCPs) including archiving of essential documents. |

# 2. LIST OF ABBREVIATIONS

The following abbreviations and specialist terms are used in this study protocol.

| Abbreviation | Term |
|---|---|
| ACE | Angiotensin-Converting Enzyme |
| AE | Adverse Event |
| ALT | Alanine Aminotransferase |
| ANOVA | Analysis of Variance |
| API | Active Pharmaceutical Ingredient |
| AST | Aspartate Aminotransferase |
| AUC | Area Under the Concentration-Time Curve |
| $AUC_{0-24h}$ | Area Under the Concentration-Time Curve from 0 to 24 Hours |
| $AUEC_{0-4h}$ | Area Under the Effect-Time Curve of Change from Baseline |
| BID | Twice Daily |
| BMI | Body Mass Index |
| BP | Blood Pressure |
| BUN | Blood Urea Nitrogen |
| $C_{max}$ | Observed Maximum Plasma Concentration |
| $CE_{av}$ | Average Change from Baseline |
| $CE_{max}$ | Maximum Change from Baseline |
| CFDA | China Food and Drug Administration |
| CFR | Code of Federal Regulations |
| CI | Confidence Interval |
| CL/F | Apparent Total Plasma Clearance of Drug after Oral Administration |
| CNS | Central Nervous System |
| CRC | Clinical Research Center |
| CRO | Contract Research Organization |
| CYP | Cytochrome P450 |
| DDI | Drug Drug Interaction |
| DMP | Data Management Plan |
| DPP-4 | Dipeptidyl Peptidase-4 |
| ECG | Electrocardiography |
| eCRF | Electronic Case Report Form |
| eGFR | Estimated Glomerular Filtration Rate |
| EOS | End-of-Study |
| FDA | Food and Drug Administration |

| Abbreviation | Term |
|---|---|
| FSH | Follicle Stimulating Hormone |
| GCP | Good Clinical Practice |
| GGT | Gamma-Glutamyl-Transferase |
| GK | Glucokinase |
| GKA | Glucokinase Activator |
| GLP | Good Laboratory Practice |
| GLP-1 | Glucagon-Like Peptide 1 |
| GMP | Good Manufacturing Practice |
| GSIR | Glucose Stimulated Insulin Release |
| HbA1c | Hemoglobin A1c |
| HBsAg | Hepatitis B Surface Antigen |
| Hct | Hematocrit |
| HCV | Hepatitis C Virus |
| HDL | High-Density Lipoprotein |
| hERG | Human Ether-a-go-go-Related Gene |
| Hgb | Hemoglobin |
| HIV | Human Immunodeficiency Virus |
| HLM | Human Liver Microsome(s) |
| IB | Investigational Brochure |
| $IC_{50}$ | The Half Inhibitory Concentration |
| ICF | Informed Consent Form |
| ICH | International Conference on Harmonisation |
| IEC | Independent Ethics Committee |
| IND | Investigational New Drug Application |
| IRB | Institutional Review Board |
| IUD | Intrauterine Device |
| $K_{el}$ | Elimination Rate Constant |
| LC-MS/MS | Liquid Chromatography-Tandem Mass Spectrometry |
| LDH | Lactate Dehydrogenase |
| LDL | Low-Density Lipoprotein |
| MAD | Multiple Ascending Dose |
| MATE | Multidrug and Toxin Extrusion |
| MedDRA | Medical Dictionary for Regulatory Activities |
| MTD | Maximum Tolerated Dose |
| NOAEL | No Observed Adverse Effect Level |
| OAT | Organic Anion Transporter |

| Abbreviation | Term |
|---|---|
| OATP | Organic Anion Transporting Polypeptide |
| OGTT | Oral Glucose Tolerance Test |
| OTC | Over-The-Counter |
| P-gp | P-Glycoprotein |
| PHI | Protected Health Information |
| PIS | Patient Information Sheet |
| PK | Pharmacokinetic(s) |
| QC | Quality Control |
| QD | Once Daily |
| QTc | Corrected QT Interval |
| RBC | Red Blood Cell |
| $S_{0.5}$ | Substrate Concentration to Give ½ $V_{max}$ |
| SAD | Single Ascending Dose |
| SAE | Serious Adverse Event |
| SAP | Statistical Analysis Plan |
| SDV | Source Data Verification |
| SGLT2 | Sodium-Glucose Cotransporter-2 |
| SOP | Standard Operating Procedure |
| $T_{1/2}$ | Terminal Elimination Half-Life |
| T2DM | Type 2 Diabetes Mellitus |
| TBiL | Total Bilirubin |
| TDI | Time Dependent Inhibition |
| TEAE | Treatment-Emergent Adverse Event |
| $T_{max}$ | Time at which $C_{max}$ was first observed |
| ULN | Upper Limit of Normal |
| $V_{max}$ | Maximum Metabolic Rate |
| $V_z/F$ | Apparent Volume of Distribution during Terminal Phase after Oral Administration |
| WBC | White Blood Count |
| WHO | World Health Organization |

# 3. INTRODUCTION

## 3.1 Background Information

Hua Medicine (Shanghai) Ltd. (hereinafter “sponsor” or “Hua Medicine”) is developing dorzagliatin, an investigational novel glucokinase activator (GKA), indicated for use as an adjunct to diet and exercise to improve glycemic control in adults with type 2 diabetes mellitus (T2DM).

Glucokinase (GK) activators represent a promising new class of investigational drugs for the treatment of T2DM. Glucokinase activators lower blood glucose levels by enhancing the ability of pancreatic β-cells to “sense glucose” and increase insulin secretion in a glucose dependent manner. Simultaneously, GKAs can suppress glucose production and increase glucose utilization in the liver. Glucokinase activators may also function through other GK-expressing cells, such as entero-endocrine K and L-cells, and many GKAs have been shown to exert anti-apoptotic effects on β-cells.

Dorzagliatin (also referred to as HMS5552) is the 4th generation of GKAs. Dorzagliatin is an allosteric activator of GK which has been shown to increase the affinity of its substrate glucose by decreasing $S_{0.5}$ and increasing the $V_{max}$ of GK. Dorzagliatin has only a minor effect on the Hill coefficient nH and preserves the positive cooperativity of GK for glucose, a unique kinetic feature of GK. Dorzagliatin enhances glucose stimulated insulin release (GSIR) in rodent pancreatic islets and increases glucose uptake in cultured rodent primary hepatocytes. The selectivity profile of dorzagliatin was evaluated by screening 402 Ambit protein kinases and 78 CEREP receptors. In all cases the $IC_{50}$ values were [redacted], which was the highest concentration tested.[1]

Dorzagliatin is a potent glucose lowering agent showing excellent dose-related effects on fasting, basal and post-prandial glucose levels in several rodent models of T2DM, both in acute as well as chronic studies. Dorzagliatin augments GSIR and improves hepatic glucose disposal *in vivo*. Furthermore, treatment of normal mice with dorzagliatin has been associated with an increase in the levels of total Glucagon-like Peptide-1 (GLP-1). Therefore, dorzagliatin is a potential new antidiabetic agent for use as an adjunct to diet and exercise to improve glycemic control in adults with T2DM.

## 3.2 Preclinical Data

Preclinical evaluations including safety pharmacology, general toxicology studies in rats and dogs, reproductive toxicology, genetic toxicology were conducted following the guidance of International Conference on Harmonisation (ICH) and China Food and Drug Administration (CFDA), in compliance with the U.S. and CFDA Good Laboratory Practices (GLP) regulations. [1]

No QTc prolongation or dorzagliatin-related qualitative electrocardiographic events or abnormalities were observed at dose level up to [redacted] of dorzagliatin in safety pharmacology studies using telemetry technology in conscious dogs. [redacted] No adverse effect on central nervous system or respiratory system was observed in rats at [redacted] dorzagliatin (maximum dose).[1]

The repeat-dose toxicity and toxicokinetic profiles for orally administered dorzagliatin have been characterized in rats for 4 weeks, 13 weeks and 26 weeks and in dogs for 4 weeks, 13 weeks, and 39 weeks. [redacted] No adverse effect (including peripheral neuropathy) was found in dogs after dorzagliatin treatment [redacted]

No adverse effect on male reproductive organs and reproductivity was found in male fertility study [redacted]. [redacted]

[redacted] In the later phase of clinical trials, exclusive criteria have included the subjects who might potentially be pregnant.

No evidence for genotoxicity or mutagenicity of dorzagliatin was identified using in vitro Ames test and chromosomal aberration assay in human peripheral blood lymphocytes, and in vivo micronucleus test in rat bone marrow.[1]

These study results indicate that dorzagliatin has an adequate safety margin to support its development in the clinical setting.

### 3.3 Summary of Clinical Studies

To date, five clinical studies evaluating dorzagliatin have been completed, four of which were Phase 1 studies and one was a Phase 2 study. A total of 335 subjects have been exposed to dorzagliatin.[1]

Study HMM0101: In a Phase 1 Single Ascending Dose (SAD) study, a total of 48 healthy subjects received single oral doses of HMS5552 ranging from 5 mg to 50 mg. HMS5552 appeared to be safe and was well tolerated at all doses studied. No serious adverse events (SAEs) were reported and no withdrawal occurred due to an adverse event (AE). All AEs were mild in intensity and no treatment was required. [redacted]

$C_{max}$ and AUC were apparently proportional to dose. No marked gender difference of $C_{max}$ and AUC was noticed, and the PK profile of HMS5552 indicated it was suitable for twice daily (BID) administration.

Study HMM0102: In a Phase 1 Multiple Ascending Dose (MAD) study, a total of 43 T2DM subjects received multiple twice-daily oral doses of HMS5552 ranging from 25 mg to 200 mg for 8 days. HMS5552 appeared to be safe and was well tolerated at all doses. There was no apparent sign or change in the pattern of any clinical laboratory value, vital signs or ECG parameters. All AEs were mild in intensity. No severe AE or SAE was reported. The most common AEs were related to mild hypoglycemia. No subjects reported serious hypoglycemia. All subjects recovered quickly without requiring additional intervention. The PK of HMS5552 appeared to be dose-proportional over the range tested without appreciable drug accumulation or food-effect. [redacted]

[redacted]

<u>Study HMM0103:</u> In a Phase 1c study, 24 T2DM subjects received daily doses of 75 mg or 150 mg (75 mg BID) HMS5552 daily for 4 weeks. HMS5552 displayed excellent safety and tolerability in subjects who participated in the study. There were no incidents of SAE, death, early termination due to AEs or severe hypoglycemia. All AEs were considered mild in intensity. No clinically significant changes were observed in laboratory, 12-lead electrocardiogram (ECG) or physical examination (PE) tests. Consistent PK Profiles were observed as in the MAD study.

[redacted] renal excretion was not the major elimination pathway of HMS5552. [redacted]

Functions of pancreatic β-cells were improved in both groups 3 days after the 28-day treatment ended, compared with baseline. The sensitivity of pancreatic β-cells to blood glucose was enhanced after 7-8 drug $T_{1/2}$ had passed.

<u>Study HMM0104</u>: In a Phase 1 drug-drug interaction (DDI) study conducted in the US, 15 T2DM subjects received HMS5552 50 mg BID or metformin 500 mg BID alone or in combination for a total of 13 days (metformin alone for 3 days, dorzagliatin and metformin co-administration for 5 days, then dorzagliatin alone for 5 days). It appeared treatment with HMS5552 50 mg BID alone or co-administration with metformin 500 mg BID was safe and well-tolerated. There were no incidents of SAE, death, or early termination due to AEs. All treatment-emergent adverse events (TEAEs) were mild in intensity and subjects recovered after intervention. There was no clear evidence to support the causal relationship between the study drug and TEAEs, and no incidence of hypoglycemic events. No clinically significant changes were observed in laboratory tests, vital signs, ECGs or PEs in this study. Combined treatment

with metformin did not affect the PK of HMS5552. [redacted] Overall, the study demonstrated that there is no apparent clinically significant DDI between HMS5552 and metformin. In contrast, the combined treatment resulted in improved glycemic control in T2DM subjects compared to treatment with HMS5552 or metformin alone.

Study HMM0201: A dose-ranging, randomized, double-blind, placebo-controlled, Phase 2 study was conducted in Chinese patients with T2DM, aiming to identify a minimum effective dose of dorzagliatin in patients with type 2 diabetes.[1,2]

Two hundred fifty-eight (258) T2DM patients enrolled in the Phase 2 study and were randomly assigned to receive placebo (n=53), 75 mg dorzagliatin once daily (n=53), 100 mg dorzagliatin once daily (n=50), 50 mg dorzagliatin twice daily (n=51), or 75 mg dorzagliatin twice daily (n=51) for 12 weeks.

No deaths, drug-related SAEs, or drug-related severe AEs were reported. Most AEs were mild and considered unrelated to study medication by investigators. The incidence of AEs was similar among groups. Adverse events that occurred in ≥5% of patients in any group (including placebo) were upper respiratory tract infection, hyperuricemia, dizziness, protein present in urine, urinary tract infection, blood creatine phosphokinase increased, white blood cells (WBC) urine positive, hepatic function abnormal, high-density lipoprotein (HDL) decreased, ventricular extrasystole and nasopharyngitis. Incidence of hypoglycemia was low, with a rate of 5.4% for ≤3.9 mmol/L, and a rate of only 1% for ≥3.0 mmol/L in the Phase 2 study in the drug-treated groups. No severe hypoglycemia was reported.

[redacted]

In conclusion, preclinical pharmacology studies demonstrated that HMS5552 is an effective allosteric activator of GK both *in vitro* and *in vivo*. Safety pharmacology, general toxicity studies

in rats and dogs up to 26 weeks and 39 weeks, reproductive toxicology, genetic toxicology and carcinogenicity studies suggested that HMS5552 has an acceptable safety profile when projected for human use. Furthermore, safety data from clinical phase 1 and phase 2 studies conducted in China and US support the continued development of HMS5552.

## 3.4 Drug Interaction Potential of Dorzagliatin

In *in vitro* studies using human liver microsomes (HLM) dorzagliatin showed no inhibition of liver cytochrome P450 (CYP) 1A2, CYP2A6, CYP2C9, CYP2C19, CYP2D6, CYP2E1 and CYP3A4/5 activities ($IC_{50}$ >50 µM) at concentrations studied.

Drug interaction potential of dorzagliatin via CYP induction was evaluated in primary human hepatocytes and results indicated that dorzagliatin was unlikely to produce induction on CYP1A2 or CYP2B6. While an absence of induction of dorzagliatin on CYP3A4 could not be excluded from this vitro study, it was later confirmed in preclinical and clinical PK studies as no decrease in exposure of dorzagliatin was observed after repeated dosing.

*In vitro* results suggest that dorzagliatin is a substrate of P-glycoprotein (P-gp) but not a P-gp inhibitor. In addition, dorzagliatin did not show clinically relevant inhibitory effect on organic anion transporting polypeptide (OATP)1B1, OATP1B3, organic anion transporter (OAT)1 and OAT3, nor is dorzagliatin a substrate of these four transporters. These study results demonstrate that transporter-related DDI is unlikely.

Since dorzagliatin is predominantly metabolized by CPY3A4 and is a substrate of P-gp, it is predicted that exposure of dorzagliatin will be increased when co-administered with CYP3A4 inhibitors and decreased when co-administered with CYP3A4 inducers.[1]

Dipeptidyl peptidase-4 (DPP-4) inhibitors are a class of oral drugs for the treatment of patients with T2DM who have not responded well to drugs such as metformin and sulfonylureas. DPP-4 inhibitors block the action of DPP-4, an enzyme that destroys incretins. Incretins are gastrointestinal hormones that help stimulate insulin production. FDA-approved DPP-4 inhibitors include sitagliptin [redacted], saxagliptin [redacted] and linagliptin [redacted]. The reported half-lives of sitagliptin, saxagliptin (and active metabolite) and linagliptin are 12.4, 2.5 (and 3.1), and 12 hours (effective half-life) respectively.[3,4,5]

Sitagliptin is mainly (79%) cleared as parent drug by urine with metabolism being a minor pathway of elimination. In vitro studies indicated that the primary enzyme responsible for the limited metabolism of sitagliptin was CYP3A4, with additional contribution from CYP2C8. Sitagliptin is a substrate for OAT 3, which may be involved in the renal elimination of sitagliptin.

Sitagliptin is not an inhibitor of CYP3A4, 2C8, 2C9, 2D6, 1A2, 2C19 or 2B6, and is not an inducer of CYP3A4. Sitagliptin is a P-gp substrate but does not inhibit P-gp mediated transport of digoxin. Based on these results sitagliptin is considered unlikely to cause interactions with other drugs that utilize these pathways. Sitagliptin is not extensively bound to plasma proteins. Therefore, the propensity of sitagliptin to be involved in clinically meaningful DDIs mediated by plasma protein binding displacement is very low.[3]

Collectively, the available PK and metabolic information for dorzagliatin and sitagliptin summarized above suggest that the DDI potential between these two drugs when co-administered is low.

In this DDI study, prior to enrollment subjects must be taking a stable dose of either metformin alone, metformin in combination with a marketed brand of a sodium-glucose cotransporter-2 (SGLT2) inhibitor or a DPP-4 inhibitor, or a marketed brand of an SGLT2 or DPP-4 inhibitor as monotherapy.

Metformin[redacted] is an oral antihyperglycemic drug that is commonly recommended as the first-line pharmacotherapy for treatment of T2DM. Mean time to peak plasma concentration ($T_{max}$) following administration of a metformin tablet is about 2-3 hours. Renal excretion is the major route of metformin elimination. Following oral administration, approximately 90% of the absorbed drug is eliminated via the renal route within the first 24 hours, with a plasma elimination half-life of approximately 6.2 hours.[6]

Sodium-glucose cotransporter-2 inhibitors are a class of oral drugs for the treatment of patients with T2DM that lower blood sugar by blocking SGLT2, a glucose transporter in the kidney, and preventing the kidney from reabsorbing glucose and releasing it into the blood. FDA-approved SGLT2 inhibitors include canagliflozin[redacted], dapagliflozin [redacted] and empagliflozin [redacted]. The reported half-lives of canagliflozin, dapagliflozin and empagliflozin are 10-13.1, 12.9 and 12.4 hours, respectively.[7,8,9]

# 4. STUDY RATIONALE AND OBJECTIVES

## 4.1. Study Rationale

Although the available drug interaction and PK data of dorzagliatin and sitagliptin detailed in Section 3.4 do not suggest a potential drug interaction, other drug transporter-mediated interactions cannot be ruled out. Furthermore, pharmacodynamic-based interaction potential needs to be evaluated. Based on the high likelihood that dorzagliatin may be co-administered with sitagliptin in T2DM patients in a clinical setting, this study will provide clinical evidence of the DDI potential between these two drugs.

The basis for the 100 mg QD dosing regimen of sitagliptin is based on the recommended 100 mg once daily dosing of [redacted] for adults.[3] The 75 mg BID for dorzagliatin is the dose regimen proven to be safe and effective in Phase 1 and Phase 2 studies and selected for two current Phase 3 studies evaluating dorzagliatin as a mono-therapy and as an add-on treatment to metformin. In the present study, both drugs will be given for five days to ensure that steady-state is attained.

The sequential, multiple-dose study is designed to determine whether the steady-state pharmacokinetics of dorzagliatin and sitagliptin are affected, while at the same time whether there's a synergistic therapeutic effect between these two drugs when co-administered. The proposed dosing regimens with dorzagliatin and sitagliptin will allow the potential interactions to be assessed under conditions that are expected to provide maximal exposure at the doses being studied. The elimination half-lives of dorzagliatin and sitagliptin are relatively short which justifies the 24-hour interval for collecting all samples to characterize the pharmacokinetics of both drugs.

## 4.2. Primary Objectives

The primary objectives of this study are:

- To assess the potential pharmacokinetic interaction between dorzagliatin and sitagliptin in subjects with type 2 diabetes mellitus;
- To evaluate the safety and tolerability of dorzagliatin with simultaneous administration of sitagliptin in subjects with type 2 diabetes mellitus.

### 4.3. Secondary Objective

The secondary objective of this study is:

- To assess the pharmacodynamic responses of glucose, GLP-1, glucagon, insulin, and C-peptide following dorzagliatin, sitagliptin, or simultaneous administration of dorzagliatin and sitagliptin in subjects with type 2 diabetes mellitus.

# 5. INVESTIGATIONAL PLAN

### 5.1. Overall Study Design

This is a Phase 1, open-label, sequential, multiple-dose, DDI study of GK activator dorzagliatin and sitagliptin in subjects with T2DM.

It is planned that 15 subjects will be enrolled to have at least 12 evaluable subjects. All subjects will receive:

- Sitagliptin 100 mg QD in the morning on Days 1-5;
- Sitagliptin 100 mg QD in the morning and dorzagliatin 75 mg BID (morning and evening) on Days 6-10, with only the morning dose on Day 10;
- Dorzagliatin 75 mg BID (morning and evening) on Days 11-15, with only the morning dose on Day 15.

Eligible subjects will have a minimum 12-day run-in period prior to admission to the CRC, during which time they will self-administer sitagliptin 100 mg QD up until and including Day -2. Sitagliptin will be dispensed by the CRC. Sitagliptin will be purchased and provided by the sponsor. Subjects will complete a diary to record sitagliptin doses taken each day and will be advised to monitor their blood glucose levels and report changes in health status to the CRC. A telephone call will be placed to all subjects at approximately Day 6 (±2 day) of the run-in period to assess general health and collect AE information.

Following completion of the run-in period, eligible subjects will be admitted to the CRC on Day -2, have a total of 18 overnight stays, and be discharged after End-of-Study procedures are completed on Day 17, or at early termination.

Blood samples for the determination of dorzagliatin and sitagliptin concentrations and corresponding PK analysis will be collected at the following time points on Days 5, 10 and 15: [redacted].

Subjects will consume a 75-gram glucose solution 30 minutes following study drug dose administration on Days 5, 10 and 15, in lieu of a breakfast. Pharmacodynamic responses will be evaluated by measuring GLP-1 and glucagon in plasma, and glucose, insulin, and C-peptide in serum at baseline (prior to the study drug dose and within 60 minutes prior to oral glucose intake) and 0.5, 1, 1.5, 2, 2.5, 3 and 4 hours after oral glucose intake.

Safety assessments will include monitoring of AEs, blood glucose via glucometer readings, vital signs (blood pressure (BP), pulse rate (PR), respiratory rate (RR) and oral temperature (T)), clinical laboratory findings, resting ECGs, and PE findings.

Vital signs will be assessed at screening, on Day -1, on Days 1-15 within 60 minutes prior to each morning and evening dose, and on Day 17. Blood glucose will be measured using a glucometer three times each day on Days -2 (prior to first meal after admission) through Day 16, within 60 minutes before each meal and after study drug dosing when applicable. Clinical laboratory evaluations (chemistry, hematology, urinalysis) will be performed at screening, on Days -1, 5, 10, 15 and 17. A resting 12-lead ECG will be completed at screening, on Day -1, and 2 hours post-dose on Days 5, 10, 15, and 17. Physical exams will be conducted at screening, on Day -1, and on Day 17.

Subjects who receive their Day 1 dose and then terminate study participation early will have Day 17 assessments performed, if possible, prior to discharge from the CRC.

Morning dosing will occur at approximately 08:00 a.m. and evening dosing will occur at approximately 06:00 p.m. All doses will be administered 60 (±5) minutes prior to a standardized meal and with approximately 240 mL (8 fluid ounces) of room temperature water. On Days 5, 10 and 15 there will be no meal offered after the morning dose and instead the oral glucose solution will be administered 30 minutes after the study drug dose. The actual time of each dose, each post-dose meal, and time of oral glucose solution administration will be recorded.

See Table 5-1 for the details of all study procedures for subjects in the study.

## Table 5-1 Schedule of Procedures

| Visit | Screening | Run-in* | In-patient at the CRC* | | | | | | | | | |
|---|---|---|---|---|---|---|---|---|---|---|---|---|
| **Day** | **-42 to -15** | **-14 to -3** | **-2** | **-1** | **1-4** | **5** | **6-9** | **10** | **11-14** | **15** | **16** | **17 EOS** |
| Informed consent | X | | | | | | | | | | | |
| Dispense/collect sitagliptin, at-home sitagliptin QD 100 mg. Tel call at Day -8±2 (Day 6 of the 12-day run-in period)* | | X | X | | | | | | | | | |
| Admission to CRC | | | X | | | | | | | | | |
| Eligibility assessment | X | | | X | | | | | | | | |
| Demographics | X | | | | | | | | | | | |
| Medical history | X | | X | | | | | | | | | |
| Physical examination | X | | | X | | | | | | | | X |
| Height (cm), Weight (kg) | X | | | X[1] | | | | | | | | X[1] |
| Body mass index | X | | | | | | | | | | | |
| Standard 12-lead ECG[2] | X | | | X | | X | | X | | X | | X |
| Vital signs (BP, PR, RR, T) | X | | | X | Within 60 min prior to each study drug dose. | | | | | | | X |
| Clinical laboratory samples[3] | X | | | X | | X | | X | | X | | X |
| HIV, hepatitis B & C | X | | | | | | | | | | | |
| Urine drug /saliva alcohol | X | | X | | | | | | | | | |
| Urine pregnancy test[4] | X | | X | | | | | | | | | X |
| FSH and estradiol[4] | X | | | | | | | | | | | |
| OGTT/PD markers [5] | | | | | | X | | X | | X | | |
| C-peptide and HbA1c | X | | | | | | | | | | | |
| Dose administration[6] | | | | Sitagliptin 100 mg QD on Day -1; Assigned treatment Days 1-15 | | | | | | | | |
| AE & con med reporting[7] | | X | X | X | X | X | X | X | X | X | X | X |
| PK blood samples[8] | | | | | | X | X | X | X | X | X | |
| Glucometer (finger stick) | | | Within 60 minutes before each meal and after study drug dosing when applicable | | | | | | | | | |
| Discharge from CRC | | | | | | | | | | | | X |

AE= adverse event; BP = blood pressure; con med = concomitant medications; CRC = clinical research center; ECG = electrocardiogram; EOS =End-of-Study; FSH: Follicle stimulating hormone; HIV = human immunodeficiency virus; PR = pulse rate; PK = pharmacokinetics; RR = respiratory rate; T = temperature.

*Run-in period is a minimum of 12 days. If longer than 12 days, admission to CRC remains on Day -2. Sitagliptin will be dispensed by CRC to eligible subjects any day prior to start of Day -14 at-home dosing.

[1]Obtain weight only.

[2]ECG obtained in supine position after at least 5 minutes rest. On Days 5, 10 and 15 ECGs obtained 2 hours (±15 minutes) after dose administration.

[3]Clinical laboratory samples include: hematology, chemistry and urinalysis. All samples collected prior to study drug dose on days of study drug administration.

[4]For female subjects, urine pregnancy test must be negative to enroll in the study. Serum FSH and estradiol will be evaluated for postmenopausal females to confirm status.

[5] Oral glucose solution administered 30 (±5) minutes after study drug dose on Days 5, 10 and 15. PD samples to measure GLP-1 and glucagon in plasma, and glucose, insulin, and C-peptide in serum collected at baseline (prior to the study drug dose and within 60 minutes prior to oral glucose intake) and at 0.5, 1, 1.5, 2, 2.5, 3 and 4 hours after oral glucose intake. All post-glucose collection times are ±5 minutes from nominal time.

[6]All subjects will receive: sitagliptin 100 mg QD on Days 1-5 (a.m. dose); sitagliptin 100 mg QD and dorzagliatin 75 mg BID on Days 6-10 (a.m. dose only on Day 10); and dorzagliatin 75 mg BID on Days 11-15 (a.m. dose only on Day 15). Morning dosing will occur at approximately 08:00 a.m. and evening dosing will occur at approximately 06:00 p.m. All doses are 60 (±5) minutes prior to meals except for Days 5, 10 and 15 morning doses when no meals are offered following the study drug dose. Subjects are to resume their regular medication on Day 16.

[7]SAE collection starts after signing ICF.

[8]PK samples collected [redacted] on Days 5, 10 and 15. Blood collection time windows: ±5 minutes from 0-4 hours post-dose and ±10 minutes ≥6 hours post-dose.

## 5.2. Number of Subjects

This study will plan to enroll up to 15 eligible subjects. Subjects may be enrolled singly or in groups. In order to have at least 12 evaluable subjects complete the study, additional subjects may be enrolled to replace subjects who discontinue study participation prematurely. Evaluable subjects are defined as subjects included in the DDI population as described in Section 12.2.

## 5.3. Treatment Assignment

All subjects will receive the same treatment assignment as follows:

**Table 5-2: Treatment Assignment and PK Sample Collection**

| Day | Sitagliptin | Dorzagliatin | PK Sample Collection |
|---|---|---|---|
| 1-5 | 100 mg (QD) for 5 days | - | Day 5:<br>Sitagliptin alone |
| 6-10 | 100 mg (QD) for 5 days | 75 mg (BID) for 4 days<br>Morning dose only on Day 10 | Day 10:<br>Sitagliptin + Dorzagliatin |
| 11-15 | - | 75 mg (BID) for 4 days<br>Morning dose only on Day 15 | Day 15:<br>Dorzagliatin alone |

## 5.4. Criteria for Study Termination

The Investigator has the right to terminate the study in the interest of subject safety and welfare in consultation with sponsor. Additionally, the sponsor reserves the right to terminate or amend the study at any time for administrative reasons or if continuation of the protocol would present a potential safety risk to the subjects.

# 6. SELECTION AND WITHDRAWAL OF SUBJECTS

## 6.1. Subject Inclusion Criteria

To participate in the study, subjects must meet all the following eligibility criteria:

1. Subjects diagnosed with T2DM at least 3 months prior to screening and are currently taking a stable dose of either of the following therapy with no change in the dose for at least 4 weeks prior to screening:
   a. ≥1000 mg per day of metformin

b. a dipeptidyl peptidase 4 (DPP-4) inhibitor
c. a sodium-glucose cotransporter-2 (SGLT2) inhibitor
d. metformin plus a DPP-4 inhibitor
e. metformin plus a SGLT2 inhibitor

2. Subjects must agree to change their current therapy to 100 mg sitagliptin QD for at least 14 days prior to dosing on Day 1;
3. In stable general health as determined by the Investigator at screening evaluation performed no more than 42 days prior to dosing on Day 1;
4. Male and/or female subjects between the ages of 30 and 65 years, inclusive;
5. Are capable of giving informed consent and complying with study procedures;
6. Body Mass Index (BMI) of 19 to 38 kg/$m^2$, inclusive, at screening;
7. Fasting C-peptide test result >0.3 nmol/L (>0.90 ng/mL);
8. HbA1c ≥7% and ≤10.5%;
9. Females must be using one of the following birth control methods: a non-hormonal intrauterine device (IUD) in place for at least 3 months; barrier method (condom, diaphragm) with spermicide for at least 14 days prior to dosing on Day 1 and throughout the study; surgical sterilization of the partner (vasectomy for 6 months minimum); OR are surgically sterile OR postmenopausal, as defined below:
   a. Surgically sterile for at least 3 months prior to screening by one of the following means:
      - Bilateral tubal ligation
      - Salpingectomy (with or without oophorectomy)
      - Surgical hysterectomy
      - Bilateral oophorectomy (with or without hysterectomy)
   b. Postmenopausal, defined as following:
      - Last menstrual period greater than 12 months prior to screening, confirmed by FSH and estradiol levels at screening;
10. Nonsmoker, defined as not having smoked or used any form of tobacco for more than 6 months prior to screening;
11. Willing and able to adhere to study restrictions and to be confined at the clinical research center.

## 6.2. Subject Exclusion Criteria

Subjects will be excluded from study entry if any of the following exclusion criteria are present:

1. Fasting blood glucose at screening or Day -1 ≤110 or ≥270 mg/dL (one repeat screening fasting blood glucose allowed);
2. Type 1 diabetes mellitus, or latent autoimmune diabetes in adults; diabetic neuropathy, retinopathy or nephropathy;
3. Reported incidence of severe hypoglycemia, defined by denoting severe cognitive impairment requiring external assistance for recovery, within 3 months prior to screening and/or within 3 months prior to dosing on Day 1;
4. Known contraindications to sitagliptin;
5. Clinically significant gastrointestinal disorder (e.g., gastroparesis, constipation, diarrhea, colitis, gastrointestinal tract obstruction, hiatal hernia with reflux esophagitis, or cholestasis);
6. History or symptoms of clinically significant cardiovascular disease, particularly coronary artery disease, arrhythmias, atrial tachycardia, or congestive heart disease within one year prior to screening;
7. History of more than three urinary tract infections and/or more than three genital fungal infections in the last 12 months. If one or more genital fungal infections in the last 12 months required systemic treatment to resolve, Investigator will determine eligibility based on treatment required;
8. Reported history of clinically significant central nervous system disease including: transient ischemic attack, stroke, seizure disorder, depression, or behavioral disturbances within one year prior to screening;
9. Reported history of liver disease (e.g., hepatitis, cirrhosis within one year of screening and/or liver enzymes (alanine aminotransferase (ALT), aspartate aminotransferase (AST), gamma-glutamyltransferase (GGT), total bilirubin (TBiL)) >2 times the upper limit of normal (ULN));
10. Reported history of clinically significant renal disease;
11. Estimated glomerular filtration rate (eGFR) ≤60 mL/min/1.73m$^2$;
12. Acute or chronic metabolic acidosis, including diabetic ketoacidosis;
13. Known or suspected malignancy;

14. Any reported hypersensitivity (anaphylaxis and angioedema) or intolerance to sitagliptin;
15. Antidiabetic treatment with insulin, sulfonylureas, thiazolidinediones or GLP-1 agonist within 3 months prior to screening;
16. Systolic blood pressure <90 or ≥160 mmHg or diastolic blood pressure <60 or ≥100 mmHg at screening (may be repeated once);
17. A hospital admission or major surgery within 90 days prior to screening;
18. Uncontrolled hypertriglyceridemia >500 mg/dL;
19. Positive blood screen for human immunodeficiency virus (HIV), hepatitis B surface antigen (HBsAg), or hepatitis C antibody;
20. Positive pregnancy test result;
21. Female is breast-feeding or planning to become pregnant;
22. Treated with any investigational drugs within 6 weeks prior to screening;
23. Reported history of prescription drug abuse, or illicit drug use within 6 months prior to screening;
24. Reported history of alcohol abuse according to medical history within 6 months prior to screening;
25. A positive screen for alcohol or drugs of abuse;
26. Reported history of donation or blood collection of more than 1 unit (approximate 450 mL) of blood (or blood products) or acute loss of blood during the 90 days prior to screening;
27. Use of prescription or over-the-counter (OTC) medications, and herbal (including St John’s Wort, herbal teas, garlic extracts) within 14 days prior to dosing (except for sitagliptin dosing during run-in period). No angiotensin-converting enzyme (ACE) inhibitors are allowed; (Note: Use of acetaminophen at <1 g/day is permitted until 24 hours prior to dosing);
28. An unwillingness of male participants to use appropriate contraceptive measures if engaging in sexual intercourse with a female partner of childbearing potential. Appropriate measures include use of a condom and spermicide and, for female partners, use of an IUD, diaphragm with spermicide, oral contraceptives, injectable progesterone, progesterone subdermal implants, or a tubal ligation. Sexual intercourse with pregnant or lactating women is prohibited.

## 6.3. Prohibitions and Restrictions

Subjects must be willing to adhere to the following prohibitions and restrictions from times noted and for the duration of the study:

- No illicit drug use, abuse of alcohol and use of tobacco-containing products within 6 months prior to screening;
- No consumption of alcohol or food containing alcohol;
- No consumption of food or drinks containing caffeine;
- No consumption of grapefruit juices;
- No administration of any prescription medications (with the exception of study drug) per Exclusion Criteria #27;
- No use of any OTC products (with the exception of acetaminophen <1 g/day until 24 hours prior to dosing) per Exclusion Criteria #27;
- Subjects must remain upright for 4 hours following administration of study drug (except during measurement of vital signs and ECGs) following the morning dose on Days 5, 10 and 15.

## 6.4. Subject Withdraw Criteria

Subjects will be informed that they have the right to withdraw from the study at any time for any reason without prejudice to their medical care. The Investigator also has the right to withdraw subjects from the study if it is in the best interest of the subject, or if the subject experiences an adverse event that warrants premature withdrawal.

Subjects may be withdrawn from the study if they:

- Have entered the study in violation of the protocol
- Use or require the use of prohibited concomitant medication(s)
- Are non-compliant with study procedures
- Experience an AE that warrants premature withdrawal

Subjects who experience emesis after dosing are not required to withdraw from the study. The event and time of emesis should be documented, and the subject permitted to continue at the discretion of the Investigator.

All treated subjects should be followed according to the Schedule of Procedures (Table 5-1). All subjects who receive a Day 1 dose, even those who have discontinued prematurely, should have all evaluations for the Day 17 End-of-Study visit performed, if possible. All procedures should be documented in the electronic case report form (eCRF). For all subjects who withdraw prematurely, the Investigator will indicate one of the following reasons for withdrawal in the eCRF:

- Adverse event
- Death
- Protocol violation
- Lost to follow-up
- Subject’s decision
- Other (reason to be specified by the Investigator)

In case of subjects’ discontinuation from the study due to an AE, such subjects will be closely monitored until the resolution or stabilization of the AE. The Investigator should document the reason for discontinuation in the source documentation and eCRF.

In the event that a subject withdraws participation from the study early, early withdrawal should be documented by the Investigator (or designee) in the appropriate eCRF pages and source documents when confirmed. The Day 17 End-of-Study assessments should be performed when a subject is discontinued.

# 7. STUDY PROCEDURES

Morning dosing will occur at approximately 08:00 a.m. and the evening dosing will occur at approximately 06:00 p.m. All doses will be administered 60 (±5) minutes prior to meals (except for on Days 5, 10 and 15 when meals are not offered after the morning dose) with about 240 mL (8 fluid ounces) of room temperature water. Actual times for each dose and start times for meals as well as oral glucose intake on days of dosing will be recorded. Standardized meals that are consistent with general dietary recommendations for diabetes will be provided while subjects are staying at CRC.

Eligible subjects will have a minimum 12-day run-in period prior to admission to the CRC on Day -2, during which time they will self-administer sitagliptin 100 mg QD each morning. Subjects will complete a diary to record sitagliptin doses taken each day and will be advised to monitor their blood glucose levels and report changes in health status to the CRC. A telephone call will be placed to all subjects at approximately Day 6 (±2 day) of the run-in period to assess general health and collect AE information. Subjects will be admitted to the CRC on Day -2 and remain sequestered at the study site until after all End-of-Study procedures are completed on Day 17 or at early termination.

The following sections describe in detail all study procedures. The schedule of procedures is presented in Table 5-1.

## 7.1. Screening Visit and Run-in Period

Subjects will report to the CRC for a screening outpatient visit between Day -42 and Day -15 relative to the Day 1 dosing day.

As outlined in Section 15.3, prior to the performance of any study-related activities or evaluations, the subject must be thoroughly informed about all aspects of the study, including scheduled visits and activities. Each subject will sign the study-specific consent form prior to any screening procedures. A signed copy of the informed consent form (ICF) will be given to each consenting subject and another signed copy will be retained in the subject's study records.

The following information and procedures will be performed and documented as part of the screening assessment:

- Collection of demographic information, including sex, race, ethnic origin, date of birth.
- Medical history, including review of prior and ongoing medications taken in previous 30 days, except for therapy to treat diabetes, which must be recorded for previous 3 months.
- Height and weight measurement and BMI calculation.
- Urine sample collection for urinalysis and test for drugs of abuse.
- Urine sample pregnancy test for women.
- Blood test for evaluation of FSH and estradiol for postmenopausal women.
- Saliva sample collection to test use of alcohol.
- Standard 12-lead ECG after resting supine for at least 5 minutes.

- Vital signs (BP, PR, RR and oral T) measurement after resting supine for at least 5 minutes.
- Blood sample collection for clinical chemistry, hematology, HIV, and hepatitis B and C evaluations, C-peptide measurement and HbA1c test.
- Physical examination.
- Assessment of eligibility according to inclusion/exclusion criteria.

Compliance with inclusion criteria (listed in Section 6.1) and exclusion criteria (listed in Section 6.2) will be verified against information collected and documented in the source documents and the eCRF. Laboratory results obtained at screening and on Day -1 will be used to verify eligibility.

Eligible subjects will be scheduled for a visit to the CRC for dispensing of sitagliptin 100 mg and start their 12-day run-in period. Subjects will be instructed to take a single dose of sitagliptin each morning, and to record each dose in a diary that will be provided by the CRC. Subjects will be advised to monitor their blood glucose levels and report changes in health status to the CRC. A telephone call will be placed to all subjects at approximately Day 6 (±2 day) of the run-in period to assess general health and collect AE information. Any signs or symptoms reported by subjects that develop or worsen compared to the subject's medical history after the first dose of sitagliptin in the run-in period will be recorded as AEs and will be followed by the Investigator. Additionally, subjects will be advised to contact their primary care physician.

An appointment will be scheduled for subjects to be admitted to the CRC on Day -2. Subjects will be advised to take their dose of sitagliptin at home prior to admission, and to bring remaining drug and their diary with them to the CRC on Day -2.

## 7.2. Domiciled at CRC on Day -2 through Day 17

### 7.2.1. Study Check-in Visit: Day -2 and Day -1

Subjects will be admitted to the CRC on Day -2 and remain for 18 overnight stays until completion of all study procedures on Day 17 or early termination.

The following assessments will be performed on Day -2:

- Collection of remaining sitagliptin and review of diary to confirm compliance with daily dosing of sitagliptin during the run-in period. Subjects with <90% compliance or compliance recorded as >110% are not eligible to continue study participation.
- Update of medical history, assessment of AEs, and medication use since the screening visit.
- Urine sample testing for drugs of abuse and pregnancy, as applicable.
- Alcohol saliva test.
- Assess blood glucose using a glucometer within 60 minutes before each meal and after study drug dosing when applicable.

The following assessments will be performed on Day -1:

- Blood and urine sample collection for clinical chemistry, hematology and urinalysis in the morning under fasting condition (after at least a 10-hour fast).
- Physical examination.
- Vital signs (BP, PR, RR and oral T) measurement after resting supine for at least 5 minutes.
- Measure weight
- Standard 12-lead ECG after resting supine for at least 5 minutes.
- Subjects will be administered sitagliptin 100 mg by the clinic staff in the morning at approximately 08:00 a.m. prior to a standardized meal.
- Assess blood glucose using a glucometer within 60 minutes before each meal and after study drug dosing when applicable.
- Confirm fasting blood glucose on Day -1 >110 and <270 mg/dL.
- Confirmation of eligibility according to inclusion/exclusion criteria.
- Assessment of AEs and concomitant medications.

### 7.2.2. Day 1 - Day 4

- Vital signs (BP, PR, RR and oral T) measurement after resting supine for at least 5 minutes within 60 minutes before each study drug dose.
- Administer 100 mg sitagliptin QD at 08:00 a.m. (±5 minutes), 60 (±5) minutes prior to a standardized meal.

- Assess blood glucose using a glucometer within 60 minutes before each meal and after study drug dosing when applicable.
- Assessment of AEs and concomitant medications.

### 7.2.3. Day 5

- Blood and urine sample collection for clinical chemistry, hematology and urinalysis in the morning under fasting condition (after at least a 10-hour fast) and before study drug dose.
- Vital signs (BP, PR, RR and oral T) measurement after resting supine for at least 5 minutes within 60 minutes before study drug dose.
- Administer 100 mg sitagliptin at 08:00 a.m. (±5 minutes). Subjects must remain upright for at least 4 hours following dosing except for measurement of vital signs or ECGs.
- Pharmacokinetic blood sample collections for the measurement of sitagliptin concentrations [redacted].
- Consume a 75-gram glucose solution 30 (±5) minutes following study drug dose in lieu of a breakfast.
- Pharmacodynamic blood sample collections to measure GLP-1 and glucagon in plasma, and glucose, insulin, and C-peptide in serum at baseline (prior to study drug dose and within 60 minutes prior to oral glucose intake) and 0.5, 1, 1.5, 2, 2.5, 3 and 4 hours after oral glucose intake.
- Assess blood glucose using a glucometer within 60 minutes before each meal and after study drug dosing when applicable.
- Standard 12-lead ECG after resting supine for at least 5 minutes, taken 2 hours (±15 minutes) after study drug dose.
- Assessment of AEs and concomitant medications.

### 7.2.4. Day 6 - Day 9

- Pharmacokinetic blood sample collections for the measurement of sitagliptin concentrations 18 and 24 hours post-dose (Day 6).

- Vital signs (BP, PR, RR and oral T) measurement after resting supine for at least 5 minutes within 60 minutes before each study drug dose.
- Administer 100 mg sitagliptin QD at 08:00 a.m. (±5 minutes) and 75 mg dorzagliatin BID (08:00 a.m., 06:00 p.m. (±5 minutes), each dose 60 (±5) minutes prior to a standardized meal.
- Assess blood glucose using a glucometer within 60 minutes before each meal and after study drug dosing when applicable.
- Assessment of AEs and concomitant medications.

### 7.2.5. Day 10

- Blood and urine sample collection for clinical chemistry, hematology and urinalysis in the morning under fasting condition (after at least a 10-hour fast) and before study drug dose.
- Vital signs (BP, PR, RR and oral T) measurement after resting supine for at least 5 minutes within 60 minutes before each study drug dose.
- Administer 100 mg sitagliptin and 75 mg dorzagliatin at 08:00 a.m. (±5 minutes). Subjects must remain upright for at least 4 hours following dosing except for measurement of vital signs or ECGs.
- Pharmacokinetic blood sample collections for the measurement of sitagliptin and dorzagliatin concentrations [redacted].
- Consume a 75-gram glucose solution 30 (±5) minutes following study drug dose in lieu of a breakfast.
- Pharmacodynamic blood sample collections to measure GLP-1 and glucagon in plasma, and glucose, insulin, and C-peptide in serum at baseline (prior to study drug dose and within 60 minutes prior to oral glucose intake) and 0.5, 1, 1.5, 2, 2.5, 3 and 4 hours after oral glucose intake.
- Assess blood glucose using a glucometer within 60 minutes before each meal and after study drug dosing when applicable.
- Standard 12-lead ECG after resting supine for at least 5 minutes, taken 2 hours (±15 minutes) after study drug dose.

- Assessment of AEs and concomitant medications.

### 7.2.6. Day 11 - Day 14

- Pharmacokinetic blood sample collections for the measurement of sitagliptin and dorzagliatin concentrations 18 and 24 hours post-dose (Day 11).
- Vital signs (BP, PR, RR and oral T) measurement after resting supine for at least 5 minutes within 60 minutes before each study drug dose.
- Administer 75 mg dorzagliatin BID (08:00 a.m., 06:00 p.m. (±5 minutes) 60 (±5) minutes prior to a standardized meal.
- Assess blood glucose using a glucometer within 60 minutes before each meal and after study drug dosing when applicable.
- Assessment of AEs and concomitant medications.

### 7.2.7. Day 15

- Blood and urine sample collection for clinical chemistry, hematology and urinalysis in the morning under fasting condition (after at least a 10-hour fast) and before each study drug dose.
- Vital signs (BP, PR, RR and oral T) measurement after resting supine for at least 5 minutes within 60 minutes before each study drug dose.
- Administer 75 mg dorzagliatin at 08:00 a.m. (±5 minutes). Subjects must remain upright for at least 4 hours following dosing except for measurement of vital signs or ECGs.
- Pharmacokinetic blood sample collections for the measurement of dorzagliatin concentrations [redacted].
- Consume a 75-gram glucose solution 30 (±5) minutes following study drug dose in lieu of a breakfast.
- Pharmacodynamic blood sample collections to measure GLP-1 and glucagon in plasma, and glucose, insulin, and C-peptide in serum at baseline (prior to study drug dose and within 60 minutes prior to oral glucose intake) and 0.5, 1, 1.5, 2, 2.5, 3 and 4 hours after oral glucose intake.

- Assess blood glucose using a glucometer within 60 minutes before each meal and after study drug dosing when applicable.
- Standard 12-lead ECG after resting supine for at least 5 minutes, taken 2 hours (±15 minutes) after study drug dose.
- Assessment of AEs and concomitant medications.

#### 7.2.8. Day 16

- Pharmacokinetic blood sample collections for the measurement of dorzagliatin concentrations 18 and 24 hours post-dose.
- Assess blood glucose using a glucometer within 60 minutes before each meal.
- Subjects will resume their regular medication schedule.
- Assessment of AEs and concomitant medications.

#### 7.2.9. Day17: End-of-Study Procedures

The following procedures will be performed at the End-of-Study or Termination Visit:

- Blood and urine sample collection for clinical chemistry, hematology and urinalysis in the morning under fasting condition (after at least a 10-hour fast).
- Urine pregnancy test for women.
- Physical examination.
- Measure weight.
- Vital signs (BP, PR, RR and oral T) measurement after resting supine for at least 5 minutes.
- Standard 12-lead ECG after resting supine for at least 5 minutes.
- Assessment of AEs and concomitant medications.
- Subjects will be offered breakfast and may be discharged from the CRC upon completion of all study procedures.

### 7.3. Early Withdrawal Visit

As outlined in Section 6.4, in the event that a subject discontinues study participation after Day 1 dose administration but prior to the final End-of-Study visit, if possible, the subject should complete all End-of Study assessments prior to being discharged from the CRC, as described in

Section 7.2.9 for Day 17. The reason for discontinuation must be fully documented in the subject's source documentation and in the eCRF.

### 7.4. Time Windows for Procedures

Any procedure performed outside the stated time windows below will be recorded as a protocol deviation.

- Sitagliptin and dorzagliatin administered by clinic staff will be taken 60 (±5) minutes prior to meals.
- Standard resting 12-lead ECGs will be performed 2 hours (±15 minutes) after the study drug dose, after the subject has rested in the supine position for at least 5 minutes.
- The oral glucose solution will be administered 30 (±5) minutes after study drug dose on Days 5, 10 and 15.
- Blood samples for PK analyses will be collected ±5 minutes from 0-4 hours post-dose and ±10 minutes ≥6 hours post-dose.
- Blood samples for PD analyses will be collected ±5 minutes from 0-4 hours after glucose intake.
- Meal start times will be within ±10 minutes of scheduled start time.

## 8. TREATMENT OF SUBJECTS

### 8.1. Description of Study Drugs

Dorzagliatin (HMS5552) is an investigational novel GKA, indicated as an adjunct to diet and exercise to improve glycemic control in adults with T2DM.

The anticipated dosing regimen is twice daily as oral administration. The proposed dosage form for this study is a film-coated tablet with 75 mg dorzagliatin. [redacted]

[redacted]

[redacted]

Sitagliptin ([redacted] 100 mg tablets for oral administration) will be supplied by Hua Medicine.

## 8.2. Methods of Assigning Subjects to Dose Groups

There is no randomization since this is an open label, single sequence study. Subjects may be enrolled singly or in groups.

## 8.3. Blinding

This is an open label study. There will be no blinding for this study.

## 8.4. Treatment Discontinuation/Subject Withdrawal

Subjects may withdraw from the study at any time at their own request, or they may be withdrawn at any time at the discretion of the Investigator or sponsor for safety, behavioral, or administrative reasons. If a subject discontinues study participation at any time prior to the final End-of-Study assessments, if possible, the subject should complete all End-of Study assessments. The reason for discontinuation must be fully documented in the subject's source documentation and in the eCRF.

## 8.5. Prior and Concomitant Medications and Substances

All prescription medications and OTC products, including herbal products, taken within 30 days prior to screening and during the study period will be documented in the subject's source documentation and the eCRF. Therapy to treat T2DM taken within 3 months prior to screening will also be documented in the subject's source documentation and the eCRF. The documented use of prescription sitagliptin during the run-in period is required.

## 8.6. Meals

Standardized meals based on the recommended American Diabetes Association diet, will be offered to all subjects. An evening snack (no later than 09:00 p.m.) will be offered on the evening of admission. Subjects will be domiciled and dosed, and have meals provided by the CRC. Standard meals will be served at approximately 60 minutes after dosing and shall be completed within 30 minutes when meals are administered after a study drug dose. While domiciled at the CRC, meals will be offered at approximately 09:00 a.m., 12:30 p.m. and

07:00 p.m., and a light evening snack will be offered at approximately 9:00 p.m. or earlier. Meal start times will be within ±10 minutes when the meal time coincides with a scheduled PK or PD blood draw. Soft drinks (sodas) without caffeine or sugar, or non-grapefruit juices without added sugar will be offered with meals and ad libitum beginning 4 hours post-dose.

Water intake will be restricted from 1 hour prior to dosing until after the 1-hour post-dose PK blood sample is collected (with the exception of 240 mL of water given with the dosing) on study Days 5, 10 and 15.

# 9. STUDY DRUG MATERIALS AND MANAGEMENT

## 9.1. Study Drug Packaging and Labeling

Dorzagliatin drug products are supplied as 75 mg strength tablets. The drug product tablets are packaged in Alu-Pla Blister as in total 8 tablets per plate.

Product will be labeled according to applicable regulatory requirements. The label will include at least the following information:

- Name and address of manufacturer
- Protocol number or other identifier to reference the study
- Name of the sponsor, product name and batch number
- Place to record subject number and initials
- Storage conditions

The CRC pharmacist or designee will prepare the study drug and maintain the drug packaging and labeling log.

Sitagliptin ([redacted] 100 mg tablets for oral administration) will be supplied by Hua Medicine.

## 9.2. Study Drug Storage and Accountability

All study drug supplies will be stored in a secure, locked area, under the responsibility of the Investigator or other authorized individual.

Dorzagliatin must be stored under controlled room temperature (10-30°C or 50-86°F) in tightly closed containers protected from light. Sitagliptin must be stored at 20-25°C (68-77°F) with

excursions permitted to 15-30°C (59-86°F). The temperature and humidity of the stored room should be monitored and recorded.

The Investigator or designee must maintain accurate records of the receipt of all study drug, including date received, lot number, expiration date if applicable, amount received, condition of the package and the disposition of all study drug.

Current dispensing records will also be maintained including the date and amount of study drug dispensed and the identity of the subject receiving the study drug.

## 9.3. Study Drug Preparation

Sitagliptin 100 mg tablets will be dispensed to subjects for at-home dosing during the 12-day run-in period. Study drug reconciliation will be performed at the end of the run-in period by tablet count.

Dorzagliatin 75 mg and sitagliptin 100 mg tablets will be dispensed to subjects while domiciled at the CRC according the study treatment scheme. Morning and evening doses of study drug will be administered by CRC staff with approximately 240 mL (8 fluid ounces) room temperature water at approximately 08:00 a.m. and 06:00 p.m., 60 (±5) minutes prior to a meal, except for Days 5, 10 and 15 when there will be no meal following the morning dose.

## 9.4. Study Drug Handling and Disposal

All unused study drug and supplies must be returned to sponsor or disposed according to sponsor’s instruction after the study is completed and the drug accountability log is reconciled.

# 10. PHARMACOKINETIC/PHARMACODYNAMIC ASSESSMENTS

## 10.1. Pharmacokinetic Sample Collection and Storage

A total of 42 blood samples (approximately 6 mL each) will be collected from each subject into $K_2EDTA$ tubes for determination of dorzagliatin and sitagliptin concentration and PK assessments. Samples will be collected [redacted] on Days 5, 10 and 15 using an indwelling catheter or direct venipuncture.

When PK blood collection times coincide with other procedures, the blood sample should be collected first, using the following post-dose windows: ±5 minutes from 0-4 hours post-dose and ±10 minutes $\geq$6 hours post-dose.

Contents of tube will be mixed thoroughly with gentle inversion at least 8 times to mix the anti-coagulant and stored on ice for no more than 30 minutes before centrifugation at approximately 4 (±1)°C at ~2000 g for 10 minutes. The harvested plasma will be split into two approximately equal aliquots and stored in 2 mL or appropriate size cryovial tubes.

Plasma samples will be stored at -70 (±10)°C within approximately 60 minutes of harvesting pending shipment to the bioanalytical laboratory for analysis. Full instructions for the collection, storage, and shipment of these samples will be provided in a separate PK Laboratory Manual.

## 10.2. Pharmacokinetic Specimen Labeling

Labels will be affixed to the cryovials in a manner that will prevent the label from being detached after being wet or freezing. The tube labels will contain the subject number, treatment, nominal day, and nominal collection time, as appropriate.

The site will provide a sample inventory page, listing the information above for each sample. The sample inventory page will also include the treatment cohort for each series of tubes.

## 10.3. Pharmacokinetic Sample Shipping Instructions

All PK plasma samples will be kept frozen and shipped on dry ice by the same day or overnight courier to:

Frontage Laboratories, Inc.
Sample Coordinator
[redacted]

Plasma samples will be shipped in two separate shipments. The first shipment of samples (the primary aliquot of each sample) will be shipped after subjects have completed the study. The second aliquot of each sample will be shipped after notification from the laboratory of receipt of primary samples.

## 10.4. Bioanalytical Methodology

The PK plasma samples will be analyzed using validated, specific and sensitive methods of liquid chromatographic separation with tandem mass spectrometric (LC-MS/MS) detection for concentrations of dorzagliatin and sitagliptin by the designated bioanalytical lab.

## 10.5. Pharmacokinetic Parameters

Dorzagliatin and sitagliptin PK parameters ($C_{max}$, $T_{max}$ and $AUC_{0-24h}$) will be computed for each subject using non-compartmental methods by Phoenix WinNonlin version 8.1 (Certara, Princeton, NJ USA) based on the actual sampling times. Additional PK parameters ($T_{1/2}$, $K_{el}$, $V_z/F$, CL/F) may be calculated if needed. Descriptive statistics will be calculated for the PK parameters for Days 5, 10 and 15. A description of PK terms is provided below.

**Table 10-1: PK Terms**

| PK Term | Description |
|---|---|
| $C_{max}$ | Observed maximum plasma concentration |
| $T_{max}$ | Time at which $C_{max}$ was first observed |
| $AUC_{0-24h}$ | Area under the concentration-time curve from 0 to 24 hours |
| $T_{1/2}$ | Terminal elimination half-life |
| $K_{el}$ | Elimination rate constant |
| $V_z/F$ | Apparent volume of distribution during terminal phase after oral administration |
| CL/F | Apparent total plasma clearance of drug after oral administration |

## 10.6. Pharmacodynamic Assessments

Subjects will consume a 75-gram glucose solution 30 (±5) minutes following study drug administration on Days 5, 10 and 15, in lieu of a breakfast. The glucose solution will be consumed within a 5-minute timeframe. A total of 48 blood samples (24 samples of approximately 8.5 mL each for measurement of plasma GLP-1 and glucagon, and 24 samples of approximately 7 mL each for measurement of serum glucose, insulin, and C-peptide will be collected from each subject for PD assessments. Samples will be collected at baseline (prior to the study drug dose and within 60 minutes prior to oral glucose intake) and 0.5, 1, 1.5, 2, 2.5, 3 and 4 hours after oral glucose intake on Days 5, 10 and 15.

When PD blood collection times coincide with other procedures, the blood sample should be collected first within the ±5 minutes time window.

For measurement of plasma GLP-1 and glucagon, approximately 8.5 mL blood will be collected into Becton Dickenson BD P800 tubes that contain spray-dried $K_2$EDTA anticoagulant and other proprietary additives. Contents will be gently inverted 8-10 times and centrifuged within 60 minutes of collection at 1100-1300 g for 20 minutes at 18-25°C. Three aliquots of plasma will be harvested as 1.0 mL for measurement of glucagon and active GLP-1, 1.0 mL for measurement of total GLP-1 and 1.5 mL as a back-up sample. All samples will be stored immediately at ≤70°C until shipment to the Bioanalytical laboratory. Samples will be shipped on dry ice to:

[redacted]
Mercodia AB
[redacted]

For measurement of serum glucose, insulin and C-Peptide, the sample collection tubes and processing instructions will be provided by BioReference Laboratories, the facility that will measure all safety laboratory assessments.

Full instructions for the collection, storage, and shipment of PD samples will be provided in a separate PD Laboratory Manual.

# 11. ASSESSMENT OF SAFETY

Safety assessments will include monitoring of AEs, blood glucose via glucometer readings, vital signs, clinical laboratory findings, resting 12-lead ECGs and PE findings.

## 11.1. Safety Parameters

### 11.1.1. Demographic/Medical History

Demographic characteristics (age, sex, race and ethnicity) will be collected at the screening visit. Medical history will be reviewed and collected at the screening and on Day -2.

### 11.1.2. Vital Signs

Vital signs, including BP, PR, RR, and oral T, will be measured at screening, on Day -1, on Days 1-15 within 60 minutes prior to each morning and evening dose, and on Day 17 or at the end of the study. Vital signs will be measured after resting supine for at least 5 minutes.

Vital signs may be repeated for clinically significant abnormal vital signs at the discretion of the Investigator.

As a guideline, vital signs outside the ranges shown below may be considered abnormal, at the discretion of the Investigator:

SBP: 95-160 mmHg

DBP: 55-100 mmHg

PR: 45-100 bpm

RR: 10-20 bpm

T: 36.0-37.2°C

### 11.1.3. Physical Examination, Height, Weight, and Body Mass Index

Full PEs will be performed by qualified personnel at the screening visit, Day -1, Day 17 and the End-of-Study visit. At the screening visit, height (centimeters) and weight (kilograms) will be measured and BMI will be calculated. Weight will also be measured on Day -1 and Day 17. All abnormal findings will be documented in the source documentation and in the eCRF.

### 11.1.4. Electrocardiogram

Standard resting 12-lead ECGs will be performed at the screening visit, Day -1, 2 hours (±15 minutes) post-dose on Days 5, 10 and 15, and on Day 17 at the End-of-Study visit. All ECGs will be performed after the subject has rested supine for at least 5 minutes.

Post-dose ECGs must be evaluated for safety by the Investigator or his/her designee. The final conduction intervals entered into the eCRF will be those generated by the ECG machine, unless deemed significantly inaccurate by the review performed by the Investigator or designee. In these cases, the over-read intervals will be documented in the eCRF. The Investigator will also record an overall assessment of the ECG.

Clinically significant abnormal ECGs may be repeated at the discretion of the Investigator.

As a guideline, ECG parameters outside the ranges shown below may be considered abnormal, at the discretion of the Investigator:

VR: 45-100 bpm

PR: 120-210 msec

QRS: ≤120 msec

QT: <500 msec

QTc: <450 msec

### 11.1.5. Clinical Laboratory Assessments

Hematology, blood chemistry, and urinalysis evaluations will be performed at the screening visit, on Day -1, Days 5, 10, and 15, and Day 17 (End-of-Study Visit). Blood and urine samples will be collected after an overnight fast of at least 10 hours, and within 30 minutes prior to the morning dose of study drug on Days 5, 10 and 15. The list of clinical laboratory assessments is included in Appendix A.

C-peptide and HbA1c testing will be performed at the screening visit to determine eligibility.

Blood glucose will be measured using a glucometer three times each day on Days -2 through Day 16, within 60 minutes before each meal and after study drug dosing when applicable.

The results of clinical laboratory tests conducted at the screening visit and on Day -1 must be assessed by the Investigator to determine each subject's eligibility for participation in the study. The Investigator should signify review of the laboratory reports by signing and dating the report. If subjects' values are out of range and the Investigator deems the out of range value is clinically significant, the Investigator must discuss and agree upon individual cases with sponsor's Medical Monitor prior to enrollment.

Any significant abnormalities should be fully investigated. Whenever possible, the etiology of the abnormal findings will be documented on the eCRF. Laboratory results with clinically significant abnormal values may be repeated for verification. Additional tests and other evaluations required to establish the significance or etiology of an abnormal result or to monitor the course of an AE should be obtained when clinically indicated. In particular, if a clinically significant abnormal result is observed that is not resolved by the final study visit, repeat tests

will be performed if possible and resolution or stability of the abnormality will be recorded in the source documentation.

Any clinically significant laboratory abnormalities that are either serious (e.g., results in hospital admission) or unexpected will be promptly reported to the representative of sponsor. Any additional relevant laboratory results obtained by the Investigator during the course of this study will be reported to sponsor.

Virus serology (HIV, and hepatitis B and C) will be assessed at the screening visit and must be negative to qualify enrollment.

Urine drug screens and saliva alcohol tests will be conducted at the screening visit and on Day -2. Results must be negative to qualify for dosing on Day 1.

Urine pregnancy test for all female subjects will be performed at the screening visit, on Day -2 and on Day 17 (End-of-Study). Results at screening and Day -2 must be negative to qualify for dosing on Day 1. Any positive pregnancy test results on Day 17 will be reported to the sponsor within 24 hours of awareness of the pregnancy using a Pregnancy Report provided by Frontage.

Postmenopausal status of females will be confirmed by serum FSH and estradiol levels at screening.

## 11.2. Adverse Events

Adverse events will be recorded after the first dose of sitagliptin in the run-in period. Subjects will be instructed to report AEs during the study and staff will query subjects regarding AEs throughout the study. The Investigator or designee must document all AEs reported by the subject after the first dose of sitagliptin in the run-in period through completion of the End-of-Study visit. Any subject who is withdrawn from the study due to an AE shall be followed until the event has resolved or stabilized, and the Investigator will document available follow-up information on the subject’s source documentation and eCRF.

### 11.2.1. Definitions of Adverse Events

Adverse event means any untoward medical occurrence in a patient or clinical investigation subject administered a pharmaceutical product and which does not necessarily have to have a causal relationship with this treatment. An AE can therefore be any unfavorable and unintended

sign, symptom, or disease temporally associated with the use of medicinal product, whether or not related to the medicinal product. An AE is any symptom, sign, illness, or experience that develops or worsens in severity and/or frequency during the course of the study. Adverse events reported after administration of the first dose of study drugs (Day 1) will be considered treatment-emergent. Adverse events will be considered treatment-emergent if not present prior to the initiation of the treatment with study drug on Day 1 or already present but worsens in either severity or frequency following exposure to the treatment.

Adverse events include serious and non-serious AEs. A Serious Adverse Event (SAE) is defined as an AE occurring during any clinical study period that meets one or more following criteria:

- Results in death
- Is life threatening
- Requires inpatient hospitalization or prolongation of existing hospitalization
- Results in persistent or significant disability/incapacity
- Is a congenital anomaly/birth defect
- Is a medically important event that may jeopardize the subject or may require medical or surgical intervention to prevent one of the other outcomes listed above

The term "life threatening" in the definition of "serious" refers to an event in which the subject was at risk of death at the time of the event; it does not refer to an event that hypothetically might have caused death if it were more severe. Death is an outcome of an event. The event that resulted in death should be recorded in source documents, the eCRF and reported on the SAE form. All SAEs that occur after consent to the End-of-Study visit must be reported to sponsor using the SAE Reporting Form provided by Frontage (see Section 11.2.3).

Abnormal results of laboratory tests or diagnostic procedures (such as test results from hematology, blood biochemistry, urinalysis, ECG, physical examination, vital signs evaluations, etc.) are considered to be AEs if the abnormality is considered by the Investigator as clinically significant, or the clinical significance worsens compared to the subject's baseline.

Any of the following abnormal results are considered clinically significant:

- The abnormality suggests a disease and/or organ toxicity that is new or has worsened from baseline;

- The abnormality needs to adjust investigational product dosage and usage, e.g., drug discontinuation;
- The abnormality requires additional active intervention, for instance an increase or a modification of the concomitant medication, close observation, or further diagnostic investigation, etc.

If a clinically significant laboratory abnormality is a manifestation of disease, then only the diagnosis will be recorded as AE. If a clinically significant laboratory abnormality is not a manifestation of disease, then the abnormality itself is recorded as AE. An appropriate description is used to record test results that are either lower or higher than normal range. If the result meets diagnostic criteria, the clinical diagnosis is recorded as AE.

The severity of each AE will be graded by the Investigator according to the following criteria:

- Mild: awareness of sign or symptom, but easily tolerated.
- Moderate: discomfort sufficient to cause interference with normal activities.
- Severe: incapacitating, with inability to perform normal activities.

The Investigator must assess the relationship between the AE and the study drug by using the following definitions:

1. **Related**: The occurrence of AE follows a reasonable temporal sequence from administration of the study drug; adverse event is consistent with known characteristics of the study drug; the event is improved when the dose of study drug is decreased or stopped; the event re-occurs when the study drug treatment is re-started. It cannot be explained by the medical condition of the subject or alternative treatment.

2. **Possibly Related**: The occurrence of AE follows a reasonable temporal sequence from administration of the study drug; AE is consistent with a known characteristic of the study drug; reducing the dosage or stop the drug will cause the AE to be alleviated or no obvious change. The medical condition of the subject or alternative treatment may have contributed to the event.

3. **Unlikely Related**: The occurrence of AE, whose temporal sequence from administration of the study drug, is unclear. The AE may not be consistent with a known characteristic

of the study drug. The medical condition of the subject or alternative treatments may have contributed to the event.

4. **Not Related**: There is no reasonable temporal relationship between the AE occurrence and study drug administration; the AE is not consistent with known characteristic of the study drug; the medical condition of the subject or alternative treatments may have contributed to the event. The AE improves or disappears when disease condition improves, or alternative treatment is stopped.

The outcome of an AE can be described as:

- Recovered: the subject returns to baseline state.
- Recovering: the events haven't been resolved completely, but subject is improving.
- Not recovered: the events are ongoing, for example, irreversible congenital anomaly
- Recovered with sequelae: only if the subject will suffer from life-time sequelae, for example, the blindness caused by diabetes and the hemiplegia after a stroke.
- Fatal: the death date is the event end date.
- Unknown: Investigator can't obtain the outcomes of AEs, e.g., the subject is lost to follow-up.

If the AE outcome is assessed as "Recovered", "Recovered with sequelae", or "Fatal", the end date of the AE must be recorded.

### 11.2.2. Recording Adverse Events

All AEs (regardless of seriousness or relationship to study drug) are to be recorded in the subject's source documents and on the corresponding page(s) in the eCRF. Whenever possible, symptoms, signs, and laboratory abnormalities should be grouped as a single syndrome or diagnosis. The Investigator should specify the date of onset and stop date of event, severity and the time of severity worsening, action taken with respect to study drug, corrective treatment/therapy given, seriousness, outcome, hospitalization date (if applicable), discharge date (if applicable), cause of the death (if applicable), date of death (if applicable), autopsy (if applicable) and his/her opinion as to whether there is a reasonable possibility that the AE was caused by the study drug, according to the definitions noted in Section 11.2.1. All medications administered to treat an AE must be recorded in the subject's source documentation and documented in the eCRF.

### 11.2.3. Reporting of Serious Adverse Events

Reporting of SAEs will be conducted in accordance with the appropriate regulatory guidelines. All SAEs that occur from the time of consent to the End-of-Study visit must be reported, whether or not the event is considered associated with the study drug. The Investigator must complete the SAE Reporting Form provided by Frontage and submit it by fax or email with other relevant source documentation to sponsor within 24 hours of awareness of the event to:

[redacted]

The Investigator must also provide with urgent priority (upon receipt of a request) other relevant documentation (e.g., copies of diagnostic test results, hospital discharge summary, and/or autopsy report) and send this information by fax or email to sponsor.

All SAEs must be recorded in the subject's source documentation and documented in the eCRF. Medications administered in association with the SAE must be documented in the eCRF and in the subject's source documentation. The Investigator must also promptly notify the Institutional Review Board (IRB) /Independent Ethics Committee (IEC) of SAEs, including any follow-up information, in accordance with local institutional policy and applicable regulatory requirements.

Regulatory authorities will be notified of any AE associated with the use of the study drug that is both serious and unexpected by sponsor, in accordance with the appropriate local regulatory guidelines. Notification of the event will be made by a written, expedited safety report.

### 11.2.4. Adverse Event Follow-up

The Investigator should take all appropriate measures to ensure the safety of the subjects, including referral to a specialist if indicated. The Investigator should follow up on the outcome of any AE until the event has resolved or stabilized.

This implies that follow-up may continue after the subject discontinues from the study and that additional information may be requested. Any SAE follow-up information that occurs after database lock should be reported to sponsor.

#### 11.2.5. Hy's Law

The study will utilize Hy's Law to monitor any potential drug-induced liver injury. Hy's Law usually means that AST or ALT > 3 x ULN and TBiL > 2 x ULN, non-biliary increase (usually alkaline phosphatase < 2 x ULN) without any other diseases can explain this increase. If ALT or AST > 3 x ULN, and TBiL > 2 x ULN without any other past diseases can explain this increase, no matter ALP increases or not, re-evaluation need to be performed. Investigator will conduct subject re-evaluation, and closely follow-up of the subjects or stop the study drug due to liver function abnormality, according to Section 6.4 Subject withdrawal. The Investigator will evaluate the etiology and perform every essential examination to rule out drug-induced liver injury. If Investigator confirms the occurrence of Hy's Law, an SAE must be reported.

#### 11.2.6. Hypoglycemia

Hypoglycemia events shall be recorded on an eCRF page. The following information will be recorded for each occurrence of hypoglycemia:

- Start/end date and time
- Blood glucose values
- Symptom descriptions
- Action taken
- Severe hypoglycemia or not
- Resolved or not
- Predisposing factors
- Causal relationship with the study drug

Hypoglycemia should be treated using standard medical practice at the Investigator's discretion. Severe hypoglycemia events will be reported as an SAE.

# 12. STATISTICAL METHODOLOGY

## 12.1. Sample Size Determination

Sample size calculations based on study design and intra-subject variability were performed by the sponsor. At least 10 evaluable subjects in the sequence will be required to achieve a power of at least 0.8 for the geometric mean ratios between two treatments (sitagliptin + dorzagliatin vs.

dorzagliatin alone or sitagliptin + dorzagliatin vs. Sitagliptin alone) for $C_{max}$ or $AUC_{0\text{-}24h}$, with the equivalence bounds of 0.8 and 1.25, assuming a true geometric mean ratio of 1 and an intra-subject variability (coefficient of variation) of 16.1%, in an equivalence test using two one-sided test at a significance level of 0.05. The intra-subject variability for sitagliptin $C_{max}$ and AUC are reported to be 16.1% and 5.7%, respectively.[10] The intra-subject variability for dorzagliatin $C_{max}$ and $AUC_{0\text{-}24h}$ are estimated to be 14.0% and 6.2%, respectively.

Therefore, to ensure a satisfactory DDI assessment, and assuming a drop-out rate of 20%, we plan to enroll 15 eligible subjects by aiming to obtain 12 evaluable subjects for DDI assessment.

## 12.2. Analysis Population

The Safety Population will be defined as all subjects who receive study drug.

The PK Population will be defined as all subjects who receive study drug, have no major protocol violations, and have sufficient PK data to obtain reliable estimates of the key PK variables.

The PD Population will be defined as all subjects who receive study drug, have no major protocol violations, and have sufficient PD data to obtain reliable estimates of the key PD parameters.

The DDI Population will consist of the PK Population subjects who complete all treatments as defined by the protocol.

## 12.3. Pharmacokinetics and Pharmacodynamics

Pharmacokinetic parameters described in Section 10 will be summarized by descriptive statistics (n, mean, standard deviation, median, minimum, maximum) or frequency counts. Descriptive statistics will be calculated for dorzagliatin and sitagliptin concentrations in plasma.

Dorzagliatin and sitagliptin PK parameters ($C_{max}$, $T_{max}$ and $AUC_{0\text{-}24h}$) will be computed for each subject using non-compartmental methods and based on the actual sampling times. Descriptive statistics will be calculated for the PK parameters on Days 5, 10 and 15.

The effect of sitagliptin on the exposure of dorzagliatin will be determined by a PK comparison between the exposure of dorzagliatin on Day 10 vs. Day 15. The effect of dorzagliatin on the

exposure of sitagliptin will be determined by a PK comparison between the exposure of sitagliptin on Day 10 vs. Day 5.

The primary PK comparison will be based on the $C_{max}$ and $AUC_{0-24h}$ of dorzagliatin and sitagliptin. A mixed model under the sequential design will be used for analyzing $C_{max}$ and $AUC_{0-24h}$ values, based on natural log-transformed values, with treatments as fixed effects and subjects within sequence as random effects. The adjusted geometric mean ratios between two treatments for these parameters and the corresponding 90% confidence intervals (CI) for the ratio will be obtained by exponentiating the adjusted mean difference in logarithms and will be used for assessing PK differences between the two treatments within each PK comparison.

A Statistical Analysis Plan (SAP) will be developed and signed prior to database lock, and will describe in more detail how PK parameters will be derived.

Pharmacodynamic measurements (glucose, GLP-1, glucagon, insulin, and C-peptide) will be listed by subject for all subjects with actual sampling time. Summary statistics of glucose, GLP-1, glucagon, insulin, and C-peptide will be provided by scheduled (nominal) time point and treatment, respectively. Baseline corrected PD markers will be calculated by subtracting the baseline value from the values post oral glucose intake. The baseline for PD correction will be defined in the SAP. Plots of mean PD markers versus the scheduled time will be generated for each treatment. Derivations and statistical analysis methods of PD parameter ($AUEC_{0-4h}$, $CE_{max}$ and $CE_{av}$) will be described in detail in the SAP.

## 12.4. Demographic Characteristics

Demographic characteristics will be summarized for the subjects enrolled in the study using descriptive statistics.

An attempt will be made to enroll similar numbers of men and women in the study.

## 12.5. Exposure to Study Drugs

Each subject’s exposure to study drug will be summarized using descriptive statistics, i.e., the number of subjects exposed to each treatment.

## 12.6. Concomitant Medications

Concomitant medications will be coded using the World Health Organization (WHO) Enhanced dictionary.

## 12.7. Safety Analyses

Safety evaluations will be based on the incidence, severity, and relatedness of AEs and changes in subjects' PE findings, ECGs, vital signs, and clinical laboratory results.

Safety variables will be tabulated and presented for all subjects in the Safety Population. Data will be summarized by treatment. Adverse events will be coded using the Medical Dictionary for Regulatory Activities (MedDRA) for preferred term and system organ class, listed by subject. TEAEs will be summarized by treatment. All AEs will be summarized by relationship to study drug and by severity.

Deaths, SAEs, and AEs resulting in study discontinuation will be tabulated and detailed in narratives.

Change from baseline, defined as time of admission to the CRC or screening, whichever value is the last value prior to first dose, in clinical laboratory parameters, 12-lead ECGs, and vital sign parameters will be summarized by treatment.

Additional safety analyses may be defined in the SAP.

## 12.8. Interim Analyses

No interim analyses are planned for this study.

# 13. DIRECT ACCESS TO SOURCE DATA/DOCUMENTS

## 13.1. Study Monitoring

At regular intervals during the study, the site will be contacted through monitoring visits, letters, and telephone calls by a representative to review study progress, Investigator and subject compliance with study protocol requirements, and any emergent problems. During monitoring visits, the following points will be reviewed in accordance with all applicable regulatory requirements and Standard Operating Procedures (SOPs): original medical records and other source documents, the Investigator site file, screening logs, ICF, subject recruitment and follow-

up, SAE documentation and reporting, documentation and reporting of endpoints, study drug allocation, subject compliance with the study drug regimen, study drug accountability, concomitant therapy use, and quality of data. In addition, other required regulatory documents will be reviewed, including but not limited to: IRB/IEC composition and correspondence, laboratory certification(s), delegation of authority, and Investigator and study personnel curricula vitae.

## 13.2. Sponsor's Responsibility

The sponsor or its designee is responsible for the following:

- Selecting qualified Investigators;
- Providing Investigators with the information they need to properly conduct an investigation;
- Ensuring proper monitoring of the investigation;
- Ensuring that the applicable regulatory authorities, and all participating Investigators are properly informed of significant new information regarding AEs or risks associated with the medication being studied.

As the sponsor, Hua Medicine has delegated some responsibilities to Frontage Clinical Services, a Contract Research Organization (CRO).

## 13.3. Audits and Inspections

The sponsor's Quality Assurance Unit (or representative) may conduct audits at the study site(s). Audits will include, but are not limited to: drug supply, presence of required documents, the informed consent process, laboratory specimen processing, and comparison of eCRFs with source documents. The Investigator agrees to cooperate with audits conducted at a reasonable time and in a reasonable manner.

Regulatory authorities worldwide may also inspect the Investigator site during or after the study. The Investigator should contact the sponsor immediately if this occurs and must fully cooperate with the Inspector.

The Investigator is required to make all study documentation promptly available for inspection, review or audit at the study site upon request by sponsor, its representatives, or any appropriate regulatory agencies.

# 14. QUALITY CONTROL AND QUALITY ASSURANCE

Each investigational site will perform internal quality management of study conduct, data and biological specimen collection, documentation and completion. An individualized quality management plan may be developed to describe a site's quality management.

Quality control (QC) procedures will be implemented beginning with the data entry system and data QC checks that will be run on the database will be generated. Any missing data or data anomalies will be communicated to the site(s) for clarification/resolution.

Following written SOPs, the monitors will verify that the clinical trial is conducted, and data are generated, and biological specimens are collected, documented (recorded), and reported in compliance with the protocol, ICH GCP, and applicable regulatory requirements (e.g., GLP, Good Manufacturing Practices (GMP)).

The investigational sites will provide direct access to all trial related sites, source data/documents, and reports for the purpose of monitoring and auditing by the sponsor, and inspection by local and regulatory authorities.

# 15. ETHICS

The procedures set out in this study protocol, pertaining to the conduct, evaluation, and documentation of this study, are designed to ensure that the sponsor and the Investigator abide by GCP, including but not limited to Title 21 Code of Federal Regulations (CFR) Parts 50, 56, and 312 and the ICH guidelines and directives. Compliance with these regulations also constitutes compliance with the ethical principles described in the current revision of the Declaration of Helsinki, Fortaleza 2013 and applicable local regulatory requirements and law.

Copies of these materials are available from Hua Medicine and Frontage Clinical Services (the CRO) designee by request. The purpose of these regulations, legal obligations and guidance is to define the standards and principles for the proper conduct of clinical studies that have been

developed by the medical, scientific, and regulatory communities. They are not intended to impede or restrict clinical research.

The ethical standards defined within GCP are intended to ensure that:

1. Human subjects are provided with an adequate understanding of the possible risks of their participation in the study, and that they have a free choice to participate or not;
2. The study is conducted with diligence and in conformance with the protocol in such a way as to protect subject safety and ensure the integrity of the findings;
3. The potential benefits of the research justify the risks.

The Investigator is responsible for protecting the rights, safety, and welfare of subjects under his/her care, and for the control of the medications under investigation.

The Principal Investigator will ensure that all persons assisting with the study are adequately informed about the protocol, any amendments to the protocol, the study drug, and their study related duties and functions. The Investigator will maintain a list of sub-Investigators and other appropriately qualified persons to whom he or she has delegated significant study-related duties. Individuals ineligible to conduct or work on clinical studies, including those ineligible as a result of debarment under the Generic Drug Enforcement Act of 1992, will not be allowed to conduct or work on studies sponsored by Hua Medicine. The Investigator is required to immediately disclose to sponsor in writing, if any person involved in the conduct of the study is debarred pursuant to a hearing by US FDA under this anti-fraud law, or if any proceeding for debarment is pending, or is (to the best of the Investigator’s knowledge) threatened.

The rest of this section of the protocol describes in more detail the specific GCP-defined responsibilities the Investigator assumes by agreeing to participate in this study.

## 15.1. Ethics Review

The Investigator (or designee) must submit this study protocol, the sponsor approved ICF, information intended for distribution to subjects, subject recruitment materials, and other appropriated documents to the appropriate IRB or IEC. Following IRB or IEC review, a copy of the signed, written and dated approval will be provided to the sponsor, along with a list of the IRB/IEC composition.

The approval/favorable opinion should clearly state the study (study number, protocol title, and version number), the documents reviewed (Protocol, ICF, IB, etc.) and the date of the review. The study will not commence at the study site until sponsor has received a copy of this written and dated approval/favorable opinion.

During the study, any amendment to the protocol and the ICF (as appropriate) shall be submitted to the IRB/IEC. The IRB/IEC should also be informed of any event likely to affect the safety of subjects or the continued conduct of the study, in particular any change in safety. Additionally, all updates to the IB will be sent to the IRB/IEC. A progress report will be sent to the IRB/IEC and the protocol will be reviewed annually (e.g., re-approved) or more frequently, as required by IRB/IEC or local regulations.

The Investigator will notify the IRB/IEC of the conclusion of the clinical study within 1 month of completion or termination of the study. The final report sent to the IRB/IEC will also be sent to sponsor, along with the completed eCRFs and all necessary regulatory documents, thereby fulfilling the Investigator's regulatory responsibility.

The Investigator will maintain a copy of all correspondence with the IRB/IEC, including copies of approved documents. The Investigator will also maintain a copy of the IRB/IEC membership list, including members' occupation and qualifications (or a statement confirming compliance with GCP requirements for committee composition). An IRB or IEC General Assurance Number may be accepted in lieu of a membership roster.

## 15.2. Ethical Conduct of the Study

This study will be conducted in accordance with GCP as delineated by Title 21 CFR Parts 50, 56, and 312, and the ICH guidelines and directives. Participating Investigators, including members of the Committees and National Coordinators, will receive compensation for their time but will receive no financial profit from their activities related to the study.

Before the first subject is enrolled in the study, all ethical, regulatory, and legal requirements must be met.

An Investigator participating in this study is required to maintain complete and accurate study documentation in compliance with current GCP standards and all applicable local regulations related to the conduct of a clinical study.

### 15.3. Written Informed Consent

Prior to performing any study-related activities under this protocol, including screening tests and assessments, written informed consent with the approved ICF must be obtained from the subject or subject's legally authorized representative, as applicable, in accordance with local practice and regulations. Written informed consent must be obtained from all subjects participating in a clinical study conducted by Hua Medicine.

The background of the proposed study, the procedures, the benefits and risks of the study, and that study participation is voluntary for the subject must be explained to the subject. The subject must be given sufficient time to consider whether to participate in the study.

A copy of the ICF, signed and dated by the subject, must be given to the subject. Confirmation of a subject's informed consent must also be documented in the subject's source documentation prior to any testing under this protocol, including screening tests and assessments.

Each consent form should contain an authorization allowing the Principal Investigator(s) and the sponsor to use and disclose patient health information (PHI) in compliance with local law.

The originally signed consent form will be retained with the study records.

## 16. DATA HANDLING AND RECORD KEEPING

### 16.1. Data Collection

All data obtained for analysis in the clinical study described in this protocol will be documented in the eCRF. Data reported in the eCRFs should be consistent with and substantiated by the subject's medical record and original source documents.

Prior to the start of the study, the Principal Investigator will complete a Delegation of Authority form (Site Signature and Delegation Log), showing the signatures and handwritten initials of all individuals and the delegation of responsibilities, such as identifying those individuals who are authorized to make or change entries on eCRFs.

### 16.2. Case Report Form Completion

All eCRF entries, corrections, and alterations must be made by the investigator or authorized study-site personnel. Data within the eCRF will be monitored by the Clinical Research Associate according to the Monitoring Plan. Queries will be generated based on discrepancies found while

monitoring. Site personnel will review and respond to these queries appropriately. Additionally, the sponsor may periodically perform aggregate data reviews, which could result in queries being generated for site personnel resolution. The final, completed eCRF for each subject must be signed and dated by the Principal Investigator to signify that the Principal Investigator has reviewed the eCRF and certifies it to be complete and accurate.

## 16.3. Database Management, Data Clarification, and Quality Assurance

A designated CRO will be responsible for data management. The Data Manager will develop a Data Management Plan (DMP) document and provide it to sponsor for approval. The DMP document will define all activities in the data collection and cleaning process. The detailed DMP will be based on the protocol, work scope, contract, analysis plans, data-flows, eCRFs, data cleaning procedures, other supporting documents, and data management standards and practices.

The programmed data validations will be run, and manual review will be conducted to check for database completeness and consistency, and queries will be generated upon data entry or via review by a Clinical Data Manager after entry. The sites will respond to the data queries in a timely manner.

Prior and concomitant medications will be coded using the World Health Organization (WHO) Drug Enhanced Dictionary (latest version, to be noted in DMP). All medical history and AEs will be coded using the Medical Dictionary for Regulatory Activities (MedDRA), using latest version, to be noted in DMP.

Quality control procedures will be conducted prior to database lock according to the designated CRO SOPs.

When the database has been declared to be complete and accurate, it will be locked. Any changes to the database after that time will only be made by joint written agreement between Hua Medicine, the study Statistician, the Data Manager, and the Quality Assurance Auditor according to designated CRO SOPs.

## 16.4. Inspection of Records/Source Documents

According to the ICH guidelines for GCP, the sponsor or designee must verify data entered in the eCRF entries against the source documents, except for the pre-identified source data directly documented in the eCRF (if applicable). The ICF will include a statement by which the subject

allows the sponsor's duly authorized personnel, the IRB/IEC, and the regulatory authorities to have direct access to source data that supports the data in the eCRF (e.g., subject's medical file, appointment books, and original laboratory records). These personnel, bound by professional secrecy, must keep confidential all personal medical information.

The objective of source document verification (SDV) is to comply with GCP and international regulatory requirements and to reduce the risks of fraud. Source document verification means ensuring that the source documents are an accurate and verifiable reflection of the subject's participation in the study and that all relevant information that is recorded in the source document is accurately entered into the eCRF.

Where source documents serve as the basis for deriving data for the study, SDV should ensure that these documents are correctly labeled and filed, and that the data derived from them are correct. All source documents pertaining to this study will be maintained by the Investigator and made available for inspection by authorized persons. If electronic progress notes and other electronic source documents are not Title 21 CFR Part 11 compliant, they are not considered a valid source for this study. All progress notes must be dated and signed by the Principal Investigator or sub-Investigator at the time of the visit. The sponsor reserves the right to terminate the study for refusal of the Investigator to supply original source documentation for this clinical study.

The Investigator will note in a source independent from the eCRF the following information:

1. Information to confirm that the subject exists (e.g., initials, date of birth, sex)
2. Confirmation that the subject satisfies the inclusion/exclusion criteria
3. Confirmation that the subject is taking part in the clinical study
4. Confirmation of the informed consent process
5. Visit dates and documentation of protocol assessments and procedures
6. Information concerning all AEs
7. Details of concomitant and investigational medications

Source document verification is not a substitute for clinical study monitoring, the purpose of which is to ensure that the protocol has been followed correctly, the eCRF has been fully and accurately completed, SDV has been carried out, and the study timelines and enrollment goals and requirements have been met.

## 16.5. Retention of Records

The Investigator/institution must maintain all study documentation as confidential and take measures to prevent accidental or premature destruction of these documents.

Essential documents should be retained until at least 2 years after the last approval of a marketing application in an ICH region and until there are no pending or contemplated marketing applications in an ICH region or at least 2 years have elapsed since the formal discontinuation of clinical development of the investigational product (e.g., the Investigational New Drug application is withdrawn). These documents should be retained for a longer period, however, if required by the applicable regulatory requirements or by an agreement with sponsor.

The Investigator/institution must notify sponsor prior to destroying any study essential documents.

If the Investigator/institution can no longer ensure archiving, he/she shall inform the sponsor. The relevant records shall be transferred to a mutually agreed upon designee.

# 17. CONFIDENTIALITY

All information disclosed or provided by sponsor (or designee) or produced during the study including, but not limited to, the protocol, the eCRFs, the IB, and the results obtained during the course of the study (if applicable), are confidential. The Investigator or any person under his/her authority agrees to undertake to keep confidential and not to disclose the information to any third party without the prior written approval of sponsor.

However, submission of this protocol and any other necessary documentation to the IRB/IEC is expressly permitted, the IRB/IEC members having the same obligation of confidentiality.

All information generated in this study must be considered highly confidential and must not be disclosed to any persons not directly concerned with the study without prior written permission from sponsor. However, authorized regulatory officials and sponsor personnel (or designee) will be allowed full access to inspect and copy the records. The copied and inspected records will remain at the site and will not be transmitted or removed from the site.

All study drugs, subject bodily fluids, and/or other materials collected shall be used solely in accordance with this protocol, unless otherwise agreed to in writing by sponsor and responsible ethics committee(s) or regulatory authorities.

Subjects will be identified only by unique subject numbers in eCRFs. Their full names may, however, be made known to a regulatory agency or other authorized officials in the event of inspections. Documents containing the full name or other personally identifiable information of the subject are to remain at the site. This information will not be transferred to sponsor nor be contained in regulatory filings. In the event of inspections by authorized agencies, this subject identification may be disclosed.

## 17.1. Subject Data Protection

Prior to any testing under this protocol, including screening tests and assessments, candidates must also provide all authorizations required by local law (e.g., PHI authorization in North America).

The subject will not be identified by name in the eCRF or in any study reports and these reports will be used for research purposes only. The sponsor, its partner(s) and designee(s), and various government health agencies may inspect the records of this study. Every effort will be made to keep the subject’s personal medical data confidential.

The sponsor will protect individual subject information to the fullest extent possible during this study. At no time will a subject become identified in any publication or presentation.

However, the subject may have to become identified in the event of a regulatory authority auditor inspection in order to verify the accuracy of the data. Access to subject information is at the discretion of sponsor and cannot occur prior to database lock or other specified events as determined solely by the discretion of sponsor.

# 18. STUDY PROTOCOL AMENDMENTS

The Investigator will not make any changes to this protocol without prior written consent from sponsor and subsequent approval by the IRB/IEC. Any permanent change to the protocol, whether it is an overall change or a change for specific study center(s), must be handled as a protocol amendment. Any amendment to the protocol that appears necessary as the study progresses will be fully discussed by the Investigator(s) and sponsor. If agreement is reached

regarding the need for an amendment, it will be written by sponsor. The written amendment must be submitted to the chairman of the IRB/IEC identified with this responsibility. Except for administrative amendments, Investigators must await IRB/IEC approval of protocol amendments before implementing the change(s).

Administrative amendments are defined as having no effect on the safety of the research subjects, scope of the investigation, or quality of the study. However, a protocol change intended to eliminate an apparent immediate hazard to subjects should be implemented immediately, and the IRB/IEC notified within 5 days. The sponsor will ensure submission of any protocol amendments to the appropriate regulatory agencies.

When, in the judgment of the chairman of the local IRB/IEC, the Investigators, and/or sponsor, the amendment to the protocol substantially alters the study design and/or increases the potential risk to the subject; the currently approved written ICF will require similar modification. In such cases, the Investigator will obtain repeat informed consent from subjects enrolled in the study before expecting continued participation.

# 19. PUBLICATION

All information concerning the product as well as any information such as clinical indications for the study drugs, their formula, their formulation, methods of manufacture and other scientific data relating to it, that have been provided by sponsor or designee, and are unpublished, are confidential and must remain the sole property of Hua Medicine.

The Investigator will agree to use the information only for the purposes of carrying out this study and for no other purpose unless prior written permission from sponsor is obtained. The sponsor has full ownership of the eCRFs completed as part of the study.

By signing the study protocol, the Investigator agrees that the results of the study may be used for the purposes of national and international registration, publication, and information for medical and pharmaceutical professionals by sponsor. If necessary, the authorities will be notified of the Investigator’s name, address, qualifications, and extent of involvement.

The sponsor or designee will prepare a final report on the study. The Investigator may not publish or present any information on this study without first presenting the information to sponsor for review.

# 20. REFERENCES

1. Hua Medicine (Shanghai), Ltd. China: Investigator's Brochure for Dorzagliatin (HMS5552), Second edition, December 29, 2017.
2. Zhu D, Gan S, Liu Y, et. al. Dorzagliatin monotherapy in Chinese patients with type 2 diabetes: dose-ranging, randomised, double-blind, placebo-controlled, phase 2 study. Lancet Diabetes Endocrinol, 2018; 6 (8): 627-636.
3. [redacted] Package Insert, 2018, [redacted] USA
4. [redacted] Package Insert, 2018, [redacted] USA.
5. [redacted] Package Insert, 2017, [redacted] USA.
6. [redacted] [redacted] [redacted] Package Insert, 2017, [redacted] USA.
7. [redacted] Package Insert, 2017, [redacted] USA.
8. [redacted] Package Insert, 2017, [redacted] USA.
9. [redacted] Package Insert, 2017, [redacted] USA.
10. Chung I, Oh J, Lee SH, et. al. A post hoc analysis of intra-subject coefficients of variation in pharmacokinetic measures to calculate optimal sample sizes for bioequivalence studies. Transl Clin Pharmacol, 2017; 25 (4): 179-182.

# APPENDIX A. LABORATORY ASSESSMENTS

| Hematology | Clinical Chemistry | Urinalysis |
|---|---|---|
| Hemoglobin (Hgb) | Blood urea nitrogen (BUN) | pH |
| Hematocrit (Hct) | Creatinine | Specific gravity |
| Platelet cout | Total bilirubin | Protein |
| Red blood cell (RBC) count | Alkaline phosphatase | Glucose |
| White blood cell (WBC) count | Aspartate transaminase (AST) | Ketones |
| with differential | Alanine transaminase (ALT) | Bilirubin |
| | Gamma-glutamyl | Blood |
| | transferase (GGT) | Nitrites |
| | Lactic dehydrogenase (LDH) | Leukocytes |
| | Glucose | Urobilinogen |
| | Albumin | Microscopic urine analysis |
| | Total protein | |
| | Bicarbonate | |
| | Phosphate | |
| | Sodium | |
| | Potassium | |
| | Chloride | |
| | Calcium | |
| | Total cholesterol | |
| | Triglyceride | |
| | HDL-C | |
| | LDL-C | |
| | Urate | |

| Urine/Saliva Drug Screen | Serology Screen |
|---|---|
| Amphetamines | Human immunodeficiency virus (HIV) |
| Barbiturates | Hepatitis B surface |
| Cannabinoids | antigen (HBsAg) |
| Cocaine metabolites | Hepatitis C virus (HCV) |
| Opiates | |
| Benzodiazepines | **Other** |
| Ethyl alcohol | Urine Pregnancy test (all women, screening, Day -1, End-of-Study) |
| | Serum FSH and estradiol (postmenopausal women, screening) |
| | HbA1c test (screening only) |
| | C-peptide test (screening only) |
| | |
| | **PD assessments** |
| | Glucose |
| | GLP-1 |
| | Glucagon |
| | Insulin |
| | C-peptide |

# TABLE OF CONTENTS

TABLE OF CONTENTS ........ 3
1. PROTOCOL SYNOPSIS ........ 8
2. LIST OF ABBREVIATIONS ........ 14
3. INTRODUCTION ........ 17
3.1 Background Information ........ 17
3.2 Preclinical Data ........ 18
3.3 Summary of Clinical Studies ........ 19
3.4 Drug Interaction Potential of Dorzagliatin ........ 22
4. STUDY RATIONALE AND OBJECTIVES ........ 24
4.1. Study Rationale ........ 24
4.2. Primary Objectives ........ 24
4.3. Secondary Objective ........ 25
5. INVESTIGATIONAL PLAN ........ 25
5.1. Overall Study Design ........ 25
5.2. Number of Subjects ........ 29
5.3. Treatment Assignment ........ 29
5.4. Criteria for Study Termination ........ 29
6. SELECTION AND WITHDRAWAL OF SUBJECTS ........ 29
6.1. Subject Inclusion Criteria ........ 29
6.2. Subject Exclusion Criteria ........ 31
6.3. Prohibitions and Restrictions ........ 33
6.4. Subject Withdraw Criteria ........ 33
7. STUDY PROCEDURES ........ 34
7.1. Screening Visit and Run-in Period ........ 35
7.2. Domiciled at CRC on Day -2 through Day 17 ........ 36
7.2.1. Study Check-in Visit: Day -2 and Day -1 ........ 36

7.2.2. Day 1 - Day 4 ........ 37
7.2.3. Day 5 ........ 38
7.2.4. Day 6 - Day 9 ........ 38
7.2.5. Day 10 ........ 39
7.2.6. Day 11 - Day 14 ........ 40
7.2.7. Day 15 ........ 40
7.2.8. Day 16 ........ 41
7.2.9. Day17: End-of-Study Procedures ........ 41
7.3. Early Withdrawal Visit ........ 41
7.4. Time Windows for Procedures ........ 42
8. TREATMENT OF SUBJECTS ........ 42
8.1. Description of Study Drugs ........ 42
8.2. Methods of Assigning Subjects to Dose Groups ........ 43
8.3. Blinding ........ 43
8.4. Treatment Discontinuation/Subject Withdrawal ........ 43
8.5. Prior and Concomitant Medications and Substances ........ 43
8.6. Meals ........ 43
9. STUDY DRUG MATERIALS AND MANAGEMENT ........ 44
9.1. Study Drug Packaging and Labeling ........ 44
9.2. Study Drug Storage and Accountability ........ 44
9.3. Study Drug Preparation ........ 45
9.4. Study Drug Handling and Disposal ........ 45
10. PHARMACOKINETIC/PHARMACODYNAMIC ASSESSMENTS ........ 45
10.1. Pharmacokinetic Sample Collection and Storage ........ 45
10.2. Pharmacokinetic Specimen Labeling ........ 46

10.3. Pharmacokinetic Sample Shipping Instructions ..... 46

10.4. Bioanalytical Methodology ..... 47

10.5. Pharmacokinetic Parameters ..... 47

10.6. Pharmacodynamic Assessments ..... 47

11. ASSESSMENT OF SAFETY ..... 48

11.1. Safety Parameters ..... 48

11.1.1. Demographic/Medical History ..... 48

11.1.2. Vital Signs ..... 49

11.1.3. Physical Examination, Height, Weight, and Body Mass Index ..... 49

11.1.4. Electrocardiogram ..... 49

11.1.5. Clinical Laboratory Assessments ..... 50

11.2. Adverse Events ..... 51

11.2.1. Definitions of Adverse Events ..... 51

11.2.2. Recording Adverse Events ..... 54

11.2.3. Reporting of Serious Adverse Events ..... 55

11.2.4. Adverse Event Follow-up ..... 55

11.2.5. Hy's Law ..... 56

11.2.6. Hypoglycemia ..... 56

12. STATISTICAL METHODOLOGY ..... 56

12.1. Sample Size Determination ..... 56

12.2. Analysis Population ..... 57

12.3. Pharmacokinetics and Pharmacodynamics ..... 57

12.4. Demographic Characteristics ..... 58

12.5. Exposure to Study Drugs ..... 58

12.6. Concomitant Medications ..... 59

12.7. Safety Analyses ........ 59
12.8. Interim Analyses ........ 59
13. DIRECT ACCESS TO SOURCE DATA/DOCUMENTS ........ 59
13.1. Study Monitoring ........ 59
13.2. Sponsor’s Responsibility ........ 60
13.3. Audits and Inspections ........ 60
14. QUALITY CONTROL AND QUALITY ASSURANCE ........ 61
15. ETHICS ........ 61
15.1. Ethics Review ........ 62
15.2. Ethical Conduct of the Study ........ 63
15.3. Written Informed Consent ........ 64
16. DATA HANDLING AND RECORD KEEPING ........ 64
16.1. Data Collection ........ 64
16.2. Case Report Form Completion ........ 64
16.3. Database Management, Data Clarification, and Quality Assurance ........ 65
16.4. Inspection of Records/Source Documents ........ 65
16.5. Retention of Records ........ 67
17. CONFIDENTIALITY ........ 67
17.1. Subject Data Protection ........ 68
18. STUDY PROTOCOL AMENDMENTS ........ 68
19. PUBLICATION ........ 69
20. REFERENCES ........ 70
APPENDIX A. LABORATORY ASSESSMENTS ........ 71

# TABLES

Table 5-1 Schedule of Procedures ........ 27
Table 5-2: Treatment Assignment and PK Sample Collection ........ 29
Table 10-1: PK Terms ........ 47

# Summary of Changes

| Main changes from Version 1.0 (11 November 2018) to Version 1.1 (27 December 2018) | |
|---|---|
| **Section** | **Change** |
| 1.0 Synopsis<br>4.3 Secondary Objective, and throughout protocol. | Wording of secondary objective changed to remove "serum" since some PD analytes are measured in plasma and some are measured in serum.<br>Throughout protocol, when matrix of PD analytes is noted, wording changed from "serum levels of GLP-1, glucagon, glucose, insulin, and C-peptide" to "GLP-1 and glucagon in plasma, and glucose, insulin, and C-peptide in serum". |
| 5.1 Overall Study Design | Schedule of Procedures footnote to Run-in Period amended to reflect that sitagliptin may be dispensed to eligible subjects prior to Day -14. |
| 5.2 Number of Subjects | Changed "Evaluable subjects are defined as subjects included in the **PD** population…" to "Evaluable subjects are defined as subjects included in the **DDI** population…" |
| 6.1 Subject Inclusion Criteria | Removed "screening" to clarify that criteria applies to screening and/or Day -1. |
| 6.2 Subject Exclusion Criteria | Removed "screening" to clarify that criteria applies to screening and/or Day -1. |
| 7.2.1 Study Check-in Visit | Added physical examination on Day -1 to be consistent with remainder of protocol. |
| 7.2.3-7.2.9 (Day 5-Day 17) | Changed 18-hour post-dose PK blood draw to occur on day after dosing.<br>Removed blood glucose assessment by glucometer on Day 17 to be consistent with remainder of protocol. |
| 10.6 Pharmacodynamic Assessments | Changed volume of blood collected for PD measurements: 8.5 mL (instead of 8.0 mL) collected for measurement of plasma GLP-1 and glucagon, and 7 mL (instead of 6 mL) collected for measurement of serum glucose, insulin and C-peptide.<br>Sample collection and processing steps for measurement of GLP-1 and glucagon added. |

# 1. PROTOCOL SYNOPSIS

| | |
|---|---|
| **Name of Sponsor/Company:**<br>Hua Medicine (Shanghai) Ltd. | |
| **Name of Investigational Product:**<br>Dorzagliatin | |
| **Name of Active Ingredient:**<br>Dorzagliatin: (S)-2-[4-(2-Chloro-phenoxy)-2-oxo-2,5-dihydro-pyrrol-1-yl]-4-methyl-pentanoic acid [1-((R)-2,3-dihydroxy-propyl)-1H-pyrazol-3-yl]-amide | |
| **Title of Study:**<br>A Phase 1, Open-Label, Sequential, Multiple-Dose, Drug-Drug Interaction Study of Dorzagliatin and Sitagliptin in Subjects with Type 2 Diabetes Mellitus | |
| **Study Center(s):** | |
| **Site:** Frontage Clinical Services, Inc., Secaucus, NJ<br>**Principal Investigator:** [redacted]<br>**Sub-investigator:** [redacted] | **Site:** To be Determined |
| **Studied Period (years):**<br>Estimated date first subject enrolled: Dec 2018<br>Estimated date last subject completed: Mar 2019 | **Phase of development:** Phase 1 |
| **Objectives:**<br>**Primary:**<br>• To assess the potential pharmacokinetic interaction between dorzagliatin and sitagliptin in subjects with type 2 diabetes mellitus;<br>• To evaluate the safety and tolerability of dorzagliatin with simultaneous administration of sitagliptin in subjects with type 2 diabetes mellitus.<br>**Secondary:**<br>• To assess the pharmacodynamic responses of glucose, GLP-1, glucagon, insulin, and C-peptide following dorzagliatin, sitagliptin, or simultaneous administration of dorzagliatin and sitagliptin in subjects with type 2 diabetes mellitus | |
| **Methodology:**<br>This is a Phase 1, open-label, sequential, multiple-dose, drug-drug interaction (DDI) study of glucokinase (GK) activator dorzagliatin and sitagliptin in subjects with type 2 diabetes mellitus (T2DM).<br>Study drugs will be administered in the following treatment scheme: | |

| Day | Sitagliptin | Dorzagliatin | PK Sample Collection |
|---|---|---|---|
| 1-5 | 100 mg (QD) for 5 days | - | Day 5:<br>Sitagliptin alone |
| 6-10 | 100 mg (QD) for 5 days | 75 mg (BID) for 4 days<br>Morning dose only on Day 10 | Day 10:<br>Sitagliptin + Dorzagliatin |
| 11-15 | - | 75 mg (BID) for 4 days<br>Morning dose only on Day 15 | Day 15:<br>Dorzagliatin alone |

Study drug will be taken 60 (±5) minutes prior to meals.

Eligible subjects will have a minimum 12-day run-in period prior to admission to the Clinical Research Center (CRC), during which time they will self-administer sitagliptin 100 mg QD each morning up until and including Day -2. Sitagliptin will be dispensed by the CRC. Subjects will complete a diary to record sitagliptin doses taken each day and will be advised to monitor their blood glucose levels and report changes in health status to the CRC. A telephone call will be placed to all subjects at approximately Day 6 (±2 day) of the run-in period to assess general health and collect adverse event (AE) information.

Following completion of the run-in period, eligible subjects will be admitted to the CRC on Day -2, have a total of 18 overnight stays, and be discharged after End-of-Study procedures are completed on Day 17, or at early termination.

Blood samples for the determination of dorzagliatin and sitagliptin concentrations and corresponding pharmacokinetic (PK) analysis will be collected at the following time points on Days 5, 10 and 15: pre-dose [redacted] and [redacted] post dose.

Subjects will consume a 75-gram glucose solution 30 minutes following study drug dose administration on Days 5, 10 and 15, in lieu of breakfast. Pharmacodynamic (PD) responses will be evaluated by measuring Glucagon-like Peptide-1 (GLP-1) and glucagon in plasma, and glucose, insulin, and C-peptide in serum within 60 minutes prior to oral glucose intake and 0.5, 1, 1.5, 2, 2.5, 3 and 4 hours after oral glucose intake.

Safety assessments will include monitoring of AEs, blood glucose via glucometer readings, vital signs (blood pressure, pulse rate, respiratory rate and oral temperature), clinical laboratory findings, resting 12-lead electrocardiograms (ECGs), and physical examination (PE) findings.

Vital signs will be assessed at screening, on Day -1, on Days 1-15 within 60 minutes prior to each morning and evening dose, and on Day 17. Blood glucose will be measured using a glucometer three times each day on Days -2 (prior to first meal after admission) through Day 16, within 60 minutes before each meal and after study drug dosing when applicable. Clinical laboratory evaluations (chemistry, hematology, urinalysis) will be performed at screening, on Days -1, 5, 10, 15 and 17. A resting 12-lead ECG will be completed at screening, on Day -1, and 2 hours post-dose on Days 5, 10, 15 and 17. Physical examinations will be conducted at screening, on Day -1, and on Day 17.

Subjects who terminate study participation early will have Day 17 assessments performed, if possible, prior to discharge from the CRC.

**Number of Subjects (planned):**

This study will plan to enroll up to 15 eligible subjects. Subjects may be enrolled singly or in groups. In order to have at least 12 evaluable subjects complete the study, additional subjects may be enrolled to replace subjects who discontinue study participation prematurely.

**Inclusion Criteria:**

1. Subjects diagnosed with T2DM within at least 3 months prior to screening and are currently taking a stable dose of either of the following therapy with no change in the dose for at least 4 weeks prior to screening:
    a. ≥1000 mg per day of metformin
    b. a dipeptidyl peptidase 4 (DPP-4) inhibitor
    c. a sodium-glucose cotransporter-2 (SGLT2) inhibitor
    d. metformin plus a DPP-4 inhibitor
    e. metformin plus a SGLT2 inhibitor
2. Subjects must agree to change their current therapy to 100 mg sitagliptin QD for at least 14 days prior to dosing on Day 1;
3. In stable general health as determined by the Investigator at screening evaluation performed no more than 42 days prior to dosing on Day 1;
4. Male and/or female subjects between the ages of 30 and 65 years, inclusive;
5. Are capable of giving informed consent and complying with study procedures;
6. Body Mass Index (BMI) of 19 to 38 kg/m$^2$, inclusive, at screening;
7. Fasting C-peptide test result >0.3 nmol/L (>0.90 ng/mL);
8. HbA1c ≥7% and ≤10.5%;
9. Females must be using one of the following birth control methods: a non-hormonal intrauterine device (IUD) in place for at least 3 months; barrier method (condom, diaphragm) with spermicide for at least 14 days prior to dosing on Day 1 and throughout the study; surgical sterilization of the partner (vasectomy for 6 months minimum); OR are surgically sterile OR postmenopausal, as defined below:
    a. Surgically sterile for at least 3 months prior to screening by one of the following means:
        - Bilateral tubal ligation
        - Salpingectomy (with or without oophorectomy)
        - Surgical hysterectomy
        - Bilateral oophorectomy (with or without hysterectomy)
    b. Postmenopausal, defined as following:
        - Last menstrual period greater than 12 months prior to screening, confirmed by FSH and estradiol levels at screening;
10. Nonsmoker, defined as not having smoked or used any form of tobacco for more than 6 months prior to screening;
11. Willing and able to adhere to study restrictions and to be confined at the clinical research center.

**Exclusion Criteria:**

1. Fasting blood glucose at screening or Day -1 ≤110 or ≥270 mg/dL (one repeat screening fasting blood glucose allowed);
2. Type 1 diabetes mellitus, or latent autoimmune diabetes in adults; diabetic neuropathy, retinopathy or nephropathy;
3. Reported incidence of severe hypoglycemia, defined by denoting severe cognitive impairment requiring external assistance for recovery, within 3 months prior to screening and/or within 3 months prior to dosing on Day 1;
4. Known contraindications to sitagliptin;

5. Clinically significant gastrointestinal disorder (e.g., gastroparesis, constipation, diarrhea, colitis, gastrointestinal tract obstruction, hiatal hernia with reflux esophagitis, or cholestasis);
6. History or symptoms of clinically significant cardiovascular disease, particularly coronary artery disease, arrhythmias, atrial tachycardia, or congestive heart disease within one year prior to screening;
7. History of more than three urinary tract infections and/or more than three genital fungal infections in the last 12 months. If one or more genital fungal infections in the last 12 months required systemic treatment to resolve, Investigator will determine eligibility based on treatment required;
8. Reported history of clinically significant central nervous system disease including: transient ischemic attack, stroke, seizure disorder, depression, or behavioral disturbances within one year prior to screening;
9. Reported history of liver disease (e.g., hepatitis, cirrhosis within one year of screening and/or liver enzymes (alanine aminotransferase (ALT), aspartate aminotransferase (AST), gamma-glutamyltransferase (GGT), total bilirubin (TBiL)) >2 times the upper limit of normal (ULN));
10. Reported history of clinically significant renal disease;
11. Estimated glomerular filtration rate (eGFR) ≤60 mL/min/1.73m$^2$;
12. Acute or chronic metabolic acidosis, including diabetic ketoacidosis;
13. Known or suspected malignancy;
14. Any reported hypersensitivity (anaphylaxis and angioedema) or intolerance to sitagliptin;
15. Antidiabetic treatment with insulin, sulfonylureas, thiazolidinediones or GLP-1 agonist within 3 months prior to screening;
16. Systolic blood pressure <90 or ≥160 mmHg or diastolic blood pressure <60 or ≥100 mmHg at screening (may be repeated once);
17. A hospital admission or major surgery within 90 days prior to screening;
18. Uncontrolled hypertriglyceridemia >500 mg/dL;
19. Positive blood screen for human immunodeficiency virus (HIV), hepatitis B surface antigen (HBsAg), or hepatitis C antibody;
20. Positive pregnancy test result;
21. Female is breast-feeding or planning to become pregnant;
22. Treated with any investigational drugs within 6 weeks prior to screening;
23. Reported history of prescription drug abuse, or illicit drug use within 6 months prior to screening;
24. Reported history of alcohol abuse according to medical history within 6 months prior to screening;
25. A positive screen for alcohol or drugs of abuse;
26. Reported history of donation or blood collection of more than 1 unit (approximate 450 mL) of blood (or blood products) or acute loss of blood during the 90 days prior to screening;
27. Use of prescription or over-the-counter (OTC) medications, and herbal (including St John's Wort, herbal teas, garlic extracts) within 14 days prior to dosing (except for sitagliptin dosing during run-in period). No angiotensin-converting enzyme (ACE) inhibitors are allowed; (Note: Use of acetaminophen at <1 g/day is permitted until 24 hours prior to dosing);
28. An unwillingness of male participants to use appropriate contraceptive measures if engaging in sexual intercourse with a female partner of childbearing potential. Appropriate measures include use of a condom and spermicide and, for female partners, use of an IUD, diaphragm with spermicide, oral contraceptives, injectable progesterone, progesterone subdermal implants, or a tubal ligation. Sexual intercourse with pregnant or lactating women is prohibited.

**Study Drugs, Dosage and Mode of Administration:**

Dorzagliatin will be provided as a film-coated tablets in 75 mg strength for oral administration 60 minutes prior to a meal.

Sitagliptin will be provided as [redacted] 100 mg tablets for oral administration 60 minutes prior to a meal.

**Duration of Treatment:**

The total duration of participation in the study for each subject is about 59 days (up to 28-day screening period, 12-day run-in period and 19-day in-clinic period).

**Criteria for Evaluation:**

**Safety:**

Safety assessments will include monitoring of AEs, blood glucose via glucometer readings, vital signs (blood pressure, pulse rate, respiratory rate and oral temperature), clinical laboratory findings, 12-lead ECGs, and PE findings.

**Pharmacokinetics:**

The plasma concentration-time data for dorzagliatin and sitagliptin will be analyzed using non-compartmental methods to calculate pharmacokinetic parameters. Actual dosing and sampling times will be used for analyses. The primary PK parameters of interest are: $C_{max}$, $T_{max}$ and $AUC_{0-24h}$. Additional parameters may be estimated and reported, as appropriate.

**Pharmacodynamics:**

Pharmacodynamic (PD) responses will be evaluated by measurement of GLP-1 and glucagon in plasma, and glucose, insulin and C-peptide in serum. The PD parameters may include, but not limited to, $AUEC_{0-4h}$, $CE_{max}$ and $CE_{av}$.

**Statistical Methods:**

Baseline demographic, concentration and safety data will be listed and summarized by descriptive statistics (n, mean, standard deviation, median, minimum, maximum) or frequency counts. Descriptive statistics will be calculated for dorzagliatin and sitagliptin concentrations in plasma.

Dorzagliatin and sitagliptin PK parameters ($C_{max}$, $T_{max}$ and $AUC_{0-24h}$) will be computed for each subject using non-compartmental methods based on the actual sampling times. Descriptive statistics will be calculated for the PK parameters for Days 5, 10 and 15.

The effect of sitagliptin on the exposure of dorzagliatin will be determined by a PK comparison between the exposure of dorzagliatin on Day 10 vs. Day 15. The effect of dorzagliatin on the exposure of sitagliptin will be determined by a PK comparison between the exposure of sitagliptin on Day 10 vs. Day 5.

The primary PK comparison will be based on the $C_{max}$ and $AUC_{0-24h}$ of dorzagliatin and sitagliptin. A mixed model under the sequential design will be used for analyzing $C_{max}$ and $AUC_{0-24h}$ values, based on natural log-transformed values, with treatments as fixed effects and subjects as random effects. The adjusted geometric mean ratios between two treatments for these parameters and the corresponding 90% confidence intervals (CI) for the ratio will be obtained by exponentiating the adjusted mean difference in logarithms and will be used for assessing PK differences between the two treatments within each PK comparison.

Baseline corrected PD markers will be calculated by subtracting the baseline value from the values post oral glucose intake. Plots of mean PD markers versus the scheduled time will be generated for each treatment. Derivations and statistical analysis methods of PD parameters ($AUEC_{0-4h}$, $CE_{max}$ and $CE_{av}$) will be described in detail in Statistical Analysis Plan (SAP).

This study will be conducted in accordance with the guidelines of Good Clinical Practices (GCPs) including archiving of essential documents.

# 2. LIST OF ABBREVIATIONS

The following abbreviations and specialist terms are used in this study protocol.

| Abbreviation | Term |
|---|---|
| ACE | Angiotensin-Converting Enzyme |
| AE | Adverse Event |
| ALT | Alanine Aminotransferase |
| ANOVA | Analysis of Variance |
| API | Active Pharmaceutical Ingredient |
| AST | Aspartate Aminotransferase |
| AUC | Area Under the Concentration-Time Curve |
| $AUC_{0-24h}$ | Area Under the Concentration-Time Curve from 0 to 24 Hours |
| $AUEC_{0-4h}$ | Area Under the Effect-Time Curve of Change from Baseline |
| BID | Twice Daily |
| BMI | Body Mass Index |
| BP | Blood Pressure |
| BUN | Blood Urea Nitrogen |
| $C_{max}$ | Observed Maximum Plasma Concentration |
| $CE_{av}$ | Average Change from Baseline |
| $CE_{max}$ | Maximum Change from Baseline |
| CFDA | China Food and Drug Administration |
| CFR | Code of Federal Regulations |
| CI | Confidence Interval |
| CL/F | Apparent Total Plasma Clearance of Drug after Oral Administration |
| CNS | Central Nervous System |
| CRC | Clinical Research Center |
| CRO | Contract Research Organization |
| CYP | Cytochrome P450 |
| DDI | Drug Drug Interaction |
| DMP | Data Management Plan |
| DPP-4 | Dipeptidyl Peptidase-4 |
| ECG | Electrocardiography |
| eCRF | Electronic Case Report Form |
| eGFR | Estimated Glomerular Filtration Rate |
| EOS | End-of-Study |
| FDA | Food and Drug Administration |

| Abbreviation | Term |
|---|---|
| FSH | Follicle Stimulating Hormone |
| GCP | Good Clinical Practice |
| GGT | Gamma-Glutamyl-Transferase |
| GK | Glucokinase |
| GKA | Glucokinase Activator |
| GLP | Good Laboratory Practice |
| GLP-1 | Glucagon-Like Peptide 1 |
| GMP | Good Manufacturing Practice |
| GSIR | Glucose Stimulated Insulin Release |
| HbA1c | Hemoglobin A1c |
| HBsAg | Hepatitis B Surface Antigen |
| Hct | Hematocrit |
| HCV | Hepatitis C Virus |
| HDL | High-Density Lipoprotein |
| hERG | Human Ether-a-go-go-Related Gene |
| Hgb | Hemoglobin |
| HIV | Human Immunodeficiency Virus |
| HLM | Human Liver Microsome(s) |
| IB | Investigational Brochure |
| $IC_{50}$ | The Half Inhibitory Concentration |
| ICF | Informed Consent Form |
| ICH | International Conference on Harmonisation |
| IEC | Independent Ethics Committee |
| IND | Investigational New Drug Application |
| IRB | Institutional Review Board |
| IUD | Intrauterine Device |
| $K_{el}$ | Elimination Rate Constant |
| LC-MS/MS | Liquid Chromatography-Tandem Mass Spectrometry |
| LDH | Lactate Dehydrogenase |
| LDL | Low-Density Lipoprotein |
| MAD | Multiple Ascending Dose |
| MATE | Multidrug and Toxin Extrusion |
| MedDRA | Medical Dictionary for Regulatory Activities |
| MTD | Maximum Tolerated Dose |
| NOAEL | No Observed Adverse Effect Level |
| OAT | Organic Anion Transporter |

| **Abbreviation** | **Term** |
|---|---|
| OATP | Organic Anion Transporting Polypeptide |
| OGTT | Oral Glucose Tolerance Test |
| OTC | Over-The-Counter |
| P-gp | P-Glycoprotein |
| PHI | Protected Health Information |
| PIS | Patient Information Sheet |
| PK | Pharmacokinetic(s) |
| QC | Quality Control |
| QD | Once Daily |
| QTc | Corrected QT Interval |
| RBC | Red Blood Cell |
| $S_{0.5}$ | Substrate Concentration to Give ½ $V_{max}$ |
| SAD | Single Ascending Dose |
| SAE | Serious Adverse Event |
| SAP | Statistical Analysis Plan |
| SDV | Source Data Verification |
| SGLT2 | Sodium-Glucose Cotransporter-2 |
| SOP | Standard Operating Procedure |
| $T_{1/2}$ | Terminal Elimination Half-Life |
| T2DM | Type 2 Diabetes Mellitus |
| TBiL | Total Bilirubin |
| TDI | Time Dependent Inhibition |
| TEAE | Treatment-Emergent Adverse Event |
| $T_{max}$ | Time at which $C_{max}$ was first observed |
| ULN | Upper Limit of Normal |
| $V_{max}$ | Maximum Metabolic Rate |
| $V_z/F$ | Apparent Volume of Distribution during Terminal Phase after Oral Administration |
| WBC | White Blood Count |
| WHO | World Health Organization |

# 3. INTRODUCTION

## 3.1 Background Information

Hua Medicine (Shanghai) Ltd. (hereinafter "sponsor" or "Hua Medicine") is developing dorzagliatin, an investigational novel glucokinase activator (GKA), indicated for use as an adjunct to diet and exercise to improve glycemic control in adults with type 2 diabetes mellitus (T2DM).

Glucokinase (GK) activators represent a promising new class of investigational drugs for the treatment of T2DM. Glucokinase activators lower blood glucose levels by enhancing the ability of pancreatic β-cells to "sense glucose" and increase insulin secretion in a glucose dependent manner. Simultaneously, GKAs can suppress glucose production and increase glucose utilization in the liver. Glucokinase activators may also function through other GK-expressing cells, such as entero-endocrine K and L-cells, and many GKAs have been shown to exert anti-apoptotic effects on β-cells.

Dorzagliatin (also referred to as HMS5552) is the 4$^{th}$ generation of GKAs. Dorzagliatin is an allosteric activator of GK which has been shown to increase the affinity of its substrate glucose by decreasing $S_{0.5}$ and increasing the $V_{max}$ of GK. Dorzagliatin has only a minor effect on the Hill coefficient nH and preserves the positive cooperativity of GK for glucose, a unique kinetic feature of GK. Dorzagliatin enhances glucose stimulated insulin release (GSIR) in rodent pancreatic islets and increases glucose uptake in cultured rodent primary hepatocytes. The selectivity profile of dorzagliatin was evaluated by screening 402 Ambit protein kinases and 78 CEREP receptors. In all cases the $IC_{50}$ values were [redacted], which was the highest concentration tested.[1]

Dorzagliatin is a potent glucose lowering agent showing excellent dose-related effects on fasting, basal and post-prandial glucose levels in several rodent models of T2DM, both in acute as well as chronic studies. Dorzagliatin augments GSIR and improves hepatic glucose disposal *in vivo*. Furthermore, treatment of normal mice with dorzagliatin has been associated with an increase in the levels of total Glucagon-like Peptide-1 (GLP-1). Therefore, dorzagliatin is a potential new antidiabetic agent for use as an adjunct to diet and exercise to improve glycemic control in adults with T2DM.

## 3.2 Preclinical Data

Preclinical evaluations including safety pharmacology, general toxicology studies in rats and dogs, reproductive toxicology, genetic toxicology were conducted following the guidance of International Conference on Harmonisation (ICH) and China Food and Drug Administration (CFDA), in compliance with the U.S. and CFDA Good Laboratory Practices (GLP) regulations. [1]

No QTc prolongation or dorzagliatin-related qualitative electrocardiographic events or abnormalities were observed at dose level up to [redacted] of dorzagliatin in safety pharmacology studies using telemetry technology in conscious dogs. [redacted] No adverse effect on central nervous system or respiratory system was observed in rats at [redacted] dorzagliatin (maximum dose).[1]

The repeat-dose toxicity and toxicokinetic profiles for orally administered dorzagliatin have been characterized in rats for 4 weeks, 13 weeks and 26 weeks and in dogs for 4 weeks, 13 weeks, and 39 weeks. [redacted] No adverse effect (including peripheral neuropathy) was found in dogs after dorzagliatin treatment [redacted]

No adverse effect on male reproductive organs and reproductivity was found in male fertility study [redacted]

[redacted] In the later phase of clinical trials, exclusive criteria have included the subjects who might potentially be pregnant.

No evidence for genotoxicity or mutagenicity of dorzagliatin was identified using in vitro Ames test and chromosomal aberration assay in human peripheral blood lymphocytes, and in vivo micronucleus test in rat bone marrow.[1]

These study results indicate that dorzagliatin has an adequate safety margin to support its development in the clinical setting.

## 3.3 Summary of Clinical Studies

To date, five clinical studies evaluating dorzagliatin have been completed, four of which were Phase 1 studies and one was a Phase 2 study. A total of 335 subjects have been exposed to dorzagliatin.[1]

Study HMM0101: In a Phase 1 Single Ascending Dose (SAD) study, a total of 48 healthy subjects received single oral doses of HMS5552 ranging from 5 mg to 50 mg. HMS5552 appeared to be safe and was well tolerated at all doses studied. No serious adverse events (SAEs) were reported and no withdrawal occurred due to an adverse event (AE). All AEs were mild in intensity and no treatment was required. [redacted] No marked gender difference of $C_{max}$ and AUC was noticed, and the PK profile of HMS5552 indicated it was suitable for twice daily (BID) administration.

Study HMM0102: In a Phase 1 Multiple Ascending Dose (MAD) study, a total of 43 T2DM subjects received multiple twice-daily oral doses of HMS5552 ranging from 25 mg to 200 mg for 8 days. HMS5552 appeared to be safe and was well tolerated at all doses. There was no apparent sign or change in the pattern of any clinical laboratory value, vital signs or ECG parameters. All AEs were mild in intensity. No severe AE or SAE was reported. The most common AEs were related to mild hypoglycemia. No subjects reported serious hypoglycemia. All subjects recovered quickly without requiring additional intervention. The PK of HMS5552 appeared to be dose-proportional over the range tested without appreciable drug accumulation or food-effect. [redacted]

[redacted]

Study HMM0103: In a Phase 1c study, 24 T2DM subjects received daily doses of 75 mg or 150 mg (75 mg BID) HMS5552 daily for 4 weeks. HMS5552 displayed excellent safety and tolerability in subjects who participated in the study. There were no incidents of SAE, death, early termination due to AEs or severe hypoglycemia. All AEs were considered mild in intensity. No clinically significant changes were observed in laboratory, 12-lead electrocardiogram (ECG) or physical examination (PE) tests. Consistent PK Profiles were observed as in the MAD study.

[redacted]

renal excretion was not the major elimination pathway of HMS5552. [redacted]

Functions of pancreatic β-cells were improved in both groups 3 days after the 28-day treatment ended, compared with baseline. The sensitivity of pancreatic β-cells to blood glucose was enhanced after 7-8 drug $T_{1/2}$ had passed.

Study HMM0104: In a Phase 1 drug-drug interaction (DDI) study conducted in the US, 15 T2DM subjects received HMS5552 50 mg BID or metformin 500 mg BID alone or in combination for a total of 13 days (metformin alone for 3 days, dorzagliatin and metformin co-administration for 5 days, then dorzagliatin alone for 5 days). It appeared treatment with HMS5552 50 mg BID alone or co-administration with metformin 500 mg BID was safe and well-tolerated. There were no incidents of SAE, death, or early termination due to AEs. All treatment-emergent adverse events (TEAEs) were mild in intensity and subjects recovered after intervention. There was no clear evidence to support the causal relationship between the study drug and TEAEs, and no incidence of hypoglycemic events. No clinically significant changes were observed in laboratory tests, vital signs, ECGs or PEs in this study. Combined treatment

with metformin did not affect the PK of HMS5552. [redacted] Overall, the study demonstrated that there is no apparent clinically significant DDI between HMS5552 and metformin. In contrast, the combined treatment resulted in improved glycemic control in T2DM subjects compared to treatment with HMS5552 or metformin alone.

Study HMM0201: A dose-ranging, randomized, double-blind, placebo-controlled, Phase 2 study was conducted in Chinese patients with T2DM, aiming to identify a minimum effective dose of dorzagliatin in patients with type 2 diabetes.[1,2]

Two hundred fifty-eight (258) T2DM patients enrolled in the Phase 2 study and were randomly assigned to receive placebo (n=53), 75 mg dorzagliatin once daily (n=53), 100 mg dorzagliatin once daily (n=50), 50 mg dorzagliatin twice daily (n=51), or 75 mg dorzagliatin twice daily (n=51) for 12 weeks.

No deaths, drug-related SAEs, or drug-related severe AEs were reported. Most AEs were mild and considered unrelated to study medication by investigators. The incidence of AEs was similar among groups. Adverse events that occurred in ≥5% of patients in any group (including placebo) were upper respiratory tract infection, hyperuricemia, dizziness, protein present in urine, urinary tract infection, blood creatine phosphokinase increased, white blood cells (WBC) urine positive, hepatic function abnormal, high-density lipoprotein (HDL) decreased, ventricular extrasystole and nasopharyngitis. Incidence of hypoglycemia was low, with a rate of 5.4% for ≤3.9 mmol/L, and a rate of only 1% for ≥3.0 mmol/L in the Phase 2 study in the drug-treated groups. No severe hypoglycemia was reported.

[redacted]

In conclusion, preclinical pharmacology studies demonstrated that HMS5552 is an effective allosteric activator of GK both *in vitro* and *in vivo*. Safety pharmacology, general toxicity studies

in rats and dogs up to 26 weeks and 39 weeks, reproductive toxicology, genetic toxicology and carcinogenicity studies suggested that HMS5552 has an acceptable safety profile when projected for human use. Furthermore, safety data from clinical phase 1 and phase 2 studies conducted in China and US support the continued development of HMS5552.

## 3.4 Drug Interaction Potential of Dorzagliatin

In *in vitro* studies using human liver microsomes (HLM) dorzagliatin showed no inhibition of liver cytochrome P450 (CYP) 1A2, CYP2A6, CYP2C9, CYP2C19, CYP2D6, CYP2E1 and CYP3A4/5 activities ($IC_{50}$ >50 µM) at concentrations studied.

Drug interaction potential of dorzagliatin via CYP induction was evaluated in primary human hepatocytes and results indicated that dorzagliatin was unlikely to produce induction on CYP1A2 or CYP2B6. While an absence of induction of dorzagliatin on CYP3A4 could not be excluded from this vitro study, it was later confirmed in preclinical and clinical PK studies as no decrease in exposure of dorzagliatin was observed after repeated dosing.

*In vitro* results suggest that dorzagliatin is a substrate of P-glycoprotein (P-gp) but not a P-gp inhibitor. In addition, dorzagliatin did not show clinically relevant inhibitory effect on organic anion transporting polypeptide (OATP)1B1, OATP1B3, organic anion transporter (OAT)1 and OAT3, nor is dorzagliatin a substrate of these four transporters. These study results demonstrate that transporter-related DDI is unlikely.

Since dorzagliatin is predominantly metabolized by CPY3A4 and is a substrate of P-gp, it is predicted that exposure of dorzagliatin will be increased when co-administered with CYP3A4 inhibitors and decreased when co-administered with CYP3A4 inducers.[1]

Dipeptidyl peptidase-4 (DPP-4) inhibitors are a class of oral drugs for the treatment of patients with T2DM who have not responded well to drugs such as metformin and sulfonylureas. DPP-4 inhibitors block the action of DPP-4, an enzyme that destroys incretins. Incretins are gastrointestinal hormones that help stimulate insulin production. FDA-approved DPP-4 inhibitors include sitagliptin [redacted], saxagliptin [redacted] and linagliptin [redacted]. The reported half-lives of sitagliptin, saxagliptin (and active metabolite) and linagliptin are 12.4, 2.5 (and 3.1), and 12 hours (effective half-life) respectively.[3,4,5]

Sitagliptin is mainly (79%) cleared as parent drug by urine with metabolism being a minor pathway of elimination. In vitro studies indicated that the primary enzyme responsible for the limited metabolism of sitagliptin was CYP3A4, with additional contribution from CYP2C8. Sitagliptin is a substrate for OAT 3, which may be involved in the renal elimination of sitagliptin.

Sitagliptin is not an inhibitor of CYP3A4, 2C8, 2C9, 2D6, 1A2, 2C19 or 2B6, and is not an inducer of CYP3A4. Sitagliptin is a P-gp substrate but does not inhibit P-gp mediated transport of digoxin. Based on these results sitagliptin is considered unlikely to cause interactions with other drugs that utilize these pathways. Sitagliptin is not extensively bound to plasma proteins. Therefore, the propensity of sitagliptin to be involved in clinically meaningful DDIs mediated by plasma protein binding displacement is very low.[3]

Collectively, the available PK and metabolic information for dorzagliatin and sitagliptin summarized above suggest that the DDI potential between these two drugs when co-administered is low.

In this DDI study, prior to enrollment subjects must be taking a stable dose of either metformin alone, metformin in combination with a marketed brand of a sodium-glucose cotransporter-2 (SGLT2) inhibitor or a DPP-4 inhibitor, or a marketed brand of an SGLT2 or DPP-4 inhibitor as monotherapy.

Metformin [redacted] is an oral antihyperglycemic drug that is commonly recommended as the first-line pharmacotherapy for treatment of T2DM. Mean time to peak plasma concentration ($T_{max}$) following administration of a metformin tablet is about 2-3 hours. Renal excretion is the major route of metformin elimination. Following oral administration, approximately 90% of the absorbed drug is eliminated via the renal route within the first 24 hours, with a plasma elimination half-life of approximately 6.2 hours.[6]

Sodium-glucose cotransporter-2 inhibitors are a class of oral drugs for the treatment of patients with T2DM that lower blood sugar by blocking SGLT2, a glucose transporter in the kidney, and preventing the kidney from reabsorbing glucose and releasing it into the blood. FDA-approved SGLT2 inhibitors include canagliflozin [redacted], dapagliflozin [redacted] and empagliflozin [redacted]. The reported half-lives of canagliflozin, dapagliflozin and empagliflozin are 10-13.1, 12.9 and 12.4 hours, respectively.[7,8,9]

# 4. STUDY RATIONALE AND OBJECTIVES

## 4.1. Study Rationale

Although the available drug interaction and PK data of dorzagliatin and sitagliptin detailed in Section 3.4 do not suggest a potential drug interaction, other drug transporter-mediated interactions cannot be ruled out. Furthermore, pharmacodynamic-based interaction potential needs to be evaluated. Based on the high likelihood that dorzagliatin may be co-administered with sitagliptin in T2DM patients in a clinical setting, this study will provide clinical evidence of the DDI potential between these two drugs.

The basis for the 100 mg QD dosing regimen of sitagliptin is based on the recommended 100 mg once daily dosing of [redacted] for adults.[3] The 75 mg BID for dorzagliatin is the dose regimen proven to be safe and effective in Phase 1 and Phase 2 studies and selected for two current Phase 3 studies evaluating dorzagliatin as a mono-therapy and as an add-on treatment to metformin. In the present study, both drugs will be given for five days to ensure that steady-state is attained.

The sequential, multiple-dose study is designed to determine whether the steady-state pharmacokinetics of dorzagliatin and sitagliptin are affected, while at the same time whether there's a synergistic therapeutic effect between these two drugs when co-administered. The proposed dosing regimens with dorzagliatin and sitagliptin will allow the potential interactions to be assessed under conditions that are expected to provide maximal exposure at the doses being studied. The elimination half-lives of dorzagliatin and sitagliptin are relatively short which justifies the 24-hour interval for collecting all samples to characterize the pharmacokinetics of both drugs.

## 4.2. Primary Objectives

The primary objectives of this study are:

- To assess the potential pharmacokinetic interaction between dorzagliatin and sitagliptin in subjects with type 2 diabetes mellitus;
- To evaluate the safety and tolerability of dorzagliatin with simultaneous administration of sitagliptin in subjects with type 2 diabetes mellitus.

### 4.3. Secondary Objective

The secondary objective of this study is:

- To assess the pharmacodynamic responses of glucose, GLP-1, glucagon, insulin, and C-peptide following dorzagliatin, sitagliptin, or simultaneous administration of dorzagliatin and sitagliptin in subjects with type 2 diabetes mellitus.

# 5. INVESTIGATIONAL PLAN

## 5.1. Overall Study Design

This is a Phase 1, open-label, sequential, multiple-dose, DDI study of GK activator dorzagliatin and sitagliptin in subjects with T2DM.

It is planned that 15 subjects will be enrolled to have at least 12 evaluable subjects. All subjects will receive:

- Sitagliptin 100 mg QD in the morning on Days 1-5;
- Sitagliptin 100 mg QD in the morning and dorzagliatin 75 mg BID (morning and evening) on Days 6-10, with only the morning dose on Day 10;
- Dorzagliatin 75 mg BID (morning and evening) on Days 11-15, with only the morning dose on Day 15.

Eligible subjects will have a minimum 12-day run-in period prior to admission to the CRC, during which time they will self-administer sitagliptin 100 mg QD up until and including Day -2. Sitagliptin will be dispensed by the CRC. Sitagliptin will be purchased and provided by the sponsor. Subjects will complete a diary to record sitagliptin doses taken each day and will be advised to monitor their blood glucose levels and report changes in health status to the CRC. A telephone call will be placed to all subjects at approximately Day 6 (±2 day) of the run-in period to assess general health and collect AE information.

Following completion of the run-in period, eligible subjects will be admitted to the CRC on Day -2, have a total of 18 overnight stays, and be discharged after End-of-Study procedures are completed on Day 17, or at early termination.

Blood samples for the determination of dorzagliatin and sitagliptin concentrations and corresponding PK analysis will be collected at the following time points on Days 5, 10 and 15: [redacted].

Subjects will consume a 75-gram glucose solution 30 minutes following study drug dose administration on Days 5, 10 and 15, in lieu of a breakfast. Pharmacodynamic responses will be evaluated by measuring GLP-1 and glucagon in plasma, and glucose, insulin, and C-peptide in serum at baseline (prior to the study drug dose and within 60 minutes prior to oral glucose intake) and 0.5, 1, 1.5, 2, 2.5, 3 and 4 hours after oral glucose intake.

Safety assessments will include monitoring of AEs, blood glucose via glucometer readings, vital signs (blood pressure (BP), pulse rate (PR), respiratory rate (RR) and oral temperature (T)), clinical laboratory findings, resting ECGs, and PE findings.

Vital signs will be assessed at screening, on Day -1, on Days 1-15 within 60 minutes prior to each morning and evening dose, and on Day 17. Blood glucose will be measured using a glucometer three times each day on Days -2 (prior to first meal after admission) through Day 16, within 60 minutes before each meal and after study drug dosing when applicable. Clinical laboratory evaluations (chemistry, hematology, urinalysis) will be performed at screening, on Days -1, 5, 10, 15 and 17. A resting 12-lead ECG will be completed at screening, on Day -1, and 2 hours post-dose on Days 5, 10, 15, and 17. Physical exams will be conducted at screening, on Day -1, and on Day 17.

Subjects who receive their Day 1 dose and then terminate study participation early will have Day 17 assessments performed, if possible, prior to discharge from the CRC.

Morning dosing will occur at approximately 08:00 a.m. and evening dosing will occur at approximately 06:00 p.m. All doses will be administered 60 (±5) minutes prior to a standardized meal and with approximately 240 mL (8 fluid ounces) of room temperature water. On Days 5, 10 and 15 there will be no meal offered after the morning dose and instead the oral glucose solution will be administered 30 minutes after the study drug dose. The actual time of each dose, each post-dose meal, and time of oral glucose solution administration will be recorded.

See Table 5-1 for the details of all study procedures for subjects in the study.

## Table 5-1 Schedule of Procedures

| Visit | Screening | Run-in* | In-patient at the CRC* | | | | | | | | | |
|---|---|---|---|---|---|---|---|---|---|---|---|---|
| **Day** | **-42 to -15** | **-14 to -3** | **-2** | **-1** | **1-4** | **5** | **6-9** | **10** | **11-14** | **15** | **16** | **17 EOS** |
| Informed consent | X | | | | | | | | | | | |
| Dispense/collect sitagliptin, at-home sitagliptin QD 100 mg. Tel call at Day -8±2 (Day 6 of the 12-day run-in period)* | | X | X | | | | | | | | | |
| Admission to CRC | | | X | | | | | | | | | |
| Eligibility assessment | X | | | X | | | | | | | | |
| Demographics | X | | | | | | | | | | | |
| Medical history | X | | X | | | | | | | | | |
| Physical examination | X | | | X | | | | | | | | X |
| Height (cm), Weight (kg) | X | | | X[1] | | | | | | | | X[1] |
| Body mass index | X | | | | | | | | | | | |
| Standard 12-lead ECG[2] | X | | | X | | X | | X | | X | | X |
| Vital signs (BP, PR, RR, T) | X | | | X | Within 60 min prior to each study drug dose. | | | | | | | X |
| Clinical laboratory samples[3] | X | | | X | | X | | X | | X | | X |
| HIV, hepatitis B & C | X | | | | | | | | | | | |
| Urine drug /saliva alcohol | X | | X | | | | | | | | | |
| Urine pregnancy test[4] | X | | X | | | | | | | | | X |
| FSH and estradiol[4] | X | | | | | | | | | | | |
| OGTT/PD markers [5] | | | | | | X | | X | | X | | |
| C-peptide and HbA1c | X | | | | | | | | | | | |
| Dose administration[6] | | | | Sitagliptin 100 mg QD on Day -1; Assigned treatment Days 1-15 | | | | | | | | |
| AE & con med reporting[7] | | X | X | X | X | X | X | X | X | X | X | X |
| PK blood samples[8] | | | | | | X | X | X | X | X | X | |
| Glucometer (finger stick) | | | Within 60 minutes before each meal and after study drug dosing when applicable | | | | | | | | | |
| Discharge from CRC | | | | | | | | | | | | X |

AE= adverse event; BP = blood pressure; con med = concomitant medications; CRC = clinical research center; ECG = electrocardiogram; EOS =End-of-Study; FSH: Follicle stimulating hormone; HIV = human immunodeficiency virus; PR = pulse rate; PK = pharmacokinetics; RR = respiratory rate; T = temperature.

*Run-in period is a minimum of 12 days. If longer than 12 days, admission to CRC remains on Day -2. Sitagliptin will be dispensed by CRC to eligible subjects any day prior to start of Day -14 at-home dosing.

[1]Obtain weight only.

[2]ECG obtained in supine position after at least 5 minutes rest. On Days 5, 10 and 15 ECGs obtained 2 hours (±15 minutes) after dose administration.

[3]Clinical laboratory samples include: hematology, chemistry and urinalysis. All samples collected prior to study drug dose on days of study drug administration.

[4]For female subjects, urine pregnancy test must be negative to enroll in the study. Serum FSH and estradiol will be evaluated for postmenopausal females to confirm status.

[5] Oral glucose solution administered 30 (±5) minutes after study drug dose on Days 5, 10 and 15. PD samples to measure GLP-1 and glucagon in plasma, and glucose, insulin, and C-peptide in serum collected at baseline (prior to the study drug dose and within 60 minutes prior to oral glucose intake) and at 0.5, 1, 1.5, 2, 2.5, 3 and 4 hours after oral glucose intake. All post-glucose collection times are ±5 minutes from nominal time.

[6]All subjects will receive: sitagliptin 100 mg QD on Days 1-5 (a.m. dose); sitagliptin 100 mg QD and dorzagliatin 75 mg BID on Days 6-10 (a.m. dose only on Day 10); and dorzagliatin 75 mg BID on Days 11-15 (a.m. dose only on Day 15). Morning dosing will occur at approximately 08:00 a.m. and evening dosing will occur at approximately 06:00 p.m. All doses are 60 (±5) minutes prior to meals except for Days 5, 10 and 15 morning doses when no meals are offered following the study drug dose. Subjects are to resume their regular medication on Day 16.

[7]SAE collection starts after signing ICF.

[8]PK samples collected [redacted] on Days 5, 10 and 15. Blood collection time windows: ±5 minutes from 0-4 hours post-dose and ±10 minutes ≥6 hours post-dose.

## 5.2. Number of Subjects

This study will plan to enroll up to 15 eligible subjects. Subjects may be enrolled singly or in groups. In order to have at least 12 evaluable subjects complete the study, additional subjects may be enrolled to replace subjects who discontinue study participation prematurely. Evaluable subjects are defined as subjects included in the DDI population as described in Section 12.2.

## 5.3. Treatment Assignment

All subjects will receive the same treatment assignment as follows:

**Table 5-2: Treatment Assignment and PK Sample Collection**

| Day | Sitagliptin | Dorzagliatin | PK Sample Collection |
|---|---|---|---|
| 1-5 | 100 mg (QD) for 5 days | - | Day 5:<br>Sitagliptin alone |
| 6-10 | 100 mg (QD) for 5 days | 75 mg (BID) for 4 days<br>Morning dose only on Day 10 | Day 10:<br>Sitagliptin + Dorzagliatin |
| 11-15 | - | 75 mg (BID) for 4 days<br>Morning dose only on Day 15 | Day 15:<br>Dorzagliatin alone |

## 5.4. Criteria for Study Termination

The Investigator has the right to terminate the study in the interest of subject safety and welfare in consultation with sponsor. Additionally, the sponsor reserves the right to terminate or amend the study at any time for administrative reasons or if continuation of the protocol would present a potential safety risk to the subjects.

# 6. SELECTION AND WITHDRAWAL OF SUBJECTS

## 6.1. Subject Inclusion Criteria

To participate in the study, subjects must meet all the following eligibility criteria:

1. Subjects diagnosed with T2DM within at least 3 months prior to screening and are currently taking a stable dose of either of the following therapy with no change in the dose for at least 4 weeks prior to screening:
    a. ≥1000 mg per day of metformin

   b. a dipeptidyl peptidase 4 (DPP-4) inhibitor
   c. a sodium-glucose cotransporter-2 (SGLT2) inhibitor
   d. metformin plus a DPP-4 inhibitor
   e. metformin plus a SGLT2 inhibitor
2. Subjects must agree to change their current therapy to 100 mg sitagliptin QD for at least 14 days prior to dosing on Day 1;
3. In stable general health as determined by the Investigator at screening evaluation performed no more than 42 days prior to dosing on Day 1;
4. Male and/or female subjects between the ages of 30 and 65 years, inclusive;
5. Are capable of giving informed consent and complying with study procedures;
6. Body Mass Index (BMI) of 19 to 38 $kg/m^2$, inclusive, at screening;
7. Fasting C-peptide test result >0.3 nmol/L (>0.90 ng/mL);
8. HbA1c ≥7% and ≤10.5%;
9. Females must be using one of the following birth control methods: a non-hormonal intrauterine device (IUD) in place for at least 3 months; barrier method (condom, diaphragm) with spermicide for at least 14 days prior to dosing on Day 1 and throughout the study; surgical sterilization of the partner (vasectomy for 6 months minimum); OR are surgically sterile OR postmenopausal, as defined below:
   a. Surgically sterile for at least 3 months prior to screening by one of the following means:
      - Bilateral tubal ligation
      - Salpingectomy (with or without oophorectomy)
      - Surgical hysterectomy
      - Bilateral oophorectomy (with or without hysterectomy)
   b. Postmenopausal, defined as following:
      - Last menstrual period greater than 12 months prior to screening, confirmed by FSH and estradiol levels at screening;
10. Nonsmoker, defined as not having smoked or used any form of tobacco for more than 6 months prior to screening;
11. Willing and able to adhere to study restrictions and to be confined at the clinical research center.

## 6.2. Subject Exclusion Criteria

Subjects will be excluded from study entry if any of the following exclusion criteria are present:

1. Fasting blood glucose at screening or Day -1 ≤110 or ≥270 mg/dL (one repeat screening fasting blood glucose allowed);
2. Type 1 diabetes mellitus, or latent autoimmune diabetes in adults; diabetic neuropathy, retinopathy or nephropathy;
3. Reported incidence of severe hypoglycemia, defined by denoting severe cognitive impairment requiring external assistance for recovery, within 3 months prior to screening and/or within 3 months prior to dosing on Day 1;
4. Known contraindications to sitagliptin;
5. Clinically significant gastrointestinal disorder (e.g., gastroparesis, constipation, diarrhea, colitis, gastrointestinal tract obstruction, hiatal hernia with reflux esophagitis, or cholestasis);
6. History or symptoms of clinically significant cardiovascular disease, particularly coronary artery disease, arrhythmias, atrial tachycardia, or congestive heart disease within one year prior to screening;
7. History of more than three urinary tract infections and/or more than three genital fungal infections in the last 12 months. If one or more genital fungal infections in the last 12 months required systemic treatment to resolve, Investigator will determine eligibility based on treatment required;
8. Reported history of clinically significant central nervous system disease including: transient ischemic attack, stroke, seizure disorder, depression, or behavioral disturbances within one year prior to screening;
9. Reported history of liver disease (e.g., hepatitis, cirrhosis within one year of screening and/or liver enzymes (alanine aminotransferase (ALT), aspartate aminotransferase (AST), gamma-glutamyltransferase (GGT), total bilirubin (TBiL)) >2 times the upper limit of normal (ULN));
10. Reported history of clinically significant renal disease;
11. Estimated glomerular filtration rate (eGFR) ≤60 mL/min/1.73m$^2$;
12. Acute or chronic metabolic acidosis, including diabetic ketoacidosis;
13. Known or suspected malignancy;

14. Any reported hypersensitivity (anaphylaxis and angioedema) or intolerance to sitagliptin;
15. Antidiabetic treatment with insulin, sulfonylureas, thiazolidinediones or GLP-1 agonist within 3 months prior to screening;
16. Systolic blood pressure <90 or ≥160 mmHg or diastolic blood pressure <60 or ≥100 mmHg at screening (may be repeated once);
17. A hospital admission or major surgery within 90 days prior to screening;
18. Uncontrolled hypertriglyceridemia >500 mg/dL;
19. Positive blood screen for human immunodeficiency virus (HIV), hepatitis B surface antigen (HBsAg), or hepatitis C antibody;
20. Positive pregnancy test result;
21. Female is breast-feeding or planning to become pregnant;
22. Treated with any investigational drugs within 6 weeks prior to screening;
23. Reported history of prescription drug abuse, or illicit drug use within 6 months prior to screening;
24. Reported history of alcohol abuse according to medical history within 6 months prior to screening;
25. A positive screen for alcohol or drugs of abuse;
26. Reported history of donation or blood collection of more than 1 unit (approximate 450 mL) of blood (or blood products) or acute loss of blood during the 90 days prior to screening;
27. Use of prescription or over-the-counter (OTC) medications, and herbal (including St John's Wort, herbal teas, garlic extracts) within 14 days prior to dosing (except for sitagliptin dosing during run-in period). No angiotensin-converting enzyme (ACE) inhibitors are allowed; (Note: Use of acetaminophen at <1 g/day is permitted until 24 hours prior to dosing);
28. An unwillingness of male participants to use appropriate contraceptive measures if engaging in sexual intercourse with a female partner of childbearing potential. Appropriate measures include use of a condom and spermicide and, for female partners, use of an IUD, diaphragm with spermicide, oral contraceptives, injectable progesterone, progesterone subdermal implants, or a tubal ligation. Sexual intercourse with pregnant or lactating women is prohibited.

## 6.3. Prohibitions and Restrictions

Subjects must be willing to adhere to the following prohibitions and restrictions from times noted and for the duration of the study:

- No illicit drug use, abuse of alcohol and use of tobacco-containing products within 6 months prior to screening;
- No consumption of alcohol or food containing alcohol;
- No consumption of food or drinks containing caffeine;
- No consumption of grapefruit juices;
- No administration of any prescription medications (with the exception of study drug) per Exclusion Criteria #27;
- No use of any OTC products (with the exception of acetaminophen <1 g/day until 24 hours prior to dosing) per Exclusion Criteria #27;
- Subjects must remain upright for 4 hours following administration of study drug (except during measurement of vital signs and ECGs) following the morning dose on Days 5, 10 and 15.

## 6.4. Subject Withdraw Criteria

Subjects will be informed that they have the right to withdraw from the study at any time for any reason without prejudice to their medical care. The Investigator also has the right to withdraw subjects from the study if it is in the best interest of the subject, or if the subject experiences an adverse event that warrants premature withdrawal.

Subjects may be withdrawn from the study if they:

- Have entered the study in violation of the protocol
- Use or require the use of prohibited concomitant medication(s)
- Are non-compliant with study procedures
- Experience an AE that warrants premature withdrawal

Subjects who experience emesis after dosing are not required to withdraw from the study. The event and time of emesis should be documented, and the subject permitted to continue at the discretion of the Investigator.

All treated subjects should be followed according to the Schedule of Procedures (Table 5-1). All subjects who receive a Day 1 dose, even those who have discontinued prematurely, should have all evaluations for the Day 17 End-of-Study visit performed, if possible. All procedures should be documented in the electronic case report form (eCRF). For all subjects who withdraw prematurely, the Investigator will indicate one of the following reasons for withdrawal in the eCRF:

- Adverse event
- Death
- Protocol violation
- Lost to follow-up
- Subject's decision
- Other (reason to be specified by the Investigator)

In case of subjects' discontinuation from the study due to an AE, such subjects will be closely monitored until the resolution or stabilization of the AE. The Investigator should document the reason for discontinuation in the source documentation and eCRF.

In the event that a subject withdraws participation from the study early, early withdrawal should be documented by the Investigator (or designee) in the appropriate eCRF pages and source documents when confirmed. The Day 17 End-of-Study assessments should be performed when a subject is discontinued.

# 7. STUDY PROCEDURES

Morning dosing will occur at approximately 08:00 a.m. and the evening dosing will occur at approximately 06:00 p.m. All doses will be administered 60 (±5) minutes prior to meals (except for on Days 5, 10 and 15 when meals are not offered after the morning dose) with about 240 mL (8 fluid ounces) of room temperature water. Actual times for each dose and start times for meals as well as oral glucose intake on days of dosing will be recorded. Standardized meals that are consistent with general dietary recommendations for diabetes will be provided while subjects are staying at CRC.

Eligible subjects will have a minimum 12-day run-in period prior to admission to the CRC on Day -2, during which time they will self-administer sitagliptin 100 mg QD each morning. Subjects will complete a diary to record sitagliptin doses taken each day and will be advised to monitor their blood glucose levels and report changes in health status to the CRC. A telephone call will be placed to all subjects at approximately Day 6 (±2 day) of the run-in period to assess general health and collect AE information. Subjects will be admitted to the CRC on Day -2 and remain sequestered at the study site until after all End-of-Study procedures are completed on Day 17 or at early termination.

The following sections describe in detail all study procedures. The schedule of procedures is presented in Table 5-1.

## 7.1. Screening Visit and Run-in Period

Subjects will report to the CRC for a screening outpatient visit between Day -42 and Day -15 relative to the Day 1 dosing day.

As outlined in Section 15.3, prior to the performance of any study-related activities or evaluations, the subject must be thoroughly informed about all aspects of the study, including scheduled visits and activities. Each subject will sign the study-specific consent form prior to any screening procedures. A signed copy of the informed consent form (ICF) will be given to each consenting subject and another signed copy will be retained in the subject's study records.

The following information and procedures will be performed and documented as part of the screening assessment:

- Collection of demographic information, including sex, race, ethnic origin, date of birth.
- Medical history, including review of prior and ongoing medications taken in previous 30 days, except for therapy to treat diabetes, which must be recorded for previous 3 months.
- Height and weight measurement and BMI calculation.
- Urine sample collection for urinalysis and test for drugs of abuse.
- Urine sample pregnancy test for women.
- Blood test for evaluation of FSH and estradiol for postmenopausal women.
- Saliva sample collection to test use of alcohol.
- Standard 12-lead ECG after resting supine for at least 5 minutes.

- Vital signs (BP, PR, RR and oral T) measurement after resting supine for at least 5 minutes.
- Blood sample collection for clinical chemistry, hematology, HIV, and hepatitis B and C evaluations, C-peptide measurement and HbA1c test.
- Physical examination.
- Assessment of eligibility according to inclusion/exclusion criteria.

Compliance with inclusion criteria (listed in Section 6.1) and exclusion criteria (listed in Section 6.2) will be verified against information collected and documented in the source documents and the eCRF. Laboratory results obtained at screening and on Day -1 will be used to verify eligibility.

Eligible subjects will be scheduled for a visit to the CRC for dispensing of sitagliptin 100 mg and start their 12-day run-in period. Subjects will be instructed to take a single dose of sitagliptin each morning, and to record each dose in a diary that will be provided by the CRC. Subjects will be advised to monitor their blood glucose levels and report changes in health status to the CRC. A telephone call will be placed to all subjects at approximately Day 6 (±2 day) of the run-in period to assess general health and collect AE information. Any signs or symptoms reported by subjects that develop or worsen compared to the subject's medical history after the first dose of sitagliptin in the run-in period will be recorded as AEs and will be followed by the Investigator. Additionally, subjects will be advised to contact their primary care physician.

An appointment will be scheduled for subjects to be admitted to the CRC on Day -2. Subjects will be advised to take their dose of sitagliptin at home prior to admission, and to bring remaining drug and their diary with them to the CRC on Day -2.

## 7.2. Domiciled at CRC on Day -2 through Day 17

### 7.2.1. Study Check-in Visit: Day -2 and Day -1

Subjects will be admitted to the CRC on Day -2 and remain for 18 overnight stays until completion of all study procedures on Day 17 or early termination.

The following assessments will be performed on Day -2:

- Collection of remaining sitagliptin and review of diary to confirm compliance with daily dosing of sitagliptin during the run-in period. Subjects with <90% compliance or compliance recorded as >110% are not eligible to continue study participation.
- Update of medical history, assessment of AEs, and medication use since the screening visit.
- Urine sample testing for drugs of abuse and pregnancy, as applicable.
- Alcohol saliva test.
- Assess blood glucose using a glucometer within 60 minutes before each meal and after study drug dosing when applicable.

The following assessments will be performed on Day -1:

- Blood and urine sample collection for clinical chemistry, hematology and urinalysis in the morning under fasting condition (after at least a 10-hour fast).
- Physical examination.
- Vital signs (BP, PR, RR and oral T) measurement after resting supine for at least 5 minutes.
- Measure weight
- Standard 12-lead ECG after resting supine for at least 5 minutes.
- Subjects will be administered sitagliptin 100 mg by the clinic staff in the morning at approximately 08:00 a.m. prior to a standardized meal.
- Assess blood glucose using a glucometer within 60 minutes before each meal and after study drug dosing when applicable.
- Confirm fasting blood glucose on Day -1 >110 and <270 mg/dL.
- Confirmation of eligibility according to inclusion/exclusion criteria.
- Assessment of AEs and concomitant medications.

### 7.2.2. Day 1 - Day 4

- Vital signs (BP, PR, RR and oral T) measurement after resting supine for at least 5 minutes within 60 minutes before each study drug dose.
- Administer 100 mg sitagliptin QD at 08:00 a.m. (±5 minutes), 60 (±5) minutes prior to a standardized meal.

- Assess blood glucose using a glucometer within 60 minutes before each meal and after study drug dosing when applicable.
- Assessment of AEs and concomitant medications.

### 7.2.3. Day 5

- Blood and urine sample collection for clinical chemistry, hematology and urinalysis in the morning under fasting condition (after at least a 10-hour fast) and before study drug dose.
- Vital signs (BP, PR, RR and oral T) measurement after resting supine for at least 5 minutes within 60 minutes before study drug dose.
- Administer 100 mg sitagliptin at 08:00 a.m. (±5 minutes). Subjects must remain upright for at least 4 hours following dosing except for measurement of vital signs or ECGs.
- Pharmacokinetic blood sample collections for the measurement of sitagliptin concentrations [redacted].
- Consume a 75-gram glucose solution 30 (±5) minutes following study drug dose in lieu of a breakfast.
- Pharmacodynamic blood sample collections to measure GLP-1 and glucagon in plasma, and glucose, insulin, and C-peptide in serum at baseline (prior to study drug dose and within 60 minutes prior to oral glucose intake) and 0.5, 1, 1.5, 2, 2.5, 3 and 4 hours after oral glucose intake.
- Assess blood glucose using a glucometer within 60 minutes before each meal and after study drug dosing when applicable.
- Standard 12-lead ECG after resting supine for at least 5 minutes, taken 2 hours (±15 minutes) after study drug dose.
- Assessment of AEs and concomitant medications.

### 7.2.4. Day 6 - Day 9

- Pharmacokinetic blood sample collections for the measurement of sitagliptin concentrations 18 and 24 hours post-dose (Day 6).

- Vital signs (BP, PR, RR and oral T) measurement after resting supine for at least 5 minutes within 60 minutes before each study drug dose.
- Administer 100 mg sitagliptin QD at 08:00 a.m. (±5 minutes) and 75 mg dorzagliatin BID (08:00 a.m., 06:00 p.m. (±5 minutes), each dose 60 (±5) minutes prior to a standardized meal.
- Assess blood glucose using a glucometer within 60 minutes before each meal and after study drug dosing when applicable.
- Assessment of AEs and concomitant medications.

### 7.2.5. Day 10

- Blood and urine sample collection for clinical chemistry, hematology and urinalysis in the morning under fasting condition (after at least a 10-hour fast) and before study drug dose.
- Vital signs (BP, PR, RR and oral T) measurement after resting supine for at least 5 minutes within 60 minutes before each study drug dose.
- Administer 100 mg sitagliptin and 75 mg dorzagliatin at 08:00 a.m. (±5 minutes). Subjects must remain upright for at least 4 hours following dosing except for measurement of vital signs or ECGs.
- Pharmacokinetic blood sample collections for the measurement of sitagliptin and dorzagliatin concentrations [redacted].
- Consume a 75-gram glucose solution 30 (±5) minutes following study drug dose in lieu of a breakfast.
- Pharmacodynamic blood sample collections to measure GLP-1 and glucagon in plasma, and glucose, insulin, and C-peptide in serum at baseline (prior to study drug dose and within 60 minutes prior to oral glucose intake) and 0.5, 1, 1.5, 2, 2.5, 3 and 4 hours after oral glucose intake.
- Assess blood glucose using a glucometer within 60 minutes before each meal and after study drug dosing when applicable.
- Standard 12-lead ECG after resting supine for at least 5 minutes, taken 2 hours (±15 minutes) after study drug dose.

- Assessment of AEs and concomitant medications.

### 7.2.6. Day 11 - Day 14

- Pharmacokinetic blood sample collections for the measurement of sitagliptin and dorzagliatin concentrations 18 and 24 hours post-dose (Day 11).
- Vital signs (BP, PR, RR and oral T) measurement after resting supine for at least 5 minutes within 60 minutes before each study drug dose.
- Administer 75 mg dorzagliatin BID (08:00 a.m., 06:00 p.m. (±5 minutes) 60 (±5) minutes prior to a standardized meal.
- Assess blood glucose using a glucometer within 60 minutes before each meal and after study drug dosing when applicable.
- Assessment of AEs and concomitant medications.

### 7.2.7. Day 15

- Blood and urine sample collection for clinical chemistry, hematology and urinalysis in the morning under fasting condition (after at least a 10-hour fast) and before each study drug dose.
- Vital signs (BP, PR, RR and oral T) measurement after resting supine for at least 5 minutes within 60 minutes before each study drug dose.
- Administer 75 mg dorzagliatin at 08:00 a.m. (±5 minutes). Subjects must remain upright for at least 4 hours following dosing except for measurement of vital signs or ECGs.
- Pharmacokinetic blood sample collections for the measurement of dorzagliatin concentrations [redacted].
- Consume a 75-gram glucose solution 30 (±5) minutes following study drug dose in lieu of a breakfast.
- Pharmacodynamic blood sample collections to measure GLP-1 and glucagon in plasma, and glucose, insulin, and C-peptide in serum at baseline (prior to study drug dose and within 60 minutes prior to oral glucose intake) and 0.5, 1, 1.5, 2, 2.5, 3 and 4 hours after oral glucose intake.

- Assess blood glucose using a glucometer within 60 minutes before each meal and after study drug dosing when applicable.
- Standard 12-lead ECG after resting supine for at least 5 minutes, taken 2 hours (±15 minutes) after study drug dose.
- Assessment of AEs and concomitant medications.

#### 7.2.8. Day 16

- Pharmacokinetic blood sample collections for the measurement of dorzagliatin concentrations 18 and 24 hours post-dose.
- Assess blood glucose using a glucometer within 60 minutes before each meal.
- Subjects will resume their regular medication schedule.
- Assessment of AEs and concomitant medications.

#### 7.2.9. Day17: End-of-Study Procedures

The following procedures will be performed at the End-of-Study or Termination Visit:

- Blood and urine sample collection for clinical chemistry, hematology and urinalysis in the morning under fasting condition (after at least a 10-hour fast).
- Urine pregnancy test for women.
- Physical examination.
- Measure weight.
- Vital signs (BP, PR, RR and oral T) measurement after resting supine for at least 5 minutes.
- Standard 12-lead ECG after resting supine for at least 5 minutes.
- Assessment of AEs and concomitant medications.
- Subjects will be offered breakfast and may be discharged from the CRC upon completion of all study procedures.

### 7.3. Early Withdrawal Visit

As outlined in Section 6.4, in the event that a subject discontinues study participation after Day 1 dose administration but prior to the final End-of-Study visit, if possible, the subject should complete all End-of Study assessments prior to being discharged from the CRC, as described in

Section 7.2.9 for Day 17. The reason for discontinuation must be fully documented in the subject's source documentation and in the eCRF.

### 7.4. Time Windows for Procedures

Any procedure performed outside the stated time windows below will be recorded as a protocol deviation.

- Sitagliptin and dorzagliatin administered by clinic staff will be taken 60 (±5) minutes prior to meals.
- Standard resting 12-lead ECGs will be performed 2 hours (±15 minutes) after the study drug dose, after the subject has rested in the supine position for at least 5 minutes.
- The oral glucose solution will be administered 30 (±5) minutes after study drug dose on Days 5, 10 and 15.
- Blood samples for PK analyses will be collected ±5 minutes from 0-4 hours post-dose and ±10 minutes ≥6 hours post-dose.
- Blood samples for PD analyses will be collected ±5 minutes from 0-4 hours after glucose intake.
- Meal start times will be within ±10 minutes of scheduled start time.

## 8. TREATMENT OF SUBJECTS

### 8.1. Description of Study Drugs

Dorzagliatin (HMS5552) is an investigational novel GKA, indicated as an adjunct to diet and exercise to improve glycemic control in adults with T2DM.

The anticipated dosing regimen is twice daily as oral administration. The proposed dosage form for this study is a film-coated tablet with 75 mg dorzagliatin. [redacted]

[redacted]

[redacted]

Sitagliptin ([redacted]® 100 mg tablets for oral administration) will be supplied by Hua Medicine.

## 8.2. Methods of Assigning Subjects to Dose Groups

There is no randomization since this is an open label, single sequence study. Subjects may be enrolled singly or in groups.

## 8.3. Blinding

This is an open label study. There will be no blinding for this study.

## 8.4. Treatment Discontinuation/Subject Withdrawal

Subjects may withdraw from the study at any time at their own request, or they may be withdrawn at any time at the discretion of the Investigator or sponsor for safety, behavioral, or administrative reasons. If a subject discontinues study participation at any time prior to the final End-of-Study assessments, if possible, the subject should complete all End-of Study assessments. The reason for discontinuation must be fully documented in the subject's source documentation and in the eCRF.

## 8.5. Prior and Concomitant Medications and Substances

All prescription medications and OTC products, including herbal products, taken within 30 days prior to screening and during the study period will be documented in the subject's source documentation and the eCRF. Therapy to treat T2DM taken within 3 months prior to screening will also be documented in the subject's source documentation and the eCRF. The documented use of prescription sitagliptin during the run-in period is required.

## 8.6. Meals

Standardized meals based on the recommended American Diabetes Association diet, will be offered to all subjects. An evening snack (no later than 09:00 p.m.) will be offered on the evening of admission. Subjects will be domiciled and dosed, and have meals provided by the CRC. Standard meals will be served at approximately 60 minutes after dosing and shall be completed within 30 minutes when meals are administered after a study drug dose. While domiciled at the CRC, meals will be offered at approximately 09:00 a.m., 12:30 p.m. and

07:00 p.m., and a light evening snack will be offered at approximately 9:00 p.m. or earlier. Meal start times will be within ±10 minutes when the meal time coincides with a scheduled PK or PD blood draw. Soft drinks (sodas) without caffeine or sugar, or non-grapefruit juices without added sugar will be offered with meals and ad libitum beginning 4 hours post-dose.

Water intake will be restricted from 1 hour prior to dosing until after the 1-hour post-dose PK blood sample is collected (with the exception of 240 mL of water given with the dosing) on study Days 5, 10 and 15.

# 9. STUDY DRUG MATERIALS AND MANAGEMENT

## 9.1. Study Drug Packaging and Labeling

Dorzagliatin drug products are supplied as 75 mg strength tablets. The drug product tablets are packaged in Alu-Pla Blister as in total 8 tablets per plate.

Product will be labeled according to applicable regulatory requirements. The label will include at least the following information:

- Name and address of manufacturer
- Protocol number or other identifier to reference the study
- Name of the sponsor, product name and batch number
- Place to record subject number and initials
- Storage conditions

The CRC pharmacist or designee will prepare the study drug and maintain the drug packaging and labeling log.

Sitagliptin ([redacted]® 100 mg tablets for oral administration) will be supplied by Hua Medicine.

## 9.2. Study Drug Storage and Accountability

All study drug supplies will be stored in a secure, locked area, under the responsibility of the Investigator or other authorized individual.

Dorzagliatin must be stored under controlled room temperature (10-30°C or 50-86°F) in tightly closed containers protected from light. Sitagliptin must be stored at 20-25°C (68-77°F) with

excursions permitted to 15-30°C (59-86°F). The temperature and humidity of the stored room should be monitored and recorded.

The Investigator or designee must maintain accurate records of the receipt of all study drug, including date received, lot number, expiration date if applicable, amount received, condition of the package and the disposition of all study drug.

Current dispensing records will also be maintained including the date and amount of study drug dispensed and the identity of the subject receiving the study drug.

## 9.3. Study Drug Preparation

Sitagliptin 100 mg tablets will be dispensed to subjects for at-home dosing during the 12-day run-in period. Study drug reconciliation will be performed at the end of the run-in period by tablet count.

Dorzagliatin 75 mg and sitagliptin 100 mg tablets will be dispensed to subjects while domiciled at the CRC according the study treatment scheme. Morning and evening doses of study drug will be administered by CRC staff with approximately 240 mL (8 fluid ounces) room temperature water at approximately 08:00 a.m. and 06:00 p.m., 60 (±5) minutes prior to a meal, except for Days 5, 10 and 15 when there will be no meal following the morning dose.

## 9.4. Study Drug Handling and Disposal

All unused study drug and supplies must be returned to sponsor or disposed according to sponsor's instruction after the study is completed and the drug accountability log is reconciled.

# 10. PHARMACOKINETIC/PHARMACODYNAMIC ASSESSMENTS

## 10.1. Pharmacokinetic Sample Collection and Storage

A total of 42 blood samples (approximately 6 mL each) will be collected from each subject into $K_2EDTA$ tubes for determination of dorzagliatin and sitagliptin concentration and PK assessments. Samples will be collected [redacted] on Days 5, 10 and 15 using an indwelling catheter or direct venipuncture.

When PK blood collection times coincide with other procedures, the blood sample should be collected first, using the following post-dose windows: ±5 minutes from 0-4 hours post-dose and ±10 minutes >6 hours post-dose.

Contents of tube will be mixed thoroughly with gentle inversion at least 8 times to mix the anti-coagulant and stored on ice for no more than 30 minutes before centrifugation at approximately 4 (±1)°C at ~2000 g for 10 minutes. The harvested plasma will be split into two approximately equal aliquots and stored in 2 mL or appropriate size cryovial tubes.

Plasma samples will be stored at -70 (±10)°C within approximately 60 minutes of harvesting pending shipment to the bioanalytical laboratory for analysis. Full instructions for the collection, storage, and shipment of these samples will be provided in a separate PK Laboratory Manual.

## 10.2. Pharmacokinetic Specimen Labeling

Labels will be affixed to the cryovials in a manner that will prevent the label from being detached after being wet or freezing. The tube labels will contain the subject number, treatment, nominal day, and nominal collection time, as appropriate.

The site will provide a sample inventory page, listing the information above for each sample. The sample inventory page will also include the treatment cohort for each series of tubes.

## 10.3. Pharmacokinetic Sample Shipping Instructions

All PK plasma samples will be kept frozen and shipped on dry ice by the same day or overnight courier to:

Frontage Laboratories, Inc.
Sample Coordinator
[redacted]

Plasma samples will be shipped in two separate shipments. The first shipment of samples (the primary aliquot of each sample) will be shipped after subjects have completed the study. The second aliquot of each sample will be shipped after notification from the laboratory of receipt of primary samples.

## 10.4. Bioanalytical Methodology

The PK plasma samples will be analyzed using validated, specific and sensitive methods of liquid chromatographic separation with tandem mass spectrometric (LC-MS/MS) detection for concentrations of dorzagliatin and sitagliptin by the designated bioanalytical lab.

## 10.5. Pharmacokinetic Parameters

Dorzagliatin and sitagliptin PK parameters ($C_{max}$, $T_{max}$ and $AUC_{0-24h}$) will be computed for each subject using non-compartmental methods by Phoenix WinNonlin version 8.1 (Certara, Princeton, NJ USA) based on the actual sampling times. Additional PK parameters ($T_{1/2}$, $K_{el}$, $V_z/F$, CL/F) may be calculated if needed. Descriptive statistics will be calculated for the PK parameters for Days 5, 10 and 15. A description of PK terms is provided below.

**Table 10-1: PK Terms**

| PK Term | Description |
|---|---|
| $C_{max}$ | Observed maximum plasma concentration |
| $T_{max}$ | Time at which $C_{max}$ was first observed |
| $AUC_{0-24h}$ | Area under the concentration-time curve from 0 to 24 hours |
| $T_{1/2}$ | Terminal elimination half-life |
| $K_{el}$ | Elimination rate constant |
| $V_z/F$ | Apparent volume of distribution during terminal phase after oral administration |
| CL/F | Apparent total plasma clearance of drug after oral administration |

## 10.6. Pharmacodynamic Assessments

Subjects will consume a 75-gram glucose solution 30 (±5) minutes following study drug administration on Days 5, 10 and 15, in lieu of a breakfast. The glucose solution will be consumed within a 5-minute timeframe. A total of 48 blood samples (24 samples of approximately 8.5 mL each for measurement of plasma GLP-1 and glucagon, and 24 samples of approximately 7 mL each for measurement of serum glucose, insulin, and C-peptide will be collected from each subject for PD assessments. Samples will be collected at baseline (prior to the study drug dose and within 60 minutes prior to oral glucose intake) and 0.5, 1, 1.5, 2, 2.5, 3 and 4 hours after oral glucose intake on Days 5, 10 and 15.

When PD blood collection times coincide with other procedures, the blood sample should be collected first within the ±5 minutes time window.

For measurement of plasma GLP-1 and glucagon, approximately 8.5 mL blood will be collected into Becton Dickenson BD P800 tubes that contain spray-dried $K_2$EDTA anticoagulant and other proprietary additives. Contents will be gently inverted 8-10 times and centrifuged within 60 minutes of collection at 1100-1300 g for 20 minutes at 18-25°C. Three aliquots of plasma will be harvested as 1.0 mL for measurement of glucagon and active GLP-1, 1.0 mL for measurement of total GLP-1 and 1.5 mL as a back-up sample. All samples will be stored immediately at ≤70°C until shipment to the Bioanalytical laboratory. Samples will be shipped on dry ice to:

[redacted]
Mercodia AB
[redacted]

For measurement of serum glucose, insulin and C-Peptide, the sample collection tubes and processing instructions will be provided by BioReference Laboratories, the facility that will measure all safety laboratory assessments.

Full instructions for the collection, storage, and shipment of PD samples will be provided in a separate PD Laboratory Manual.

# 11. ASSESSMENT OF SAFETY

Safety assessments will include monitoring of AEs, blood glucose via glucometer readings, vital signs, clinical laboratory findings, resting 12-lead ECGs and PE findings.

## 11.1. Safety Parameters

### 11.1.1. Demographic/Medical History

Demographic characteristics (age, sex, race and ethnicity) will be collected at the screening visit. Medical history will be reviewed and collected at the screening and on Day -2.

### 11.1.2. Vital Signs

Vital signs, including BP, PR, RR, and oral T, will be measured at screening, on Day -1, on Days 1-15 within 60 minutes prior to each morning and evening dose, and on Day 17 or at the end of the study. Vital signs will be measured after resting supine for at least 5 minutes.

Vital signs may be repeated for clinically significant abnormal vital signs at the discretion of the Investigator.

As a guideline, vital signs outside the ranges shown below may be considered abnormal, at the discretion of the Investigator:

SBP: 95-160 mmHg

DBP: 55-100 mmHg

PR: 45-100 bpm

RR: 10-20 bpm

T: 36.0-37.2°C

### 11.1.3. Physical Examination, Height, Weight, and Body Mass Index

Full PEs will be performed by qualified personnel at the screening visit, Day -1, Day 17 and the End-of-Study visit. At the screening visit, height (centimeters) and weight (kilograms) will be measured and BMI will be calculated. Weight will also be measured on Day -1 and Day 17. All abnormal findings will be documented in the source documentation and in the eCRF.

### 11.1.4. Electrocardiogram

Standard resting 12-lead ECGs will be performed at the screening visit, Day -1, 2 hours (±15 minutes) post-dose on Days 5, 10 and 15, and on Day 17 at the End-of-Study visit. All ECGs will be performed after the subject has rested supine for at least 5 minutes.

Post-dose ECGs must be evaluated for safety by the Investigator or his/her designee. The final conduction intervals entered into the eCRF will be those generated by the ECG machine, unless deemed significantly inaccurate by the review performed by the Investigator or designee. In these cases, the over-read intervals will be documented in the eCRF. The Investigator will also record an overall assessment of the ECG.

Clinically significant abnormal ECGs may be repeated at the discretion of the Investigator.

As a guideline, ECG parameters outside the ranges shown below may be considered abnormal, at the discretion of the Investigator:

VR: 45-100 bpm

PR: 120-210 msec

QRS: ≤120 msec

QT: <500 msec

QTc: <450 msec

### 11.1.5. Clinical Laboratory Assessments

Hematology, blood chemistry, and urinalysis evaluations will be performed at the screening visit, on Day -1, Days 5, 10, and 15, and Day 17 (End-of-Study Visit). Blood and urine samples will be collected after an overnight fast of at least 10 hours, and within 30 minutes prior to the morning dose of study drug on Days 5, 10 and 15. The list of clinical laboratory assessments is included in Appendix A.

C-peptide and HbA1c testing will be performed at the screening visit to determine eligibility.

Blood glucose will be measured using a glucometer three times each day on Days -2 through Day 16, within 60 minutes before each meal and after study drug dosing when applicable.

The results of clinical laboratory tests conducted at the screening visit and on Day -1 must be assessed by the Investigator to determine each subject's eligibility for participation in the study. The Investigator should signify review of the laboratory reports by signing and dating the report. If subjects' values are out of range and the Investigator deems the out of range value is clinically significant, the Investigator must discuss and agree upon individual cases with sponsor's Medical Monitor prior to enrollment.

Any significant abnormalities should be fully investigated. Whenever possible, the etiology of the abnormal findings will be documented on the eCRF. Laboratory results with clinically significant abnormal values may be repeated for verification. Additional tests and other evaluations required to establish the significance or etiology of an abnormal result or to monitor the course of an AE should be obtained when clinically indicated. In particular, if a clinically significant abnormal result is observed that is not resolved by the final study visit, repeat tests

will be performed if possible and resolution or stability of the abnormality will be recorded in the source documentation.

Any clinically significant laboratory abnormalities that are either serious (e.g., results in hospital admission) or unexpected will be promptly reported to the representative of sponsor. Any additional relevant laboratory results obtained by the Investigator during the course of this study will be reported to sponsor.

Virus serology (HIV, and hepatitis B and C) will be assessed at the screening visit and must be negative to qualify enrollment.

Urine drug screens and saliva alcohol tests will be conducted at the screening visit and on Day -2. Results must be negative to qualify for dosing on Day 1.

Urine pregnancy test for all female subjects will be performed at the screening visit, on Day -2 and on Day 17 (End-of-Study). Results at screening and Day -2 must be negative to qualify for dosing on Day 1. Any positive pregnancy test results on Day 17 will be reported to the sponsor within 24 hours of awareness of the pregnancy using a Pregnancy Report provided by Frontage.

Postmenopausal status of females will be confirmed by serum FSH and estradiol levels at screening.

## 11.2. Adverse Events

Adverse events will be recorded after the first dose of sitagliptin in the run-in period. Subjects will be instructed to report AEs during the study and staff will query subjects regarding AEs throughout the study. The Investigator or designee must document all AEs reported by the subject after the first dose of sitagliptin in the run-in period through completion of the End-of-Study visit. Any subject who is withdrawn from the study due to an AE shall be followed until the event has resolved or stabilized, and the Investigator will document available follow-up information on the subject’s source documentation and eCRF.

### 11.2.1. Definitions of Adverse Events

Adverse event means any untoward medical occurrence in a patient or clinical investigation subject administered a pharmaceutical product and which does not necessarily have to have a causal relationship with this treatment. An AE can therefore be any unfavorable and unintended

sign, symptom, or disease temporally associated with the use of medicinal product, whether or not related to the medicinal product. An AE is any symptom, sign, illness, or experience that develops or worsens in severity and/or frequency during the course of the study. Adverse events reported after administration of the first dose of study drugs (Day 1) will be considered treatment-emergent. Adverse events will be considered treatment-emergent if not present prior to the initiation of the treatment with study drug on Day 1 or already present but worsens in either severity or frequency following exposure to the treatment.

Adverse events include serious and non-serious AEs. A Serious Adverse Event (SAE) is defined as an AE occurring during any clinical study period that meets one or more following criteria:

- Results in death
- Is life threatening
- Requires inpatient hospitalization or prolongation of existing hospitalization
- Results in persistent or significant disability/incapacity
- Is a congenital anomaly/birth defect
- Is a medically important event that may jeopardize the subject or may require medical or surgical intervention to prevent one of the other outcomes listed above

The term "life threatening" in the definition of "serious" refers to an event in which the subject was at risk of death at the time of the event; it does not refer to an event that hypothetically might have caused death if it were more severe. Death is an outcome of an event. The event that resulted in death should be recorded in source documents, the eCRF and reported on the SAE form. All SAEs that occur after consent to the End-of-Study visit must be reported to sponsor using the SAE Reporting Form provided by Frontage (see Section 11.2.3).

Abnormal results of laboratory tests or diagnostic procedures (such as test results from hematology, blood biochemistry, urinalysis, ECG, physical examination, vital signs evaluations, etc.) are considered to be AEs if the abnormality is considered by the Investigator as clinically significant, or the clinical significance worsens compared to the subject's baseline.

Any of the following abnormal results are considered clinically significant:

- The abnormality suggests a disease and/or organ toxicity that is new or has worsened from baseline;

- The abnormality needs to adjust investigational product dosage and usage, e.g., drug discontinuation;
- The abnormality requires additional active intervention, for instance an increase or a modification of the concomitant medication, close observation, or further diagnostic investigation, etc.

If a clinically significant laboratory abnormality is a manifestation of disease, then only the diagnosis will be recorded as AE. If a clinically significant laboratory abnormality is not a manifestation of disease, then the abnormality itself is recorded as AE. An appropriate description is used to record test results that are either lower or higher than normal range. If the result meets diagnostic criteria, the clinical diagnosis is recorded as AE.

The severity of each AE will be graded by the Investigator according to the following criteria:

- Mild: awareness of sign or symptom, but easily tolerated.
- Moderate: discomfort sufficient to cause interference with normal activities.
- Severe: incapacitating, with inability to perform normal activities.

The Investigator must assess the relationship between the AE and the study drug by using the following definitions:

1. **Related**: The occurrence of AE follows a reasonable temporal sequence from administration of the study drug; adverse event is consistent with known characteristics of the study drug; the event is improved when the dose of study drug is decreased or stopped; the event re-occurs when the study drug treatment is re-started. It cannot be explained by the medical condition of the subject or alternative treatment.

2. **Possibly Related**: The occurrence of AE follows a reasonable temporal sequence from administration of the study drug; AE is consistent with a known characteristic of the study drug; reducing the dosage or stop the drug will cause the AE to be alleviated or no obvious change. The medical condition of the subject or alternative treatment may have contributed to the event.

3. **Unlikely Related**: The occurrence of AE, whose temporal sequence from administration of the study drug, is unclear. The AE may not be consistent with a known characteristic

of the study drug. The medical condition of the subject or alternative treatments may have contributed to the event.

4. **Not Related**: There is no reasonable temporal relationship between the AE occurrence and study drug administration; the AE is not consistent with known characteristic of the study drug; the medical condition of the subject or alternative treatments may have contributed to the event. The AE improves or disappears when disease condition improves, or alternative treatment is stopped.

The outcome of an AE can be described as:

- Recovered: the subject returns to baseline state.
- Recovering: the events haven't been resolved completely, but subject is improving.
- Not recovered: the events are ongoing, for example, irreversible congenital anomaly
- Recovered with sequelae: only if the subject will suffer from life-time sequelae, for example, the blindness caused by diabetes and the hemiplegia after a stroke.
- Fatal: the death date is the event end date.
- Unknown: Investigator can't obtain the outcomes of AEs, e.g., the subject is lost to follow-up.

If the AE outcome is assessed as "Recovered", "Recovered with sequelae", or "Fatal", the end date of the AE must be recorded.

### 11.2.2. Recording Adverse Events

All AEs (regardless of seriousness or relationship to study drug) are to be recorded in the subject's source documents and on the corresponding page(s) in the eCRF. Whenever possible, symptoms, signs, and laboratory abnormalities should be grouped as a single syndrome or diagnosis. The Investigator should specify the date of onset and stop date of event, severity and the time of severity worsening, action taken with respect to study drug, corrective treatment/therapy given, seriousness, outcome, hospitalization date (if applicable), discharge date (if applicable), cause of the death (if applicable), date of death (if applicable), autopsy (if applicable) and his/her opinion as to whether there is a reasonable possibility that the AE was caused by the study drug, according to the definitions noted in Section 11.2.1. All medications administered to treat an AE must be recorded in the subject's source documentation and documented in the eCRF.

### 11.2.3. Reporting of Serious Adverse Events

Reporting of SAEs will be conducted in accordance with the appropriate regulatory guidelines. All SAEs that occur from the time of consent to the End-of-Study visit must be reported, whether or not the event is considered associated with the study drug. The Investigator must complete the SAE Reporting Form provided by Frontage and submit it by fax or email with other relevant source documentation to sponsor within 24 hours of awareness of the event to:

[redacted]

The Investigator must also provide with urgent priority (upon receipt of a request) other relevant documentation (e.g., copies of diagnostic test results, hospital discharge summary, and/or autopsy report) and send this information by fax or email to sponsor.

All SAEs must be recorded in the subject’s source documentation and documented in the eCRF. Medications administered in association with the SAE must be documented in the eCRF and in the subject’s source documentation. The Investigator must also promptly notify the Institutional Review Board (IRB) /Independent Ethics Committee (IEC) of SAEs, including any follow-up information, in accordance with local institutional policy and applicable regulatory requirements.

Regulatory authorities will be notified of any AE associated with the use of the study drug that is both serious and unexpected by sponsor, in accordance with the appropriate local regulatory guidelines. Notification of the event will be made by a written, expedited safety report.

### 11.2.4. Adverse Event Follow-up

The Investigator should take all appropriate measures to ensure the safety of the subjects, including referral to a specialist if indicated. The Investigator should follow up on the outcome of any AE until the event has resolved or stabilized.

This implies that follow-up may continue after the subject discontinues from the study and that additional information may be requested. Any SAE follow-up information that occurs after database lock should be reported to sponsor.

#### 11.2.5. Hy’s Law

The study will utilize Hy’s Law to monitor any potential drug-induced liver injury. Hy’s Law usually means that AST or ALT > 3 x ULN and TBiL > 2 x ULN, non-biliary increase (usually alkaline phosphatase < 2 x ULN) without any other diseases can explain this increase. If ALT or AST > 3 x ULN, and TBiL > 2 x ULN without any other past diseases can explain this increase, no matter ALP increases or not, re-evaluation need to be performed. Investigator will conduct subject re-evaluation, and closely follow-up of the subjects or stop the study drug due to liver function abnormality, according to Section 6.4 Subject withdrawal. The Investigator will evaluate the etiology and perform every essential examination to rule out drug-induced liver injury. If Investigator confirms the occurrence of Hy’s Law, an SAE must be reported.

#### 11.2.6. Hypoglycemia

Hypoglycemia events shall be recorded on an eCRF page. The following information will be recorded for each occurrence of hypoglycemia:

- Start/end date and time
- Blood glucose values
- Symptom descriptions
- Action taken
- Severe hypoglycemia or not
- Resolved or not
- Predisposing factors
- Causal relationship with the study drug

Hypoglycemia should be treated using standard medical practice at the Investigator’s discretion. Severe hypoglycemia events will be reported as an SAE.

# 12. STATISTICAL METHODOLOGY

## 12.1. Sample Size Determination

Sample size calculations based on study design and intra-subject variability were performed by the sponsor. At least 10 evaluable subjects in the sequence will be required to achieve a power of at least 0.8 for the geometric mean ratios between two treatments (sitagliptin + dorzagliatin vs.

dorzagliatin alone or sitagliptin + dorzagliatin vs. Sitagliptin alone) for $C_{max}$ or $AUC_{0-24h}$, with the equivalence bounds of 0.8 and 1.25, assuming a true geometric mean ratio of 1 and an intra-subject variability (coefficient of variation) of 16.1%, in an equivalence test using two one-sided test at a significance level of 0.05. The intra-subject variability for sitagliptin $C_{max}$ and AUC are reported to be 16.1% and 5.7%, respectively.[10] The intra-subject variability for dorzagliatin $C_{max}$ and $AUC_{0-24h}$ are estimated to be 14.0% and 6.2%, respectively.

Therefore, to ensure a satisfactory DDI assessment, and assuming a drop-out rate of 20%, we plan to enroll 15 eligible subjects by aiming to obtain 12 evaluable subjects for DDI assessment.

## 12.2. Analysis Population

The Safety Population will be defined as all subjects who receive study drug.

The PK Population will be defined as all subjects who receive study drug, have no major protocol violations, and have sufficient PK data to obtain reliable estimates of the key PK variables.

The PD Population will be defined as all subjects who receive study drug, have no major protocol violations, and have sufficient PD data to obtain reliable estimates of the key PD parameters.

The DDI Population will consist of the PK Population subjects who complete all treatments as defined by the protocol.

## 12.3. Pharmacokinetics and Pharmacodynamics

Pharmacokinetic parameters described in Section 10 will be summarized by descriptive statistics (n, mean, standard deviation, median, minimum, maximum) or frequency counts. Descriptive statistics will be calculated for dorzagliatin and sitagliptin concentrations in plasma.

Dorzagliatin and sitagliptin PK parameters ($C_{max}$, $T_{max}$ and $AUC_{0-24h}$) will be computed for each subject using non-compartmental methods and based on the actual sampling times. Descriptive statistics will be calculated for the PK parameters on Days 5, 10 and 15.

The effect of sitagliptin on the exposure of dorzagliatin will be determined by a PK comparison between the exposure of dorzagliatin on Day 10 vs. Day 15. The effect of dorzagliatin on the

exposure of sitagliptin will be determined by a PK comparison between the exposure of sitagliptin on Day 10 vs. Day 5.

The primary PK comparison will be based on the $C_{max}$ and $AUC_{0-24h}$ of dorzagliatin and sitagliptin. A mixed model under the sequential design will be used for analyzing $C_{max}$ and $AUC_{0-24h}$ values, based on natural log-transformed values, with treatments as fixed effects and subjects within sequence as random effects. The adjusted geometric mean ratios between two treatments for these parameters and the corresponding 90% confidence intervals (CI) for the ratio will be obtained by exponentiating the adjusted mean difference in logarithms and will be used for assessing PK differences between the two treatments within each PK comparison.

A Statistical Analysis Plan (SAP) will be developed and signed prior to database lock, and will describe in more detail how PK parameters will be derived.

Pharmacodynamic measurements (glucose, GLP-1, glucagon, insulin, and C-peptide) will be listed by subject for all subjects with actual sampling time. Summary statistics of glucose, GLP-1, glucagon, insulin, and C-peptide will be provided by scheduled (nominal) time point and treatment, respectively. Baseline corrected PD markers will be calculated by subtracting the baseline value from the values post oral glucose intake. The baseline for PD correction will be defined in the SAP. Plots of mean PD markers versus the scheduled time will be generated for each treatment. Derivations and statistical analysis methods of PD parameter ($AUEC_{0-4h}$, $CE_{max}$ and $CE_{av}$) will be described in detail in the SAP.

## 12.4. Demographic Characteristics

Demographic characteristics will be summarized for the subjects enrolled in the study using descriptive statistics.

An attempt will be made to enroll similar numbers of men and women in the study.

## 12.5. Exposure to Study Drugs

Each subject’s exposure to study drug will be summarized using descriptive statistics, i.e., the number of subjects exposed to each treatment.

### 12.6. Concomitant Medications

Concomitant medications will be coded using the World Health Organization (WHO) Enhanced dictionary.

### 12.7. Safety Analyses

Safety evaluations will be based on the incidence, severity, and relatedness of AEs and changes in subjects' PE findings, ECGs, vital signs, and clinical laboratory results.

Safety variables will be tabulated and presented for all subjects in the Safety Population. Data will be summarized by treatment. Adverse events will be coded using the Medical Dictionary for Regulatory Activities (MedDRA) for preferred term and system organ class, listed by subject. TEAEs will be summarized by treatment. All AEs will be summarized by relationship to study drug and by severity.

Deaths, SAEs, and AEs resulting in study discontinuation will be tabulated and detailed in narratives.

Change from baseline, defined as time of admission to the CRC or screening, whichever value is the last value prior to first dose, in clinical laboratory parameters, 12-lead ECGs, and vital sign parameters will be summarized by treatment.

Additional safety analyses may be defined in the SAP.

### 12.8. Interim Analyses

No interim analyses are planned for this study.

## 13. DIRECT ACCESS TO SOURCE DATA/DOCUMENTS

### 13.1. Study Monitoring

At regular intervals during the study, the site will be contacted through monitoring visits, letters, and telephone calls by a representative to review study progress, Investigator and subject compliance with study protocol requirements, and any emergent problems. During monitoring visits, the following points will be reviewed in accordance with all applicable regulatory requirements and Standard Operating Procedures (SOPs): original medical records and other source documents, the Investigator site file, screening logs, ICF, subject recruitment and follow-

up, SAE documentation and reporting, documentation and reporting of endpoints, study drug allocation, subject compliance with the study drug regimen, study drug accountability, concomitant therapy use, and quality of data. In addition, other required regulatory documents will be reviewed, including but not limited to: IRB/IEC composition and correspondence, laboratory certification(s), delegation of authority, and Investigator and study personnel curricula vitae.

## 13.2. Sponsor's Responsibility

The sponsor or its designee is responsible for the following:

- Selecting qualified Investigators;
- Providing Investigators with the information they need to properly conduct an investigation;
- Ensuring proper monitoring of the investigation;
- Ensuring that the applicable regulatory authorities, and all participating Investigators are properly informed of significant new information regarding AEs or risks associated with the medication being studied.

As the sponsor, Hua Medicine has delegated some responsibilities to Frontage Clinical Services, a Contract Research Organization (CRO).

## 13.3. Audits and Inspections

The sponsor's Quality Assurance Unit (or representative) may conduct audits at the study site(s). Audits will include, but are not limited to: drug supply, presence of required documents, the informed consent process, laboratory specimen processing, and comparison of eCRFs with source documents. The Investigator agrees to cooperate with audits conducted at a reasonable time and in a reasonable manner.

Regulatory authorities worldwide may also inspect the Investigator site during or after the study. The Investigator should contact the sponsor immediately if this occurs and must fully cooperate with the Inspector.

The Investigator is required to make all study documentation promptly available for inspection, review or audit at the study site upon request by sponsor, its representatives, or any appropriate regulatory agencies.

# 14. QUALITY CONTROL AND QUALITY ASSURANCE

Each investigational site will perform internal quality management of study conduct, data and biological specimen collection, documentation and completion. An individualized quality management plan may be developed to describe a site's quality management.

Quality control (QC) procedures will be implemented beginning with the data entry system and data QC checks that will be run on the database will be generated. Any missing data or data anomalies will be communicated to the site(s) for clarification/resolution.

Following written SOPs, the monitors will verify that the clinical trial is conducted, and data are generated, and biological specimens are collected, documented (recorded), and reported in compliance with the protocol, ICH GCP, and applicable regulatory requirements (e.g., GLP, Good Manufacturing Practices (GMP)).

The investigational sites will provide direct access to all trial related sites, source data/documents, and reports for the purpose of monitoring and auditing by the sponsor, and inspection by local and regulatory authorities.

# 15. ETHICS

The procedures set out in this study protocol, pertaining to the conduct, evaluation, and documentation of this study, are designed to ensure that the sponsor and the Investigator abide by GCP, including but not limited to Title 21 Code of Federal Regulations (CFR) Parts 50, 56, and 312 and the ICH guidelines and directives. Compliance with these regulations also constitutes compliance with the ethical principles described in the current revision of the Declaration of Helsinki, Fortaleza 2013 and applicable local regulatory requirements and law.

Copies of these materials are available from Hua Medicine and Frontage Clinical Services (the CRO) designee by request. The purpose of these regulations, legal obligations and guidance is to define the standards and principles for the proper conduct of clinical studies that have been

developed by the medical, scientific, and regulatory communities. They are not intended to impede or restrict clinical research.

The ethical standards defined within GCP are intended to ensure that:

1. Human subjects are provided with an adequate understanding of the possible risks of their participation in the study, and that they have a free choice to participate or not;
2. The study is conducted with diligence and in conformance with the protocol in such a way as to protect subject safety and ensure the integrity of the findings;
3. The potential benefits of the research justify the risks.

The Investigator is responsible for protecting the rights, safety, and welfare of subjects under his/her care, and for the control of the medications under investigation.

The Principal Investigator will ensure that all persons assisting with the study are adequately informed about the protocol, any amendments to the protocol, the study drug, and their study related duties and functions. The Investigator will maintain a list of sub-Investigators and other appropriately qualified persons to whom he or she has delegated significant study-related duties. Individuals ineligible to conduct or work on clinical studies, including those ineligible as a result of debarment under the Generic Drug Enforcement Act of 1992, will not be allowed to conduct or work on studies sponsored by Hua Medicine. The Investigator is required to immediately disclose to sponsor in writing, if any person involved in the conduct of the study is debarred pursuant to a hearing by US FDA under this anti-fraud law, or if any proceeding for debarment is pending, or is (to the best of the Investigator’s knowledge) threatened.

The rest of this section of the protocol describes in more detail the specific GCP-defined responsibilities the Investigator assumes by agreeing to participate in this study.

## 15.1. Ethics Review

The Investigator (or designee) must submit this study protocol, the sponsor approved ICF, information intended for distribution to subjects, subject recruitment materials, and other appropriated documents to the appropriate IRB or IEC. Following IRB or IEC review, a copy of the signed, written and dated approval will be provided to the sponsor, along with a list of the IRB/IEC composition.

The approval/favorable opinion should clearly state the study (study number, protocol title, and version number), the documents reviewed (Protocol, ICF, IB, etc.) and the date of the review. The study will not commence at the study site until sponsor has received a copy of this written and dated approval/favorable opinion.

During the study, any amendment to the protocol and the ICF (as appropriate) shall be submitted to the IRB/IEC. The IRB/IEC should also be informed of any event likely to affect the safety of subjects or the continued conduct of the study, in particular any change in safety. Additionally, all updates to the IB will be sent to the IRB/IEC. A progress report will be sent to the IRB/IEC and the protocol will be reviewed annually (e.g., re-approved) or more frequently, as required by IRB/IEC or local regulations.

The Investigator will notify the IRB/IEC of the conclusion of the clinical study within 1 month of completion or termination of the study. The final report sent to the IRB/IEC will also be sent to sponsor, along with the completed eCRFs and all necessary regulatory documents, thereby fulfilling the Investigator's regulatory responsibility.

The Investigator will maintain a copy of all correspondence with the IRB/IEC, including copies of approved documents. The Investigator will also maintain a copy of the IRB/IEC membership list, including members' occupation and qualifications (or a statement confirming compliance with GCP requirements for committee composition). An IRB or IEC General Assurance Number may be accepted in lieu of a membership roster.

## 15.2. Ethical Conduct of the Study

This study will be conducted in accordance with GCP as delineated by Title 21 CFR Parts 50, 56, and 312, and the ICH guidelines and directives. Participating Investigators, including members of the Committees and National Coordinators, will receive compensation for their time but will receive no financial profit from their activities related to the study.

Before the first subject is enrolled in the study, all ethical, regulatory, and legal requirements must be met.

An Investigator participating in this study is required to maintain complete and accurate study documentation in compliance with current GCP standards and all applicable local regulations related to the conduct of a clinical study.

### 15.3. Written Informed Consent

Prior to performing any study-related activities under this protocol, including screening tests and assessments, written informed consent with the approved ICF must be obtained from the subject or subject's legally authorized representative, as applicable, in accordance with local practice and regulations. Written informed consent must be obtained from all subjects participating in a clinical study conducted by Hua Medicine.

The background of the proposed study, the procedures, the benefits and risks of the study, and that study participation is voluntary for the subject must be explained to the subject. The subject must be given sufficient time to consider whether to participate in the study.

A copy of the ICF, signed and dated by the subject, must be given to the subject. Confirmation of a subject's informed consent must also be documented in the subject's source documentation prior to any testing under this protocol, including screening tests and assessments.

Each consent form should contain an authorization allowing the Principal Investigator(s) and the sponsor to use and disclose patient health information (PHI) in compliance with local law.

The originally signed consent form will be retained with the study records.

## 16. DATA HANDLING AND RECORD KEEPING

### 16.1. Data Collection

All data obtained for analysis in the clinical study described in this protocol will be documented in the eCRF. Data reported in the eCRFs should be consistent with and substantiated by the subject's medical record and original source documents.

Prior to the start of the study, the Principal Investigator will complete a Delegation of Authority form (Site Signature and Delegation Log), showing the signatures and handwritten initials of all individuals and the delegation of responsibilities, such as identifying those individuals who are authorized to make or change entries on eCRFs.

### 16.2. Case Report Form Completion

All eCRF entries, corrections, and alterations must be made by the investigator or authorized study-site personnel. Data within the eCRF will be monitored by the Clinical Research Associate according to the Monitoring Plan. Queries will be generated based on discrepancies found while

monitoring. Site personnel will review and respond to these queries appropriately. Additionally, the sponsor may periodically perform aggregate data reviews, which could result in queries being generated for site personnel resolution. The final, completed eCRF for each subject must be signed and dated by the Principal Investigator to signify that the Principal Investigator has reviewed the eCRF and certifies it to be complete and accurate.

## 16.3. Database Management, Data Clarification, and Quality Assurance

A designated CRO will be responsible for data management. The Data Manager will develop a Data Management Plan (DMP) document and provide it to sponsor for approval. The DMP document will define all activities in the data collection and cleaning process. The detailed DMP will be based on the protocol, work scope, contract, analysis plans, data-flows, eCRFs, data cleaning procedures, other supporting documents, and data management standards and practices.

The programmed data validations will be run, and manual review will be conducted to check for database completeness and consistency, and queries will be generated upon data entry or via review by a Clinical Data Manager after entry. The sites will respond to the data queries in a timely manner.

Prior and concomitant medications will be coded using the World Health Organization (WHO) Drug Enhanced Dictionary (latest version, to be noted in DMP). All medical history and AEs will be coded using the Medical Dictionary for Regulatory Activities (MedDRA), using latest version, to be noted in DMP.

Quality control procedures will be conducted prior to database lock according to the designated CRO SOPs.

When the database has been declared to be complete and accurate, it will be locked. Any changes to the database after that time will only be made by joint written agreement between Hua Medicine, the study Statistician, the Data Manager, and the Quality Assurance Auditor according to designated CRO SOPs.

## 16.4. Inspection of Records/Source Documents

According to the ICH guidelines for GCP, the sponsor or designee must verify data entered in the eCRF entries against the source documents, except for the pre-identified source data directly documented in the eCRF (if applicable). The ICF will include a statement by which the subject

allows the sponsor's duly authorized personnel, the IRB/IEC, and the regulatory authorities to have direct access to source data that supports the data in the eCRF (e.g., subject's medical file, appointment books, and original laboratory records). These personnel, bound by professional secrecy, must keep confidential all personal medical information.

The objective of source document verification (SDV) is to comply with GCP and international regulatory requirements and to reduce the risks of fraud. Source document verification means ensuring that the source documents are an accurate and verifiable reflection of the subject's participation in the study and that all relevant information that is recorded in the source document is accurately entered into the eCRF.

Where source documents serve as the basis for deriving data for the study, SDV should ensure that these documents are correctly labeled and filed, and that the data derived from them are correct. All source documents pertaining to this study will be maintained by the Investigator and made available for inspection by authorized persons. If electronic progress notes and other electronic source documents are not Title 21 CFR Part 11 compliant, they are not considered a valid source for this study. All progress notes must be dated and signed by the Principal Investigator or sub-Investigator at the time of the visit. The sponsor reserves the right to terminate the study for refusal of the Investigator to supply original source documentation for this clinical study.

The Investigator will note in a source independent from the eCRF the following information:

1. Information to confirm that the subject exists (e.g., initials, date of birth, sex)
2. Confirmation that the subject satisfies the inclusion/exclusion criteria
3. Confirmation that the subject is taking part in the clinical study
4. Confirmation of the informed consent process
5. Visit dates and documentation of protocol assessments and procedures
6. Information concerning all AEs
7. Details of concomitant and investigational medications

Source document verification is not a substitute for clinical study monitoring, the purpose of which is to ensure that the protocol has been followed correctly, the eCRF has been fully and accurately completed, SDV has been carried out, and the study timelines and enrollment goals and requirements have been met.

## 16.5. Retention of Records

The Investigator/institution must maintain all study documentation as confidential and take measures to prevent accidental or premature destruction of these documents.

Essential documents should be retained until at least 2 years after the last approval of a marketing application in an ICH region and until there are no pending or contemplated marketing applications in an ICH region or at least 2 years have elapsed since the formal discontinuation of clinical development of the investigational product (e.g., the Investigational New Drug application is withdrawn). These documents should be retained for a longer period, however, if required by the applicable regulatory requirements or by an agreement with sponsor.

The Investigator/institution must notify sponsor prior to destroying any study essential documents.

If the Investigator/institution can no longer ensure archiving, he/she shall inform the sponsor. The relevant records shall be transferred to a mutually agreed upon designee.

# 17. CONFIDENTIALITY

All information disclosed or provided by sponsor (or designee) or produced during the study including, but not limited to, the protocol, the eCRFs, the IB, and the results obtained during the course of the study (if applicable), are confidential. The Investigator or any person under his/her authority agrees to undertake to keep confidential and not to disclose the information to any third party without the prior written approval of sponsor.

However, submission of this protocol and any other necessary documentation to the IRB/IEC is expressly permitted, the IRB/IEC members having the same obligation of confidentiality.

All information generated in this study must be considered highly confidential and must not be disclosed to any persons not directly concerned with the study without prior written permission from sponsor. However, authorized regulatory officials and sponsor personnel (or designee) will be allowed full access to inspect and copy the records. The copied and inspected records will remain at the site and will not be transmitted or removed from the site.

All study drugs, subject bodily fluids, and/or other materials collected shall be used solely in accordance with this protocol, unless otherwise agreed to in writing by sponsor and responsible ethics committee(s) or regulatory authorities.

Subjects will be identified only by unique subject numbers in eCRFs. Their full names may, however, be made known to a regulatory agency or other authorized officials in the event of inspections. Documents containing the full name or other personally identifiable information of the subject are to remain at the site. This information will not be transferred to sponsor nor be contained in regulatory filings. In the event of inspections by authorized agencies, this subject identification may be disclosed.

## 17.1. Subject Data Protection

Prior to any testing under this protocol, including screening tests and assessments, candidates must also provide all authorizations required by local law (e.g., PHI authorization in North America).

The subject will not be identified by name in the eCRF or in any study reports and these reports will be used for research purposes only. The sponsor, its partner(s) and designee(s), and various government health agencies may inspect the records of this study. Every effort will be made to keep the subject’s personal medical data confidential.

The sponsor will protect individual subject information to the fullest extent possible during this study. At no time will a subject become identified in any publication or presentation.

However, the subject may have to become identified in the event of a regulatory authority auditor inspection in order to verify the accuracy of the data. Access to subject information is at the discretion of sponsor and cannot occur prior to database lock or other specified events as determined solely by the discretion of sponsor.

# 18. STUDY PROTOCOL AMENDMENTS

The Investigator will not make any changes to this protocol without prior written consent from sponsor and subsequent approval by the IRB/IEC. Any permanent change to the protocol, whether it is an overall change or a change for specific study center(s), must be handled as a protocol amendment. Any amendment to the protocol that appears necessary as the study progresses will be fully discussed by the Investigator(s) and sponsor. If agreement is reached

regarding the need for an amendment, it will be written by sponsor. The written amendment must be submitted to the chairman of the IRB/IEC identified with this responsibility. Except for administrative amendments, Investigators must await IRB/IEC approval of protocol amendments before implementing the change(s).

Administrative amendments are defined as having no effect on the safety of the research subjects, scope of the investigation, or quality of the study. However, a protocol change intended to eliminate an apparent immediate hazard to subjects should be implemented immediately, and the IRB/IEC notified within 5 days. The sponsor will ensure submission of any protocol amendments to the appropriate regulatory agencies.

When, in the judgment of the chairman of the local IRB/IEC, the Investigators, and/or sponsor, the amendment to the protocol substantially alters the study design and/or increases the potential risk to the subject; the currently approved written ICF will require similar modification. In such cases, the Investigator will obtain repeat informed consent from subjects enrolled in the study before expecting continued participation.

# 19. PUBLICATION

All information concerning the product as well as any information such as clinical indications for the study drugs, their formula, their formulation, methods of manufacture and other scientific data relating to it, that have been provided by sponsor or designee, and are unpublished, are confidential and must remain the sole property of Hua Medicine.

The Investigator will agree to use the information only for the purposes of carrying out this study and for no other purpose unless prior written permission from sponsor is obtained. The sponsor has full ownership of the eCRFs completed as part of the study.

By signing the study protocol, the Investigator agrees that the results of the study may be used for the purposes of national and international registration, publication, and information for medical and pharmaceutical professionals by sponsor. If necessary, the authorities will be notified of the Investigator’s name, address, qualifications, and extent of involvement.

The sponsor or designee will prepare a final report on the study. The Investigator may not publish or present any information on this study without first presenting the information to sponsor for review.

# 20. REFERENCES

1. Hua Medicine (Shanghai), Ltd. China: Investigator's Brochure for Dorzagliatin (HMS5552), Second edition, December 29, 2017.
2. Zhu D, Gan S, Liu Y, et. al. Dorzagliatin monotherapy in Chinese patients with type 2 diabetes: dose-ranging, randomised, double-blind, placebo-controlled, phase 2 study. Lancet Diabetes Endocrinol, 2018; 6 (8): 627-636.
3. [redacted] Package Insert, 2018, [redacted] USA
4. [redacted] Package Insert, 2018, [redacted] USA.
5. [redacted] Package Insert, 2017, [redacted] USA.
6. [redacted] [redacted] XR Package Insert, 2017, [redacted] USA.
7. [redacted] Package Insert, 2017, [redacted] USA.
8. [redacted] Package Insert, 2017, [redacted] USA.
9. [redacted] Package Insert, 2017, [redacted] USA.
10. Chung I, Oh J, Lee SH, et. al. A post hoc analysis of intra-subject coefficients of variation in pharmacokinetic measures to calculate optimal sample sizes for bioequivalence studies. Transl Clin Pharmacol, 2017; 25 (4): 179-182.

# APPENDIX A. LABORATORY ASSESSMENTS

| Hematology | Clinical Chemistry | Urinalysis |
|---|---|---|
| Hemoglobin (Hgb) | Blood urea nitrogen (BUN) | pH |
| Hematocrit (Hct) | Creatinine | Specific gravity |
| Platelet cout | Total bilirubin | Protein |
| Red blood cell (RBC) count | Alkaline phosphatase | Glucose |
| White blood cell (WBC) count | Aspartate transaminase (AST) | Ketones |
| | Alanine transaminase (ALT) | Bilirubin |
| with differential | Gamma-glutamyl | Blood |
| | transferase (GGT) | Nitrites |
| | Lactic dehydrogenase (LDH) | Leukocytes |
| | Glucose | Urobilinogen |
| | Albumin | Microscopic urine analysis |
| | Total protein | |
| | Bicarbonate | |
| | Phosphate | |
| | Sodium | |
| | Potassium | |
| | Chloride | |
| | Calcium | |
| | Total cholesterol | |
| | Triglyceride | |
| | HDL-C | |
| | LDL-C | |
| | Urate | |

| **Urine/Saliva Drug Screen** | **Serology Screen** |
|---|---|
| Amphetamines | Human immunodeficiency virus (HIV) |
| Barbiturates | Hepatitis B surface |
| Cannabinoids | antigen (HBsAg) |
| Cocaine metabolites | Hepatitis C virus (HCV) |
| Opiates | |
| Benzodiazepines | **Other** |
| Ethyl alcohol | Urine Pregnancy test (all women, screening, Day -1, End-of-Study) |
| | Serum FSH and estradiol (postmenopausal women, screening) |
| | HbA1c test (screening only) |
| | C-peptide test (screening only) |
| | **PD assessments** |
| | Glucose |
| | GLP-1 |
| | Glucagon |
| | Insulin |
| | C-peptide |

# TABLE OF CONTENTS

TABLE OF CONTENTS ........................................................................................................ 3
1. PROTOCOL SYNOPSIS ........................................................................................ 7
2. LIST OF ABBREVIATIONS ................................................................................ 14
3. INTRODUCTION ................................................................................................ 17
3.1 Background Information ................................................................................ 17
3.2 Preclinical Data ............................................................................................... 18
3.3 Summary of Clinical Studies .......................................................................... 19
3.4 Drug Interaction Potential of Dorzagliatin ..................................................... 22
4. STUDY RATIONALE AND OBJECTIVES .......................................................... 24
4.1. Study Rationale ............................................................................................... 24
4.2. Primary Objectives .......................................................................................... 24
4.3. Secondary Objective ....................................................................................... 25
5. INVESTIGATIONAL PLAN ................................................................................ 25
5.1. Overall Study Design ...................................................................................... 25
5.2. Number of Subjects ......................................................................................... 29
5.3. Treatment Assignment .................................................................................... 29
5.4. Criteria for Study Termination ........................................................................ 29
6. SELECTION AND WITHDRAWAL OF SUBJECTS .......................................... 29
6.1. Subject Inclusion Criteria ............................................................................... 29
6.2. Subject Exclusion Criteria .............................................................................. 31
6.3. Prohibitions and Restrictions .......................................................................... 33
6.4. Subject Withdraw Criteria .............................................................................. 33
7. STUDY PROCEDURES ...................................................................................... 34
7.1. Screening Visit and Run-in Period ................................................................. 35
7.2. Domiciled at CRC on Day -2 through Day 17 ................................................ 36
7.2.1. Study Check-in Visit: Day -2 and Day -1 ................................................... 36

7.2.2. Day 1 - Day 4 .......... 37

7.2.3. Day 5 .......... 38

7.2.4. Day 6 - Day 9 .......... 38

7.2.5. Day 10 .......... 39

7.2.6. Day 11 - Day 14 .......... 40

7.2.7. Day 15 .......... 40

7.2.8. Day 16 .......... 41

7.2.9. Day17: End-of Study Procedures .......... 41

7.3. Early Withdrawal Visit .......... 41

7.4. Time Windows for Procedures .......... 42

8. TREATMENT OF SUBJECTS .......... 42

8.1. Description of Study Drugs .......... 42

8.2. Methods of Assigning Subjects to Dose Groups .......... 43

8.3. Blinding .......... 43

8.4. Treatment Discontinuation/Subject Withdrawal .......... 43

8.5. Prior and Concomitant Medications and Substances .......... 43

8.6. Meals .......... 43

9. STUDY DRUG MATERIALS AND MANAGEMENT .......... 44

9.1. Study Drug Packaging and Labeling .......... 44

9.2. Study Drug Storage and Accountability .......... 44

9.3. Study Drug Preparation .......... 45

9.4. Study Drug Handling and Disposal .......... 45

10. PHARMACOKINETIC/PHARMACODYNAMIC ASSESSMENTS .......... 45

10.1. Pharmacokinetic Sample Collection and Storage .......... 45

10.2. Pharmacokinetic Specimen Labeling .......... 46

10.3. Pharmacokinetic Sample Shipping Instructions ..... 46
10.4. Bioanalytical Methodology ..... 47
10.5. Pharmacokinetic Parameters ..... 47
10.6. Pharmacodynamic Assessments ..... 47
11. ASSESSMENT OF SAFETY ..... 48
11.1. Safety Parameters ..... 48
11.1.1. Demographic/Medical History ..... 48
11.1.2. Vital Signs ..... 48
11.1.3. Physical Examination, Height, Weight, and Body Mass Index ..... 48
11.1.4. Electrocardiogram ..... 49
11.1.5. Clinical Laboratory Assessments ..... 49
11.2. Adverse Events ..... 51
11.2.1. Definitions of Adverse Events ..... 51
11.2.2. Recording Adverse Events ..... 54
11.2.3. Reporting of Serious Adverse Events ..... 54
11.2.4. Adverse Event Follow-up ..... 55
11.2.5. Hy's Law ..... 55
11.2.6. Hypoglycemia ..... 55
12. STATISTICAL METHODOLOGY ..... 56
12.1. Sample Size Determination ..... 56
12.2. Analysis Population ..... 56
12.3. Pharmacokinetics and Pharmacodynamics ..... 57
12.4. Demographic Characteristics ..... 58
12.5. Exposure to Study Drugs ..... 58
12.6. Concomitant Medications ..... 58

12.7. Safety Analyses ... 58

12.8. Interim Analyses ... 58

13. DIRECT ACCESS TO SOURCE DATA/DOCUMENTS ... 59

13.1. Study Monitoring ... 59

13.2. Sponsor's Responsibility ... 59

13.3. Audits and Inspections ... 59

14. QUALITY CONTROL AND QUALITY ASSURANCE ... 60

15. ETHICS ... 60

15.1. Ethics Review ... 62

15.2. Ethical Conduct of the Study ... 62

15.3. Written Informed Consent ... 63

16. DATA HANDLING AND RECORD KEEPING ... 63

16.1. Data Collection ... 63

16.2. Case Report Form Completion ... 64

16.3. Database Management, Data Clarification, and Quality Assurance ... 64

16.4. Inspection of Records/Source Documents ... 65

16.5. Retention of Records ... 66

17. CONFIDENTIALITY ... 66

17.1. Subject Data Protection ... 67

18. STUDY PROTOCOL AMENDMENTS ... 68

19. PUBLICATION ... 68

20. REFERENCES ... 70

APPENDIX A. LABORATORY ASSESSMENTS ... 71

# TABLES

Table 5-1 Schedule of Procedures ... 27

Table 5-2: Treatment Assignment and PK Sample Collection ... 29

Table 10-1: PK Terms ... 47

# 1. PROTOCOL SYNOPSIS

| | |
|---|---|
| **Name of Sponsor/Company:**<br>Hua Medicine (Shanghai) Ltd. | |
| **Name of Investigational Product:**<br>Dorzagliatin | |
| **Name of Active Ingredient:**<br>Dorzagliatin: (S)-2-[4-(2-Chloro-phenoxy)-2-oxo-2,5-dihydro-pyrrol-1-yl]-4-methyl-pentanoic acid [1-((R)-2,3-dihydroxy-propyl)-1H-pyrazol-3-yl]-amide | |
| **Title of Study:**<br>A Phase 1, Open-Label, Sequential, Multiple-Dose, Drug-Drug Interaction Study of Dorzagliatin and Sitagliptin in Subjects with Type 2 Diabetes Mellitus | |
| **Study Center(s):** | |
| **Site:** Frontage Clinical Services, Inc., Secaucus, NJ<br>**Principal Investigator:** [redacted]<br>**Sub-investigator:** [redacted] | **Site:** To be Determined |
| **Studied Period (years):**<br>Estimated date first subject enrolled: Dec 2018<br>Estimated date last subject completed: Mar 2019 | **Phase of development:** Phase 1 |
| **Objectives:**<br>**Primary:**<br>• To assess the potential pharmacokinetic interaction between dorzagliatin and sitagliptin in subjects with type 2 diabetes mellitus;<br>• To evaluate the safety and tolerability of dorzagliatin with simultaneous administration of sitagliptin in subjects with type 2 diabetes mellitus.<br>**Secondary:**<br>• To assess the pharmacodynamic responses of glucose, GLP-1, glucagon, insulin, and C-peptide in serum following dorzagliatin, sitagliptin, or simultaneous administration of dorzagliatin and sitagliptin in subjects with type 2 diabetes mellitus | |
| **Methodology:**<br>This is a Phase 1, open-label, sequential, multiple-dose, drug-drug interaction (DDI) study of glucokinase (GK) activator dorzagliatin and sitagliptin in subjects with type 2 diabetes mellitus (T2DM).<br>Study drugs will be administered in the following treatment scheme: | |

| Day | Sitagliptin | Dorzagliatin | PK Sample Collection |
|---|---|---|---|
| 1-5 | 100 mg (QD) for 5 days | - | Day 5:<br>Sitagliptin alone |
| 6-10 | 100 mg (QD) for 5 days | 75 mg (BID) for 4 days<br>Morning dose only on Day 10 | Day 10:<br>Sitagliptin + Dorzagliatin |
| 11-15 | - | 75 mg (BID) for 4 days<br>Morning dose only on Day 15 | Day 15:<br>Dorzagliatin alone |

Study drug will be taken 60 (±5) minutes prior to meals.

Eligible subjects will have a minimum 12-day run-in period prior to admission to the Clinical Research Center (CRC), during which time they will self-administer sitagliptin 100 mg QD each morning up until and including Day -2. Sitagliptin will be dispensed by the CRC. Subjects will complete a diary to record sitagliptin doses taken each day and will be advised to monitor their blood glucose levels and report changes in health status to the CRC. A telephone call will be placed to all subjects at approximately Day 6 (±2 day) of the run-in period to assess general health and collect adverse event (AE) information.

Following completion of the run-in period, eligible subjects will be admitted to the CRC on Day -2, have a total of 18 overnight stays, and be discharged after End-of-Study procedures are completed on Day 17, or at early termination.

Blood samples for the determination of dorzagliatin and sitagliptin concentrations and corresponding pharmacokinetic (PK) analysis will be collected at the following time points on Days 5, 10 and 15: pre-dose [redacted] and [redacted] post dose.

Subjects will consume a 75-gram glucose solution 30 minutes following study drug dose administration on Days 5, 10 and 15, in lieu of breakfast. Pharmacodynamic (PD) responses will be evaluated by measuring serum levels of glucose, Glucagon-like Peptide-1 (GLP-1), glucagon, insulin, and C-peptide within 60 minutes prior to oral glucose intake and 0.5, 1, 1.5, 2, 2.5, 3 and 4 hours after oral glucose intake.

Safety assessments will include monitoring of AEs, blood glucose via glucometer readings, vital signs (blood pressure, pulse rate, respiratory rate and oral temperature), clinical laboratory findings, resting 12-lead electrocardiograms (ECGs), and physical examination (PE) findings.

Vital signs will be assessed at screening, on Day -1, on Days 1-15 within 60 minutes prior to each morning and evening dose, and on Day 17. Blood glucose will be measured using a glucometer three times each day on Days -2 (prior to first meal after admission) through Day 16, within 60 minutes before each meal and after study drug dosing when applicable. Clinical laboratory evaluations (chemistry, hematology, urinalysis) will be performed at screening, on Days -1, 5, 10, 15 and 17. A resting 12-lead ECG will be completed at screening, on Day -1, and 2 hours post-dose on Days 5, 10, 15 and 17. Physical examinations will be conducted at screening, on Day -1, and on Day 17.

Subjects who terminate study participation early will have Day 17 assessments performed, if possible, prior to discharge from the CRC.

| **Number of Subjects (planned):** |
| --- |
| This study will plan to enroll up to 15 eligible subjects. Subjects may be enrolled singly or in groups. In order to have at least 12 evaluable subjects complete the study, additional subjects may be enrolled to replace subjects who discontinue study participation prematurely. |

**Inclusion Criteria:**

1. Subjects diagnosed with T2DM within at least 3 months prior to screening and are currently taking a stable dose of either of the following therapy with no change in the dose for at least 4 weeks prior to screening:
   a. ≥1000 mg per day of metformin
   b. a dipeptidyl peptidase 4 (DPP-4) inhibitor
   c. a sodium-glucose cotransporter-2 (SGLT2) inhibitor
   d. metformin plus a DPP-4 inhibitor
   e. metformin plus a SGLT2 inhibitor
2. Subjects must agree to change their current therapy to 100 mg sitagliptin QD for at least 14 days prior to dosing on Day 1;
3. In stable general health as determined by the Investigator at screening evaluation performed no more than 42 days prior to dosing on Day 1;
4. Male and/or female subjects between the ages of 30 and 65 years, inclusive;
5. Are capable of giving informed consent and complying with study procedures;
6. Body Mass Index (BMI) of 19 to 38 kg/$m^2$, inclusive, at screening;
7. Fasting C-peptide test result >0.3 nmol/L (>0.90 ng/mL);
8. HbA1c ≥7% and ≤10.5%;
9. Females must be using one of the following birth control methods: a non-hormonal intrauterine device (IUD) in place for at least 3 months; barrier method (condom, diaphragm) with spermicide for at least 14 days prior to dosing on Day 1 and throughout the study; surgical sterilization of the partner (vasectomy for 6 months minimum); OR are surgically sterile OR postmenopausal, as defined below:
   a. Surgically sterile for at least 3 months prior to screening by one of the following means:
      - Bilateral tubal ligation
      - Salpingectomy (with or without oophorectomy)
      - Surgical hysterectomy
      - Bilateral oophorectomy (with or without hysterectomy)
   b. Postmenopausal, defined as following:
      - Last menstrual period greater than 12 months prior to screening, confirmed by FSH and estradiol levels at screening;
10. Nonsmoker, defined as not having smoked or used any form of tobacco for more than 6 months prior to screening;
11. Willing and able to adhere to study restrictions and to be confined at the clinical research center.

**Exclusion Criteria:**

1. Fasting blood glucose at screening or Day -1 ≤110 or ≥270 mg/dL (one repeat screening fasting blood glucose allowed);
2. Type 1 diabetes mellitus, or latent autoimmune diabetes in adults; diabetic neuropathy, retinopathy or nephropathy;
3. Reported incidence of severe hypoglycemia, defined by denoting severe cognitive impairment requiring external assistance for recovery, within 3 months prior to screening and/or within 3 months prior to dosing on Day 1;
4. Known contraindications to sitagliptin;

5. Clinically significant gastrointestinal disorder (e.g., gastroparesis, constipation, diarrhea, colitis, gastrointestinal tract obstruction, hiatal hernia with reflux esophagitis, or cholestasis);
6. History or symptoms of clinically significant cardiovascular disease, particularly coronary artery disease, arrhythmias, atrial tachycardia, or congestive heart disease within one year prior to screening;
7. History of more than three urinary tract infections and/or more than three genital fungal infections in the last 12 months. If one or more genital fungal infections in the last 12 months required systemic treatment to resolve, Investigator will determine eligibility based on treatment required;
8. Reported history of clinically significant central nervous system disease including: transient ischemic attack, stroke, seizure disorder, depression, or behavioral disturbances within one year prior to screening;
9. Reported history of liver disease (e.g., hepatitis, cirrhosis within one year of screening and/or liver enzymes (alanine aminotransferase (ALT), aspartate aminotransferase (AST), gamma-glutamyltransferase (GGT), total bilirubin (TBiL)) >2 times the upper limit of normal (ULN));
10. Reported history of clinically significant renal disease;
11. Estimated glomerular filtration rate (eGFR) ≤60 mL/min/1.73m$^2$;
12. Acute or chronic metabolic acidosis, including diabetic ketoacidosis;
13. Known or suspected malignancy;
14. Any reported hypersensitivity (anaphylaxis and angioedema) or intolerance to sitagliptin;
15. Antidiabetic treatment with insulin, sulfonylureas, thiazolidinediones or GLP-1 agonist within 3 months prior to screening;
16. Systolic blood pressure <90 or ≥160 mmHg or diastolic blood pressure <60 or ≥100 mmHg at screening (may be repeated once);
17. A hospital admission or major surgery within 90 days prior to screening;
18. Uncontrolled hypertriglyceridemia >500 mg/dL;
19. Positive blood screen for human immunodeficiency virus (HIV), hepatitis B surface antigen (HBsAg), or hepatitis C antibody;
20. Positive pregnancy test result;
21. Female is breast-feeding or planning to become pregnant;
22. Treated with any investigational drugs within 6 weeks prior to screening;
23. Reported history of prescription drug abuse, or illicit drug use within 6 months prior to screening;
24. Reported history of alcohol abuse according to medical history within 6 months prior to screening;
25. A positive screen for alcohol or drugs of abuse;
26. Reported history of donation or blood collection of more than 1 unit (approximate 450 mL) of blood (or blood products) or acute loss of blood during the 90 days prior to screening;
27. Use of prescription or over-the-counter (OTC) medications, and herbal (including St John’s Wort, herbal teas, garlic extracts) within 14 days prior to dosing (except for sitagliptin dosing during run-in period). No angiotensin-converting enzyme (ACE) inhibitors are allowed; (Note: Use of acetaminophen at <1 g/day is permitted until 24 hours prior to dosing);
28. An unwillingness of male participants to use appropriate contraceptive measures if engaging in sexual intercourse with a female partner of childbearing potential. Appropriate measures include use of a condom and spermicide and, for female partners, use of an IUD, diaphragm with spermicide, oral contraceptives, injectable progesterone, progesterone subdermal implants, or a tubal ligation. Sexual intercourse with pregnant or lactating women is prohibited.

**Study Drugs, Dosage and Mode of Administration:**

Dorzagliatin will be provided as a film-coated tablets in 75 mg strength for oral administration 60 minutes prior to a meal.

Sitagliptin will be provided as [redacted]® 100 mg tablets for oral administration 60 minutes prior to a meal.

**Duration of Treatment:**

The total duration of participation in the study for each subject is about 59 days (up to 28-day screening period, 12-day run-in period and 19-day in-clinic period).

**Criteria for Evaluation:**

**Safety:**

Safety assessments will include monitoring of AEs, blood glucose via glucometer readings, vital signs (blood pressure, pulse rate, respiratory rate and oral temperature), clinical laboratory findings, 12-lead ECGs, and PE findings.

**Pharmacokinetics:**

The plasma concentration-time data for dorzagliatin and sitagliptin will be analyzed using non-compartmental methods to calculate pharmacokinetic parameters. Actual dosing and sampling times will be used for analyses. The primary PK parameters of interest are: $C_{max}$, $T_{max}$ and $AUC_{0\text{-}24h}$. Additional parameters may be estimated and reported, as appropriate.

**Pharmacodynamics:**

Pharmacodynamic (PD) responses will be evaluated by measurement of serum levels of glucose, GLP-1, glucagon, insulin and C-peptide. The PD parameters may include, but not limited to, $AUEC_{0\text{-}4h}$, $CE_{max}$ and $CE_{av}$.

**Statistical Methods:**

Baseline demographic, concentration and safety data will be listed and summarized by descriptive statistics (n, mean, standard deviation, median, minimum, maximum) or frequency counts. Descriptive statistics will be calculated for dorzagliatin and sitagliptin concentrations in plasma.

Dorzagliatin and sitagliptin PK parameters ($C_{max}$, $T_{max}$ and $AUC_{0\text{-}24h}$) will be computed for each subject using non-compartmental methods based on the actual sampling times. Descriptive statistics will be calculated for the PK parameters for Days 5, 10 and 15.

The effect of sitagliptin on the exposure of dorzagliatin will be determined by a PK comparison between the exposure of dorzagliatin on Day 10 vs. Day 15. The effect of dorzagliatin on the exposure of sitagliptin will be determined by a PK comparison between the exposure of sitagliptin on Day 10 vs. Day 5.

The primary PK comparison will be based on the $C_{max}$ and $AUC_{0\text{-}24h}$ of dorzagliatin and sitagliptin. A mixed model under the sequential design will be used for analyzing $C_{max}$ and $AUC_{0\text{-}24h}$ values, based on natural log-transformed values, with treatments as fixed effects and subjects as random effects. The adjusted geometric mean ratios between two treatments for these parameters and the corresponding 90% confidence intervals (CI) for the ratio will be obtained by exponentiating the adjusted mean difference in logarithms and will be used for assessing PK differences between the two treatments within each PK comparison.

Baseline corrected PD markers will be calculated by subtracting the baseline value from the values post oral glucose intake. Plots of mean PD markers versus the scheduled time will be generated for each treatment. Derivations and statistical analysis methods of PD parameters ($AUEC_{0\text{-}4h}$, $CE_{max}$ and $CE_{av}$) will be described in detail in Statistical Analysis Plan (SAP).

| |
|---|
| This study will be conducted in accordance with the guidelines of Good Clinical Practices (GCPs) including archiving of essential documents. |

# 2. LIST OF ABBREVIATIONS

The following abbreviations and specialist terms are used in this study protocol.

| Abbreviation | Term |
|---|---|
| ACE | Angiotensin-Converting Enzyme |
| AE | Adverse Event |
| ALT | Alanine Aminotransferase |
| ANOVA | Analysis of Variance |
| API | Active Pharmaceutical Ingredient |
| AST | Aspartate Aminotransferase |
| AUC | Area Under the Concentration-Time Curve |
| $AUC_{0-24h}$ | Area Under the Concentration-Time Curve from 0 to 24 Hours |
| $AUEC_{0-4h}$ | Area Under the Effect-Time Curve of Change from Baseline |
| BID | Twice Daily |
| BMI | Body Mass Index |
| BP | Blood Pressure |
| BUN | Blood Urea Nitrogen |
| $C_{max}$ | Observed Maximum Plasma Concentration |
| $CE_{av}$ | Average Change from Baseline |
| $CE_{max}$ | Maximum Change from Baseline |
| CFDA | China Food and Drug Administration |
| CFR | Code of Federal Regulations |
| CI | Confidence Interval |
| CL/F | Apparent Total Plasma Clearance of Drug after Oral Administration |
| CNS | Central Nervous System |
| CRC | Clinical Research Center |
| CRO | Contract Research Organization |
| CYP | Cytochrome P450 |
| DDI | Drug Drug Interaction |
| DMP | Data Management Plan |
| DPP-4 | Dipeptidyl Peptidase-4 |
| ECG | Electrocardiography |
| eCRF | Electronic Case Report Form |
| eGFR | Estimated Glomerular Filtration Rate |
| EOS | End-of-Study |
| FDA | Food and Drug Administration |

| Abbreviation | Term |
|---|---|
| FSH | Follicle Stimulating Hormone |
| GCP | Good Clinical Practice |
| GGT | Gamma-Glutamyl-Transferase |
| GK | Glucokinase |
| GKA | Glucokinase Activator |
| GLP | Good Laboratory Practice |
| GLP-1 | Glucagon-Like Peptide 1 |
| GMP | Good Manufacturing Practice |
| GSIR | Glucose Stimulated Insulin Release |
| HbA1c | Hemoglobin A1c |
| HBsAg | Hepatitis B Surface Antigen |
| Hct | Hematocrit |
| HCV | Hepatitis C Virus |
| HDL | High-Density Lipoprotein |
| hERG | Human Ether-a-go-go-Related Gene |
| Hgb | Hemoglobin |
| HIV | Human Immunodeficiency Virus |
| HLM | Human Liver Microsome(s) |
| IB | Investigational Brochure |
| $IC_{50}$ | The Half Inhibitory Concentration |
| ICF | Informed Consent Form |
| ICH | International Conference on Harmonisation |
| IEC | Independent Ethics Committee |
| IND | Investigational New Drug Application |
| IRB | Institutional Review Board |
| IUD | Intrauterine Device |
| $K_{el}$ | Elimination Rate Constant |
| LC-MS/MS | Liquid Chromatography-Tandem Mass Spectrometry |
| LDH | Lactate Dehydrogenase |
| LDL | Low-Density Lipoprotein |
| MAD | Multiple Ascending Dose |
| MATE | Multidrug and Toxin Extrusion |
| MedDRA | Medical Dictionary for Regulatory Activities |
| MTD | Maximum Tolerated Dose |
| NOAEL | No Observed Adverse Effect Level |
| OAT | Organic Anion Transporter |

| Abbreviation | Term |
|---|---|
| OATP | Organic Anion Transporting Polypeptide |
| OGTT | Oral Glucose Tolerance Test |
| OTC | Over-The-Counter |
| P-gp | P-Glycoprotein |
| PHI | Protected Health Information |
| PIS | Patient Information Sheet |
| PK | Pharmacokinetic(s) |
| QC | Quality Control |
| QD | Once Daily |
| QTc | Corrected QT Interval |
| RBC | Red Blood Cell |
| $S_{0.5}$ | Substrate Concentration to Give ½ $V_{max}$ |
| SAD | Single Ascending Dose |
| SAE | Serious Adverse Event |
| SAP | Statistical Analysis Plan |
| SDV | Source Data Verification |
| SGLT2 | Sodium-Glucose Cotransporter-2 |
| SOP | Standard Operating Procedure |
| $T_{1/2}$ | Terminal Elimination Half-Life |
| T2DM | Type 2 Diabetes Mellitus |
| TBiL | Total Bilirubin |
| TDI | Time Dependent Inhibition |
| TEAE | Treatment-Emergent Adverse Event |
| $T_{max}$ | Time at which $C_{max}$ was first observed |
| ULN | Upper Limit of Normal |
| $V_{max}$ | Maximum Metabolic Rate |
| $V_z/F$ | Apparent Volume of Distribution during Terminal Phase after Oral Administration |
| WBC | White Blood Count |
| WHO | World Health Organization |

# 3. INTRODUCTION

## 3.1 Background Information

Hua Medicine (Shanghai) Ltd. (hereinafter "sponsor" or "Hua Medicine") is developing dorzagliatin, an investigational novel glucokinase activator (GKA), indicated for use as an adjunct to diet and exercise to improve glycemic control in adults with type 2 diabetes mellitus (T2DM).

Glucokinase (GK) activators represent a promising new class of investigational drugs for the treatment of T2DM. Glucokinase activators lower blood glucose levels by enhancing the ability of pancreatic β-cells to "sense glucose" and increase insulin secretion in a glucose dependent manner. Simultaneously, GKAs can suppress glucose production and increase glucose utilization in the liver. Glucokinase activators may also function through other GK-expressing cells, such as entero-endocrine K and L-cells, and many GKAs have been shown to exert anti-apoptotic effects on β-cells.

Dorzagliatin (also referred to as HMS5552) is the 4$^{th}$ generation of GKAs. Dorzagliatin is an allosteric activator of GK which has been shown to increase the affinity of its substrate glucose by decreasing $S_{0.5}$ and increasing the $V_{max}$ of GK. Dorzagliatin has only a minor effect on the Hill coefficient nH and preserves the positive cooperativity of GK for glucose, a unique kinetic feature of GK. Dorzagliatin enhances glucose stimulated insulin release (GSIR) in rodent pancreatic islets and increases glucose uptake in cultured rodent primary hepatocytes. The selectivity profile of dorzagliatin was evaluated by screening 402 Ambit protein kinases and 78 CEREP receptors. In all cases the $IC_{50}$ values were > 10 µM, which was the highest concentration tested.[1]

Dorzagliatin is a potent glucose lowering agent showing excellent dose-related effects on fasting, basal and post-prandial glucose levels in several rodent models of T2DM, both in acute as well as chronic studies. Dorzagliatin augments GSIR and improves hepatic glucose disposal *in vivo*. Furthermore, treatment of normal mice with dorzagliatin has been associated with an increase in the levels of total Glucagon-like Peptide-1 (GLP-1). Therefore, dorzagliatin is a potential new antidiabetic agent for use as an adjunct to diet and exercise to improve glycemic control in adults with T2DM.

## 3.2 Preclinical Data

Preclinical evaluations including safety pharmacology, general toxicology studies in rats and dogs, reproductive toxicology, genetic toxicology were conducted following the guidance of International Conference on Harmonisation (ICH) and China Food and Drug Administration (CFDA), in compliance with the U.S. and CFDA Good Laboratory Practices (GLP) regulations. [1]

No QTc prolongation or dorzagliatin-related qualitative electrocardiographic events or abnormalities were observed at dose level up to [redacted] of dorzagliatin in safety pharmacology studies using telemetry technology in conscious dogs. [redacted] No adverse effect on central nervous system or respiratory system was observed in rats at [redacted] dorzagliatin (maximum dose).[1]

The repeat-dose toxicity and toxicokinetic profiles for orally administered dorzagliatin have been characterized in rats for 4 weeks, 13 weeks and 26 weeks and in dogs for 4 weeks, 13 weeks, and 39 weeks. [redacted] No adverse effect (including peripheral neuropathy) was found in dogs after dorzagliatin treatment [redacted]

No adverse effect on male reproductive organs and reproductivity was found in male fertility study [redacted]. [redacted]

[redacted] In the later phase of clinical trials, exclusive criteria have included the subjects who might potentially be pregnant.

No evidence for genotoxicity or mutagenicity of dorzagliatin was identified using in vitro Ames test and chromosomal aberration assay in human peripheral blood lymphocytes, and in vivo micronucleus test in rat bone marrow.[1]

These study results indicate that dorzagliatin has an adequate safety margin to support its development in the clinical setting.

## 3.3 Summary of Clinical Studies

To date, five clinical studies evaluating dorzagliatin have been completed, four of which were Phase 1 studies and one was a Phase 2 study. A total of 335 subjects have been exposed to dorzagliatin.[1]

Study HMM0101: In a Phase 1 Single Ascending Dose (SAD) study, a total of 48 healthy subjects received single oral doses of HMS5552 ranging from 5 mg to 50 mg. HMS5552 appeared to be safe and was well tolerated at all doses studied. No serious adverse events (SAEs) were reported and no withdrawal occurred due to an adverse event (AE). All AEs were mild in intensity and no treatment was required. [redacted]

$C_{max}$ and AUC were apparently proportional to dose. No marked gender difference of $C_{max}$ and AUC was noticed, and the PK profile of HMS5552 indicated it was suitable for twice daily (BID) administration.

Study HMM0102: In a Phase 1 Multiple Ascending Dose (MAD) study, a total of 43 T2DM subjects received multiple twice-daily oral doses of HMS5552 ranging from 25 mg to 200 mg for 8 days. HMS5552 appeared to be safe and was well tolerated at all doses. There was no apparent sign or change in the pattern of any clinical laboratory value, vital signs or ECG parameters. All AEs were mild in intensity. No severe AE or SAE was reported. The most common AEs were related to mild hypoglycemia. No subjects reported serious hypoglycemia. All subjects recovered quickly without requiring additional intervention. The PK of HMS5552 appeared to be dose-proportional over the range tested without appreciable drug accumulation or food-effect. [redacted]

[redacted]

Study HMM0103: In a Phase 1c study, 24 T2DM subjects received daily doses of 75 mg or 150 mg (75 mg BID) HMS5552 daily for 4 weeks. HMS5552 displayed excellent safety and tolerability in subjects who participated in the study. There were no incidents of SAE, death, early termination due to AEs or severe hypoglycemia. All AEs were considered mild in intensity. No clinically significant changes were observed in laboratory, 12-lead electrocardiogram (ECG) or physical examination (PE) tests. Consistent PK Profiles were observed as in the MAD study.

[redacted] renal excretion was not the major elimination pathway of HMS5552. [redacted]

Functions of pancreatic β-cells were improved in both groups 3 days after the 28-day treatment ended, compared with baseline. The sensitivity of pancreatic β-cells to blood glucose was enhanced after 7-8 drug $T_{1/2}$ had passed.

Study HMM0104: In a Phase 1 drug-drug interaction (DDI) study conducted in the US, 15 T2DM subjects received HMS5552 50 mg BID or metformin 500 mg BID alone or in combination for a total of 13 days (metformin alone for 3 days, dorzagliatin and metformin co-administration for 5 days, then dorzagliatin alone for 5 days). It appeared treatment with HMS5552 50 mg BID alone or co-administration with metformin 500 mg BID was safe and well-tolerated. There were no incidents of SAE, death, or early termination due to AEs. All treatment-emergent adverse events (TEAEs) were mild in intensity and subjects recovered after intervention. There was no clear evidence to support the causal relationship between the study drug and TEAEs, and no incidence of hypoglycemic events. No clinically significant changes were observed in laboratory tests, vital signs, ECGs or PEs in this study. Combined treatment

with metformin did not affect the PK of HMS5552. [redacted] Overall, the study demonstrated that there is no apparent clinically significant DDI between HMS5552 and metformin. In contrast, the combined treatment resulted in improved glycemic control in T2DM subjects compared to treatment with HMS5552 or metformin alone.

Study HMM0201: A dose-ranging, randomized, double-blind, placebo-controlled, Phase 2 study was conducted in Chinese patients with T2DM, aiming to identify a minimum effective dose of dorzagliatin in patients with type 2 diabetes.[1,2]

Two hundred fifty-eight (258) T2DM patients enrolled in the Phase 2 study and were randomly assigned to receive placebo (n=53), 75 mg dorzagliatin once daily (n=53), 100 mg dorzagliatin once daily (n=50), 50 mg dorzagliatin twice daily (n=51), or 75 mg dorzagliatin twice daily (n=51) for 12 weeks.

No deaths, drug-related SAEs, or drug-related severe AEs were reported. Most AEs were mild and considered unrelated to study medication by investigators. The incidence of AEs was similar among groups. Adverse events that occurred in ≥5% of patients in any group (including placebo) were upper respiratory tract infection, hyperuricemia, dizziness, protein present in urine, urinary tract infection, blood creatine phosphokinase increased, white blood cells (WBC) urine positive, hepatic function abnormal, high-density lipoprotein (HDL) decreased, ventricular extrasystole and nasopharyngitis. Incidence of hypoglycemia was low, with a rate of 5.4% for ≤3.9 mmol/L, and a rate of only 1% for ≥3.0 mmol/L in the Phase 2 study in the drug-treated groups. No severe hypoglycemia was reported.

[redacted]

In conclusion, preclinical pharmacology studies demonstrated that HMS5552 is an effective allosteric activator of GK both *in vitro* and *in vivo*. Safety pharmacology, general toxicity studies

in rats and dogs up to 26 weeks and 39 weeks, reproductive toxicology, genetic toxicology and carcinogenicity studies suggested that HMS5552 has an acceptable safety profile when projected for human use. Furthermore, safety data from clinical phase 1 and phase 2 studies conducted in China and US support the continued development of HMS5552.

## 3.4 Drug Interaction Potential of Dorzagliatin

In *in vitro* studies using human liver microsomes (HLM) dorzagliatin showed no inhibition of liver cytochrome P450 (CYP) 1A2, CYP2A6, CYP2C9, CYP2C19, CYP2D6, CYP2E1 and CYP3A4/5 activities ($IC_{50}$ >50 µM) at concentrations studied.

Drug interaction potential of dorzagliatin via CYP induction was evaluated in primary human hepatocytes and results indicated that dorzagliatin was unlikely to produce induction on CYP1A2 or CYP2B6. While an absence of induction of dorzagliatin on CYP3A4 could not be excluded from this vitro study, it was later confirmed in preclinical and clinical PK studies as no decrease in exposure of dorzagliatin was observed after repeated dosing.

*In vitro* results suggest that dorzagliatin is a substrate of P-glycoprotein (P-gp) but not a P-gp inhibitor. In addition, dorzagliatin did not show clinically relevant inhibitory effect on organic anion transporting polypeptide (OATP)1B1, OATP1B3, organic anion transporter (OAT)1 and OAT3, nor is dorzagliatin a substrate of these four transporters. These study results demonstrate that transporter-related DDI is unlikely.

Since dorzagliatin is predominantly metabolized by CPY3A4 and is a substrate of P-gp, it is predicted that exposure of dorzagliatin will be increased when co-administered with CYP3A4 inhibitors and decreased when co-administered with CYP3A4 inducers.[1]

Dipeptidyl peptidase-4 (DPP-4) inhibitors are a class of oral drugs for the treatment of patients with T2DM who have not responded well to drugs such as metformin and sulfonylureas. DPP-4 inhibitors block the action of DPP-4, an enzyme that destroys incretins. Incretins are gastrointestinal hormones that help stimulate insulin production. FDA-approved DPP-4 inhibitors include sitagliptin ([redacted]), saxagliptin [redacted] and linagliptin [redacted]. The reported half-lives of sitagliptin, saxagliptin (and active metabolite) and linagliptin are 12.4, 2.5 (and 3.1), and 12 hours (effective half-life) respectively.[3,4,5]

Sitagliptin is mainly (79%) cleared as parent drug by urine with metabolism being a minor pathway of elimination. In vitro studies indicated that the primary enzyme responsible for the limited metabolism of sitagliptin was CYP3A4, with additional contribution from CYP2C8. Sitagliptin is a substrate for OAT 3, which may be involved in the renal elimination of sitagliptin.

Sitagliptin is not an inhibitor of CYP3A4, 2C8, 2C9, 2D6, 1A2, 2C19 or 2B6, and is not an inducer of CYP3A4. Sitagliptin is a P-gp substrate but does not inhibit P-gp mediated transport of digoxin. Based on these results sitagliptin is considered unlikely to cause interactions with other drugs that utilize these pathways. Sitagliptin is not extensively bound to plasma proteins. Therefore, the propensity of sitagliptin to be involved in clinically meaningful DDIs mediated by plasma protein binding displacement is very low.[3]

Collectively, the available PK and metabolic information for dorzagliatin and sitagliptin summarized above suggest that the DDI potential between these two drugs when co-administered is low.

In this DDI study, prior to enrollment subjects must be taking a stable dose of either metformin alone, metformin in combination with a marketed brand of a sodium-glucose cotransporter-2 (SGLT2) inhibitor or a DPP-4 inhibitor, or a marketed brand of an SGLT2 or DPP-4 inhibitor as monotherapy.

Metformin [redacted] is an oral antihyperglycemic drug that is commonly recommended as the first-line pharmacotherapy for treatment of T2DM. Mean time to peak plasma concentration ($T_{max}$) following administration of a metformin tablet is about 2-3 hours. Renal excretion is the major route of metformin elimination. Following oral administration, approximately 90% of the absorbed drug is eliminated via the renal route within the first 24 hours, with a plasma elimination half-life of approximately 6.2 hours.[6]

Sodium-glucose cotransporter-2 inhibitors are a class of oral drugs for the treatment of patients with T2DM that lower blood sugar by blocking SGLT2, a glucose transporter in the kidney, and preventing the kidney from reabsorbing glucose and releasing it into the blood. FDA-approved SGLT2 inhibitors include canagliflozin [redacted], dapagliflozin [redacted] and empagliflozin [redacted]. The reported half-lives of canagliflozin, dapagliflozin and empagliflozin are 10-13.1, 12.9 and 12.4 hours, respectively.[7,8,9]

# 4. STUDY RATIONALE AND OBJECTIVES

## 4.1. Study Rationale

Although the available drug interaction and PK data of dorzagliatin and sitagliptin detailed in Section 3.4 do not suggest a potential drug interaction, other drug transporter-mediated interactions cannot be ruled out. Furthermore, pharmacodynamic-based interaction potential needs to be evaluated. Based on the high likelihood that dorzagliatin may be co-administered with sitagliptin in T2DM patients in a clinical setting, this study will provide clinical evidence of the DDI potential between these two drugs.

The basis for the 100 mg QD dosing regimen of sitagliptin is based on the recommended 100 mg once daily dosing of [redacted] for adults.[3] The 75 mg BID for dorzagliatin is the dose regimen proven to be safe and effective in Phase 1 and Phase 2 studies and selected for two current Phase 3 studies evaluating dorzagliatin as a mono-therapy and as an add-on treatment to metformin. In the present study, both drugs will be given for five days to ensure that steady-state is attained.

The sequential, multiple-dose study is designed to determine whether the steady-state pharmacokinetics of dorzagliatin and sitagliptin are affected, while at the same time whether there's a synergistic therapeutic effect between these two drugs when co-administered. The proposed dosing regimens with dorzagliatin and sitagliptin will allow the potential interactions to be assessed under conditions that are expected to provide maximal exposure at the doses being studied. The elimination half-lives of dorzagliatin and sitagliptin are relatively short which justifies the 24-hour interval for collecting all samples to characterize the pharmacokinetics of both drugs.

## 4.2. Primary Objectives

The primary objectives of this study are:

- To assess the potential pharmacokinetic interaction between dorzagliatin and sitagliptin in subjects with type 2 diabetes mellitus;
- To evaluate the safety and tolerability of dorzagliatin with simultaneous administration of sitagliptin in subjects with type 2 diabetes mellitus.

### 4.3. Secondary Objective

The secondary objective of this study is:

- To assess the pharmacodynamic responses of glucose, GLP-1, glucagon, insulin, and C-peptide in serum following dorzagliatin, sitagliptin, or simultaneous administration of dorzagliatin and sitagliptin in subjects with type 2 diabetes mellitus.

## 5. INVESTIGATIONAL PLAN

### 5.1. Overall Study Design

This is a Phase 1, open-label, sequential, multiple-dose, DDI study of GK activator dorzagliatin and sitagliptin in subjects with T2DM.

It is planned that 15 subjects will be enrolled to have at least 12 evaluable subjects. All subjects will receive:

- Sitagliptin 100 mg QD in the morning on Days 1-5;
- Sitagliptin 100 mg QD in the morning and dorzagliatin 75 mg BID (morning and evening) on Days 6-10, with only the morning dose on Day 10;
- Dorzagliatin 75 mg BID (morning and evening) on Days 11-15, with only the morning dose on Day 15.

Eligible subjects will have a minimum 12-day run-in period prior to admission to the CRC, during which time they will self-administer sitagliptin 100 mg QD up until and including Day -2. Sitagliptin will be dispensed by the CRC. Sitagliptin will be purchased and provided by the sponsor. Subjects will complete a diary to record sitagliptin doses taken each day and will be advised to monitor their blood glucose levels and report changes in health status to the CRC. A telephone call will be placed to all subjects at approximately Day 6 (±2 day) of the run-in period to assess general health and collect AE information.

Following completion of the run-in period, eligible subjects will be admitted to the CRC on Day -2, have a total of 18 overnight stays, and be discharged after End-of-Study procedures are completed on Day 17, or at early termination.

Blood samples for the determination of dorzagliatin and sitagliptin concentrations and corresponding PK analysis will be collected at the following time points on Days 5, 10 and 15: [redacted].

Subjects will consume a 75-gram glucose solution 30 minutes following study drug dose administration on Days 5, 10 and 15, in lieu of a breakfast. Pharmacodynamic responses will be evaluated by measuring serum levels of glucose, GLP-1, glucagon, insulin, and C-peptide at baseline (prior to the study drug dose and within 60 minutes prior to oral glucose intake) and 0.5, 1, 1.5, 2, 2.5, 3 and 4 hours after oral glucose intake.

Safety assessments will include monitoring of AEs, blood glucose via glucometer readings, vital signs (blood pressure (BP), pulse rate (PR), respiratory rate (RR) and oral temperature (T)), clinical laboratory findings, resting ECGs, and PE findings.

Vital signs will be assessed at screening, on Day -1, on Days 1-15 within 60 minutes prior to each morning and evening dose, and on Day 17. Blood glucose will be measured using a glucometer three times each day on Days -2 (prior to first meal after admission) through Day 16, within 60 minutes before each meal and after study drug dosing when applicable. Clinical laboratory evaluations (chemistry, hematology, urinalysis) will be performed at screening, on Days -1, 5, 10, 15 and 17. A resting 12-lead ECG will be completed at screening, on Day -1, and 2 hours post-dose on Days 5, 10, 15, and 17. Physical exams will be conducted at screening, on Day -1, and on Day 17.

Subjects who receive their Day 1 dose and then terminate study participation early will have Day 17 assessments performed, if possible, prior to discharge from the CRC.

Morning dosing will occur at approximately 08:00 a.m. and evening dosing will occur at approximately 06:00 p.m. All doses will be administered 60 (±5) minutes prior to a standardized meal and with approximately 240 mL (8 fluid ounces) of room temperature water. On Days 5, 10 and 15 there will be no meal offered after the morning dose and instead the oral glucose solution will be administered 30 minutes after the study drug dose. The actual time of each dose, each post-dose meal, and time of oral glucose solution administration will be recorded.

See Table 5-1 for the details of all study procedures for subjects in the study.

**Table 5-1 Schedule of Procedures**

| **Visit** | **Screening** | **Run-in*** | **In-patient at the CRC*** | | | | | | | | | |
|---|---|---|---|---|---|---|---|---|---|---|---|---|
| **Day** | **-42 to -15** | **-14 to -3** | **-2** | **-1** | **1-4** | **5** | **6-9** | **10** | **11-14** | **15** | **16** | **17 EOS** |
| Informed consent | X | | | | | | | | | | | |
| Dispense/collect sitagliptin, at-home sitagliptin QD 100 mg. Tel call at Day -8±2 (Day 6 of the 12-day run-in period) | | X | X | | | | | | | | | |
| Admission to CRC | | | X | | | | | | | | | |
| Eligibility assessment | X | | | X | | | | | | | | |
| Demographics | X | | | | | | | | | | | |
| Medical history | X | | X | | | | | | | | | |
| Physical examination | X | | | X | | | | | | | | X |
| Height (cm), Weight (kg) | X | | | X[1] | | | | | | | | X[1] |
| Body mass index | X | | | | | | | | | | | |
| Standard 12-lead ECG[2] | X | | | X | | X | | X | | X | | X |
| Vital signs (BP, PR, RR, T) | X | | | X | Within 60 min prior to each study drug dose. | | | | | | | X |
| Clinical laboratory samples[3] | X | | | X | | X | | X | | X | | X |
| HIV, hepatitis B & C | X | | | | | | | | | | | |
| Urine drug /saliva alcohol | X | | X | | | | | | | | | |
| Urine pregnancy test[4] | X | | X | | | | | | | | | X |
| FSH and estradiol[4] | X | | | | | | | | | | | |
| OGTT/PD markers [5] | | | | | | X | | X | | X | | |
| C-peptide and HbA1c | X | | | | | | | | | | | |
| Dose administration[6] | | | | Sitagliptin 100 mg QD on Day -1; Assigned treatment Days 1-15 | | | | | | | | |
| AE & con med reporting[7] | | X | X | X | X | X | X | X | X | X | X | X |
| PK blood samples[8] | | | | | | X | X | X | X | X | X | |
| Glucometer (finger stick) | | | Within 60 minutes before each meal and after study drug dosing when applicable | | | | | | | | | |
| Discharge from CRC | | | | | | | | | | | | X |

AE= adverse event; BP = blood pressure; con med = concomitant medications; CRC = clinical research center; ECG = electrocardiogram; EOS =End-of-Study; FSH: Follicle stimulating hormone; HIV = human immunodeficiency virus; PR = pulse rate; PK = pharmacokinetics; RR = respiratory rate; T = temperature.

*Run-in period is a minimum of 12 days. If longer than 12 days, admission to CRC remains on Day -2.

[1]Obtain weight only.

[2]ECG obtained in supine position after at least 5 minutes rest. On Days 5, 10 and 15 ECGs obtained 2 hours (±15 minutes) after dose administration.

[3]Clinical laboratory samples include: hematology, chemistry and urinalysis. All samples collected prior to study drug dose on days of study drug administration.

[4]For female subjects, urine pregnancy test must be negative to enroll in the study. Serum FSH and estradiol will be evaluated for postmenopausal females to confirm status.

[5] Oral glucose solution administered 30 (±5) minutes after study drug dose on Days 5, 10 and 15. PD samples to measure serum levels of glucose, GLP-1, glucagon, insulin, and C-peptide collected at baseline (prior to the study drug dose and within 60 minutes prior to oral glucose intake) and at 0.5, 1, 1.5, 2, 2.5, 3 and 4 hours after oral glucose intake. All post-glucose collection times are ±5 minutes from nominal time.

[6]All subjects will receive: sitagliptin 100 mg QD on Days 1-5 (a.m. dose); sitagliptin 100 mg QD and dorzagliatin 75 mg BID on Days 6-10 (a.m. dose only on Day 10); and dorzagliatin 75 mg BID on Days 11-15 (a.m. dose only on Day 15). Morning dosing will occur at approximately 08:00 a.m. and evening dosing will occur at approximately 06:00 p.m. All doses are 60 (±5) minutes prior to meals except for Days 5, 10 and 15 morning doses when no meals are offered following the study drug dose. Subjects are to resume their regular medication on Day 16.

[7]SAE collection starts after signing ICF.

[8]PK samples collected [redacted] on Days 5, 10 and 15. Blood collection time windows: ±5 minutes from 0-4 hours post-dose and ±10 minutes ≥6 hours post-dose.

## 5.2. Number of Subjects

This study will plan to enroll up to 15 eligible subjects. Subjects may be enrolled singly or in groups. In order to have at least 12 evaluable subjects complete the study, additional subjects may be enrolled to replace subjects who discontinue study participation prematurely. Evaluable subjects are defined as subjects included in the PD population as described in Section 12.2.

## 5.3. Treatment Assignment

All subjects will receive the same treatment assignment as follows:

**Table 5-2: Treatment Assignment and PK Sample Collection**

| Day | Sitagliptin | Dorzagliatin | PK Sample Collection |
|---|---|---|---|
| 1-5 | 100 mg (QD) for 5 days | - | Day 5:<br>Sitagliptin alone |
| 6-10 | 100 mg (QD) for 5 days | 75 mg (BID) for 4 days<br>Morning dose only on Day 10 | Day 10:<br>Sitagliptin + Dorzagliatin |
| 11-15 | - | 75 mg (BID) for 4 days<br>Morning dose only on Day 15 | Day 15:<br>Dorzagliatin alone |

## 5.4. Criteria for Study Termination

The Investigator has the right to terminate the study in the interest of subject safety and welfare in consultation with sponsor. Additionally, the sponsor reserves the right to terminate or amend the study at any time for administrative reasons or if continuation of the protocol would present a potential safety risk to the subjects.

# 6. SELECTION AND WITHDRAWAL OF SUBJECTS

## 6.1. Subject Inclusion Criteria

To participate in the study, subjects must meet all the following eligibility criteria at screening:

1. Subjects diagnosed with T2DM within at least 3 months prior to screening and are currently taking a stable dose of either of the following therapy with no change in the dose for at least 4 weeks prior to screening:
   a. ≥1000 mg per day of metformin

b. a dipeptidyl peptidase 4 (DPP-4) inhibitor
c. a sodium-glucose cotransporter-2 (SGLT2) inhibitor
d. metformin plus a DPP-4 inhibitor
e. metformin plus a SGLT2 inhibitor

2. Subjects must agree to change their current therapy to 100 mg sitagliptin QD for at least 14 days prior to dosing on Day 1;
3. In stable general health as determined by the Investigator at screening evaluation performed no more than 42 days prior to dosing on Day 1;
4. Male and/or female subjects between the ages of 30 and 65 years, inclusive;
5. Are capable of giving informed consent and complying with study procedures;
6. Body Mass Index (BMI) of 19 to 38 kg/m$^2$, inclusive, at screening;
7. Fasting C-peptide test result >0.3 nmol/L (>0.90 ng/mL);
8. HbA1c ≥7% and ≤10.5%;
9. Females must be using one of the following birth control methods: a non-hormonal intrauterine device (IUD) in place for at least 3 months; barrier method (condom, diaphragm) with spermicide for at least 14 days prior to dosing on Day 1 and throughout the study; surgical sterilization of the partner (vasectomy for 6 months minimum); OR are surgically sterile OR postmenopausal, as defined below:
   a. Surgically sterile for at least 3 months prior to screening by one of the following means:
      - Bilateral tubal ligation
      - Salpingectomy (with or without oophorectomy)
      - Surgical hysterectomy
      - Bilateral oophorectomy (with or without hysterectomy)
   b. Postmenopausal, defined as following:
      - Last menstrual period greater than 12 months prior to screening, confirmed by FSH and estradiol levels at screening;
10. Nonsmoker, defined as not having smoked or used any form of tobacco for more than 6 months prior to screening;
11. Willing and able to adhere to study restrictions and to be confined at the clinical research center.

## 6.2. Subject Exclusion Criteria

Subjects will be excluded from study entry if any of the following exclusion criteria are present at screening:

1. Fasting blood glucose at screening or Day -1 ≤110 or ≥270 mg/dL (one repeat screening fasting blood glucose allowed);
2. Type 1 diabetes mellitus, or latent autoimmune diabetes in adults; diabetic neuropathy, retinopathy or nephropathy;
3. Reported incidence of severe hypoglycemia, defined by denoting severe cognitive impairment requiring external assistance for recovery, within 3 months prior to screening and/or within 3 months prior to dosing on Day 1;
4. Known contraindications to sitagliptin;
5. Clinically significant gastrointestinal disorder (e.g., gastroparesis, constipation, diarrhea, colitis, gastrointestinal tract obstruction, hiatal hernia with reflux esophagitis, or cholestasis);
6. History or symptoms of clinically significant cardiovascular disease, particularly coronary artery disease, arrhythmias, atrial tachycardia, or congestive heart disease within one year prior to screening;
7. History of more than three urinary tract infections and/or more than three genital fungal infections in the last 12 months. If one or more genital fungal infections in the last 12 months required systemic treatment to resolve, Investigator will determine eligibility based on treatment required;
8. Reported history of clinically significant central nervous system disease including: transient ischemic attack, stroke, seizure disorder, depression, or behavioral disturbances within one year prior to screening;
9. Reported history of liver disease (e.g., hepatitis, cirrhosis within one year of screening and/or liver enzymes (alanine aminotransferase (ALT), aspartate aminotransferase (AST), gamma-glutamyltransferase (GGT), total bilirubin (TBiL)) >2 times the upper limit of normal (ULN));
10. Reported history of clinically significant renal disease;
11. Estimated glomerular filtration rate (eGFR) ≤60 mL/min/1.73m$^2$;
12. Acute or chronic metabolic acidosis, including diabetic ketoacidosis;

13. Known or suspected malignancy;
14. Any reported hypersensitivity (anaphylaxis and angioedema) or intolerance to sitagliptin;
15. Antidiabetic treatment with insulin, sulfonylureas, thiazolidinediones or GLP-1 agonist within 3 months prior to screening;
16. Systolic blood pressure <90 or ≥160 mmHg or diastolic blood pressure <60 or ≥100 mmHg at screening (may be repeated once);
17. A hospital admission or major surgery within 90 days prior to screening;
18. Uncontrolled hypertriglyceridemia >500 mg/dL;
19. Positive blood screen for human immunodeficiency virus (HIV), hepatitis B surface antigen (HBsAg), or hepatitis C antibody;
20. Positive pregnancy test result;
21. Female is breast-feeding or planning to become pregnant;
22. Treated with any investigational drugs within 6 weeks prior to screening;
23. Reported history of prescription drug abuse, or illicit drug use within 6 months prior to screening;
24. Reported history of alcohol abuse according to medical history within 6 months prior to screening;
25. A positive screen for alcohol or drugs of abuse;
26. Reported history of donation or blood collection of more than 1 unit (approximate 450 mL) of blood (or blood products) or acute loss of blood during the 90 days prior to screening;
27. Use of prescription or over-the-counter (OTC) medications, and herbal (including St John's Wort, herbal teas, garlic extracts) within 14 days prior to dosing (except for sitagliptin dosing during run-in period). No angiotensin-converting enzyme (ACE) inhibitors are allowed; (Note: Use of acetaminophen at <1 g/day is permitted until 24 hours prior to dosing);
28. An unwillingness of male participants to use appropriate contraceptive measures if engaging in sexual intercourse with a female partner of childbearing potential. Appropriate measures include use of a condom and spermicide and, for female partners, use of an IUD, diaphragm with spermicide, oral contraceptives, injectable progesterone, progesterone subdermal implants, or a tubal ligation. Sexual intercourse with pregnant or lactating women is prohibited.

## 6.3. Prohibitions and Restrictions

Subjects must be willing to adhere to the following prohibitions and restrictions from times noted and for the duration of the study:

- No illicit drug use, abuse of alcohol and use of tobacco-containing products within 6 months prior to screening;
- No consumption of alcohol or food containing alcohol;
- No consumption of food or drinks containing caffeine;
- No consumption of grapefruit juices;
- No administration of any prescription medications (with the exception of study drug) per Exclusion Criteria #27;
- No use of any OTC products (with the exception of acetaminophen <1 g/day until 24 hours prior to dosing) per Exclusion Criteria #27;
- Subjects must remain upright for 4 hours following administration of study drug (except during measurement of vital signs and ECGs) following the morning dose on Days 5, 10 and 15.

## 6.4. Subject Withdraw Criteria

Subjects will be informed that they have the right to withdraw from the study at any time for any reason without prejudice to their medical care. The Investigator also has the right to withdraw subjects from the study if it is in the best interest of the subject, or if the subject experiences an adverse event that warrants premature withdrawal.

Subjects may be withdrawn from the study if they:

- Have entered the study in violation of the protocol
- Use or require the use of prohibited concomitant medication(s)
- Are non-compliant with study procedures
- Experience an AE that warrants premature withdrawal

Subjects who experience emesis after dosing are not required to withdraw from the study. The event and time of emesis should be documented, and the subject permitted to continue at the discretion of the Investigator.

All treated subjects should be followed according to the Schedule of Procedures (Table 5-1). All subjects who receive a Day 1 dose, even those who have discontinued prematurely, should have all evaluations for the Day 17 End-of-Study visit performed, if possible. All procedures should be documented in the electronic case report form (eCRF). For all subjects who withdraw prematurely, the Investigator will indicate one of the following reasons for withdrawal in the eCRF:

- Adverse event
- Death
- Protocol violation
- Lost to follow-up
- Subject's decision
- Other (reason to be specified by the Investigator)

In case of subjects' discontinuation from the study due to an AE, such subjects will be closely monitored until the resolution or stabilization of the AE. The Investigator should document the reason for discontinuation in the source documentation and eCRF.

In the event that a subject withdraws participation from the study early, early withdrawal should be documented by the Investigator (or designee) in the appropriate eCRF pages and source documents when confirmed. The Day 17 End-of-Study assessments should be performed when a subject is discontinued.

# 7. STUDY PROCEDURES

Morning dosing will occur at approximately 08:00 a.m. and the evening dosing will occur at approximately 06:00 p.m. All doses will be administered 60 (±5) minutes prior to meals (except for on Days 5, 10 and 15 when meals are not offered after the morning dose) with about 240 mL (8 fluid ounces) of room temperature water. Actual times for each dose and start times for meals as well as oral glucose intake on days of dosing will be recorded. Standardized meals that are consistent with general dietary recommendations for diabetes will be provided while subjects are staying at CRC.

Eligible subjects will have a minimum 12-day run-in period prior to admission to the CRC on Day -2, during which time they will self-administer sitagliptin 100 mg QD each morning. Subjects will complete a diary to record sitagliptin doses taken each day and will be advised to monitor their blood glucose levels and report changes in health status to the CRC. A telephone call will be placed to all subjects at approximately Day 6 (±2 day) of the run-in period to assess general health and collect AE information. Subjects will be admitted to the CRC on Day -2 and remain sequestered at the study site until after all End-of-Study procedures are completed on Day 17 or at early termination.

The following sections describe in detail all study procedures. The schedule of procedures is presented in Table 5-1.

## 7.1. Screening Visit and Run-in Period

Subjects will report to the CRC for a screening outpatient visit between Day -42 and Day -15 relative to the Day 1 dosing day.

As outlined in Section 15.3, prior to the performance of any study-related activities or evaluations, the subject must be thoroughly informed about all aspects of the study, including scheduled visits and activities. Each subject will sign the study-specific consent form prior to any screening procedures. A signed copy of the informed consent form (ICF) will be given to each consenting subject and another signed copy will be retained in the subject's study records.

The following information and procedures will be performed and documented as part of the screening assessment:

- Collection of demographic information, including sex, race, ethnic origin, date of birth.
- Medical history, including review of prior and ongoing medications taken in previous 30 days, except for therapy to treat diabetes, which must be recorded for previous 3 months.
- Height and weight measurement and BMI calculation.
- Urine sample collection for urinalysis and test for drugs of abuse.
- Urine sample pregnancy test for women.
- Blood test for evaluation of FSH and estradiol for postmenopausal women.
- Saliva sample collection to test use of alcohol.
- Standard 12-lead ECG after resting supine for at least 5 minutes.

- Vital signs (BP, PR, RR and oral T) measurement after resting supine for at least 5 minutes.
- Blood sample collection for clinical chemistry, hematology, HIV, and hepatitis B and C evaluations, C-peptide measurement and HbA1c test.
- Physical examination.
- Assessment of eligibility according to inclusion/exclusion criteria.

Compliance with inclusion criteria (listed in Section 6.1) and exclusion criteria (listed in Section 6.2) will be verified against information collected and documented in the source documents and the eCRF. Laboratory results obtained at screening and on Day -1 will be used to verify eligibility.

Eligible subjects will be scheduled for a visit to the CRC for dispensing of sitagliptin 100 mg and start their 12-day run-in period. Subjects will be instructed to take a single dose of sitagliptin each morning, and to record each dose in a diary that will be provided by the CRC. Subjects will be advised to monitor their blood glucose levels and report changes in health status to the CRC. A telephone call will be placed to all subjects at approximately Day 6 (±2 day) of the run-in period to assess general health and collect AE information. Any signs or symptoms reported by subjects that develop or worsen compared to the subject's medical history after the first dose of sitagliptin in the run-in period will be recorded as AEs and will be followed by the Investigator. Additionally, subjects will be advised to contact their primary care physician.

An appointment will be scheduled for subjects to be admitted to the CRC on Day -2. Subjects will be advised to take their dose of sitagliptin at home prior to admission, and to bring remaining drug and their diary with them to the CRC on Day -2.

## 7.2. Domiciled at CRC on Day -2 through Day 17

### 7.2.1. Study Check-in Visit: Day -2 and Day -1

Subjects will be admitted to the CRC on Day -2 and remain for 18 overnight stays until completion of all study procedures on Day 17 or early termination.

The following assessments will be performed on Day -2:

- Collection of remaining sitagliptin and review of diary to confirm compliance with daily dosing of sitagliptin during the run-in period. Subjects with <90% compliance or compliance recorded as >110% are not eligible to continue study participation.
- Update of medical history, assessment of AEs, and medication use since the screening visit.
- Urine sample testing for drugs of abuse and pregnancy, as applicable.
- Alcohol saliva test.
- Assess blood glucose using a glucometer within 60 minutes before each meal and after study drug dosing when applicable.

The following assessments will be performed on Day -1:

- Blood and urine sample collection for clinical chemistry, hematology and urinalysis in the morning under fasting condition (after at least a 10-hour fast).
- Vital signs (BP, PR, RR and oral T) measurement after resting supine for at least 5 minutes.
- Measure weight
- Standard 12-lead ECG after resting supine for at least 5 minutes.
- Subjects will be administered sitagliptin 100 mg by the clinic staff in the morning at approximately 08:00 a.m. prior to a standardized meal.
- Assess blood glucose using a glucometer within 60 minutes before each meal and after study drug dosing when applicable.
- Confirm fasting blood glucose on Day -1 >110 and <270 mg/dL.
- Confirmation of eligibility according to inclusion/exclusion criteria.
- Assessment of AEs and concomitant medications.

### 7.2.2. Day 1 - Day 4

- Vital signs (BP, PR, RR and oral T) measurement after resting supine for at least 5 minutes within 60 minutes before each study drug dose.
- Administer 100 mg sitagliptin QD at 08:00 a.m. (±5 minutes), 60 (±5) minutes prior to a standardized meal.

- Assess blood glucose using a glucometer within 60 minutes before each meal and after study drug dosing when applicable.
- Assessment of AEs and concomitant medications.

### 7.2.3. Day 5

- Blood and urine sample collection for clinical chemistry, hematology and urinalysis in the morning under fasting condition (after at least a 10-hour fast) and before study drug dose.
- Vital signs (BP, PR, RR and oral T) measurement after resting supine for at least 5 minutes within 60 minutes before study drug dose.
- Administer 100 mg sitagliptin at 08:00 a.m. (±5 minutes). Subjects must remain upright for at least 4 hours following dosing except for measurement of vital signs or ECGs.
- Pharmacokinetic blood sample collections for the measurement of sitagliptin concentrations [redacted].
- Consume a 75-gram glucose solution 30 (±5) minutes following study drug dose in lieu of a breakfast.
- Pharmacodynamic blood sample collections to measure serum levels of glucose, GLP-1, glucagon, insulin, and C-peptide at baseline (prior to study drug dose and within 60 minutes prior to oral glucose intake) and 0.5, 1, 1.5, 2, 2.5, 3 and 4 hours after oral glucose intake.
- Assess blood glucose using a glucometer within 60 minutes before each meal and after study drug dosing when applicable.
- Standard 12-lead ECG after resting supine for at least 5 minutes, taken 2 hours (±15 minutes) after study drug dose.
- Assessment of AEs and concomitant medications.

### 7.2.4. Day 6 - Day 9

- Pharmacokinetic blood sample collections for the measurement of sitagliptin concentrations 24 hours post-dose (Day 6).

- Vital signs (BP, PR, RR and oral T) measurement after resting supine for at least 5 minutes within 60 minutes before each study drug dose.
- Administer 100 mg sitagliptin QD at 08:00 a.m. (±5 minutes) and 75 mg dorzagliatin BID (08:00 a.m., 06:00 p.m. (±5 minutes), each dose 60 (±5) minutes prior to a standardized meal.
- Assess blood glucose using a glucometer within 60 minutes before each meal and after study drug dosing when applicable.
- Assessment of AEs and concomitant medications.

#### 7.2.5. Day 10

- Blood and urine sample collection for clinical chemistry, hematology and urinalysis in the morning under fasting condition (after at least a 10-hour fast) and before study drug dose.
- Vital signs (BP, PR, RR and oral T) measurement after resting supine for at least 5 minutes within 60 minutes before each study drug dose.
- Administer 100 mg sitagliptin and 75 mg dorzagliatin at 08:00 a.m. (±5 minutes). Subjects must remain upright for at least 4 hours following dosing except for measurement of vital signs or ECGs.
- Pharmacokinetic blood sample collections for the measurement of sitagliptin and dorzagliatin concentrations [redacted].
- Consume a 75-gram glucose solution 30 (±5) minutes following study drug dose in lieu of a breakfast.
- Pharmacodynamic blood sample collections to measure serum levels of glucose, GLP-1, glucagon, insulin, and C-peptide at baseline (prior to study drug dose and within 60 minutes prior to oral glucose intake) and 0.5, 1, 1.5, 2, 2.5, 3 and 4 hours after oral glucose intake.
- Assess blood glucose using a glucometer within 60 minutes before each meal and after study drug dosing when applicable.
- Standard 12-lead ECG after resting supine for at least 5 minutes, taken 2 hours (±15 minutes) after study drug dose.

- Assessment of AEs and concomitant medications.

### 7.2.6. Day 11 - Day 14

- Pharmacokinetic blood sample collections for the measurement of sitagliptin and dorzagliatin concentrations 24 hours post-dose (Day 11).
- Vital signs (BP, PR, RR and oral T) measurement after resting supine for at least 5 minutes within 60 minutes before each study drug dose.
- Administer 75 mg dorzagliatin BID (08:00 a.m., 06:00 p.m. (±5 minutes) 60 (±5) minutes prior to a standardized meal.
- Assess blood glucose using a glucometer within 60 minutes before each meal and after study drug dosing when applicable.
- Assessment of AEs and concomitant medications.

### 7.2.7. Day 15

- Blood and urine sample collection for clinical chemistry, hematology and urinalysis in the morning under fasting condition (after at least a 10-hour fast) and before each study drug dose.
- Vital signs (BP, PR, RR and oral T) measurement after resting supine for at least 5 minutes within 60 minutes before each study drug dose.
- Administer 75 mg dorzagliatin at 08:00 a.m. (±5 minutes). Subjects must remain upright for at least 4 hours following dosing except for measurement of vital signs or ECGs.
- Pharmacokinetic blood sample collections for the measurement of dorzagliatin concentrations [redacted].
- Consume a 75-gram glucose solution 30 (±5) minutes following study drug dose in lieu of a breakfast.
- Pharmacodynamic blood sample collections to measure serum levels of glucose, GLP-1, glucagon, insulin, and C-peptide at baseline (prior to study drug dose and within 60 minutes prior to oral glucose intake) and 0.5, 1, 1.5, 2, 2.5, 3 and 4 hours after oral glucose intake.

- Assess blood glucose using a glucometer within 60 minutes before each meal and after study drug dosing when applicable.
- Standard 12-lead ECG after resting supine for at least 5 minutes, taken 2 hours (±15 minutes) after study drug dose.
- Assessment of AEs and concomitant medications.

#### 7.2.8. Day 16

- Pharmacokinetic blood sample collections for the measurement of dorzagliatin concentrations 24 hours post-dose.
- Assess blood glucose using a glucometer within 60 minutes before each meal.
- Subjects will resume their regular medication schedule.
- Assessment of AEs and concomitant medications.

#### 7.2.9. Day17: End-of Study Procedures

The following procedures will be performed at the End-of-Study or Termination Visit:

- Assess blood glucose using a glucometer within 60 minutes before morning meal.
- Blood and urine sample collection for clinical chemistry, hematology and urinalysis in the morning under fasting condition (after at least a 10-hour fast).
- Urine pregnancy test for women.
- Physical examination.
- Measure weight.
- Vital signs (BP, PR, RR and oral T) measurement after resting supine for at least 5 minutes.
- Standard 12-lead ECG after resting supine for at least 5 minutes.
- Assessment of AEs and concomitant medications.
- Subjects will be offered breakfast and may be discharged from the CRC upon completion of all study procedures.

### 7.3. Early Withdrawal Visit

As outlined in Section 6.4, in the event that a subject discontinues study participation after Day 1 dose administration but prior to the final End-of-Study visit, if possible, the subject should

complete all End-of Study assessments prior to being discharged from the CRC, as described in Section 7.2.9 for Day 17. The reason for discontinuation must be fully documented in the subject's source documentation and in the eCRF.

### 7.4. Time Windows for Procedures

Any procedure performed outside the stated time windows below will be recorded as a protocol deviation.

- Sitagliptin and dorzagliatin administered by clinic staff will be taken 60 (±5) minutes prior to meals.
- Standard resting 12-lead ECGs will be performed 2 hours (±15 minutes) after the study drug dose, after the subject has rested in the supine position for at least 5 minutes.
- The oral glucose solution will be administered 30 (±5) minutes after study drug dose on Days 5, 10 and 15.
- Blood samples for PK analyses will be collected ±5 minutes from 0-4 hours post-dose and ±10 minutes ≥6 hours post-dose.
- Blood samples for PD analyses will be collected ±5 minutes from 0-4 hours after glucose intake.
- Meal start times will be within ±10 minutes of scheduled start time.

## 8. TREATMENT OF SUBJECTS

### 8.1. Description of Study Drugs

Dorzagliatin (HMS5552) is an investigational novel GKA, indicated as an adjunct to diet and exercise to improve glycemic control in adults with T2DM.

The anticipated dosing regimen is twice daily as oral administration. The proposed dosage form for this study is a film-coated tablet with 75 mg dorzagliatin. [redacted]

[redacted]

[redacted]

Sitagliptin ([redacted]® 100 mg tablets for oral administration) will be supplied by Hua Medicine.

## 8.2. Methods of Assigning Subjects to Dose Groups

There is no randomization since this is an open label, single sequence study. Subjects may be enrolled singly or in groups.

## 8.3. Blinding

This is an open label study. There will be no blinding for this study.

## 8.4. Treatment Discontinuation/Subject Withdrawal

Subjects may withdraw from the study at any time at their own request, or they may be withdrawn at any time at the discretion of the Investigator or sponsor for safety, behavioral, or administrative reasons. If a subject discontinues study participation at any time prior to the final End-of-Study assessments, if possible, the subject should complete all End-of Study assessments. The reason for discontinuation must be fully documented in the subject’s source documentation and in the eCRF.

## 8.5. Prior and Concomitant Medications and Substances

All prescription medications and OTC products, including herbal products, taken within 30 days prior to screening and during the study period will be documented in the subject’s source documentation and the eCRF. Therapy to treat T2DM taken within 3 months prior to screening will also be documented in the subject’s source documentation and the eCRF. The documented use of prescription sitagliptin during the run-in period is required.

## 8.6. Meals

Standardized meals based on the recommended American Diabetes Association diet, will be offered to all subjects. An evening snack (no later than 09:00 p.m.) will be offered on the evening of admission. Subjects will be domiciled and dosed, and have meals provided by the CRC. Standard meals will be served at approximately 60 minutes after dosing and shall be completed within 30 minutes when meals are administered after a study drug dose. While domiciled at the CRC, meals will be offered at approximately 09:00 a.m., 12:30 p.m. and

07:00 p.m., and a light evening snack will be offered at approximately 9:00 p.m. or earlier. Meal start times will be within ±10 minutes when the meal time coincides with a scheduled PK or PD blood draw. Soft drinks (sodas) without caffeine or sugar, or non-grapefruit juices without added sugar will be offered with meals and ad libitum beginning 4 hours post-dose.

Water intake will be restricted from 1 hour prior to dosing until after the 1-hour post-dose PK blood sample is collected (with the exception of 240 mL of water given with the dosing) on study Days 5, 10 and 15.

# 9. STUDY DRUG MATERIALS AND MANAGEMENT

## 9.1. Study Drug Packaging and Labeling

Dorzagliatin drug products are supplied as 75 mg strength tablets. The drug product tablets are packaged in Alu-Pla Blister as in total 8 tablets per plate.

Product will be labeled according to applicable regulatory requirements. The label will include at least the following information:

- Name and address of manufacturer
- Protocol number or other identifier to reference the study
- Name of the sponsor, product name and batch number
- Place to record subject number and initials
- Storage conditions

The CRC pharmacist or designee will prepare the study drug and maintain the drug packaging and labeling log.

Sitagliptin ([redacted]® 100 mg tablets for oral administration) will be supplied by Hua Medicine.

## 9.2. Study Drug Storage and Accountability

All study drug supplies will be stored in a secure, locked area, under the responsibility of the Investigator or other authorized individual.

Dorzagliatin must be stored under controlled room temperature (10-30°C or 50-86°F) in tightly closed containers protected from light. Sitagliptin must be stored at 20-25°C (68-77°F) with

excursions permitted to 15-30°C (59-86°F). The temperature and humidity of the stored room should be monitored and recorded.

The Investigator or designee must maintain accurate records of the receipt of all study drug, including date received, lot number, expiration date if applicable, amount received, condition of the package and the disposition of all study drug.

Current dispensing records will also be maintained including the date and amount of study drug dispensed and the identity of the subject receiving the study drug.

## 9.3. Study Drug Preparation

Sitagliptin 100 mg tablets will be dispensed to subjects for at-home dosing during the 12-day run-in period. Study drug reconciliation will be performed at the end of the run-in period by tablet count.

Dorzagliatin 75 mg and sitagliptin 100 mg tablets will be dispensed to subjects while domiciled at the CRC according the study treatment scheme. Morning and evening doses of study drug will be administered by CRC staff with approximately 240 mL (8 fluid ounces) room temperature water at approximately 08:00 a.m. and 06:00 p.m., 60 (±5) minutes prior to a meal, except for Days 5, 10 and 15 when there will be no meal following the morning dose.

## 9.4. Study Drug Handling and Disposal

All unused study drug and supplies must be returned to sponsor or disposed according to sponsor's instruction after the study is completed and the drug accountability log is reconciled.

# 10. PHARMACOKINETIC/PHARMACODYNAMIC ASSESSMENTS

## 10.1. Pharmacokinetic Sample Collection and Storage

A total of 42 blood samples (approximately 6 mL each) will be collected from each subject into $K_2EDTA$ tubes for determination of dorzagliatin and sitagliptin concentration and PK assessments. Samples will be collected [redacted] on Days 5, 10 and 15 using an indwelling catheter or direct venipuncture.

When PK blood collection times coincide with other procedures, the blood sample should be collected first, using the following post-dose windows: ±5 minutes from 0-4 hours post-dose and ±10 minutes $\geq$6 hours post-dose.

Contents of tube will be mixed thoroughly with gentle inversion at least 8 times to mix the anti-coagulant and stored on ice for no more than 30 minutes before centrifugation at approximately 4 (±1)°C at ~2000 g for 10 minutes. The harvested plasma will be split into two approximately equal aliquots and stored in 2 mL or appropriate size cryovial tubes.

Plasma samples will be stored at -70 (±10)°C within approximately 60 minutes of harvesting pending shipment to the bioanalytical laboratory for analysis. Full instructions for the collection, storage, and shipment of these samples will be provided in a separate PK Laboratory Manual.

## 10.2. Pharmacokinetic Specimen Labeling

Labels will be affixed to the cryovials in a manner that will prevent the label from being detached after being wet or freezing. The tube labels will contain the subject number, treatment, nominal day, and nominal collection time, as appropriate.

The site will provide a sample inventory page, listing the information above for each sample. The sample inventory page will also include the treatment cohort for each series of tubes.

## 10.3. Pharmacokinetic Sample Shipping Instructions

All PK plasma samples will be kept frozen and shipped on dry ice by the same day or overnight courier to:

Frontage Laboratories, Inc.
Sample Coordinator
[redacted]

Plasma samples will be shipped in two separate shipments. The first shipment of samples (the primary aliquot of each sample) will be shipped after subjects have completed the study. The second aliquot of each sample will be shipped after notification from the laboratory of receipt of primary samples.

## 10.4. Bioanalytical Methodology

The PK plasma samples will be analyzed using validated, specific and sensitive methods of liquid chromatographic separation with tandem mass spectrometric (LC-MS/MS) detection for concentrations of dorzagliatin and sitagliptin by the designated bioanalytical lab.

## 10.5. Pharmacokinetic Parameters

Dorzagliatin and sitagliptin PK parameters ($C_{max}$, $T_{max}$ and $AUC_{0-24h}$) will be computed for each subject using non-compartmental methods by Phoenix WinNonlin version 8.1 (Certara, Princeton, NJ USA) based on the actual sampling times. Additional PK parameters ($T_{1/2}$, $K_{el}$, $V_z/F$, CL/F) may be calculated if needed. Descriptive statistics will be calculated for the PK parameters for Days 5, 10 and 15. A description of PK terms is provided below.

**Table 10-1: PK Terms**

| PK Term | Description |
|---|---|
| $C_{max}$ | Observed maximum plasma concentration |
| $T_{max}$ | Time at which $C_{max}$ was first observed |
| $AUC_{0-24h}$ | Area under the concentration-time curve from 0 to 24 hours |
| $T_{1/2}$ | Terminal elimination half-life |
| $K_{el}$ | Elimination rate constant |
| $V_z/F$ | Apparent volume of distribution during terminal phase after oral administration |
| CL/F | Apparent total plasma clearance of drug after oral administration |

## 10.6. Pharmacodynamic Assessments

Subjects will consume a 75-gram glucose solution 30 (±5) minutes following study drug administration on Days 5, 10 and 15, in lieu of a breakfast. The glucose solution will be consumed within a 5-minute timeframe. A total of 48 blood samples (24 samples of approximately 8 mL each for measurement of GLP-1 and 24 samples of approximately 6 mL each for measurement of glucose, glucagon, insulin, and C-peptide will be collected from each subject for PD assessments. Samples will be collected at baseline (prior to the study drug dose and within 60 minutes prior to oral glucose intake) and 0.5, 1, 1.5, 2, 2.5, 3 and 4 hours after oral glucose intake on Days 5, 10 and 15.

When PD blood collection times coincide with other procedures, the blood sample should be collected first within the ±5 minutes time window.

Instructions for the collection, storage, and shipment of PD samples will be provided in a separate PD Laboratory Manual.

# 11. ASSESSMENT OF SAFETY

Safety assessments will include monitoring of AEs, blood glucose via glucometer readings, vital signs, clinical laboratory findings, resting 12-lead ECGs and PE findings.

## 11.1. Safety Parameters

### 11.1.1. Demographic/Medical History

Demographic characteristics (age, sex, race and ethnicity) will be collected at the screening visit. Medical history will be reviewed and collected at the screening and on Day -2.

### 11.1.2. Vital Signs

Vital signs, including BP, PR, RR, and oral T, will be measured at screening, on Day -1, on Days 1-15 within 60 minutes prior to each morning and evening dose, and on Day 17 or at the end of the study. Vital signs will be measured after resting supine for at least 5 minutes.

Vital signs may be repeated for clinically significant abnormal vital signs at the discretion of the Investigator.

As a guideline, vital signs outside the ranges shown below may be considered abnormal, at the discretion of the Investigator:

SBP: 95-160 mmHg

DBP: 55-100 mmHg

PR: 45-100 bpm

RR: 10-20 bpm

T: 36.0-37.2°C

### 11.1.3. Physical Examination, Height, Weight, and Body Mass Index

Full PEs will be performed by qualified personnel at the screening visit, Day -1, Day 17 and the End-of-Study visit. At the screening visit, height (centimeters) and weight (kilograms) will be

measured and BMI will be calculated. Weight will also be measured on Day -1 and Day 17. All abnormal findings will be documented in the source documentation and in the eCRF.

### 11.1.4. Electrocardiogram

Standard resting 12-lead ECGs will be performed at the screening visit, Day -1, 2 hours (±15 minutes) post-dose on Days 5, 10 and 15, and on Day 17 at the End-of-Study visit. All ECGs will be performed after the subject has rested supine for at least 5 minutes.

Post-dose ECGs must be evaluated for safety by the Investigator or his/her designee. The final conduction intervals entered into the eCRF will be those generated by the ECG machine, unless deemed significantly inaccurate by the review performed by the Investigator or designee. In these cases, the over-read intervals will be documented in the eCRF. The Investigator will also record an overall assessment of the ECG.

Clinically significant abnormal ECGs may be repeated at the discretion of the Investigator.

As a guideline, ECG parameters outside the ranges shown below may be considered abnormal, at the discretion of the Investigator:

VR: 45-100 bpm

PR: 120-210 msec

QRS: ≤120 msec

QT: <500 msec

QTc: <450 msec

### 11.1.5. Clinical Laboratory Assessments

Hematology, blood chemistry, and urinalysis evaluations will be performed at the screening visit, on Day -1, Days 5, 10, and 15, and Day 17 (End-of-Study Visit). Blood and urine samples will be collected after an overnight fast of at least 10 hours, and within 30 minutes prior to the morning dose of study drug on Days 5, 10 and 15. The list of clinical laboratory assessments is included in Appendix A.

C-peptide and HbA1c testing will be performed at the screening visit to determine eligibility.

Blood glucose will be measured using a glucometer three times each day on Days -2 through Day 16, within 60 minutes before each meal and after study drug dosing when applicable.

The results of clinical laboratory tests conducted at the screening visit and on Day -1 must be assessed by the Investigator to determine each subject's eligibility for participation in the study. The Investigator should signify review of the laboratory reports by signing and dating the report. If subjects' values are out of range and the Investigator deems the out of range value is clinically significant, the Investigator must discuss and agree upon individual cases with sponsor's Medical Monitor prior to enrollment.

Any significant abnormalities should be fully investigated. Whenever possible, the etiology of the abnormal findings will be documented on the eCRF. Laboratory results with clinically significant abnormal values may be repeated for verification. Additional tests and other evaluations required to establish the significance or etiology of an abnormal result or to monitor the course of an AE should be obtained when clinically indicated. In particular, if a clinically significant abnormal result is observed that is not resolved by the final study visit, repeat tests will be performed if possible and resolution or stability of the abnormality will be recorded in the source documentation.

Any clinically significant laboratory abnormalities that are either serious (e.g., results in hospital admission) or unexpected will be promptly reported to the representative of sponsor. Any additional relevant laboratory results obtained by the Investigator during the course of this study will be reported to sponsor.

Virus serology (HIV, and hepatitis B and C) will be assessed at the screening visit and must be negative to qualify enrollment.

Urine drug screens and saliva alcohol tests will be conducted at the screening visit and on Day -2. Results must be negative to qualify for dosing on Day 1.

Urine pregnancy test for all female subjects will be performed at the screening visit, on Day -2 and on Day 17 (End-of-Study). Results at screening and Day -2 must be negative to qualify for dosing on Day 1. Any positive pregnancy test results on Day 17 will be reported to the sponsor within 24 hours of awareness of the pregnancy using a Pregnancy Report provided by Frontage.

Postmenopausal status of females will be confirmed by serum FSH and estradiol levels at screening.

## 11.2. Adverse Events

Adverse events will be recorded after the first dose of sitagliptin in the run-in period. Subjects will be instructed to report AEs during the study and staff will query subjects regarding AEs throughout the study. The Investigator or designee must document all AEs reported by the subject after the first dose of sitagliptin in the run-in period through completion of the End-of-Study visit. Any subject who is withdrawn from the study due to an AE shall be followed until the event has resolved or stabilized, and the Investigator will document available follow-up information on the subject's source documentation and eCRF.

### 11.2.1. Definitions of Adverse Events

Adverse event means any untoward medical occurrence in a patient or clinical investigation subject administered a pharmaceutical product and which does not necessarily have to have a causal relationship with this treatment. An AE can therefore be any unfavorable and unintended sign, symptom, or disease temporally associated with the use of medicinal product, whether or not related to the medicinal product. An AE is any symptom, sign, illness, or experience that develops or worsens in severity and/or frequency during the course of the study. Adverse events reported after administration of the first dose of study drugs (Day 1) will be considered treatment-emergent. Adverse events will be considered treatment-emergent if not present prior to the initiation of the treatment with study drug on Day 1 or already present but worsens in either severity or frequency following exposure to the treatment.

Adverse events include serious and non-serious AEs. A Serious Adverse Event (SAE) is defined as an AE occurring during any clinical study period that meets one or more following criteria:

- Results in death
- Is life threatening
- Requires inpatient hospitalization or prolongation of existing hospitalization
- Results in persistent or significant disability/incapacity
- Is a congenital anomaly/birth defect
- Is a medically important event that may jeopardize the subject or may require medical or surgical intervention to prevent one of the other outcomes listed above

The term "life threatening" in the definition of "serious" refers to an event in which the subject was at risk of death at the time of the event; it does not refer to an event that hypothetically might have caused death if it were more severe. Death is an outcome of an event. The event that resulted in death should be recorded in source documents, the eCRF and reported on the SAE form. All SAEs that occur after consent to the End-of-Study visit must be reported to sponsor using the SAE Reporting Form provided by Frontage (see Section 11.2.3).

Abnormal results of laboratory tests or diagnostic procedures (such as test results from hematology, blood biochemistry, urinalysis, ECG, physical examination, vital signs evaluations, etc.) are considered to be AEs if the abnormality is considered by the Investigator as clinically significant, or the clinical significance worsens compared to the subject's baseline.

Any of the following abnormal results are considered clinically significant:

- The abnormality suggests a disease and/or organ toxicity that is new or has worsened from baseline;
- The abnormality needs to adjust investigational product dosage and usage, e.g., drug discontinuation;
- The abnormality requires additional active intervention, for instance an increase or a modification of the concomitant medication, close observation, or further diagnostic investigation, etc.

If a clinically significant laboratory abnormality is a manifestation of disease, then only the diagnosis will be recorded as AE. If a clinically significant laboratory abnormality is not a manifestation of disease, then the abnormality itself is recorded as AE. An appropriate description is used to record test results that are either lower or higher than normal range. If the result meets diagnostic criteria, the clinical diagnosis is recorded as AE.

The severity of each AE will be graded by the Investigator according to the following criteria:

- Mild: awareness of sign or symptom, but easily tolerated.
- Moderate: discomfort sufficient to cause interference with normal activities.
- Severe: incapacitating, with inability to perform normal activities.

The Investigator must assess the relationship between the AE and the study drug by using the following definitions:

1. **Related**: The occurrence of AE follows a reasonable temporal sequence from administration of the study drug; adverse event is consistent with known characteristics of the study drug; the event is improved when the dose of study drug is decreased or stopped; the event re-occurs when the study drug treatment is re-started. It cannot be explained by the medical condition of the subject or alternative treatment.

2. **Possibly Related**: The occurrence of AE follows a reasonable temporal sequence from administration of the study drug; AE is consistent with a known characteristic of the study drug; reducing the dosage or stop the drug will cause the AE to be alleviated or no obvious change. The medical condition of the subject or alternative treatment may have contributed to the event.

3. **Unlikely Related**: The occurrence of AE, whose temporal sequence from administration of the study drug, is unclear. The AE may not be consistent with a known characteristic of the study drug. The medical condition of the subject or alternative treatments may have contributed to the event.

4. **Not Related**: There is no reasonable temporal relationship between the AE occurrence and study drug administration; the AE is not consistent with known characteristic of the study drug; the medical condition of the subject or alternative treatments may have contributed to the event. The AE improves or disappears when disease condition improves, or alternative treatment is stopped.

The outcome of an AE can be described as:

- Recovered: the subject returns to baseline state.
- Recovering: the events haven't been resolved completely, but subject is improving.
- Not recovered: the events are ongoing, for example, irreversible congenital anomaly
- Recovered with sequelae: only if the subject will suffer from life-time sequelae, for example, the blindness caused by diabetes and the hemiplegia after a stroke.
- Fatal: the death date is the event end date.
- Unknown: Investigator can't obtain the outcomes of AEs, e.g., the subject is lost to follow-up.

If the AE outcome is assessed as "Recovered", "Recovered with sequelae", or "Fatal", the end date of the AE must be recorded.

### 11.2.2. Recording Adverse Events

All AEs (regardless of seriousness or relationship to study drug) are to be recorded in the subject's source documents and on the corresponding page(s) in the eCRF. Whenever possible, symptoms, signs, and laboratory abnormalities should be grouped as a single syndrome or diagnosis. The Investigator should specify the date of onset and stop date of event, severity and the time of severity worsening, action taken with respect to study drug, corrective treatment/therapy given, seriousness, outcome, hospitalization date (if applicable), discharge date (if applicable), cause of the death (if applicable), date of death (if applicable), autopsy (if applicable) and his/her opinion as to whether there is a reasonable possibility that the AE was caused by the study drug, according to the definitions noted in Section 11.2.1. All medications administered to treat an AE must be recorded in the subject's source documentation and documented in the eCRF.

### 11.2.3. Reporting of Serious Adverse Events

Reporting of SAEs will be conducted in accordance with the appropriate regulatory guidelines. All SAEs that occur from the time of consent to the End-of-Study visit must be reported, whether or not the event is considered associated with the study drug. The Investigator must complete the SAE Reporting Form provided by Frontage and submit it by fax or email with other relevant source documentation to sponsor within 24 hours of awareness of the event to:

[redacted]

The Investigator must also provide with urgent priority (upon receipt of a request) other relevant documentation (e.g., copies of diagnostic test results, hospital discharge summary, and/or autopsy report) and send this information by fax or email to sponsor.

All SAEs must be recorded in the subject's source documentation and documented in the eCRF. Medications administered in association with the SAE must be documented in the eCRF and in the subject's source documentation. The Investigator must also promptly notify the Institutional Review Board (IRB) /Independent Ethics Committee (IEC) of SAEs, including any follow-up information, in accordance with local institutional policy and applicable regulatory requirements.

Regulatory authorities will be notified of any AE associated with the use of the study drug that is both serious and unexpected by sponsor, in accordance with the appropriate local regulatory guidelines. Notification of the event will be made by a written, expedited safety report.

### 11.2.4. Adverse Event Follow-up

The Investigator should take all appropriate measures to ensure the safety of the subjects, including referral to a specialist if indicated. The Investigator should follow up on the outcome of any AE until the event has resolved or stabilized.

This implies that follow-up may continue after the subject discontinues from the study and that additional information may be requested. Any SAE follow-up information that occurs after database lock should be reported to sponsor.

### 11.2.5. Hy's Law

The study will utilize Hy's Law to monitor any potential drug-induced liver injury. Hy's Law usually means that AST or ALT > 3 x ULN and TBiL > 2 x ULN, non-biliary increase (usually alkaline phosphatase < 2 x ULN) without any other diseases can explain this increase. If ALT or AST > 3 x ULN, and TBiL > 2 x ULN without any other past diseases can explain this increase, no matter ALP increases or not, re-evaluation need to be performed. Investigator will conduct subject re-evaluation, and closely follow-up of the subjects or stop the study drug due to liver function abnormality, according to Section 6.4 Subject withdrawal. The Investigator will evaluate the etiology and perform every essential examination to rule out drug-induced liver injury. If Investigator confirms the occurrence of Hy's Law, an SAE must be reported.

### 11.2.6. Hypoglycemia

Hypoglycemia events shall be recorded on an eCRF page. The following information will be recorded for each occurrence of hypoglycemia:

- Start/end date and time
- Blood glucose values
- Symptom descriptions
- Action taken
- Severe hypoglycemia or not
- Resolved or not

- Predisposing factors
- Causal relationship with the study drug

Hypoglycemia should be treated using standard medical practice at the Investigator's discretion. Severe hypoglycemia events will be reported as an SAE.

# 12. STATISTICAL METHODOLOGY

## 12.1. Sample Size Determination

Sample size calculations based on study design and intra-subject variability were performed by the sponsor. At least 10 evaluable subjects in the sequence will be required to achieve a power of at least 0.8 for the geometric mean ratios between two treatments (sitagliptin + dorzagliatin vs. dorzagliatin alone or sitagliptin + dorzagliatin vs. Sitagliptin alone) for $C_{max}$ or $AUC_{0\text{-}24h}$, with the equivalence bounds of 0.8 and 1.25, assuming a true geometric mean ratio of 1 and an intra-subject variability (coefficient of variation) of 16.1%, in an equivalence test using two one-sided test at a significance level of 0.05. The intra-subject variability for sitagliptin $C_{max}$ and AUC are reported to be 16.1% and 5.7%, respectively.[10] The intra-subject variability for dorzagliatin $C_{max}$ and $AUC_{0\text{-}24h}$ are estimated to be 14.0% and 6.2%, respectively.

Therefore, to ensure a satisfactory DDI assessment, and assuming a drop-out rate of 20%, we plan to enroll 15 eligible subjects by aiming to obtain 12 evaluable subjects for DDI assessment.

## 12.2. Analysis Population

The Safety Population will be defined as all subjects who receive study drug.

The PK Population will be defined as all subjects who receive study drug, have no major protocol violations, and have sufficient PK data to obtain reliable estimates of the key PK variables.

The PD Population will be defined as all subjects who receive study drug, have no major protocol violations, and have sufficient PD data to obtain reliable estimates of the key PD parameters.

The DDI Population will consist of the PK Population subjects who complete all treatments as defined by the protocol.

## 12.3. Pharmacokinetics and Pharmacodynamics

Pharmacokinetic parameters described in Section 10 will be summarized by descriptive statistics (n, mean, standard deviation, median, minimum, maximum) or frequency counts. Descriptive statistics will be calculated for dorzagliatin and sitagliptin concentrations in plasma.

Dorzagliatin and sitagliptin PK parameters ($C_{max}$, $T_{max}$ and $AUC_{0-24h}$) will be computed for each subject using non-compartmental methods and based on the actual sampling times. Descriptive statistics will be calculated for the PK parameters on Days 5, 10 and 15.

The effect of sitagliptin on the exposure of dorzagliatin will be determined by a PK comparison between the exposure of dorzagliatin on Day 10 vs. Day 15. The effect of dorzagliatin on the exposure of sitagliptin will be determined by a PK comparison between the exposure of sitagliptin on Day 10 vs. Day 5.

The primary PK comparison will be based on the $C_{max}$ and $AUC_{0-24h}$ of dorzagliatin and sitagliptin. A mixed model under the sequential design will be used for analyzing $C_{max}$ and $AUC_{0-24h}$ values, based on natural log-transformed values, with treatments as fixed effects and subjects within sequence as random effects. The adjusted geometric mean ratios between two treatments for these parameters and the corresponding 90% confidence intervals (CI) for the ratio will be obtained by exponentiating the adjusted mean difference in logarithms and will be used for assessing PK differences between the two treatments within each PK comparison.

A Statistical Analysis Plan (SAP) will be developed and signed prior to database lock, and will describe in more detail how PK parameters will be derived.

Pharmacodynamic measurements (glucose, GLP-1, glucagon, insulin, and C-peptide) will be listed by subject for all subjects with actual sampling time. Summary statistics of glucose, GLP-1, glucagon, insulin, and C-peptide will be provided by scheduled (nominal) time point and treatment, respectively. Baseline corrected PD markers will be calculated by subtracting the baseline value from the values post oral glucose intake. The baseline for PD correction will be defined in the SAP. Plots of mean PD markers versus the scheduled time will be generated for each treatment. Derivations and statistical analysis methods of PD parameter ($AUEC_{0-4h}$, $CE_{max}$ and $CE_{av}$) will be described in detail in the SAP.

## 12.4. Demographic Characteristics

Demographic characteristics will be summarized for the subjects enrolled in the study using descriptive statistics.

An attempt will be made to enroll similar numbers of men and women in the study.

## 12.5. Exposure to Study Drugs

Each subject's exposure to study drug will be summarized using descriptive statistics, i.e., the number of subjects exposed to each treatment.

## 12.6. Concomitant Medications

Concomitant medications will be coded using the World Health Organization (WHO) Enhanced dictionary.

## 12.7. Safety Analyses

Safety evaluations will be based on the incidence, severity, and relatedness of AEs and changes in subjects' PE findings, ECGs, vital signs, and clinical laboratory results.

Safety variables will be tabulated and presented for all subjects in the Safety Population. Data will be summarized by treatment. Adverse events will be coded using the Medical Dictionary for Regulatory Activities (MedDRA) for preferred term and system organ class, listed by subject. TEAEs will be summarized by treatment. All AEs will be summarized by relationship to study drug and by severity.

Deaths, SAEs, and AEs resulting in study discontinuation will be tabulated and detailed in narratives.

Change from baseline, defined as time of admission to the CRC or screening, whichever value is the last value prior to first dose, in clinical laboratory parameters, 12-lead ECGs, and vital sign parameters will be summarized by treatment.

Additional safety analyses may be defined in the SAP.

## 12.8. Interim Analyses

No interim analyses are planned for this study.

# 13. DIRECT ACCESS TO SOURCE DATA/DOCUMENTS

## 13.1. Study Monitoring

At regular intervals during the study, the site will be contacted through monitoring visits, letters, and telephone calls by a representative to review study progress, Investigator and subject compliance with study protocol requirements, and any emergent problems. During monitoring visits, the following points will be reviewed in accordance with all applicable regulatory requirements and Standard Operating Procedures (SOPs): original medical records and other source documents, the Investigator site file, screening logs, ICF, subject recruitment and follow-up, SAE documentation and reporting, documentation and reporting of endpoints, study drug allocation, subject compliance with the study drug regimen, study drug accountability, concomitant therapy use, and quality of data. In addition, other required regulatory documents will be reviewed, including but not limited to: IRB/IEC composition and correspondence, laboratory certification(s), delegation of authority, and Investigator and study personnel curricula vitae.

## 13.2. Sponsor's Responsibility

The sponsor or its designee is responsible for the following:

- Selecting qualified Investigators;
- Providing Investigators with the information they need to properly conduct an investigation;
- Ensuring proper monitoring of the investigation;
- Ensuring that the applicable regulatory authorities, and all participating Investigators are properly informed of significant new information regarding AEs or risks associated with the medication being studied.

As the sponsor, Hua Medicine has delegated some responsibilities to Frontage Clinical Services, a Contract Research Organization (CRO).

## 13.3. Audits and Inspections

The sponsor's Quality Assurance Unit (or representative) may conduct audits at the study site(s). Audits will include, but are not limited to: drug supply, presence of required documents, the

informed consent process, laboratory specimen processing, and comparison of eCRFs with source documents. The Investigator agrees to cooperate with audits conducted at a reasonable time and in a reasonable manner.

Regulatory authorities worldwide may also inspect the Investigator site during or after the study. The Investigator should contact the sponsor immediately if this occurs and must fully cooperate with the Inspector.

The Investigator is required to make all study documentation promptly available for inspection, review or audit at the study site upon request by sponsor, its representatives, or any appropriate regulatory agencies.

# 14. QUALITY CONTROL AND QUALITY ASSURANCE

Each investigational site will perform internal quality management of study conduct, data and biological specimen collection, documentation and completion. An individualized quality management plan may be developed to describe a site's quality management.

Quality control (QC) procedures will be implemented beginning with the data entry system and data QC checks that will be run on the database will be generated. Any missing data or data anomalies will be communicated to the site(s) for clarification/resolution.

Following written SOPs, the monitors will verify that the clinical trial is conducted, and data are generated, and biological specimens are collected, documented (recorded), and reported in compliance with the protocol, ICH GCP, and applicable regulatory requirements (e.g., GLP, Good Manufacturing Practices (GMP)).

The investigational sites will provide direct access to all trial related sites, source data/documents, and reports for the purpose of monitoring and auditing by the sponsor, and inspection by local and regulatory authorities.

# 15. ETHICS

The procedures set out in this study protocol, pertaining to the conduct, evaluation, and documentation of this study, are designed to ensure that the sponsor and the Investigator abide by GCP, including but not limited to Title 21 Code of Federal Regulations (CFR) Parts 50, 56, and 312 and the ICH guidelines and directives. Compliance with these regulations also constitutes

compliance with the ethical principles described in the current revision of the Declaration of Helsinki, Fortaleza 2013 and applicable local regulatory requirements and law.

Copies of these materials are available from Hua Medicine and Frontage Clinical Services (the CRO) designee by request. The purpose of these regulations, legal obligations and guidance is to define the standards and principles for the proper conduct of clinical studies that have been developed by the medical, scientific, and regulatory communities. They are not intended to impede or restrict clinical research.

The ethical standards defined within GCP are intended to ensure that:

1. Human subjects are provided with an adequate understanding of the possible risks of their participation in the study, and that they have a free choice to participate or not;
2. The study is conducted with diligence and in conformance with the protocol in such a way as to protect subject safety and ensure the integrity of the findings;
3. The potential benefits of the research justify the risks.

The Investigator is responsible for protecting the rights, safety, and welfare of subjects under his/her care, and for the control of the medications under investigation.

The Principal Investigator will ensure that all persons assisting with the study are adequately informed about the protocol, any amendments to the protocol, the study drug, and their study related duties and functions. The Investigator will maintain a list of sub-Investigators and other appropriately qualified persons to whom he or she has delegated significant study-related duties. Individuals ineligible to conduct or work on clinical studies, including those ineligible as a result of debarment under the Generic Drug Enforcement Act of 1992, will not be allowed to conduct or work on studies sponsored by Hua Medicine. The Investigator is required to immediately disclose to sponsor in writing, if any person involved in the conduct of the study is debarred pursuant to a hearing by US FDA under this anti-fraud law, or if any proceeding for debarment is pending, or is (to the best of the Investigator's knowledge) threatened.

The rest of this section of the protocol describes in more detail the specific GCP-defined responsibilities the Investigator assumes by agreeing to participate in this study.

## 15.1. Ethics Review

The Investigator (or designee) must submit this study protocol, the sponsor approved ICF, information intended for distribution to subjects, subject recruitment materials, and other appropriated documents to the appropriate IRB or IEC. Following IRB or IEC review, a copy of the signed, written and dated approval will be provided to the sponsor, along with a list of the IRB/IEC composition.

The approval/favorable opinion should clearly state the study (study number, protocol title, and version number), the documents reviewed (Protocol, ICF, IB, etc.) and the date of the review. The study will not commence at the study site until sponsor has received a copy of this written and dated approval/favorable opinion.

During the study, any amendment to the protocol and the ICF (as appropriate) shall be submitted to the IRB/IEC. The IRB/IEC should also be informed of any event likely to affect the safety of subjects or the continued conduct of the study, in particular any change in safety. Additionally, all updates to the IB will be sent to the IRB/IEC. A progress report will be sent to the IRB/IEC and the protocol will be reviewed annually (e.g., re-approved) or more frequently, as required by IRB/IEC or local regulations.

The Investigator will notify the IRB/IEC of the conclusion of the clinical study within 1 month of completion or termination of the study. The final report sent to the IRB/IEC will also be sent to sponsor, along with the completed eCRFs and all necessary regulatory documents, thereby fulfilling the Investigator's regulatory responsibility.

The Investigator will maintain a copy of all correspondence with the IRB/IEC, including copies of approved documents. The Investigator will also maintain a copy of the IRB/IEC membership list, including members' occupation and qualifications (or a statement confirming compliance with GCP requirements for committee composition). An IRB or IEC General Assurance Number may be accepted in lieu of a membership roster.

## 15.2. Ethical Conduct of the Study

This study will be conducted in accordance with GCP as delineated by Title 21 CFR Parts 50, 56, and 312, and the ICH guidelines and directives. Participating Investigators, including

members of the Committees and National Coordinators, will receive compensation for their time but will receive no financial profit from their activities related to the study.

Before the first subject is enrolled in the study, all ethical, regulatory, and legal requirements must be met.

An Investigator participating in this study is required to maintain complete and accurate study documentation in compliance with current GCP standards and all applicable local regulations related to the conduct of a clinical study.

## 15.3. Written Informed Consent

Prior to performing any study-related activities under this protocol, including screening tests and assessments, written informed consent with the approved ICF must be obtained from the subject or subject’s legally authorized representative, as applicable, in accordance with local practice and regulations. Written informed consent must be obtained from all subjects participating in a clinical study conducted by Hua Medicine.

The background of the proposed study, the procedures, the benefits and risks of the study, and that study participation is voluntary for the subject must be explained to the subject. The subject must be given sufficient time to consider whether to participate in the study.

A copy of the ICF, signed and dated by the subject, must be given to the subject. Confirmation of a subject’s informed consent must also be documented in the subject’s source documentation prior to any testing under this protocol, including screening tests and assessments.

Each consent form should contain an authorization allowing the Principal Investigator(s) and the sponsor to use and disclose patient health information (PHI) in compliance with local law.

The originally signed consent form will be retained with the study records.

# 16. DATA HANDLING AND RECORD KEEPING

## 16.1. Data Collection

All data obtained for analysis in the clinical study described in this protocol will be documented in the eCRF. Data reported in the eCRFs should be consistent with and substantiated by the subject’s medical record and original source documents.

Prior to the start of the study, the Principal Investigator will complete a Delegation of Authority form (Site Signature and Delegation Log), showing the signatures and handwritten initials of all individuals and the delegation of responsibilities, such as identifying those individuals who are authorized to make or change entries on eCRFs.

## 16.2. Case Report Form Completion

All eCRF entries, corrections, and alterations must be made by the investigator or authorized study-site personnel. Data within the eCRF will be monitored by the Clinical Research Associate according to the Monitoring Plan. Queries will be generated based on discrepancies found while monitoring. Site personnel will review and respond to these queries appropriately. Additionally, the sponsor may periodically perform aggregate data reviews, which could result in queries being generated for site personnel resolution. The final, completed eCRF for each subject must be signed and dated by the Principal Investigator to signify that the Principal Investigator has reviewed the eCRF and certifies it to be complete and accurate.

## 16.3. Database Management, Data Clarification, and Quality Assurance

A designated CRO will be responsible for data management. The Data Manager will develop a Data Management Plan (DMP) document and provide it to sponsor for approval. The DMP document will define all activities in the data collection and cleaning process. The detailed DMP will be based on the protocol, work scope, contract, analysis plans, data-flows, eCRFs, data cleaning procedures, other supporting documents, and data management standards and practices.

The programmed data validations will be run, and manual review will be conducted to check for database completeness and consistency, and queries will be generated upon data entry or via review by a Clinical Data Manager after entry. The sites will respond to the data queries in a timely manner.

Prior and concomitant medications will be coded using the World Health Organization (WHO) Drug Enhanced Dictionary (latest version, to be noted in DMP). All medical history and AEs will be coded using the Medical Dictionary for Regulatory Activities (MedDRA), using latest version, to be noted in DMP.

Quality control procedures will be conducted prior to database lock according to the designated CRO SOPs.

When the database has been declared to be complete and accurate, it will be locked. Any changes to the database after that time will only be made by joint written agreement between Hua Medicine, the study Statistician, the Data Manager, and the Quality Assurance Auditor according to designated CRO SOPs.

## 16.4. Inspection of Records/Source Documents

According to the ICH guidelines for GCP, the sponsor or designee must verify data entered in the eCRF entries against the source documents, except for the pre-identified source data directly documented in the eCRF (if applicable). The ICF will include a statement by which the subject allows the sponsor's duly authorized personnel, the IRB/IEC, and the regulatory authorities to have direct access to source data that supports the data in the eCRF (e.g., subject's medical file, appointment books, and original laboratory records). These personnel, bound by professional secrecy, must keep confidential all personal medical information.

The objective of source document verification (SDV) is to comply with GCP and international regulatory requirements and to reduce the risks of fraud. Source document verification means ensuring that the source documents are an accurate and verifiable reflection of the subject's participation in the study and that all relevant information that is recorded in the source document is accurately entered into the eCRF.

Where source documents serve as the basis for deriving data for the study, SDV should ensure that these documents are correctly labeled and filed, and that the data derived from them are correct. All source documents pertaining to this study will be maintained by the Investigator and made available for inspection by authorized persons. If electronic progress notes and other electronic source documents are not Title 21 CFR Part 11 compliant, they are not considered a valid source for this study. All progress notes must be dated and signed by the Principal Investigator or sub-Investigator at the time of the visit. The sponsor reserves the right to terminate the study for refusal of the Investigator to supply original source documentation for this clinical study.

The Investigator will note in a source independent from the eCRF the following information:

1. Information to confirm that the subject exists (e.g., initials, date of birth, sex)
2. Confirmation that the subject satisfies the inclusion/exclusion criteria

3. Confirmation that the subject is taking part in the clinical study
4. Confirmation of the informed consent process
5. Visit dates and documentation of protocol assessments and procedures
6. Information concerning all AEs
7. Details of concomitant and investigational medications

Source document verification is not a substitute for clinical study monitoring, the purpose of which is to ensure that the protocol has been followed correctly, the eCRF has been fully and accurately completed, SDV has been carried out, and the study timelines and enrollment goals and requirements have been met.

## 16.5. Retention of Records

The Investigator/institution must maintain all study documentation as confidential and take measures to prevent accidental or premature destruction of these documents.

Essential documents should be retained until at least 2 years after the last approval of a marketing application in an ICH region and until there are no pending or contemplated marketing applications in an ICH region or at least 2 years have elapsed since the formal discontinuation of clinical development of the investigational product (e.g., the Investigational New Drug application is withdrawn). These documents should be retained for a longer period, however, if required by the applicable regulatory requirements or by an agreement with sponsor.

The Investigator/institution must notify sponsor prior to destroying any study essential documents.

If the Investigator/institution can no longer ensure archiving, he/she shall inform the sponsor. The relevant records shall be transferred to a mutually agreed upon designee.

# 17. CONFIDENTIALITY

All information disclosed or provided by sponsor (or designee) or produced during the study including, but not limited to, the protocol, the eCRFs, the IB, and the results obtained during the course of the study (if applicable), are confidential. The Investigator or any person under his/her authority agrees to undertake to keep confidential and not to disclose the information to any third party without the prior written approval of sponsor.

However, submission of this protocol and any other necessary documentation to the IRB/IEC is expressly permitted, the IRB/IEC members having the same obligation of confidentiality.

All information generated in this study must be considered highly confidential and must not be disclosed to any persons not directly concerned with the study without prior written permission from sponsor. However, authorized regulatory officials and sponsor personnel (or designee) will be allowed full access to inspect and copy the records. The copied and inspected records will remain at the site and will not be transmitted or removed from the site.

All study drugs, subject bodily fluids, and/or other materials collected shall be used solely in accordance with this protocol, unless otherwise agreed to in writing by sponsor and responsible ethics committee(s) or regulatory authorities.

Subjects will be identified only by unique subject numbers in eCRFs. Their full names may, however, be made known to a regulatory agency or other authorized officials in the event of inspections. Documents containing the full name or other personally identifiable information of the subject are to remain at the site. This information will not be transferred to sponsor nor be contained in regulatory filings. In the event of inspections by authorized agencies, this subject identification may be disclosed.

## 17.1. Subject Data Protection

Prior to any testing under this protocol, including screening tests and assessments, candidates must also provide all authorizations required by local law (e.g., PHI authorization in North America).

The subject will not be identified by name in the eCRF or in any study reports and these reports will be used for research purposes only. The sponsor, its partner(s) and designee(s), and various government health agencies may inspect the records of this study. Every effort will be made to keep the subject’s personal medical data confidential.

The sponsor will protect individual subject information to the fullest extent possible during this study. At no time will a subject become identified in any publication or presentation.

However, the subject may have to become identified in the event of a regulatory authority auditor inspection in order to verify the accuracy of the data. Access to subject information is at

the discretion of sponsor and cannot occur prior to database lock or other specified events as determined solely by the discretion of sponsor.

# 18. STUDY PROTOCOL AMENDMENTS

The Investigator will not make any changes to this protocol without prior written consent from sponsor and subsequent approval by the IRB/IEC. Any permanent change to the protocol, whether it is an overall change or a change for specific study center(s), must be handled as a protocol amendment. Any amendment to the protocol that appears necessary as the study progresses will be fully discussed by the Investigator(s) and sponsor. If agreement is reached regarding the need for an amendment, it will be written by sponsor. The written amendment must be submitted to the chairman of the IRB/IEC identified with this responsibility. Except for administrative amendments, Investigators must await IRB/IEC approval of protocol amendments before implementing the change(s).

Administrative amendments are defined as having no effect on the safety of the research subjects, scope of the investigation, or quality of the study. However, a protocol change intended to eliminate an apparent immediate hazard to subjects should be implemented immediately, and the IRB/IEC notified within 5 days. The sponsor will ensure submission of any protocol amendments to the appropriate regulatory agencies.

When, in the judgment of the chairman of the local IRB/IEC, the Investigators, and/or sponsor, the amendment to the protocol substantially alters the study design and/or increases the potential risk to the subject; the currently approved written ICF will require similar modification. In such cases, the Investigator will obtain repeat informed consent from subjects enrolled in the study before expecting continued participation.

# 19. PUBLICATION

All information concerning the product as well as any information such as clinical indications for the study drugs, their formula, their formulation, methods of manufacture and other scientific data relating to it, that have been provided by sponsor or designee, and are unpublished, are confidential and must remain the sole property of Hua Medicine.

The Investigator will agree to use the information only for the purposes of carrying out this study and for no other purpose unless prior written permission from sponsor is obtained. The sponsor has full ownership of the eCRFs completed as part of the study.

By signing the study protocol, the Investigator agrees that the results of the study may be used for the purposes of national and international registration, publication, and information for medical and pharmaceutical professionals by sponsor. If necessary, the authorities will be notified of the Investigator's name, address, qualifications, and extent of involvement.

The sponsor or designee will prepare a final report on the study. The Investigator may not publish or present any information on this study without first presenting the information to sponsor for review.

# 20. REFERENCES

1. Hua Medicine (Shanghai), Ltd. China: Investigator's Brochure for Dorzagliatin (HMS5552), Second edition, December 29, 2017.
2. Zhu D, Gan S, Liu Y, et. al. Dorzagliatin monotherapy in Chinese patients with type 2 diabetes: dose-ranging, randomised, double-blind, placebo-controlled, phase 2 study. Lancet Diabetes Endocrinol, 2018; 6 (8): 627-636.
3. [redacted] Package Insert, 2018, [redacted] USA
4. [redacted] Package Insert, 2018, [redacted] USA.
5. [redacted] Package Insert, 2017, [redacted] USA.
6. [redacted] [redacted] [redacted] Package Insert, 2017, [redacted] USA.
7. [redacted] Package Insert, 2017, [redacted] USA.
8. [redacted] Package Insert, 2017, [redacted] USA.
9. [redacted] Package Insert, 2017, [redacted] USA.
10. Chung I, Oh J, Lee SH, et. al. A post hoc analysis of intra-subject coefficients of variation in pharmacokinetic measures to calculate optimal sample sizes for bioequivalence studies. Transl Clin Pharmacol, 2017; 25 (4): 179-182.

# APPENDIX A. LABORATORY ASSESSMENTS

| Hematology | Clinical Chemistry | Urinalysis |
|---|---|---|
| Hemoglobin (Hgb) | Blood urea nitrogen (BUN) | pH |
| Hematocrit (Hct) | Creatinine | Specific gravity |
| Platelet cout | Total bilirubin | Protein |
| Red blood cell (RBC) count | Alkaline phosphatase | Glucose |
| White blood cell (WBC) count | Aspartate transaminase (AST) | Ketones |
| | Alanine transaminase (ALT) | Bilirubin |
| with differential | Gamma-glutamyl | Blood |
| | transferase (GGT) | Nitrites |
| | Lactic dehydrogenase (LDH) | Leukocytes |
| | Glucose | Urobilinogen |
| | Albumin | Microscopic urine analysis |
| | Total protein | |
| | Bicarbonate | |
| | Phosphate | |
| | Sodium | |
| | Potassium | |
| | Chloride | |
| | Calcium | |
| | Total cholesterol | |
| | Triglyceride | |
| | HDL-C | |
| | LDL-C | |
| | Urate | |

| **Urine/Saliva Drug Screen** | **Serology Screen** |
| --- | --- |
| Amphetamines | Human immunodeficiency virus (HIV) |
| Barbiturates | Hepatitis B surface antigen (HBsAg) |
| Cannabinoids | Hepatitis C virus (HCV) |
| Cocaine metabolites | **Other** |
| Opiates | Urine Pregnancy test (all women, screening, Day -1, End-of-Study) |
| Benzodiazepines | Serum FSH and estradiol (postmenopausal women, screening) |
| Ethyl alcohol | HbA1c test (screening only) |
| | C-peptide test (screening only) |
| | **PD assessments** |
| | Glucose |
| | GLP-1 |
| | Glucagon |
| | Insulin |
| | C-peptide |
