# Supplementary material for: A phase I open-label clinical trial to study drug-drug interactions of Dorzagliatin and Sitagliptin in patients with type 2 diabetes and obesity
Source: Nat Commun. 2023 Mar 14;14:1405. doi: 10.1038/s41467-023-36946-7 (PMC10014962; doi:10.1038/s41467-023-36946-7)
Supplement: Supplementary file 3 — Reporting Summary [file 41467_2023_36946_MOESM3_ESM.pdf]

nature portfolio

Corresponding author(s): Li Chen

Last updated by author(s): Dec 27, 2022

Reporting Summary

Nature Portfolio wishes to improve the reproducibility of the work that we publish. This form provides structure for consistency and transparency in reporting. For further information on Nature Portfolio policies, see our Editorial Policies and the Editorial Policy Checklist.

Statistics

For all statistical analyses, confirm that the following items are present in the figure legend, table legend, main text, or Methods section.

n/a	Confirmed	
☐	☒	The exact sample size (n) for each experimental group/condition, given as a discrete number and unit of measurement
☐	☒	A statement on whether measurements were taken from distinct samples or whether the same sample was measured repeatedly
☐	☒	The statistical test(s) used AND whether they are one- or two-sided<br>*Only common tests should be described solely by name; describe more complex techniques in the Methods section.*
☐	☒	A description of all covariates tested
☐	☒	A description of any assumptions or corrections, such as tests of normality and adjustment for multiple comparisons
☐	☒	A full description of the statistical parameters including central tendency (e.g. means) or other basic estimates (e.g. regression coefficient) AND variation (e.g. standard deviation) or associated estimates of uncertainty (e.g. confidence intervals)
☐	☒	For null hypothesis testing, the test statistic (e.g. F, t, r) with confidence intervals, effect sizes, degrees of freedom and P value noted<br>*Give P values as exact values whenever suitable.*
☒	☐	For Bayesian analysis, information on the choice of priors and Markov chain Monte Carlo settings
☒	☐	For hierarchical and complex designs, identification of the appropriate level for tests and full reporting of outcomes
☒	☐	Estimates of effect sizes (e.g. Cohen's d, Pearson's r), indicating how they were calculated

Our web collection on statistics for biologists contains articles on many of the points above.

Software and code

Policy information about availability of computer code

Data collection	The Electronic Data Capture (Medrio Version R39.2) software (Medidata RAVE, Classic Rave version 2018.2.4) was used for clinical trial patient data collection through the study.
Data analysis	Commercially available pharmacokinetic (PK) data analysis software (WinNonlin Version 8.1) was used to analyze PK concentrations; and statistical analysis software (SAS Version 9.4 and R 3.6.6) was used to analyze clinical data from this study.

For manuscripts utilizing custom algorithms or software that are central to the research but not yet described in published literature, software must be made available to editors and reviewers. We strongly encourage code deposition in a community repository (e.g. GitHub). See the Nature Portfolio guidelines for submitting code & software for further information.

Data

Policy information about availability of data

All manuscripts must include a data availability statement. This statement should provide the following information, where applicable:

- Accession codes, unique identifiers, or web links for publicly available datasets
- A description of any restrictions on data availability
- For clinical datasets or third party data, please ensure that the statement adheres to our policy

The data from this study are available under restricted access for sponsor's contractual obligations, access can be obtained by application to the corresponding author (lichen@huamedicine.com) for non-commercial use. Applications should specifically outline the data the parties are interested in receiving and how the data will be used; the use of the data must also comply with country- or region-specific regulations. A signed data access agreement with the sponsor is required before

accessing the shared data. The study protocol is provided with the paper.

Human research participants

Policy information about studies involving human research participants and Sex and Gender in Research.

Reporting on sex and gender	This is a Phase 1, open-label, single-sequence study which included both male and female adults diagnosed as T2D. All eligible patients received 3 treatments (sitagliptin, sitagliptin+dorzagliatin, dorzagliatin) in the same sequence in hospitalization. There is no different MOA of T2D between genders, no different administration of sitagliptin labelled between genders, furthermore, metabolic profiles of dorzagliatin is not distinct between genders, thus gender-based analysis was not considered in this study.
Population characteristics	15 T2D subjects were enrolled in this study with T2DM male (n=4, 26.7%) and female (n=11, 73.3%) population ranging from 40 to 64 years of age. Overall mean (SD) age was 56.7 (5.39) years and mean (SD) BMI was 32.06 (3.571) kg/m2. Three of 15 subjects (20.0%) were black or African American, and 12 subjects (80.0%) were White. Fourteen of 15 subjects (93.3%) were Hispanic or Latino and one subject (6.7%) was not Hispanic or Latino. Mean (SD) of HbA1c was 8.24 (0.99)% and fasting blood glucose was 178.9 (44.79) mg/dL at baseline. The demographic and baseline characteristic were described in the manuscript (Table 1).
Recruitment	Subjects were recruited from 21 December 2018 to 30 August 2019. All subjects provided written informed consent prior to participating in the study and were compensated for the completed trial procedures. A total of 15 subjects were confirmed eligible at clinical pharmacology unit by the investigator.<br>The key inclusion criteria included: male and female adults eligible for inclusion had to meet the following criteria: patients aged between 30 and 65 years, in genera l good health who had been diagnosed as T2D for at least 3 months, with HbA1c between 7.0% and 10.5%, body mass index (BMI) between 19.0 and 38.0 kg/m2, taking a stable dose of metformin ≥1000 mg per day, or a DPP-4 inhibitor, or a sodium-glucose cotransporter-2 (SGLT-2) inhibitor, or metformin plus a DPP-4 inhibitor with no change in the dose for at least 4 weeks prior to screening, and accepting to change their current therapy to 100 mg sitagliptin QD for at least 14 days prior to dosing on Day 1.<br>The key exclusion criteria included: fasting blood glucose (FBG) ≤110 or ≥270 mg/dL, reported incidence of severe or serious hypoglycemia within 3 months prior to screening, type 1 diabetes or latent autoimmune diabetes, known hypersensitivity/ contraindication to study drugs, evidence of any clinically significant medical illness or functional disorders, and pregnant or breast-feeding women.<br>This is a single-sequence study, all eligible patients received the same run-in period and 3 sequential treatments. The patients fit the definition of analysis set were all included to perform analysis, thus biases seem unlikely.
Ethics oversight	The protocol was approved by an Institutional Review Board (IntegReview Ethics Review Board, Austin, USA) at the study site, and conducted in accordance with Declaration of Helsinki and International Conference on Harmonization Good Clinical Practice (ICH-GCP) guidelines, as well as US Food and Drug Administration regulations.

Note that full information on the approval of the study protocol must also be provided in the manuscript.

Field-specific reporting

Please select the one below that is the best fit for your research. If you are not sure, read the appropriate sections before making your selection.

☒ Life sciences ☐ Behavioural & social sciences ☐ Ecological, evolutionary & environmental sciences

For a reference copy of the document with all sections, see nature.com/documents/nr-reporting-summary-flat.pdf

Life sciences study design

All studies must disclose on these points even when the disclosure is negative.

Sample size	Sample size calculations based on study design and intra-subject variability were performed by the sponsor. At least 10 evaluable subjects in the sequence will be required to achieve a power of at least 0.8 for the geometric mean ratios between two treatments (sitagliptin + dorzagliatin vs. dorzagliatin alone or sitagliptin + dorzagliatin vs. Sitagliptin alone) for Cmax or AUC0-24h, with the equivalence bounds of 0.8 and 1.25, assuming a true geometric mean ratio of 1 and an intrasubject variability (coefficient of variation) of 16.1%, in an equivalence test using two one-sided test at a significance level of 0.05. The intra-subject variability for sitagliptin Cmax and AUC are reported to be 16.1% and 5.7%, respectively.10 The intra-subject variability for dorzagliatin Cmax and AUC0-24h are estimated to be 14.0% and 6.2%, respectively.<br>Therefore, to ensure a satisfactory DDI assessment, and assuming a drop-out rate of 20%, we plan to enroll 15 eligible subjects by aiming to obtain 12 evaluable subjects for DDI assessment.
Data exclusions	Four analysis populations were used to summarize the results from this study. Safety population includes all subjects who received study drug on Day 1; Pharmacokinetic (PK) and pharmacodynamic (PD) populations include all subjects who received study drug, had no major protocol deviations, and who had sufficient PK or PD data to obtain reliable estimates of the key PK or PD parameters; and drug-drug interaction (DDI) population includes all subjects in the PK population who completed all treatments as defined by the protocol. Of 15 enrolled subjects, one subject discontinued after treatment of sitagliptin and dorzagliatin (on Day 11) and was excluded from the DDI population. No data were excluded from safety, PK or PD populations.

Replication	This study enrolled diagnosed T2D patients in male and female aged 30-65 covering the main T2D prevalence interval.<br>The entire clinical study and blood sample collection/praparation strictly followed the standard procedures of clinical trials.<br>For the analytical methods of PK and PD samples, we followed the standard and well-validated methodology (specified the methods and process in the article and supplementary material to article).<br>Therefore, it should be replicated well following the conduction/measurement in this study.
Randomization	This is a Phase 1, open-label, single-sequence, 3-treatment study. All eligible patients received 3 treatments (sitagliptin, sitagliptin +dorzagliatin, dorzagliatin) in the same sequence in hospitalization, thus randomization is not necessary.
Blinding	This is a Phase 1, open-label, single-sequence, 3-treatment study. All eligible patients received 3 treatments (sitagliptin, sitagliptin +dorzagliatin, dorzagliatin) in the same sequence in hospitalization, thus blinding is not necessary.

Reporting for specific materials, systems and methods

We require information from authors about some types of materials, experimental systems and methods used in many studies. Here, indicate whether each material, system or method listed is relevant to your study. If you are not sure if a list item applies to your research, read the appropriate section before selecting a response.

Materials & experimental systems

n/a	Involved in the study
☒	☐ Antibodies
☒	☐ Eukaryotic cell lines
☒	☐ Palaeontology and archaeology
☒	☐ Animals and other organisms
☐	☒ Clinical data
☒	☐ Dual use research of concern

Methods

n/a	Involved in the study
☒	☐ ChIP-seq
☒	☐ Flow cytometry
☒	☐ MRI-based neuroimaging

Clinical data

Policy information about clinical studies

All manuscripts should comply with the ICMJEguidelines for publication of clinical research and a completedCONSORT checklist must be included with all submissions.

Clinical trial registration	ClinicalTrials.gov identifier: NCT03790839.
Study protocol	Protocol is provided as the supplementary information.
Data collection	The recruitment was conducted in clinical pharmacology unit in the US (Frontage Clinical Services, Inc., Secaucus, NJ) in 2019, and data collection were performed during screening, run-in period and the treatments in hospitalization.
Outcomes	The primary objectives of this study are to assess the potential PK interaction between dorzagliatin and sitagliptin and evaluate the safety and tolerability of dorzagliatin with simultaneous administration of sitagliptin in subjects with T2DM. The secondary objective of this study is to assess the pharmacodynamic (PD) responses of PD markers, such as glucose, GLP-1, and C-peptide, following dorzagliatin, sitagliptin, or simultaneous administration of dorzagliatin and sitagliptin in subjects with T2DM.<br>PK and PD assessment:<br>The non-compartmental analysis was applied to determine the PK using WinNonlin software (Certara, Princeton, NJ, USA). PK parameters were derived from plasma concentration-time curve, Cmax and Tmax were directly determined from the plasma concentration-time profile of each subject. The AUC0-24h was calculated using linear trapezoidal method.<br>PD parameters were evaluated by measurement of serum glucose, C-peptide, and plasma GLP-1 concentrations, using incremental area under curve for 4 hours, iAUC0-4h, incremental maximum concentration, iCmax, and average concentration (iCav, calculated as iAUC0-4h/4) from fasting state (at time of 0) before OGTT.<br>Safety assessment:<br>Safety evaluations were conducted throughout each study based on clinical laboratory tests, vital signs, physical examinations, 12-lead ECG, and AE. Any AE reported especially TEAE was recorded and coded using the Medical Dictionary for Drug Regulatory Activities (MedDRA), and its relationship to the drug treatment was determined by the investigator.<br>All AEs were monitored after administration of the study drugs.
